# Two Beta-Phosphorylamide Compounds as Ligands for Sm^3+^, Eu^3+^, and Tb^3+^: X-ray Crystallography and Luminescence Properties

**DOI:** 10.3390/molecules25132971

**Published:** 2020-06-28

**Authors:** Alan R. Lear, Jonah Lenters, Michael G. Patterson, Richard J. Staples, Eric J. Werner, Shannon M. Biros

**Affiliations:** 1 Department of Chemistry, Grand Valley State University, 1 Campus Dr., Allendale, MI 49401, USA; arlear@iu.edu (A.R.L.); lentersj@mail.gvsu.edu (J.L.); 2 Department of Chemistry, Biochemistry and Physics, The University of Tampa, 401 W. Kennedy Blvd., Tampa, FL 33606, USA; mgp74@cornell.edu (M.G.P.); ewerner@ut.edu (E.J.W.); 3 Center for Crystallographic Research, Department of Chemistry, Michigan State University, 578 S. Shaw Lane, East Lansing, MI 48824, USA; staples@chemistry.msu.edu

**Keywords:** X-ray crystallography, lanthanide luminescence, carbamoylmethylphosphine oxide (CMPO), lanthanide coordination chemistry

## Abstract

This paper describes the synthesis of two beta-phosphorylamide ligands and their coordination chemistry with the Ln ions Tb^3+^, Eu^3+^, and Sm^3+^. Both the ligands and Ln complexes were characterized by IR, NMR, MS, and X-ray crystallography. The luminescence properties of the Tb^3+^ and Eu^3+^ complexes were also characterized, including the acquisition of lifetime decay curves. In the solid state, the Tb^3+^ and Sm^3+^ ligand complexes were found to have a 2:2 stoichiometry when analyzed by X-ray diffraction. In these structures, the Ln ion was bound by both oxygen atoms of each beta-phosphorylamide moiety of the ligands. The Tb^3+^ and Eu^3+^ complexes were modestly emissive as solutions in acetonitrile, with lifetime values that fell within typical ranges.

## 1. Introduction

Studies into the coordination chemistry of lanthanide (Ln) metals have gained attention due to their unique chemical and photophysical properties. The vast majority of Ln metals exhibit a stable +3 oxidation state and have coordination numbers that range from 5 to 10, with values of 8 and 9 being most common [1]. Since the Ln ions′ *f*-orbitals are minimally used for metal-ligand binding, the coordination geometries are varied and often dictated by the identity of the ligands.

Most Ln metals also emit light in the ultraviolet, visible, and near-infrared regions of the electromagnetic spectrum. This “lanthanide luminescence” is most often facilitated by the incorporation of an organic molecule that binds to the metal and acts as an antenna to harvest incident light [2,3,4,5,6]. As such, Ln ligand complexes have found use in a wide variety of applications such as computer screens, probes [7,8,9], sensors [10,11], imaging agents [12,13], magnets [14], and other functional materials [15,16]. Current efforts in the field of *f*-element coordination chemistry are focused on the development of new ligands that can bind these metals in both solution and in the solid state and be useful as new materials, or in efforts to purify Ln ions from recycled materials [17,18] and ground ores [19,20,21].

This paper describes the synthesis and characterization of two new organic ligands, along with their complexes with the Ln ions Sm^3+^, Tb^3+^, and Eu^3+^. One of these ligands contains the carbamoylmethylphosphine oxide (CMPO) chelating group, which was initially developed as part of the organic compounds used in the sequestration of transuranic elements from spent nuclear fuel [22]. The other ligand contains a similar chelating group, with a phosphate ester in place of the phosphine oxide. We also discuss here the characterization of the Ln complexes of these ligands using IR and X-Ray crystallography, as well as the ability of these complexes to exhibit lanthanide luminescence.

## 2. Results and Discussion

### 2.1. Synthesis of the bis-CMPO Ligands 1 and 2, and the 1:1 Ln Complexes

**Figure 1:**
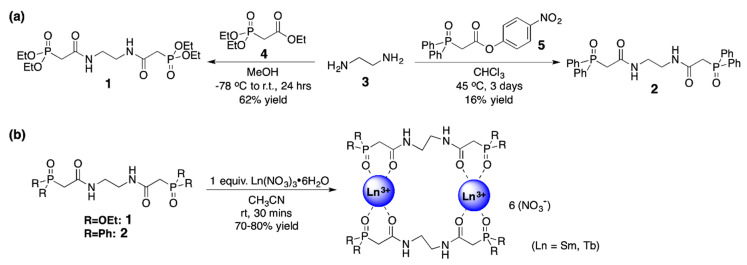
Synthetic pathways to (**a**) ligands **1** and **2**, along with (**b**) their 2:2 Ln ligand complexes. The Ln ligand complex shown in part (**b**) has been simplified for clarity; details regarding the precise coordination environments, including metal-bound nitrate groups and solvent molecules, are described with the X-ray diffraction studies.

Both organic ligands were synthesized following well-trodden paths (Figure 1) and were isolated in reasonable yields. The ethoxy-substituted ligand **1** was prepared in one step by the direct condensation of ethylenediamine **3** and triethyl phosphonoacetate **4** at high concentration in methanol [23]. Analogously, the phenyl-substituted ligand **2** was synthesized by combining ethylenediamine and the p-nitrophenol ester **5** [24]. The Ln(NO_3_)_3_-ligand complexes were prepared by stirring a 1:1 ratio of lanthanide nitrate hydrate (Ln = Sm, Eu, Tb) and the ligand in acetonitrile. The complexes were purified by removal of the volatiles and trituration of the resultant solid film with diethyl ether to give beige powders. Figure 1 depicts the stoichiometry of the product Ln ligand complexes as 2:2 based on the X-Ray structural data (vide infra).

### 2.2. Characterization of the Ligands and Complexes in the Solid State—IR Spectra and Single Crystal X-Ray Diffraction Studies

Both the ligands and their Ln complexes were characterized in the solid state by IR spectroscopy and single crystal X-Ray diffraction. The IR spectroscopy revealed shifts in both the ligand C=O and P=O stretches to smaller wavenumbers for the Ln complexes of both ligands (Δν ≈ 40 cm^−1^ and 15 cm^−1^, respectively; Table 1). This suggests that the oxygen atoms of both the carbonyl and phosphonate/phosphine oxide groups are coordinated to the metal in the solid state. For the Ln complexes of the ethoxy-substituted ligand, the frequency of the P-O stretch did not shift significantly compared to the free ligand, indicating that it is not involved in metal binding.

**Figure 2:**
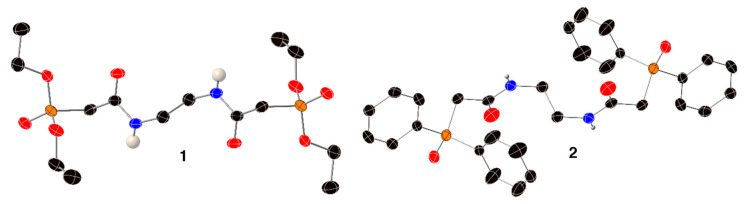
X-ray crystal structures of organic ligands **1** [25] and **2**. Thermal ellipsoids are depicted at the 50% probability level using standard CPK colors, and all hydrogen atoms bonded to carbon atoms have been omitted for clarity. Only the major component of compound **2** is shown here; details regarding the treatment of this disorder are given in the Supplementary Materials.

Single crystals of both ligands **1** [25] and **2** have been isolated and analyzed by X-ray diffraction (Figure 2). For the structure of compound **2**, the electron density corresponding to one of the phenyl rings was disordered and modeled as two orientations of the ring. Additionally, crystals of the Sm^3+^ and Tb^3+^ complexes of both ligands were also analyzed. Unfortunately, despite our best efforts, crystals of the Eu^3+^ complexes of either ligand were not isolated. The crystal data and structure refinement information for all the new structures reported here are given in Table 2, and pertinent bond lengths and angles for ligands **1** and **2**, as well as their Sm(NO_3_)_3_ and Tb(NO_3_)_3_ complexes, are given in Table 3 and Table 4. Additional structural and experimental details regarding all the new crystal structures, along with figures depicting the thermal ellipsoids of every non-hydrogen atom, can be found in the Supplementary Materials.

**Figure 3:**
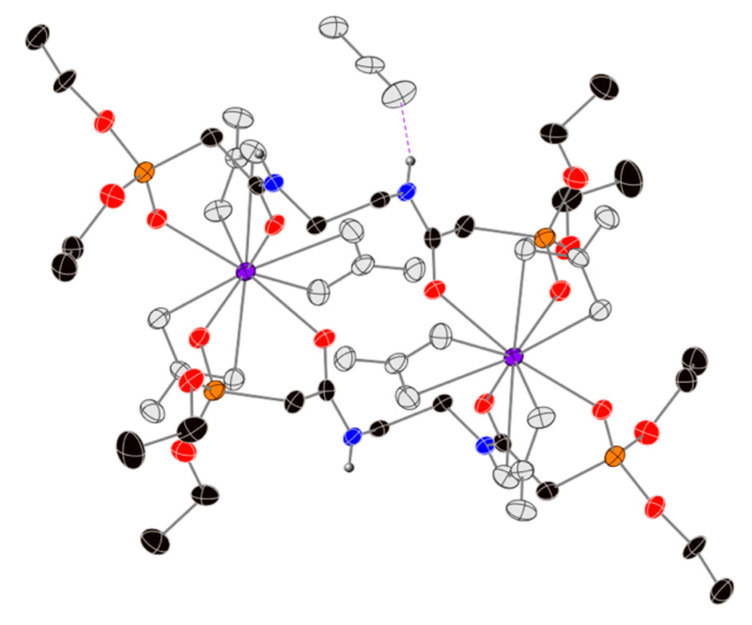
X-ray crystal structure of the [Sm(NO_3_)_3_(**1**)]**_2_**(CH_3_CN) complex, using standard CPK colors for the atoms of ligand **1** (Sm^3+^ = purple spheres). The atoms of the nitrate groups and solvent acetonitrile are depicted as grey spheres to make it easier to visualize the structure of the ligands and the metal’s coordination geometry. Thermal ellipsoids are drawn at the 40% probability level, only hydrogen atoms bonded to nitrogen atoms are shown for clarity, and the hydrogen bonding interaction is depicted with a purple, dashed line. Only the major component is shown for clarity. The Tb^3+^ complex is isomorphic to this structure, and shown in the Supplementary Materials.

The X-ray crystal structures of the Sm(NO_3_)_3_ and Tb(NO_3_)_3_ complexes with ethoxy-substituted ligand **1** reveal formations of 2:2 dimers in the solid state, where the Ln centers are bound by both the phosphonate P=O and amide C=O groups (Figure 3 shows the structure of the Sm(NO_3_)_3_ complex). In previous work, our group also reported the formation of a polymeric structure for the Sm^3+^ complex [26], which also shows the bidendate binding of the phosphate ester and amide groups. The new structures reported here are isomorphs of one another, where the inner sphere of each metal is completed with three bidentate nitrate groups to give two 10-coordinate metal centers. There is also one molecule of solvent acetonitrile that is not directly bonded to the Ln center but engaged in a hydrogen bond with one of the ligand’s amide hydrogen atoms. For both structures, the electron density corresponding to the methylene group between the P=O and C=O was disordered. This disorder was modeled over two positions, with occupancy ratios of ~0.62:~0.38 for both structures. More details about the treatment of this disorder are included in the Supplementary Materials. 

The key bond lengths and angles for the free ligand **1** and its Sm(NO_3_)_3_ and Tb(NO_3_)_3_ complexes are summarized in Table 3. The length of the ligand’s carbonyl (C=O) bond increases about 0.02 Å upon metal complexation, indicating that this group is donating electron density to the Ln center. This increase in bond length, and therefore weakening of the C=O bond, is in agreement with the observed decrease in absorption frequency for this bond in the IR data (vide supra). For the phosphonate functional group, the length of the P=O bond of the ligand remains more or less the same upon Ln complexation, with a maximum increase of 0.01 Å. This slight increase in length also agrees with the IR data discussed above. The bond angle for the beta-phosphonate amide group [C(O)-C-P(O)] is largely unchanged between the free and complexed ligand, and the O(C)-Ln-O(P) bond angles are also consistent between the Sm(NO_3_)_3_ and Tb(NO_3_)_3_ complexes at ~74°. Finally, the O-Ln bonds of the ligand are longer for the Sm(NO_3_)_3_ complex than for the Tb(NO_3_)_3_ complex, which is expected due to the larger ionic radius of Sm^3+^.

**Figure 4:**
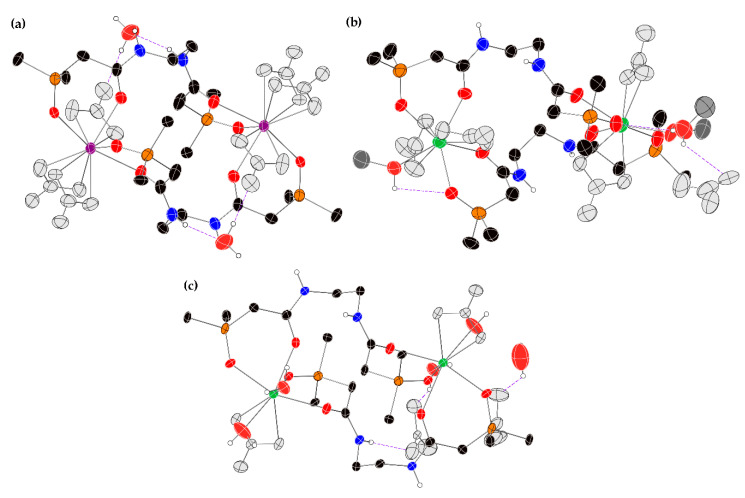
X-ray crystal structures of the 2:2 complexes between phenyl-substituted ligand **2** and Sm(NO_3_)_3_ and Tb(NO_3_)_3_ using standard CPK colors (Sm^3+^: purple, Tb^3+^: green). The atoms of the nitrate groups are colored grey in order to make it easier to visualize the ligand and metal coordination geometry. Thermal ellipsoids are drawn at the 40% probability level, and hydrogen bonding interactions are depicted with purple, dashed lines. (**a**) The [Sm(NO_3_)_3_(**2**)(H_2_O)_2_]_2_ complex; (**b**) the 2:2 [Tb(NO_3_)_3_(**2**)]_2_•H_2_O complex; (**c**) the major component of the 2:2 [Tb(NO_3_)_3_(**2**)(MeOH)]_2_ complex. For clarity, only the ipso carbon atom of each phenyl ring is shown, and only the hydrogen atoms bonded to oxygen or nitrogen atoms are shown. Complete structures are shown in the Supplementary Materials.

The X-ray crystal structures of the Sm(NO_3_)_3_ and Tb(NO_3_)_3_ complexes with phenyl-substituted ligand **2** were also solved as 2:2 dimers (Figure 4). Here, two different solvates of the Tb(NO_3_)_3_ complex were isolated and analyzed (CH_3_OH and H_2_O). In all three structures, the CMPO groups are bound in a bidentate manner to the Ln center. For the Sm(NO_3_)_3_ complex, the inner coordination sphere of the Ln ion is completed with three bidentate nitrate groups to give a 10-coordinate metal center. In the structure of the Tb(NO_3_)_3_(**2**)-methanol solvate, one inner sphere nitrate group was completely displaced by a CH_3_OH ligand to give a 9-coordinate metal. The coordination geometry of this metal center is a distorted monocapped square antiprism, where the oxygen atom O9 is considered to be the cap. A figure showing the inner coordination sphere of this metal is shown in the Supplementary Materials. For this structure, the electron density corresponding to phenyl ring C13–C28, along with the ipso P1 atom, was disordered. This disorder was modeled over two positions, with an occupancy ratio of 0.773(17) to 0.267(17). In the structure of the Tb(NO_3_)_3_(**2**)-water solvate, two inner sphere nitrates were displaced by two aqua ligands to give an 8-coordinate metal with a distorted dodecahedral geometry (see Supplementary Materials for a figure showing the atoms of the inner coordination sphere). All three Ln(NO_3_)_3_(**2**) complex structures show an extensive hydrogen bonding network between an amide hydrogen atom and a nearby nitrate group or solvent molecule. Interestingly, the only structure that contained intermolecular pi-pi interactions between the aromatic rings of the ligands was the Tb(NO_3_)_3_(**2**)-methanol solvate [27,28,29,30,31]. The key bond lengths and angles for the phenyl-substituted ligand **2** and its Sm(NO_3_)_3_ and Tb(NO_3_)_3_ complexes are summarized in Table 4. For this set of complexes, a similar set of trends regarding bond lengths and angles are observed as described for the complexes of these metals with the ethoxy-substituted ligand **1**.

### 2.3. Characterization of the Ln-Ligand Complexes in Solution Using ^1^H-NMR

**Figure 5:**
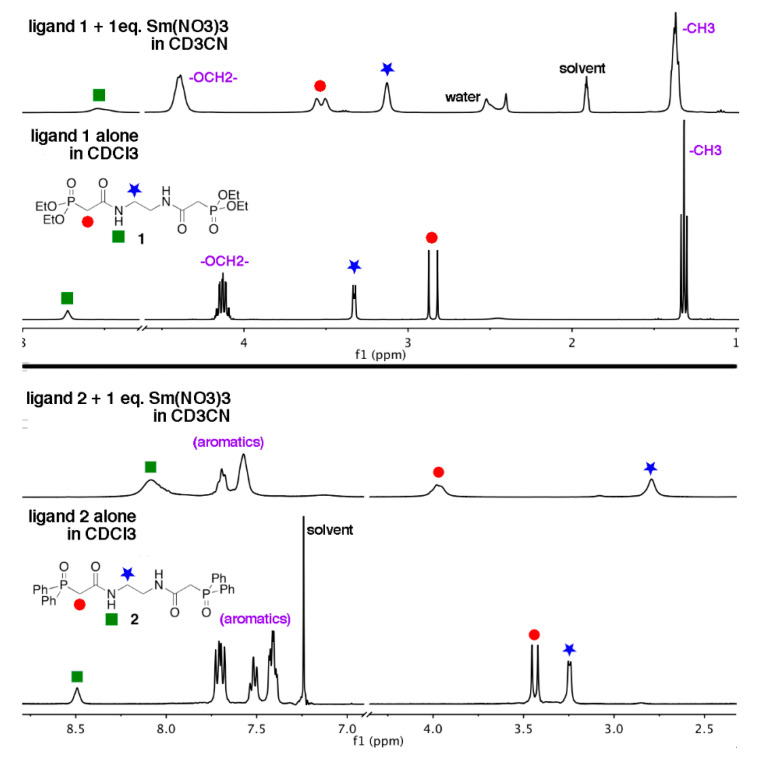
^1^H NMR spectra of ligands 1 and 2, along with their Sm(NO_3_)_3_ complexes. (**Top**): ligand **1** in CDCl_3_, and with 1 eq. Sm(NO_3_)_3_ in CD_3_CN. (**Bottom**): ligand **2** in CDCl_3_, and with 1 eq. Sm(NO_3_)_3_ in CD_3_CN. For all spectra, the key resonances have been labeled as: -N*H* (green square), -P-C*H*_2_-C- (red circle), -NH-C*H*_2_- (blue star).

The complexation of the Ln nitrates of Sm^3+^, Eu^3+^, and Tb^3+^ in solution by ligands **1** and **2** was studied by NMR spectroscopy. Initial work with DMSO-*d*_6_ as a solvent for these experiments revealed that the Lnligand complexes were not formed in this highly competitive solvent. As such, we settled on acetonitrile-*d*_3_ for the NMR studies, which parallels the results of our luminescence experiments (vide infra). For the ^1^H-NMR spectra of the Eu^3+^ and Tb^3+^ complexes, the resonances were severely broadened relative to the spectra of the ligands alone. However, the resonances in the ^1H-NMR^ spectra of the Sm^3+^ complexes were only slightly broadened and were interpretable (Figure 5).

In the ^1^H-NMR spectrum of the Sm^3+^ complex of the ethoxy-substituted ligand **1** (Figure 5, top), the resonance corresponding to the methylene group between the C=O and P=O bonds is shifted downfield relative to that of the free ligand **1** (Δδ = 0.68 ppm). The resonance corresponding to the methylene groups of the ethylenediamine cap shifts upfield 0.21 ppm for the Sm^3+^ complex relative to the free ligand. Although the magnitudes of these shifts are likely influenced by the changing solvent system (CDCl_3_ for free ligand **1**, CD_3_CN for the Sm^3+^ complex), we are confident the magnitudes of the shifts are indicative of the formation of a Sm^3+^-ligand **1** complex in the solution. Furthermore, the direction of these shifts indicates that the Sm^3+^ ion is being bound in solution by either (or both) of the C=O and P=O groups of the ligand. We attribute the upfield shift of the ethylenediamine -C*H*_2_- resonance to the proximity of this group to a paramagnetic metal center.

Information regarding the relative stability of the complexes in solution can also be gleaned by examining these spectra. The ^1^H-NMR spectrum of the Sm^3+^-ligand **1** complex displays one set of broad signals rather than separate signals for free and bound ligands. The broadness of the signals is likely from the presence of the paramagnetic Sm^3+^ ion. The presence of one set of signals indicates that the system experiences fast exchange on the ^1^H NMR timescale. As described above, our group isolated Sm^3+^-**1** dimeric complexes with a 2:2 metal-ligand stoichiometry in the solid state, but in previous work we reported the crystal structure of a metallopolymer chain having a 2:1 Sm(NO_3_)_3_-ligand **1** stoichiometry [26]. We have interpreted the combination of this solution ^1^H NMR and solid-state crystallographic data to mean that, in solutions of acetonitrile, there are varying geometries and stoichiometries of the Sm(NO_3_)_3_-ligand complexes, and they are all exchanging at a rate that is faster than the ^1^H NMR timescale. This dynamic solution is also likely present for the related Eu(NO_3_)_3_ and Tb(NO_3_)_3_ complexes.

For the ^1^H NMR spectrum of the Sm(NO_3_)_3_ complex of ligand **2** in CD_3_CN, we also see one set of relatively broad resonances, indicating that this system is experiencing a metal-ligand exchange that is fast on the ^1^H NMR timescale. Similar shifts in the resonances corresponding to the C(O)-C*H*_2_-P(O) and ethylene diamine methylene groups are observed for this ligand system, as described for ligand **1** (Δδ = +0.52 and −0.47 ppm, respectively). This indicates that, again, the Sm^3+^ ion is bound by the C=O and P=O groups rather than the amide -NH group. In the solid state, only the dimeric Ln-ligand **2** complexes were isolated by our group. However, the composition of the inner coordination sphere of the metal differed depending on the solvent used to grow the crystals (CH_3_CN vs. H_2_O vs. CH_3_OH). We propose that all the Ln-ligand **2** complexes discussed in this paper are also present in a variety of geometries and stoichiometries in solutions of acetonitrile.

### 2.4. Characterization of the Ln-Ligand Complexes in Solution Using Luminescence

The final set of experiments our research group carried out to probe the solution behavior of these Ln-ligand complexes involved the characterization of their luminescence properties. We have previously studied a variety of multipodal ligands bearing this beta-phosphonate/phosphine oxide-amide chelating group, and we have shown that these types of compounds have the ability to sensitize the luminescence of Eu^3+^ and Tb^3+^ [32,33,34]. Ligands **1** and **2** described in this paper are also able to sensitize this luminescence, albeit to a modest extent.

Most lanthanide metals (except for La^3+^ and Lu^3+^) luminesce at wavelengths in the near-IR, visible, and UV regions of the electromagnetic spectrum. In the visible region, these emission bands are quite narrow (~10 nm), which renders these metals useful for inclusion in a variety of materials [5,16], bioprobes [9,13], sensors [11,35], and metallopolymers [36]. The relatively low molar absorptivities of the Ln ions can make excitation a challenge for such applications; this can be overcome by instead exciting an appropriate bound organic ligand. To facilitate the efficient sensitization of the Ln ion and enhance the luminescence, the ligand must have an excited triplet state that is slightly higher in energy than the Ln ion emitting state (Δ*E* = 2000–4000 cm^−1^). This process was first discovered by Weissman in 1942 [37], and a simplified Jablonski diagram describing the sensitization process (or “antenna effect”) is shown in the Supplementary Materials.

**Figure 6:**
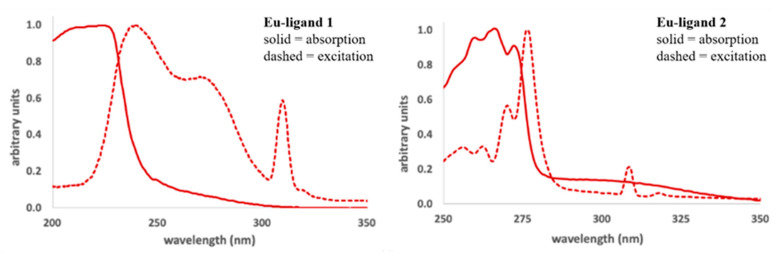
Absorption (solid line) and excitation (dashed line) spectra for the (**left**) Eu(NO_3_)_3_(**1**) and (**right**) Eu(NO_3_)_3_(**2**) complexes in acetonitrile (1.0 mM complex concentration, emission wavelengths were monitored at 620 nm for the complex with ligand **1**, and 617 nm for the complex with ligand **2**, 2.0 nm excitation and emission slit widths). Note that the intensity values of all the spectra shown here have been normalized to have their λ_max_ equal one (1.0) for ease of comparison.

We investigated the ability of ligands **1** and **2** to sensitize the luminescence of Eu^3+^ and Tb^3+^ in solutions of acetonitrile. A complex stoichiometry of 1:1 in solution was used to mimic what was observed in our solid-state X-ray diffraction studies. The absorption and excitation spectra of the Eu(NO_3_)_3_-**1** and -**2** complexes are shown in Figure 6. The absorption spectrum of ethoxy-substituted ligand **1** is broad, with a λ_max_ at 207 nm (shown in the Supplementary Materials). The λ_max_ of the UV-VIS absorption spectrum of the Eu(NO_3_)_3_(**1**)complex is red-shifted to 224 nm, and the excitation spectrum has a λ_mas_ at 240 nm. All three spectra are broad and have similar shapes. Similarly, the absorption and excitation spectra of the Eu(NO_3_)_3_(**2**) complex are similar, with λ_max_ values of 266 and 277 nm, respectively (Figure 6). The absorption spectrum of the free phenyl-substituted ligand **2** is also broad, with a λ_max_ of 224 nm (shown in Supplementary Materials). These features support the complexation of the Eu^3+^ ion by ligands **1** and **2** in solutions of acetonitrile, as well as the sensitization of metal-centered emission. The spectra of the Tb(NO_3_)_3_ complexes of both ligands **1** and **2** are similar to those of the Eu^3+^ complexes and are shown in the Supplementary Materials.

**Figure 7:**
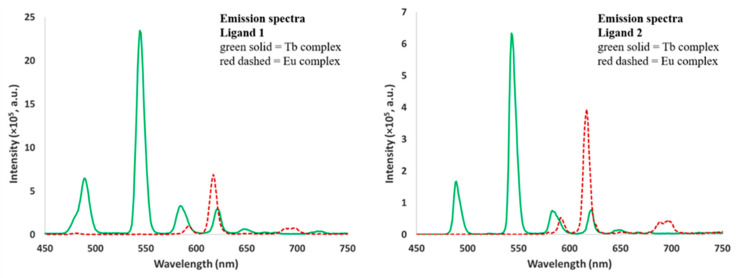
Emission spectra of the solutions of ligands **1** (**left**) and **2** (**right**), with one equivalent of Eu(NO_3_)_3_ and Tb(NO_3_)_3_ in acetonitrile. For ligand **1**: excitation wavelength 238 nm, excitation and emission slit widths = 5 nm, 2.2 mM ligand concentration; for ligand **2**: excitation wavelength 260 nm, excitation and emission slit widths = 2 nm, 1.0 mM ligand concentration. Note: for the acquisition of the emission spectra of the ligand **1** complexes, the complex concentration is more than twice that of ligand **2**, and the excitation and emission slits were widened to accommodate the dimmer emission of these complexes.

The emission spectra for 1:1 solutions of ligands **1** and **2** with Eu(NO_3_)_3_ and Tb(NO_3_)_3_ in acetonitrile are shown in Figure 7. Solutions containing the Ln complexes of ligand **1** were excited at 238 nm, while the complexes of ligand **2** were excited at 260 nm. In both cases, characteristic emission bands are observed [16], with the Tb^3+^ complexes being brighter than the Eu^3+^ complexes. The spectra of the Tb^3+^ complexes are nearly identical, with the ^5^D_4_→^7^F*_J_* (*J* = 6, 5, 4, 3) transitions appearing at 489, 544, 585, and 622 nm, respectively. The spectra of the Eu^3+^ complexes are also quite similar, with peaks at 593 and 617 nm, corresponding to the ^5^D_0_→^7^F*_J_* transitions (*J* = 1, 2).

The luminescence decay lifetimes for each of the 1:1 Ln-ligand complexes of the phenyl-substituted ligand **2** were acquired in acetonitrile. The lifetime values shown in Table 5 are from fits of the data to a single exponential decay. The decay lifetimes of 1.35 and 1.83 ms were obtained for the Eu^3+^ and the Tb^3+^ complex of ligand **2**, respectively. We did not measure the luminescence decay lifetimes for complexes of the ethoxy-substituted ligand **1** because they were substantially dimmer than those with the phenyl-substituted ligand **2**.

## 3. Scope and Outlook

We have described here the characterization of two compounds bearing two-bidentate chelating groups as ligands for the Ln nitrate salts of Sm^3+^, Eu^3+^, and Tb^3+^. These organic compounds feature a relatively flexible ethylenediamine backbone, with hard (albeit neutral) oxygen donor atoms to facilitate Ln binding. As such, the Ln-ligand complexes are dynamic in solution, as evidenced by the ^1^H NMR data and varied solid state X-ray structures presented here. These ligands are also able to sensitize the luminescence of Eu(NO_3_)_3_ and Tb(NO_3_)_3_, showing a modest emission in solutions of acetonitrile. The impacts of straightforward derivations of the ligand backbone on these properties would be an interesting next set of experiments. For instance, would complex stability be increased with a more rigid backbone? Would the metal-centered luminescence be brighter with a conjugated backbone? These are questions we will continue to investigate to further expand the scope of such ligands for lanthanide coordination.

## 4. Materials and Methods

### 4.1. General Considerations

All the chemicals (including deuterated solvents) were used as purchased from Sigma-Aldrich (St. Louis, MO, USA) or Strem Chemical (Newburyport, MA, USA) and used without further purification. The ^1^H, ^13^C, and ^31^P-NMR spectral data were recorded on either a JEOL ECZS 400 (Peabody, MA, USA) or Varian Inova 400 FTNMR spectrophotometer. For the ^1^H and ^13^C-NMR spectra, chemical shifts are expressed as parts per million (δ) relative to SiMe_4_ (TMS, δ = 0) and referenced internally with respect to the protio solvent impurity. For the ^31^P NMR spectra, the chemical shifts are expressed as parts per million (δ) relative to H_3_PO_4_ (δ = 0). Both the ^13^C and ^31^P-NMR spectra were obtained as proton-decoupled data. The IR spectra were acquired neat on a Jasco 4100 FTIR (Easton, MD, USA). The elemental (CHN) analyses were performed by Atlantic Microlab Inc., Norcross, GA; all CHN percentages calculated for the lanthanide complexes assume one CMPO ligand + Ln(NO_3_)_3_ + residual water/solvents as indicated. The mass spectrometry data were acquired by the Lumigen Instrument Center at Wayne State University (Detroit, MI, USA). The absorption spectra were acquired on a Shimadzu UV-2450 (West Chicago, IL, USA) or Agilent 8453 UV-VIS spectrophotometer (Santa Clara, CA, USA). The luminescence data were collected on either a Horiba Fluoromax 4 (Kyoto, Japan) or a Hitachi F-7000 (Chiyoda City, Tokyo, Japan) spectrophotometer. For the determination of lifetime values, the decay curve was fit to a single exponential decay using the Horiba FluorEssence software package. These curves, along with the residuals, are shown in the Supplementary Materials.

### 4.2. Single Crystal X-Ray Crystallography

The crystals suitable for X-ray diffraction were mounted on a nylon loop using a small amount of paratone oil. Data were collected using a Bruker CCD (charge coupled device)-based diffractometer equipped with an Oxford Cryostream low-temperature apparatus operating at 173(2) K. The data were measured using omega and phi scans of 0.5° per frame. The total number of images was based on results from the program COSMO [38], where the redundancy was expected to be 4.0 and a completeness of 100% out to 0.83 Å. The cell parameters were retrieved using the APEX II software [39] and refined using SAINT on all observed reflections. The data reduction was performed using the SAINT software [40], which corrects for Lp. Scaling and absorption corrections were applied using the SADABS [41] multi-scan technique, supplied by George Sheldrick. The structures were solved by the direct method using the SHELXS-97 program and refined by least squares method on F^2^, SHELXL-2014 [42], which are incorporated into OLEX2 [43,44]. All the non-hydrogen atoms were refined anisotropically. Unless otherwise noted, the locations of the hydrogen atoms were calculated by geometrical methods and refined as a riding model. The crystals used for the diffraction studies showed no decomposition during data collection. Further crystallographic data and experimental details for the structural analysis of all the complexes are summarized in Table 2, and the selected bond lengths and angles with their estimated standard deviations are given in Table 3 and Table 4. For each structure reported here the Supplementary Information file contains diagrams depicting the thermal ellipsoids, complete experimental tables, and descriptions of how any disordered electron density was treated.

### 4.3. Photophysical Studies

All the luminescence studies were carried out with a 1:1 ratio of Ln(NO_3_)_3_•xH_2_O to ligands **1** and **2** in chromasolv grade CH_3_CN, or HPLC grade CH_3_OH. The solutions of complexes were prepared by either (a) dissolving solid ligand into an appropriate volume of solvent and adding solid metal or (b) combining appropriate volumes of ligand and metal stock solutions. Metal complex solutions were prepared immediately before analysis and were all at an overall 1.0 mM concentration of the 1:1 Ln-**1** and Ln-**2** complexes. The emission spectra were corrected for the varying sensitivity of the detector by applying the manufacturer’s protocols using the Horiba FluorEssence software package.

### 4.4. Synthesis

**Ligand 1.** This synthetic procedure represents an alternative to what was previously published by our group [25]. Ethylene diamine (1.0 mL, 0.015 mol) was dissolved in 8.3 mL methanol in a 25 mL round bottom flask. The mixture was cooled to −78 °C, and 8.9 mL (0.045 mol) triethyl phosphonoacetate **4** was added dropwise. The reaction mixture was allowed to warm to room temperature and stirred overnight. The solution was poured into methylene chloride (150 mL) and was extracted with 1M HCl (3 × 25 mL), saturated NaHCO_3_ (3 × 25 mL), and brine (1 × 25 mL). The aqueous layers were combined, and the product was back extracted with chloroform (3 × 25 mL). The volatiles were removed under reduced pressure to give 0.59 mg of pure ligand **1** (62%) as an off-white solid. IR: ν (cm^−1^): 3268 (N-H), 1666 (C=O), 1203 (P=O), 1016 (C-O), 963 (C-O); UV-VIS (1.0 mM, CH_3_CN): λ_max_ 207 nm; ^1^H NMR (300 MHz, CDCl_3_): δ 7.73 (broad, 2H, -N*H*), 4.14 (q, *J* = 7.0 Hz, 8H), 3.34 (d, *J* = 5.9 Hz, 4H), 2.85 (d, *J*_P-H_ = 15.8 Hz, 4H), 1.33 (t, *J* = 7.0 Hz, 12H); ^13^C NMR (75 MHz, CDCl_3_): δ 165.4, 62.9, 35.8 (d, *J*_P-C_ = 128 Hz), 16.5; ^31^P NMR (121 MHz, CDCl_3_): δ 24.5; HR-MS (ESI): expected for C_14_H_30_N_2_O_8_P_2_Na^+^: 439.1370; found: 439.1367. The X-ray crystal structure of this compound has been reported [25].

**Ligand 2.** Ethylenediamine (0.135 mL, 0.002 mol) and the *p*-nitrophenol ester **5** [24] (2.32 g, 0.006 mol) were dissolved in anhydrous amylene-stabilized chloroform (42.5 mL) and stirred in a round bottom flask equipped with a reflux condenser under an atmosphere of nitrogen. The reaction mixture was heated to 45 °C and stirred for three days. After the solution was allowed to cool to room temperature, a few cm^3^ of 40% KOH was added and the reaction was stirred for four hours. The organic layer was separated and washed with 5% sodium carbonate (30 mL) and water (3 × 30 mL) and dried over anhydrous MgSO_4_. The organic volatiles were removed under reduced pressure to give a solid, which was triturated with ethyl acetate (3 × 5 mL). The off-white solid was placed under a high vacuum overnight to give the pure product (0.38 g, 16% yield). IR: ν = 1661 cm^−1^ (C=O), 1173 cm^−1^ (P=O). UV-VIS (1.0 mM, CH_3_CN): λ_max_ 224 nm (note: there was undissolved ligand in the cuvette for this analysis; ligand **2** is poorly soluble in CH_3_CN); ^1^H NMR (400 MHz, CDCl_3_): δ = 8.49 (broad s, 2H, N*H*), 7.72 (m, 8H), 7.69 (m, 8H), 7.55 (m, 4H), 3.45 (d, *J*_P-H_ = 13.6 Hz, 4H), 3.26 (m, 4H); ^31^C NMR (100 MHz, CDCl_3_): δ = 165.4 (d, *J*_P-C_ = 5.9 Hz), 132.3 (d, *J*_P-C_ = 2.8 Hz), 131.8 (d, *J*_P-C_ = 103 Hz), 131.1 (d, *J*_P-C_ = 10.1 Hz), 128.8 (d, *J*_P-C_ = 12.2 Hz), 39.6 (d, *J*_P-C_ = 60.1 Hz), 38.4 (s); ^31^P NMR (161 MHz, CDCl_3_): δ = 32.1. HR-MS (ESI): expected for C_30_H_30_N_2_O_4_P_2_H^+^: 545.1754; found: 545.1755. Crystals suitable for X-ray diffraction were grown by the vapor diffusion of diethyl ether into a solution of the Tb complex in methanol.

**Ln-ligand complexes.** A general procedure for the synthesis of all the Ln-ligand complexes is described below. Note that ligand **2** is poorly soluble in acetonitrile, but upon the addition of the Ln(NO_3_)_3_ to the reaction mixture the ligand dissolved readily. Compound **1** or **2** (50 mg) was added to a round bottom flask and dissolved in 10–15 mL of acetonitrile. One molar equivalent of the lanthanide nitrate hydrate was added as a solid and the reaction mixture was stirred for thirty minutes at room temperature. The solvent was removed under reduced pressure to give a thin clear film. Approximately 5 mL of diethyl ether was added to the flask and the film was scraped away from the glass using a metal spatula to give an off-white powder. The ether was removed with a Pasteur pipette, and the remaining solid was triturated one or two more times with fresh ether (~5 mL). The metal-ligand complexes were placed under high vacuum overnight to give the products in isolated yields that ranged from 70–80%. The characterization data for each Ln-ligand complex are given below; the ^1^H NMR spectra of the Eu^3+^ and Tb^3+^ complexes were quite broad and are not reported.

**Sm-1(NO_3_)_3_.** IR: ν (cm^−1^) = 3317 (N-H), 1626 (C=O), 1191 (P=O), 1014 (C-O); ^1^H NMR (CD_3_CN, 400 MHz, integration values are not reliable for this complex so they have not been reported): δ 7.54 (broad), 4.39 (broad s), 3.53 (d, *J*_P-H_ = 20.8 Hz), 3.13 (broad s), 1.37 (broad t, *J* = 3.7 Hz); ^13^C NMR (CD_3_CN, 100 MHz): δ 168.3 (d, *J*_P-c_ = 41 Hz), 65.3 (s), 39.6 (s), 32.9 (d, *J*_P-C_ = 139 Hz), 15.6 (s); ^31^P NMR (CD_3_CN, 161 MHz): δ 24.4; ESI-LRMS (M^+^, *m/z*): calcd for (C_14_H_30_N_2_O_8_P_2_)Sm(NO_3_)_2_: 692, found 692; calcd for (C_14_H_30_N_2_O_8_P_2_)_2_Sm(NO_3_)_2_: 1108, found 1108; Anal. calcd. for (C_14_H_30_N_2_O_8_P_2_)Sm(NO_3_)_3_(CH_3_CN)(C_4_H_10_O) (found): C, 24.44 (24.16); H, 4.41 (4.23); N, 8.55 (8.19). Crystals suitable for analysis by X-ray diffraction were grown by the vapor diffusion of diethyl ether into a solution of the complex in acetonitrile. We note here that the crystal used to collect data was quite large and refracted exceptionally well. Because of this, the absorption correction was not adequate, and there are large residual positive and negative electron density peaks in the model.

**Eu-1(NO_3_)_3_.** IR: ν (cm^−1^) = 3328 (N-H), 1627 (C=O), 1191 (P=O), 1013 (C-O); ESI-LRMS (M^+^, *m/z*): calcd for (C_14_H_30_N_2_O_8_P_2_)Eu(NO_3_)_2_: 693, found 693; calcd for (C_14_H_30_N_2_O_8_P_2_)_2_Eu(NO_3_)_2_: 1109, found 1109; Anal. calcd. for (C_14_H_30_N_2_O_8_P_2_)Eu(NO_3_)_3_ (found): C, 22.29 (22.69); H, 4.01 (4.23); N, 9.28 (8.75).

**Tb-1(NO_3_)_3_.** IR: ν (cm^−1^) = 3321 (N-H), 1627 (C=O), 1191 (P=O), 1014 (C-O); ESI-LRMS (M^+^, *m/z*): calcd for (C_14_H_30_N_2_O_8_P_2_)_2_Tb(NO_3_)_2_: 1115, found 1115; Anal. calcd. for (C_14_H_30_N_2_O_8_P_2_)Tb(NO_3_)_3_ (found): C, 22.09 (22.46); H, 3.97 (4.30); N, 9.20 (8.71). Crystals suitable for analysis by X-ray diffraction were grown by the vapor diffusion of diethyl ether into a solution of the complex in acetonitrile.

**Sm-2(NO_3_)_3_.** IR: ν (cm^−1^) = 1623 (C=O), 1157 (P=O); ^1^H NMR (CD_3_CN, 400 MHz, all the resonances are broad, and integration values are not reliable for this complex so they have not been reported): δ 8.08 (N*H*), 7.80-7.50, 3.97, 2.79; ^31^P NMR (161 MHz, CD_3_CN): δ 39.4 (broad); ^13^C NMR data was not obtained for this complex due to the relatively low solubility in CD_3_CN. ESI-LRMS (M^+^, *m/z*): calcd for (C_30_H_30_N_2_O_4_P_2_)_2_ Tb(NO_3_)_2_: 1364, found 1364; Anal. calcd. for (C_30_H_30_N_2_O_4_P_2_)Sm(NO_3_)_3_(CH_3_CN)(C_4_H_10_O) (found): C, 38.91 (39.20); H, 3.90 (3.61); N, 7.56 (7.38). Crystals suitable for analysis by X-ray diffraction were grown by the slow evaporation of a CDCl_3_ solution in an NMR tube.

**Eu-2(NO_3_)_3_.** IR: ν (cm^−1^) = 1621 (C=O), 1156 (P=O); ESI-LRMS (M^+^, *m/z*): calcd for (C_30_H_30_N_2_O_4_P_2_)_2_Eu(NO_3_)_2_: 1365, found 1365; Anal. calcd. for (C_30_H_30_N_2_O_4_P_2_)Eu(NO_3_)_3_(H_2_O) (found): C, 37.75 (38.00); H, 3.59 (3.46); N, 7.34 (7.26).

**Tb-2(NO_3_)_3_.** IR: ν (cm^−1^) = 1621 (C=O), 1156 (P=O); ESI-LRMS (M^+^, *m/z*): calcd for (C_30_H_30_N_2_O_4_P_2_) Tb(NO_3_)_2_: 827, found 827; calcd for (C_30_H_30_N_2_O_4_P_2_)_2_Tb(NO_3_)_2_: 1371, found 1371; Anal. calcd. for (C_30_H_30_N_2_O_4_P_2_)Tb(NO_3_)_3_(H_2_O) (found): C, 37.49 (37.45); H, 3.57 (3.31); N, 7.29 (7.11). Crystals suitable for analysis by X-ray diffraction were grown by the vapor diffusion of diethyl ether into a solution of the complex in acetonitrile.

## Figures and Tables

**Table 1:** FT-IR data of ligands **1** and the resultant Ln complexes; peak values reported in wavenumbers (cm^−1^).

Stretch	Ligand 1	Sm-1	Eu-1	Tb-1	Ligand 2	Sm-2	Eu-2	Tb-2
C=O	1666	1626	1627	1627	1661	1623	1621	1621
P=O	1203	1191	1191	1191	1173	1157	1156	1156
P-O	1016	1014	1013	1014	-----	-----	-----	-----
N-H	3268	3317	3328	3321	3271	weak	weak	weak

**Table 2:** Crystal data and structure refinement for free ligand **2**, and the metal complexes [Sm(NO_3_)_3_(**1**)]_2_•(CH_3_CN), [Tb(NO_3_)_3_(**1**)]_2_•(CH_3_CN), [Sm(NO_3_)_3_(**2)**]_2_**•**H_2_O, and [Tb(NO_3_)_3_(**2**)(H_2_O)_2_]_2_, [Tb(NO_3_)_3_(**2)**(MeOH)]_2_.

Structure Number	2	[Sm(NO_3_)_3_(1)]_2_• (CH_3_CN)	[Tb(NO_3_)_3_(1)]_2_•(CH_3_CN)	[Sm(NO_3_)_3_(2)]_2_•H_2_O	[Tb(NO_3_)_3_(2)(H_2_O)_2_]_2_	[Tb(NO_3_)_3_(2)(MeOH)]_2_
CCDC number	2003372	2003370	2003373	2003369	2003374	2003371
Empirical formula	C_30_H_30_N_2_O_4_P_2_	C_32_H_66_N_12_O_34_P_4_Sm_2_	C_32_H_66_N_12_O_34_P_4_Tb_2_	C_60_H_64_N_10_O_28_P_4_Sm_2_	C_60_H_72_N_10_O_32_P_4_Tb_2_	C_64_H_76_N_10_O_30_P_4_Tb_2_
Formula weight	544.50	1587.54	1604.68	1797.79	1886.99	1907.06
Temperature/K	173(2)	173(2)	173(2)	173(2)	173(2)	173(2)
Crystal system	monoclinic	monoclinic	monoclinic	monoclinic	triclinic	tetragonal
Space group	P2_1_/c	C2/c	C2/c	P2_1_/n	P-1	P-42_1_c
a/Å	5.65550(10)	32.318(11)	32.3189(5)	10.5235(15)	11.438(2)	18.2898(4)
b/Å	28.8994(5)	12.538(4)	12.5255(2)	18.133(3)	12.724(2)	18.2898(4)
c/Å	8.4517(2)	16.447(6)	16.4158(3)	19.488(3)	14.681(3)	27.4073(6)
α/°	90	90	90	90	91.784(2)	90
β/°	109.4290(10)	116.217(4)	116.1620(10)	96.944(12)	106.593(2)	90
γ/°	90	90	90	90	113.139(2)	90
Volume/Å^3^	1302.69(5)	5979(4)	5964.48(18)	3691.6(9)	1857.8(6)	9168.2(4)
Z	2	4	4	2	1	4
ρ_calc_g/cm^3^	1.388	1.764	1.787	1.617	1.687	1.382
μ/mm^−1^	1.848	2.154	13.430	13.393	2.068	8.794
F(000)	572.0	3192.0	3216.0	1804.0	948.0	3840.0
Crystal size/mm^3^	0.169 × 0.093 × 0.035	0.279 × 0.218 × 0.082	0.264 × 0.173 × 0.103	0.262 × 0.126 × 0.063	0.187 × 0.11 × 0.038	0.284 × 0.2 × 0.183
Radiation	CuKα (λ = 1.54178)	MoKα (λ = 0.71073)	CuKα (λ = 1.54178)	CuKα (λ = 1.54178)	MoKα (λ = 0.71073)	CuKα (λ = 1.54178)
2Θ range for data collection/°	6.116 to 136.61	3.54 to 52.802	6.094 to 140.374	6.68 to 143.44	3.528 to 50.95	5.81 to 136.676
Index ranges	−6 ≤ h ≤ 6, −34 ≤ k ≤ 34, −10 ≤ l ≤ 10	−40 ≤ h ≤ 40, −15 ≤ k ≤ 15, −20 ≤ l ≤ 20	−39 ≤ h ≤ 39, −15 ≤ k ≤ 15, −17 ≤ l ≤ 19	−12 ≤ h ≤ 12, −22 ≤ k ≤ 22, −23 ≤ l ≤ 21	−13 ≤ h ≤ 13, −12 ≤ k ≤ 15, −17 ≤ l ≤ 16	−22 ≤ h ≤ 22, −21 ≤ k ≤ 22, −33 ≤ l ≤ 33
Reflections collected	17,978	26,038	44,653	24,709	18,678	111,590
Independent reflections	2388 [R_int_ = 0.0757, R_sigma_ = 0.0373]	6120 [R_int_ = 0.0770, R_sigma_ = 0.0704]	5582 [R_int_ = 0.0588, R_sigma_ = 0.0348]	6996 [R_int_ = 0.1460, R_sigma_ = 0.1397]	6825 [R_int_ = 0.0585, R_sigma_ = 0.0735]	8408 [R_int_ = 0.1335, R_sigma_ = 0.0634]
Data/restraints/parameters	2388/0/213	6120/1/402	5582/0/402	6996/48/472	6825/0/492	8408/165/536
Goodness-of-fit on F^2^	1.022	1.058	1.086	1.020	1.024	1.094
Final R indexes [I> = 2σ (I)]	R_1_ = 0.0468, wR_2_ = 0.1144	R_1_ = 0.0532, wR_2_ = 0.1296	R_1_ = 0.0287, wR_2_ = 0.0636	R_1_ = 0.0722, wR_2_ = 0.1524	R_1_ = 0.0626, wR_2_ = 0.1578	R_1_ = 0.0638, wR_2_ = 0.1895
Final R indexes [all data]	R_1_ = 0.0610, wR_2_ = 0.1234	R_1_ = 0.0776, wR_2_ = 0.1487	R_1_ = 0.0336, wR_2_ = 0.0653	R_1_ = 0.1453, wR_2_ = 0.1815	R_1_ = 0.0791, wR_2_ = 0.1717	R_1_ = 0.0827, wR_2_ = 0.2052
Largest diff. peak/hole/e Å^−3^	0.67/−0.34	3.54/−0.79	0.57/−0.75	0.91/−0.91	3.52/−1.60	1.10/−0.77
						0.023(4)

**Table 3:** Selected bond distances (Å) and angles (°) for ligand **1**[25] and its Sm(NO_3_)_3_ (dimeric), Sm(NO_3_)_3_ (polymeric) [26], and Tb(NO_3_)_3_ complexes.

	Ligand 1 [25]	[Sm(NO_3_)_3_(1)]_2_• (CH_3_CN)	[Sm(NO_3_)_3_(1)]_2_(polymer) [26]	[Tb(NO_3_)_3_(1)]_2_• (CH_3_CN)
ligand				
C=O	1.226(2)	1.242(7), 1.242(7)	1.245(5), 1.249(4)	1.246(4), 1.245(4)
P=O	1.474(2)	1.473(4), 1.478(4)	1.482(3), 1.485(3)	1.477(2), 1.478(3)
P-O	1.5791(16), 1.5619(15)	1.551(5), 1.552(5), 1.551(5), 1.563(5)	1.515(4), 1.537(4), 1.553(3), 1.542(3)	1.554(2), 1.560(3), 1.564(3), 1.561(3)
Ln-O(C)	-----	2.387(4), 2.417(4)	2.387(3), 2.382(3)	2.378(2), 2.400(2)
Ln-O(P)	-----	2.400(4), 2.434(4)	2.344(3), 2.340(3)	2.390(2), 2.424(2)
C(O)-C-P(O)	110.9(2)	110.6(4), 113.1(4)	109.7(3), 110.1(3)	110.4(2), 111.2(2)
O(C)-Ln-O(P)	-----	75.24(14), 73.53(15)	74.17(10), 74.70(9)	75.44(8), 74.06(8)
inner sphere water				
Ln-O(water)	-----	-----	2.413(3)	-----

**Table 4:** Selected bond distances (Å) and angles (°) for ligand **2** and its Sm(NO_3_)_3_ and Tb(NO_3_)_3_ complexes. For the Tb(NO_3_)_3_ complex, only the atoms that were not disordered are included here.

	Ligand 2	[Sm(NO_3_)_3_(2)]_2_•H_2_O	[Tb(NO_3_)_3_(2)(MeOH)]_2_	[Tb(NO_3_)_3_(2)(H_2_O)_2_]_2_
ligand				
C=O	1.224(3)	1.251(11), 1.253(13)	1.245(15), 1.255(14)	1.262(8), 1.266(9)
P=O	1.4912(18)	1.511(7), 1.508(7)	1.505(9)	1.511(5), 1.509(5)
Ln-O(C)	-----	2.514(7), 2.396(7)	2.365(8), 2.372(8)	2.412(5), 2.352(5)
Ln-O(P)	-----	2.360(7), 2.387(7)	2.332(8)	2.277(5), 2.310(5)
C(O)-C-P(O)	111.80(17)	113.0(7), 115.0(8)	111.7(9)	112.8(5), 111.3(5)
O(C)-Ln-O(P)	-----	76.1(2), 74.5(2)	75.3(3)	79.05(17), 77.64(18)
inner sphere water				
Ln-O(water)	-----	-----	-----	2.356(5), 2.414(6)

**Table 5:** Emission lifetimes for 1:1 complexes of Eu(NO_3_)_3_ and Tb(NO_3_)_3_ with ligand **2** in acetonitrile. The error bars on these numbers represent the standard deviation from three trials.

Ligand 2 Complex	τ_ACN_/ms
Eu	1.35 ± 0.08 (at 616 nm)
Tb	1.83 ± 0.05 (at 545 nm)

## Supplementary Materials

(1) X-ray crystallography: full refinement details, tables of bond lengths and angles, figures of each structure depicting the thermal ellipsoids and atom numbering scheme, and descriptions of how any disordered electron density was treated; (2) Luminescence data: all UV-VIS absorption and excitation spectra, as well as all lifetime decay curves. All the crystallographic data has been submitted to the Cambridge Structural Database with CCDC numbers 2003369-2003374. These files can be accessed free of charge at: www.ccdc.cam.ac.uk.
