# Supplementary material for: Two Beta-Phosphorylamide Compounds as Ligands for Sm3+, Eu3+, and Tb3+: X-ray Crystallography and Luminescence Properties
Source: Molecules. 2020 Jun 28;25(13):2971. doi: 10.3390/molecules25132971 (PMC7411983; doi:10.3390/molecules25132971)
Supplement: Supplementary file 1 [file molecules-25-02971-s001.zip › supp_files_rev2/Lear_Molecules_SI_rev2.pdf]

# Two beta-phosphorylamide compounds as ligands for $Sm^{3+}$, $Eu^{3+}$ and $Tb^{3+}$: X-ray crystallography and luminescence properties

# Supplementary Materials

**Alan R. Lear[1], Jonah Lenters[1], Michael G. Patterson[2], Richard J. Staples[3], Eric J. Werner[2], and Shannon M. Biros[1]***

[1] Department of Chemistry, Grand Valley State University, 1 Campus Dr., Allendale, MI 49401, USA
[2] Department of Chemistry, Biochemistry and Physics, The University of Tampa, 401 W. Kennedy Blvd., Box U, Tampa, FL 33606, USA
[3] Center for Crystallographic Research, Department of Chemistry, Michigan State University, 578 S. Shaw Lane, East Lansing, MI 48824, USA
* Correspondence: biross@gvsu.edu; Tel.: +1-616-331-8955

## Table of Contents

**I. X-Ray Crystallography**

**A. Ligand 2……………………………………………………………………………………………2**

**B. Sm(NO$_3$)$_3$-2…………………………………………………………………………..……..7**

**C. Tb(NO$_3$)$_3$-2 (MeOH solvate) …………………………………………………………………16**

**D. Tb(NO$_3$)$_3$-1……………………………………………………………………………..….26**

**E. Sm(NO$_3$)$_3$-1……………………………………………………………………………..….33**

**F. Tb(NO$_3$)$_3$-2 (water solvate) ………………………………………………………………....41**

**2. Luminescence Data**

**A. Abbreviated Jablonski diagram…………………………………………………………………50**

**A. Absorption and excitation spectra………………………………………………………………50**

**B. Lifetime decay curves…………………………………………………………………………..…53**

# I. X-Ray crystallography

## A. Ligand 2

This figure shows the complete molecular structure of compound **1** using standard CPK colors, with thermal ellipsoids at the 40% probability level. The atom labeling scheme for one unique set of atoms is also depicted here, and only hydrogen atoms bonded to nitrogen atoms have been shown for clarity. Only the major component of ring C10-C15 is shown here.

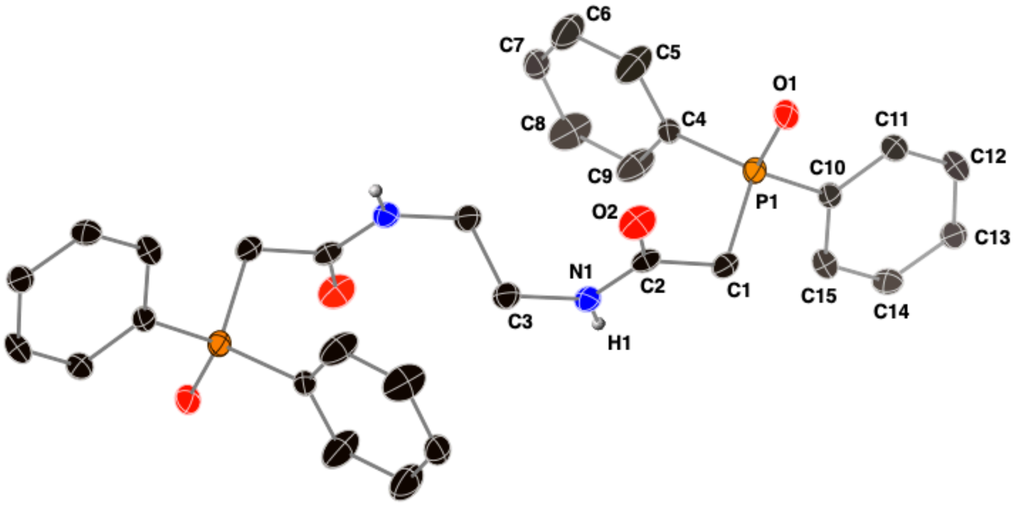


**Refinement notes**
(1) Hydrogen atom (O)H1 was located using electron density difference maps and refined.
(2) The electron density corresponding to the aromatic ring C10-C15 was disordered and modeled over two positions with a relative occupancy ratio of 0.499(4) to 0.501(4) by refining against a free variable. No restraints were needed.

**Experimental**

Single crystals of $C_{30}H_{30}N_2O_4P_2$ compound **2** were grown by vapor diffusion of diethyl ether into a solution of the Eu-ligand complex in methanol and used as received. A suitable crystal was selected and mounted on a nylon loop using a small amount of paratone oil on a 'Bruker APEX-II CCD' diffractometer. The crystal was kept at 173(2) K during data collection. Using Olex2 [1], the structure was solved with the ShelXT [2] structure solution program using Intrinsic Phasing and refined with the ShelXL [3] refinement package using Least Squares minimization.

1. Dolomanov, O.V., Bourhis, L.J., Gildea, R.J, Howard, J.A.K. & Puschmann, H. (2009), J. Appl. Cryst. 42, 339-341.
2. Sheldrick, G.M. (2015). Acta Cryst. A71, 3-8.
3. Sheldrick, G.M. (2015). Acta Cryst. C71, 3-8.

**Table 1 Crystal data and structure refinement for ligand 2.**

| | |
|---|---|
| CCDC number | 2003372 |
| Empirical formula | $C_{30}H_{30}N_2O_4P_2$ |
| Formula weight | 544.50 |
| Temperature/K | 173(2) |
| Crystal system | monoclinic |
| Space group | $P2_1/c$ |
| a/Å | 5.65550(10) |
| b/Å | 28.8994(5) |
| c/Å | 8.4517(2) |

| | |
|---|---|
| α/° | 90 |
| β/° | 109.4290(10) |
| γ/° | 90 |
| Volume/Å$^3$ | 1302.69(5) |
| Z | 2 |
| $\rho_{calc}$g/cm$^3$ | 1.388 |
| μ/mm$^{-1}$ | 1.848 |
| F(000) | 572.0 |
| Crystal size/mm$^3$ | 0.169 × 0.093 × 0.035 |
| Radiation | CuKα (λ = 1.54178) |
| 2Θ range for data collection/° | 6.116 to 136.61 |
| Index ranges | -6 ≤ h ≤ 6, -34 ≤ k ≤ 34, -10 ≤ l ≤ 10 |
| Reflections collected | 17978 |
| Independent reflections | 2388 [$R_{int}$ = 0.0757, $R_{sigma}$ = 0.0373] |
| Data/restraints/parameters | 2388/0/213 |
| Goodness-of-fit on F$^2$ | 1.022 |
| Final R indexes [I>=2σ (I)] | $R_1$ = 0.0468, w$R_2$ = 0.1144 |
| Final R indexes [all data] | $R_1$ = 0.0610, w$R_2$ = 0.1234 |
| Largest diff. peak/hole / e Å$^{-3}$ | 0.67/-0.34 |

**Table 2 Fractional Atomic Coordinates (×10$^4$) and Equivalent Isotropic Displacement Parameters (Å$^2$×10$^3$) for LIGAND 2. $U_{eq}$ is defined as 1/3 of of the trace of the orthogonalised $U_{IJ}$ tensor.**

| Atom | *x* | *y* | *z* | U(eq) |
|---|---|---|---|---|
| P1 | 4503.2(11) | 6439.5(2) | 5051.3(8) | 24.3(2) |
| O1 | 7249(3) | 6365.2(6) | 5448(2) | 32.5(4) |
| O2 | 4800(4) | 5450.7(7) | 6928(3) | 41.7(5) |
| N1 | 619(4) | 5567.5(7) | 6115(3) | 26.4(5) |
| C1 | 3307(4) | 6231.8(9) | 6661(3) | 26.1(5) |
| C2 | 2991(5) | 5710.4(9) | 6597(3) | 25.6(5) |
| C3 | -51(5) | 5080.5(9) | 5849(3) | 32.0(6) |
| C4 | 2695(4) | 6152.1(8) | 3136(3) | 24.4(5) |
| C5 | 3889(6) | 5836.0(13) | 2447(5) | 58.8(11) |
| C6 | 2585(6) | 5606.1(13) | 971(5) | 59.5(10) |
| C7 | 114(5) | 5685.6(10) | 182(3) | 35.7(6) |
| C8 | -1096(6) | 5987.4(13) | 890(4) | 52.6(9) |
| C9 | 170(6) | 6216.7(12) | 2365(4) | 45.0(8) |
| C10 | 3754(5) | 7052.4(9) | 4894(3) | 26.7(6) |
| C11 | 5619(9) | 7331.0(18) | 4791(7) | 27.8(12) |
| C12 | 5256(10) | 7800.9(19) | 4777(7) | 33.1(13) |
| C13 | 2937(5) | 8001.5(9) | 4895(3) | 33.4(6) |
| C14 | 1235(10) | 7689.1(18) | 5096(7) | 30.4(13) |
| C15 | 1597(9) | 7211.9(18) | 5120(6) | 28.9(12) |
| C11A | 5352(9) | 7355.8(17) | 5997(7) | 28.2(12) |
| C12A | 4878(10) | 7828.1(18) | 6011(7) | 31.4(13) |
| C14A | 1152(10) | 7740.7(18) | 3655(7) | 30.4(13) |
| C15A | 1596(9) | 7270.0(18) | 3622(6) | 27.2(12) |

**Table 3 Anisotropic Displacement Parameters (Å$^2$×10$^3$) for LIGAND 2. The Anisotropic displacement factor exponent takes the form: $-2\pi^2[h^2a^{*2}U_{11}+2hka^*b^*U_{12}+\ldots]$.**

| Atom | $U_{11}$ | $U_{22}$ | $U_{33}$ | $U_{23}$ | $U_{13}$ | $U_{12}$ |
|---|---|---|---|---|---|---|
| P1 | 16.1(3) | 25.1(3) | 30.6(4) | -7.1(3) | 6.2(2) | -1.0(2) |
| O1 | 19.0(9) | 31.7(10) | 45.6(11) | -8.4(8) | 9.3(8) | -0.7(7) |
| O2 | 34.7(11) | 43.0(12) | 42.5(12) | 2.3(9) | 6.4(9) | 17.9(9) |
| N1 | 30.2(12) | 19.6(11) | 32.1(12) | -0.5(9) | 14.0(10) | 3.2(9) |
| C1 | 21.6(12) | 31.1(13) | 23.3(13) | -7.2(10) | 4.5(10) | 0.0(10) |
| C2 | 29.8(13) | 30.2(13) | 16.4(12) | -0.8(9) | 7.3(10) | 5.5(11) |
| C3 | 45.1(15) | 21.8(13) | 35.4(15) | 0.2(11) | 21.8(12) | 0.1(11) |
| C4 | 25.6(12) | 24.5(12) | 23.4(13) | -2.2(9) | 8.4(10) | -5.1(10) |
| C5 | 24.9(15) | 78(3) | 64(2) | -40.5(19) | 2.0(14) | 8.0(15) |
| C6 | 44.4(19) | 66(2) | 65(2) | -38.9(19) | 14.8(17) | 6.4(17) |
| C7 | 43.5(16) | 40.3(16) | 21.9(13) | -4.3(11) | 8.8(12) | -13.9(13) |
| C8 | 30.4(15) | 79(2) | 35.6(17) | -10.7(16) | -5.5(13) | 12.0(16) |
| C9 | 37.0(16) | 58(2) | 33.3(16) | -12.5(14) | 2.4(13) | 17.1(14) |
| C10 | 23.4(12) | 25.7(13) | 32.2(14) | -5.5(10) | 10.9(10) | -3.0(10) |
| C11 | 26(3) | 28(3) | 29(3) | 2(2) | 9(2) | 1(2) |
| C12 | 31(3) | 29(3) | 41(3) | 4(2) | 14(2) | -7(2) |
| C13 | 46.8(17) | 23.1(13) | 37.1(16) | 0.3(11) | 22.9(13) | -2.6(12) |
| C14 | 25(3) | 32(3) | 36(3) | 1(2) | 13(2) | 8(2) |
| C15 | 26(3) | 28(3) | 31(3) | 3(2) | 7(2) | -4(2) |
| C11A | 24(3) | 27(3) | 31(3) | -3(2) | 6(2) | -1(2) |
| C12A | 35(3) | 24(3) | 40(3) | -11(2) | 18(3) | -9(2) |
| C14A | 30(3) | 29(3) | 34(3) | 5(2) | 15(2) | 2(2) |
| C15A | 26(3) | 29(3) | 27(3) | -2(2) | 8(2) | -3(2) |

**Table 4 Bond Lengths for LIGAND 2.**

| Atom | Atom | Length/Å | Atom | Atom | Length/Å |
|---|---|---|---|---|---|
| P1 | O1 | 1.4912(18) | C8 | C9 | 1.383(4) |
| P1 | C1 | 1.811(3) | C10 | C11 | 1.352(6) |
| P1 | C4 | 1.804(2) | C10 | C15 | 1.376(6) |
| P1 | C10 | 1.816(3) | C10 | C11A | 1.376(6) |
| O2 | C2 | 1.224(3) | C10 | C15A | 1.473(6) |
| N1 | C2 | 1.332(3) | C11 | C12 | 1.373(7) |
| N1 | C3 | 1.455(3) | C12 | C13 | 1.467(6) |
| C1 | C2 | 1.516(4) | C13 | C14 | 1.371(6) |
| C3 | C3[1] | 1.528(5) | C13 | C12A | 1.287(6) |
| C4 | C5 | 1.375(4) | C13 | C14A | 1.408(6) |
| C4 | C9 | 1.371(4) | C14 | C15 | 1.393(7) |
| C5 | C6 | 1.389(4) | C11A | C12A | 1.392(7) |
| C6 | C7 | 1.352(4) | C14A | C15A | 1.385(7) |
| C7 | C8 | 1.364(4) | | | |

[1]-X,1-Y,1-Z

**Table 5 Bond Angles for LIGAND 2.**

| Atom | Atom | Atom | Angle/° | Atom | Atom | Atom | Angle/° |
|---|---|---|---|---|---|---|---|
| O1 | P1 | C1 | 113.87(11) | C7 | C8 | C9 | 121.2(3) |
| O1 | P1 | C4 | 111.87(11) | C4 | C9 | C8 | 120.5(3) |
| O1 | P1 | C10 | 110.91(11) | C11 | C10 | P1 | 114.7(3) |
| C1 | P1 | C10 | 103.99(12) | C11 | C10 | C15 | 123.8(4) |
| C4 | P1 | C1 | 106.24(11) | C15 | C10 | P1 | 120.9(3) |
| C4 | P1 | C10 | 109.56(12) | C11A | C10 | P1 | 119.2(3) |
| C2 | N1 | C3 | 122.1(2) | C11A | C10 | C15A | 114.9(3) |
| C2 | C1 | P1 | 111.80(17) | C15A | C10 | P1 | 125.9(3) |
| O2 | C2 | N1 | 124.1(3) | C10 | C11 | C12 | 118.2(5) |
| O2 | C2 | C1 | 121.4(2) | C11 | C12 | C13 | 121.7(4) |
| N1 | C2 | C1 | 114.5(2) | C14 | C13 | C12 | 115.4(3) |
| N1 | C3 | C3[1] | 110.5(3) | C12A | C13 | C14A | 124.2(4) |
| C5 | C4 | P1 | 118.3(2) | C13 | C14 | C15 | 123.2(4) |
| C9 | C4 | P1 | 123.6(2) | C10 | C15 | C14 | 117.5(4) |
| C9 | C4 | C5 | 118.1(2) | C10 | C11A | C12A | 123.1(5) |
| C4 | C5 | C6 | 120.6(3) | C13 | C12A | C11A | 119.7(5) |
| C7 | C6 | C5 | 120.9(3) | C15A | C14A | C13 | 116.8(5) |
| C6 | C7 | C8 | 118.6(3) | C14A | C15A | C10 | 121.1(4) |

[1]-X,1-Y,1-Z

**Table 6 Hydrogen Bonds for LIGAND 2.**

| D | H | A | d(D-H)/Å | d(H-A)/Å | d(D-A)/Å | D-H-A/° |
|---|---|---|---|---|---|---|
| N1 | H1 | O1[1] | 0.80(3) | 2.13(3) | 2.924(3) | 169(3) |

[1]-1+X,+Y,+Z

**Table 7 Hydrogen Atom Coordinates (Å×10$^4$) and Isotropic Displacement Parameters (Å$^2$×10$^3$) for LIGAND 2.**

| Atom | *x* | *y* | *z* | U(eq) |
|---|---|---|---|---|
| H1A | 4477.27 | 6322.24 | 7776.81 | 31 |
| H1B | 1668.15 | 6379.56 | 6512.67 | 31 |
| H3A | -1763.82 | 5033.57 | 5890.17 | 38 |
| H3B | 1123.04 | 4893.66 | 6754.32 | 38 |
| H5 | 5621.6 | 5774.32 | 2985.3 | 71 |
| H6 | 3439.84 | 5390.02 | 508.23 | 71 |
| H7 | -760.13 | 5534.23 | -843.61 | 43 |
| H8 | -2838.79 | 6041.13 | 360.67 | 63 |
| H9 | -717.5 | 6420.62 | 2849.07 | 54 |
| H11 | 7137.16 | 7205.05 | 4729.46 | 33 |
| H12 | 6538.22 | 8001.17 | 4689.6 | 40 |
| H13 | 2635.45 | 8325.46 | 4836.69 | 40 |
| H13A | 2692.19 | 8326.46 | 4910.22 | 40 |
| H14 | -265.05 | 7803.52 | 5224.8 | 36 |
| H15 | 398.37 | 7004.38 | 5286.98 | 35 |
| H11A | 6849.23 | 7237.34 | 6785.02 | 34 |

| | | | | |
|---|---|---|---|---|
| H12A | 5981.89 | 8021.67 | 6839.42 | 38 |
| H14A | -287.66 | 7880.84 | 2877.99 | 36 |
| H15A | 490.43 | 7084.5 | 2764.27 | 33 |
| H1 | -460(60) | 5760(10) | 5940(40) | 30(8) |

**Table 8 Atomic Occupancy for LIGAND 2.**

| **Atom** | ***Occupancy*** | **Atom** | ***Occupancy*** | **Atom** | ***Occupancy*** |
|---|---|---|---|---|---|
| C11 | 0.499(4) | H11 | 0.499(4) | C12 | 0.499(4) |
| H12 | 0.499(4) | H13 | 0.499(4) | H13A | 0.501(4) |
| C14 | 0.499(4) | H14 | 0.499(4) | C15 | 0.499(4) |
| H15 | 0.499(4) | C11A | 0.501(4) | H11A | 0.501(4) |
| C12A | 0.501(4) | H12A | 0.501(4) | C14A | 0.501(4) |
| H14A | 0.501(4) | C15A | 0.501(4) | H15A | 0.501(4) |

**Refinement model description**

Number of restraints - 0, number of constraints - unknown.

Details:

1. Fixed Uiso

At 1.2 times of:

All C(H) groups, All C(H,H) groups

2. Others

Sof(H13A)=Sof(C11A)=Sof(H11A)=Sof(C12A)=Sof(H12A)=Sof(C14A)=Sof(H14A)=
Sof(C15A)=Sof(H15A)=1-FVAR(1)

Sof(C11)=Sof(H11)=Sof(C12)=Sof(H12)=Sof(H13)=Sof(C14)=Sof(H14)=Sof(C15)=
Sof(H15)=FVAR(1)

3.a Secondary CH2 refined with riding coordinates:

C1(H1A,H1B), C3(H3A,H3B)

3.b Aromatic/amide H refined with riding coordinates:

C5(H5), C6(H6), C7(H7), C8(H8), C9(H9), C11(H11), C12(H12), C13(H13),
C13(H13A), C14(H14), C15(H15), C11A(H11A), C12A(H12A), C14A(H14A), C15A(H15A)

**B. $Sm(NO_3)_3$-2**

This figure depicts the 2:2 **$Sm(NO_3)_3$-2** complex, where only one ligand is shown for clarity. Both Sm(III) metal centers are shown, along with all of the atoms of the inner coordination sphere. The water of hydration is also shown in two locations, with hydrogen bonds depicted with dashed lines. The structure is shown with thermal ellipsoids at the 30% probability level using standard CPK colors, as well as the atom labeling scheme.

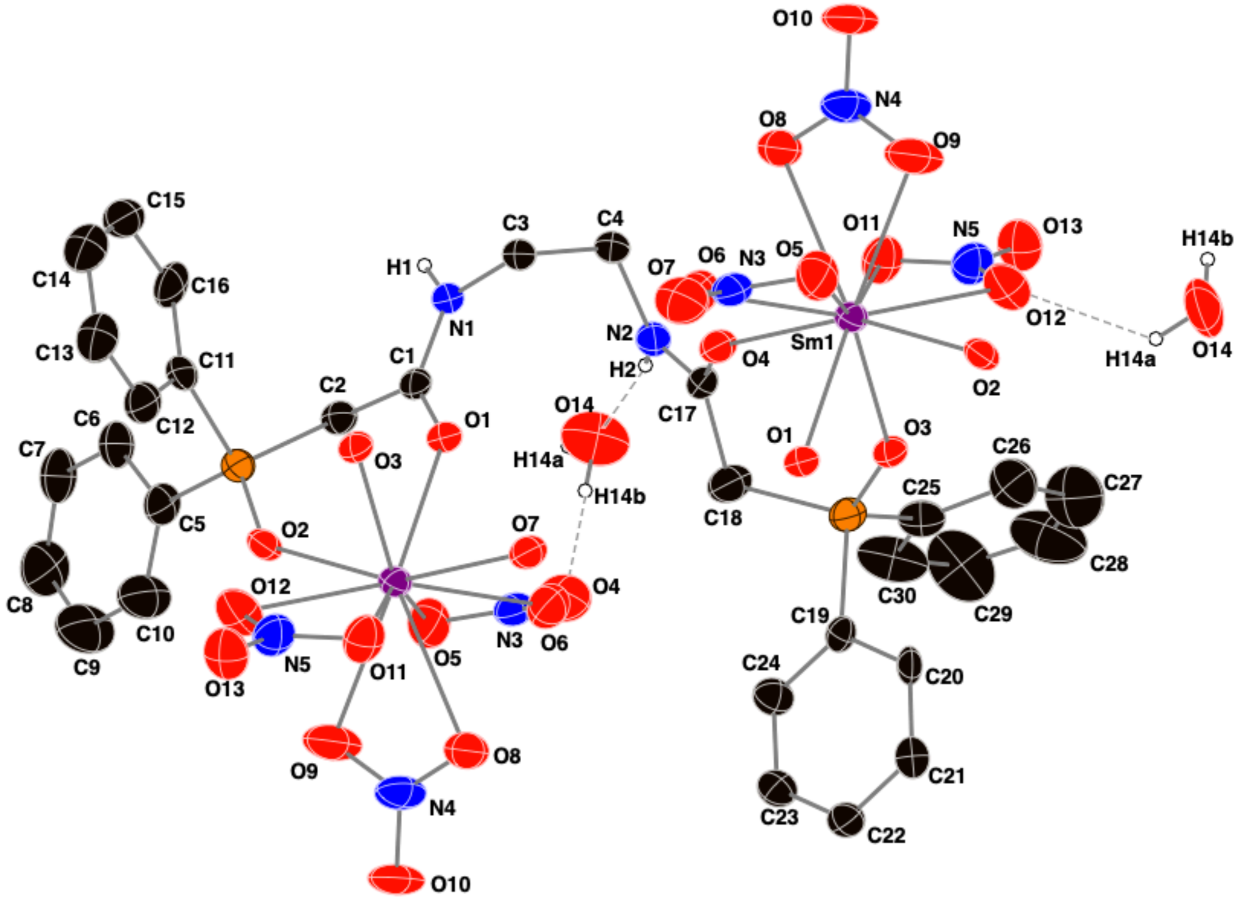


### Refinement notes

(1) SIMU and DELU commands were applied to the atoms of ring C5-C10 to ensure more uniform thermal values.

### Experimental

Single crystals of $C_{60}H_{64}N_{10}O_{28}P_4Sm_2$ (**$Sm(NO_3)_3$-2)**were grown by slow evaporation of a chloroform solution in a NMR tube. A suitable crystal was selected and mounted on a nylon loop using a small amount of paratone oil on a Bruker APEX-II CCD diffractometer. The crystal was kept at 173(2) K during data collection. Using Olex2 [1], the structure was solved with the ShelXS [2] structure solution program using Direct Methods and refined with the ShelXL [3] refinement package using Least Squares minimization.

1. Dolomanov, O.V., Bourhis, L.J., Gildea, R.J, Howard, J.A.K. & Puschmann, H. (2009), J. Appl. Cryst. 42, 339-341.
2. Sheldrick, G.M. (2015). Acta Cryst. A71, 3-8.
3. Sheldrick, G.M. (2015). Acta Cryst. C71, 3-8.

**Table 1 Crystal data and structure refinement for $Sm(NO_3)_3$-2**

| | |
|---|---|
| CCDC Code | 2003369 |
| Empirical formula | $C_{60}H_{64}N_{10}O_{28}P_4Sm_2$ |
| Formula weight | 1797.79 |
| Temperature/K | 173(2) |

| | |
|---|---|
| Crystal system | monoclinic |
| Space group | $P2_1/n$ |
| a/Å | 10.5235(15) |
| b/Å | 18.133(3) |
| c/Å | 19.488(3) |
| α/° | 90 |
| β/° | 96.944(12) |
| γ/° | 90 |
| Volume/Å$^3$ | 3691.6(9) |
| Z | 2 |
| $\rho_{calc}$g/cm$^3$ | 1.617 |
| μ/mm$^{-1}$ | 13.393 |
| F(000) | 1804.0 |
| Crystal size/mm$^3$ | 0.262 × 0.126 × 0.063 |
| Radiation | CuKα (λ = 1.54178) |
| 2Θ range for data collection/° | 6.68 to 143.44 |
| Index ranges | $-12 \le h \le 12, -22 \le k \le 22, -23 \le l \le 21$ |
| Reflections collected | 24709 |
| Independent reflections | 6996 [$R_{int}$ = 0.1460, $R_{sigma}$ = 0.1397] |
| Data/restraints/parameters | 6996/48/472 |
| Goodness-of-fit on $F^2$ | 1.020 |
| Final R indexes [I>=2σ (I)] | $R_1$ = 0.0722, $wR_2$ = 0.1524 |
| Final R indexes [all data] | $R_1$ = 0.1453, $wR_2$ = 0.1815 |
| Largest diff. peak/hole / e Å$^{-3}$ | 0.91/-0.91 |

**Table 2 Fractional Atomic Coordinates (×10$^4$) and Equivalent Isotropic Displacement Parameters (Å$^2$×10$^3$) for Sm(NO3)3-2. $U_{eq}$ is defined as 1/3 of of the trace of the orthogonalised $U_{IJ}$ tensor.**

| Atom | *x* | *y* | *z* | U(eq) |
|---|---|---|---|---|
| Sm1 | 6438.6(5) | 8266.0(3) | 5882.5(4) | 43.10(18) |
| P1 | 4050(2) | 6923.0(14) | 6186.2(16) | 43.9(7) |
| P2 | 5101(2) | 9666.9(15) | 6947.8(17) | 46.7(7) |
| O1 | 4121(6) | 8465(4) | 5437(4) | 46.8(19) |
| O2 | 5417(6) | 7189(4) | 6218(4) | 44.8(18) |
| O3 | 5402(6) | 8892(4) | 6738(4) | 45.2(18) |
| O4 | 6678(7) | 9554(4) | 5656(4) | 54(2) |
| O5 | 5827(10) | 7440(5) | 4825(6) | 91(3) |
| O6 | 6150(8) | 8565(6) | 4601(5) | 77(3) |
| O7 | 5082(9) | 7892(6) | 3823(5) | 89(3) |
| O8 | 8630(7) | 8323(5) | 5341(5) | 71(2) |
| O9 | 8168(8) | 7316(5) | 5801(7) | 112(4) |
| O10 | 9915(7) | 7371(5) | 5307(6) | 86(3) |
| O11 | 8210(8) | 8815(5) | 6743(5) | 70(3) |
| O12 | 7404(7) | 7816(5) | 7098(5) | 74(3) |
| O13 | 8851(8) | 8399(5) | 7789(5) | 77(3) |
| N1 | 1956(7) | 8464(5) | 5288(4) | 45(2) |
| N2 | 2320(8) | 9373(5) | 4036(5) | 48(2) |
| N3 | 5681(9) | 7961(7) | 4411(6) | 59(3) |
| N4 | 8923(8) | 7672(6) | 5466(7) | 71(3) |
| N5 | 8175(9) | 8342(6) | 7236(6) | 68(3) |
| C1 | 3078(9) | 8128(6) | 5386(6) | 42(3) |

| | | | | |
|---|---|---|---|---|
| C2 | 3068(9) | 7277(6) | 5430(5) | 45(3) |
| C3 | 1784(9) | 9259(6) | 5237(6) | 47(3) |
| C4 | 1346(9) | 9504(6) | 4500(6) | 51(3) |
| C5 | 3934(11) | 5933(6) | 6104(7) | 57(3) |
| C6 | 2851(12) | 5549(7) | 6176(7) | 65(3) |
| C7 | 2778(14) | 4786(7) | 6110(7) | 74(3) |
| C8 | 3842(16) | 4418(8) | 5992(9) | 97(4) |
| C9 | 4939(17) | 4770(8) | 5951(11) | 119(5) |
| C10 | 5002(14) | 5543(8) | 6014(9) | 98(4) |
| C11 | 3364(10) | 7214(6) | 6948(7) | 54(3) |
| C12 | 4294(13) | 7387(6) | 7529(8) | 68(4) |
| C13 | 3867(14) | 7602(7) | 8132(7) | 72(4) |
| C14 | 2595(14) | 7625(7) | 8198(8) | 73(4) |
| C15 | 1668(13) | 7454(7) | 7634(8) | 75(4) |
| C16 | 2099(12) | 7235(6) | 7004(7) | 65(4) |
| C17 | 6742(9) | 10143(6) | 5995(6) | 50(3) |
| C18 | 5658(12) | 10377(6) | 6404(8) | 76(4) |
| C19 | 3409(9) | 9827(6) | 6923(5) | 40(2) |
| C20 | 2642(10) | 9221(6) | 7056(5) | 46(3) |
| C21 | 1359(10) | 9318(6) | 7095(6) | 52(3) |
| C22 | 807(9) | 10010(6) | 6990(6) | 50(3) |
| C23 | 1550(10) | 10595(6) | 6837(6) | 51(3) |
| C24 | 2832(10) | 10510(6) | 6796(6) | 56(3) |
| C25 | 5727(10) | 9820(7) | 7840(7) | 56(3) |
| C26 | 6115(13) | 9195(9) | 8244(8) | 83(4) |
| C27 | 6452(18) | 9297(13) | 8953(9) | 123(8) |
| C28 | 6477(19) | 9962(15) | 9253(12) | 144(10) |
| C29 | 6190(20) | 10568(12) | 8876(12) | 131(9) |
| C30 | 5763(14) | 10511(10) | 8132(10) | 110(7) |
| O14 | 2474(11) | 7938(6) | 3419(8) | 109(4) |

**Table 3 Anisotropic Displacement Parameters (Å$^2$×10$^3$) for Sm(NO3)3-2. The Anisotropic displacement factor exponent takes the form: $-2\pi^2[h^2a^{*2}U_{11}+2hka^*b^*U_{12}+\ldots]$.**

| Atom | $U_{11}$ | $U_{22}$ | $U_{33}$ | $U_{23}$ | $U_{13}$ | $U_{12}$ |
|---|---|---|---|---|---|---|
| Sm1 | 38.1(3) | 36.0(3) | 57.0(4) | 4.8(4) | 13.2(2) | 3.8(3) |
| P1 | 44.6(13) | 35.9(15) | 52.8(19) | 5.1(13) | 12.9(12) | 2.3(10) |
| P2 | 45.6(14) | 40.2(15) | 57(2) | -1.2(14) | 16.8(13) | -2.4(11) |
| O1 | 46(4) | 45(4) | 53(5) | 3(4) | 18(3) | 5(3) |
| O2 | 38(3) | 37(4) | 59(5) | 5(4) | 3(3) | 8(3) |
| O3 | 52(4) | 43(4) | 43(5) | -2(4) | 19(3) | 4(3) |
| O4 | 56(4) | 39(4) | 73(6) | 4(4) | 30(4) | 1(3) |
| O5 | 130(9) | 55(6) | 94(9) | 9(6) | 43(7) | 19(6) |
| O6 | 72(6) | 90(7) | 67(7) | 19(6) | 7(5) | -12(5) |
| O7 | 90(7) | 112(9) | 63(7) | -20(6) | 6(6) | 14(6) |
| O8 | 54(5) | 65(6) | 96(7) | 7(6) | 21(4) | 7(4) |
| O9 | 55(5) | 69(7) | 222(14) | 21(8) | 55(7) | 7(5) |
| O10 | 43(5) | 62(6) | 159(10) | -4(6) | 38(5) | 7(4) |
| O11 | 68(5) | 69(6) | 72(7) | 6(5) | 7(5) | -22(4) |
| O12 | 44(4) | 66(6) | 106(8) | 9(5) | -19(4) | -10(4) |

| | | | | | | |
|---|---|---|---|---|---|---|
| O13 | 68(5) | 89(8) | 69(7) | 9(5) | -17(5) | -5(5) |
| N1 | 39(4) | 53(6) | 44(6) | 10(4) | 13(4) | 4(4) |
| N2 | 57(5) | 44(5) | 43(6) | 0(4) | 13(4) | 10(4) |
| N3 | 51(6) | 78(8) | 51(8) | 2(6) | 18(5) | 11(5) |
| N4 | 37(5) | 64(7) | 113(11) | -6(7) | 18(6) | -2(5) |
| N5 | 52(6) | 81(8) | 70(8) | 10(7) | 6(5) | -11(6) |
| C1 | 43(5) | 45(6) | 43(7) | 4(5) | 22(5) | 6(4) |
| C2 | 41(5) | 56(7) | 37(7) | -3(5) | 4(4) | 2(5) |
| C3 | 38(5) | 48(6) | 56(8) | 10(6) | 14(5) | 4(4) |
| C4 | 35(5) | 59(7) | 58(8) | 13(6) | 8(5) | 6(5) |
| C5 | 72(6) | 31(5) | 72(8) | 4(6) | 21(6) | -3(4) |
| C6 | 76(6) | 48(6) | 72(8) | 13(6) | 11(6) | -10(5) |
| C7 | 95(7) | 52(6) | 75(9) | 12(7) | 7(7) | -24(6) |
| C8 | 128(10) | 42(6) | 126(11) | -2(8) | 34(10) | -11(6) |
| C9 | 114(9) | 51(7) | 199(13) | 1(10) | 51(10) | 14(6) |
| C10 | 87(7) | 48(6) | 165(12) | 3(8) | 45(9) | 0(6) |
| C11 | 52(6) | 36(6) | 77(10) | 22(6) | 18(6) | 1(5) |
| C12 | 80(9) | 43(7) | 85(11) | 15(7) | 33(8) | 10(6) |
| C13 | 97(11) | 61(9) | 61(10) | -5(7) | 17(8) | -8(7) |
| C14 | 99(11) | 59(9) | 65(11) | -13(8) | 25(9) | -10(7) |
| C15 | 83(9) | 43(7) | 106(13) | 12(8) | 46(9) | 5(6) |
| C16 | 83(9) | 42(7) | 73(10) | 16(7) | 29(8) | -5(6) |
| C17 | 53(7) | 47(7) | 55(8) | 14(6) | 25(6) | 9(5) |
| C18 | 82(9) | 40(7) | 115(13) | 5(8) | 55(9) | 2(6) |
| C19 | 46(5) | 47(6) | 28(6) | 2(5) | 4(4) | -3(5) |
| C20 | 63(7) | 45(6) | 32(7) | 15(5) | 11(5) | 11(5) |
| C21 | 60(7) | 48(7) | 47(8) | 5(6) | 3(6) | -3(5) |
| C22 | 36(5) | 71(8) | 41(7) | 4(6) | -3(5) | 4(5) |
| C23 | 52(6) | 46(7) | 56(8) | -11(6) | 9(5) | 8(5) |
| C24 | 54(7) | 49(7) | 63(9) | -4(6) | 7(6) | 2(5) |
| C25 | 44(6) | 61(8) | 62(9) | -19(7) | 0(5) | -10(5) |
| C26 | 82(10) | 91(12) | 73(12) | -2(9) | -5(8) | -3(8) |
| C27 | 130(16) | 170(20) | 58(13) | -13(13) | -28(11) | 17(15) |
| C28 | 109(15) | 190(30) | 120(20) | -70(20) | -25(13) | 31(17) |
| C29 | 145(18) | 100(16) | 130(20) | -62(15) | -45(15) | 12(14) |
| C30 | 79(10) | 104(14) | 141(18) | -54(13) | -9(10) | 14(9) |
| O14 | 108(8) | 80(8) | 136(11) | -57(8) | 0(8) | -6(6) |

**Table 4 Bond Lengths for Sm(NO3)3-2.**

| Atom | Atom | Length/Å | Atom | Atom | Length/Å |
|---|---|---|---|---|---|
| Sm1 | O1 | 2.514(7) | N1 | C3 | 1.456(12) |
| Sm1 | O2 | 2.360(7) | N2 | C4 | 1.466(13) |
| Sm1 | O3 | 2.387(7) | N2 | C17[1] | 1.329(13) |
| Sm1 | O4 | 2.396(7) | C1 | C2 | 1.545(13) |
| Sm1 | O5 | 2.567(11) | C3 | C4 | 1.521(14) |
| Sm1 | O6 | 2.537(9) | C5 | C6 | 1.356(15) |
| Sm1 | O8 | 2.651(8) | C5 | C10 | 1.357(17) |
| Sm1 | O9 | 2.525(9) | C6 | C7 | 1.391(16) |
| Sm1 | O11 | 2.554(8) | C7 | C8 | 1.347(19) |

| | | | | | |
|---|---|---|---|---|---|
| Sm1 | O12 | 2.594(9) | C8 | C9 | 1.33(2) |
| Sm1 | N3 | 2.935(12) | C9 | C10 | 1.407(18) |
| P1 | O2 | 1.511(7) | C11 | C12 | 1.438(17) |
| P1 | C2 | 1.813(10) | C11 | C16 | 1.349(15) |
| P1 | C5 | 1.806(11) | C12 | C13 | 1.365(17) |
| P1 | C11 | 1.807(13) | C13 | C14 | 1.361(17) |
| P2 | O3 | 1.508(7) | C14 | C15 | 1.413(18) |
| P2 | C18 | 1.809(12) | C15 | C16 | 1.415(17) |
| P2 | C19 | 1.800(10) | C17 | C18 | 1.528(14) |
| P2 | C25 | 1.805(13) | C19 | C20 | 1.406(14) |
| O1 | C1 | 1.251(11) | C19 | C24 | 1.388(14) |
| O4 | C17 | 1.253(13) | C20 | C21 | 1.373(14) |
| O5 | N3 | 1.239(13) | C21 | C22 | 1.389(14) |
| O6 | N3 | 1.240(13) | C22 | C23 | 1.372(15) |
| O7 | N3 | 1.246(13) | C23 | C24 | 1.370(14) |
| O8 | N4 | 1.236(12) | C25 | C26 | 1.411(17) |
| O9 | N4 | 1.265(13) | C25 | C30 | 1.374(18) |
| O10 | N4 | 1.249(11) | C26 | C27 | 1.40(2) |
| O11 | N5 | 1.292(12) | C27 | C28 | 1.34(2) |
| O12 | N5 | 1.260(12) | C28 | C29 | 1.34(3) |
| O13 | N5 | 1.223(12) | C29 | C30 | 1.47(2) |
| N1 | C1 | 1.322(12) | | | |

[1]1-X,2-Y,1-Z

**Table 5 Bond Angles for Sm(NO3)3-2.**

| Atom | Atom | Atom | Angle/° | Atom | Atom | Atom | Angle/° |
|---|---|---|---|---|---|---|---|
| O1 | Sm1 | O5 | 70.6(3) | C1 | O1 | Sm1 | 139.7(7) |
| O1 | Sm1 | O6 | 68.5(3) | P1 | O2 | Sm1 | 135.7(4) |
| O1 | Sm1 | O8 | 135.3(3) | P2 | O3 | Sm1 | 139.6(4) |
| O1 | Sm1 | O9 | 138.8(3) | C17 | O4 | Sm1 | 137.1(8) |
| O1 | Sm1 | O11 | 140.5(3) | N3 | O5 | Sm1 | 94.4(8) |
| O1 | Sm1 | O12 | 128.1(2) | N3 | O6 | Sm1 | 95.8(8) |
| O1 | Sm1 | N3 | 63.4(2) | N4 | O8 | Sm1 | 95.3(6) |
| O2 | Sm1 | O1 | 76.1(2) | N4 | O9 | Sm1 | 100.8(7) |
| O2 | Sm1 | O3 | 86.4(2) | N5 | O11 | Sm1 | 98.3(6) |
| O2 | Sm1 | O4 | 156.8(2) | N5 | O12 | Sm1 | 97.3(7) |
| O2 | Sm1 | O5 | 70.2(3) | C1 | N1 | C3 | 124.6(9) |
| O2 | Sm1 | O6 | 116.4(3) | C17[1] | N2 | C4 | 120.0(9) |
| O2 | Sm1 | O8 | 126.1(2) | O5 | N3 | Sm1 | 60.7(7) |
| O2 | Sm1 | O9 | 79.0(3) | O5 | N3 | O6 | 117.7(12) |
| O2 | Sm1 | O11 | 117.5(3) | O5 | N3 | O7 | 122.1(14) |
| O2 | Sm1 | O12 | 68.1(2) | O6 | N3 | Sm1 | 59.3(7) |
| O2 | Sm1 | N3 | 92.0(3) | O6 | N3 | O7 | 120.1(12) |
| O3 | Sm1 | O1 | 70.5(2) | O7 | N3 | Sm1 | 164.9(8) |
| O3 | Sm1 | O4 | 74.5(2) | O8 | N4 | O9 | 115.5(9) |
| O3 | Sm1 | O5 | 138.3(3) | O8 | N4 | O10 | 124.3(10) |
| O3 | Sm1 | O6 | 125.1(3) | O10 | N4 | O9 | 120.1(11) |
| O3 | Sm1 | O8 | 138.8(3) | O12 | N5 | O11 | 114.8(10) |
| O3 | Sm1 | O9 | 139.7(3) | O13 | N5 | O11 | 122.3(11) |
| O3 | Sm1 | O11 | 73.5(3) | O13 | N5 | O12 | 122.9(12) |

| | | | | | | | |
|---|---|---|---|---|---|---|---|
| O3 | Sm1 | O12 | 71.0(3) | O1 | C1 | N1 | 123.1(10) |
| O3 | Sm1 | N3 | 132.8(3) | O1 | C1 | C2 | 119.7(8) |
| O4 | Sm1 | O1 | 85.1(2) | N1 | C1 | C2 | 117.2(9) |
| O4 | Sm1 | O5 | 116.3(3) | C1 | C2 | P1 | 113.0(7) |
| O4 | Sm1 | O6 | 67.2(3) | N1 | C3 | C4 | 112.0(10) |
| O4 | Sm1 | O8 | 76.9(3) | N2 | C4 | C3 | 112.4(8) |
| O4 | Sm1 | O9 | 124.1(3) | C6 | C5 | P1 | 123.4(10) |
| O4 | Sm1 | O11 | 69.9(3) | C6 | C5 | C10 | 117.7(12) |
| O4 | Sm1 | O12 | 115.9(3) | C10 | C5 | P1 | 118.7(10) |
| O4 | Sm1 | N3 | 91.5(3) | C5 | C6 | C7 | 122.6(13) |
| O5 | Sm1 | O8 | 81.3(3) | C8 | C7 | C6 | 118.1(13) |
| O5 | Sm1 | O12 | 125.6(3) | C9 | C8 | C7 | 121.2(14) |
| O5 | Sm1 | N3 | 24.9(3) | C8 | C9 | C10 | 120.3(15) |
| O6 | Sm1 | O5 | 49.1(3) | C5 | C10 | C9 | 119.9(14) |
| O6 | Sm1 | O8 | 66.8(3) | C12 | C11 | P1 | 114.1(9) |
| O6 | Sm1 | O11 | 123.6(3) | C16 | C11 | P1 | 124.8(11) |
| O6 | Sm1 | O12 | 162.4(3) | C16 | C11 | C12 | 120.9(13) |
| O6 | Sm1 | N3 | 24.9(3) | C13 | C12 | C11 | 118.4(13) |
| O8 | Sm1 | N3 | 76.4(3) | C14 | C13 | C12 | 121.3(14) |
| O9 | Sm1 | O5 | 70.4(4) | C13 | C14 | C15 | 120.9(14) |
| O9 | Sm1 | O6 | 94.9(4) | C14 | C15 | C16 | 118.2(13) |
| O9 | Sm1 | O8 | 48.2(3) | C11 | C16 | C15 | 120.1(14) |
| O9 | Sm1 | O11 | 80.4(3) | O4 | C17 | N2[1] | 122.1(10) |
| O9 | Sm1 | O12 | 68.7(4) | O4 | C17 | C18 | 121.1(10) |
| O9 | Sm1 | N3 | 85.6(4) | N2[1] | C17 | C18 | 116.5(11) |
| O11 | Sm1 | O5 | 147.9(3) | C17 | C18 | P2 | 115.0(8) |
| O11 | Sm1 | O8 | 69.2(3) | C20 | C19 | P2 | 117.2(8) |
| O11 | Sm1 | O12 | 49.4(3) | C24 | C19 | P2 | 124.0(8) |
| O11 | Sm1 | N3 | 143.7(3) | C24 | C19 | C20 | 118.8(10) |
| O12 | Sm1 | O8 | 96.4(3) | C21 | C20 | C19 | 120.0(10) |
| O12 | Sm1 | N3 | 149.7(3) | C20 | C21 | C22 | 120.3(11) |
| O2 | P1 | C2 | 111.5(5) | C23 | C22 | C21 | 119.4(10) |
| O2 | P1 | C5 | 112.0(5) | C24 | C23 | C22 | 121.1(11) |
| O2 | P1 | C11 | 110.2(5) | C23 | C24 | C19 | 120.2(11) |
| C5 | P1 | C2 | 104.7(5) | C26 | C25 | P2 | 117.4(10) |
| C5 | P1 | C11 | 109.5(5) | C30 | C25 | P2 | 121.8(13) |
| C11 | P1 | C2 | 108.6(5) | C30 | C25 | C26 | 120.6(15) |
| O3 | P2 | C18 | 114.3(5) | C27 | C26 | C25 | 117.8(17) |
| O3 | P2 | C19 | 112.5(5) | C28 | C27 | C26 | 123(2) |
| O3 | P2 | C25 | 109.9(5) | C29 | C28 | C27 | 121(2) |
| C19 | P2 | C18 | 105.0(5) | C28 | C29 | C30 | 120.5(19) |
| C19 | P2 | C25 | 104.2(5) | C25 | C30 | C29 | 117.6(18) |
| C25 | P2 | C18 | 110.4(7) | | | | |

[1]1-X,2-Y,1-Z

**Table 6 Hydrogen Bonds for Sm(NO3)3-2.**

| D | H | A | d(D-H)/Å | d(H-A)/Å | d(D-A)/Å | D-H-A/° |
|---|---|---|---|---|---|---|
| N1 | H1 | O10[1] | 0.88 | 2.06 | 2.926(12) | 166.6 |
| N2 | H2 | O14 | 0.88 | 2.02 | 2.878(13) | 164.3 |
| O14 | H14B | O7 | 0.87 | 1.90 | 2.764(15) | 175.0 |

[1]-1+X,+Y,+Z

**Table 7 Torsion Angles for Sm(NO3)3-2.**

| A | B | C | D | Angle/° | A | B | C | D | Angle/° |
|---|---|---|---|---|---|---|---|---|---|
| Sm1 | O1 | C1 | N1 | 160.8(7) | C5 | P1 | O2 | Sm1 | 149.6(6) |
| Sm1 | O1 | C1 | C2 | -19.9(16) | C5 | P1 | C2 | C1 | -177.9(7) |
| Sm1 | O4 | C17 | N2[1] | 128.5(10) | C5 | P1 | C11 | C12 | 102.2(9) |
| Sm1 | O4 | C17 | C18 | -58.4(16) | C5 | P1 | C11 | C16 | -74.0(11) |
| Sm1 | O5 | N3 | O6 | 17.0(11) | C5 | C6 | C7 | C8 | 2(2) |
| Sm1 | O5 | N3 | O7 | -162.8(10) | C6 | C5 | C10 | C9 | 4(3) |
| Sm1 | O6 | N3 | O5 | -17.3(11) | C6 | C7 | C8 | C9 | 1(3) |
| Sm1 | O6 | N3 | O7 | 162.5(9) | C7 | C8 | C9 | C10 | -1(3) |
| Sm1 | O8 | N4 | O9 | -3.9(13) | C8 | C9 | C10 | C5 | -1(3) |
| Sm1 | O8 | N4 | O10 | 179.0(12) | C10 | C5 | C6 | C7 | -5(2) |
| Sm1 | O9 | N4 | O8 | 4.2(14) | C11 | P1 | O2 | Sm1 | -88.2(7) |
| Sm1 | O9 | N4 | O10 | -178.6(10) | C11 | P1 | C2 | C1 | 65.2(8) |
| Sm1 | O11 | N5 | O12 | 3.8(11) | C11 | P1 | C5 | C6 | 46.5(14) |
| Sm1 | O11 | N5 | O13 | -177.1(11) | C11 | P1 | C5 | C10 | -128.8(13) |
| Sm1 | O12 | N5 | O11 | -3.7(11) | C11 | C12 | C13 | C14 | 2.7(19) |
| Sm1 | O12 | N5 | O13 | 177.2(11) | C12 | C11 | C16 | C15 | 2.2(17) |
| P1 | C5 | C6 | C7 | 179.9(11) | C12 | C13 | C14 | C15 | -2(2) |
| P1 | C5 | C10 | C9 | 179.8(14) | C13 | C14 | C15 | C16 | 2(2) |
| P1 | C11 | C12 | C13 | -179.0(9) | C14 | C15 | C16 | C11 | -1.8(18) |
| P1 | C11 | C16 | C15 | 178.2(9) | C16 | C11 | C12 | C13 | -2.6(17) |
| P2 | C19 | C20 | C21 | 175.2(9) | C17[1] | N2 | C4 | C3 | -83.1(13) |
| P2 | C19 | C24 | C23 | -175.1(9) | C18 | P2 | O3 | Sm1 | 0.6(9) |
| P2 | C25 | C26 | C27 | -172.3(13) | C18 | P2 | C19 | C20 | 155.1(9) |
| P2 | C25 | C30 | C29 | 174.7(13) | C18 | P2 | C19 | C24 | -26.3(12) |
| O1 | C1 | C2 | P1 | 51.9(12) | C18 | P2 | C25 | C26 | -140.8(10) |
| O2 | P1 | C2 | C1 | -56.5(8) | C18 | P2 | C25 | C30 | 42.7(13) |
| O2 | P1 | C5 | C6 | 169.1(11) | C19 | P2 | O3 | Sm1 | 120.2(6) |
| O2 | P1 | C5 | C10 | -6.2(15) | C19 | P2 | C18 | C17 | -145.3(10) |
| O2 | P1 | C11 | C12 | -21.5(9) | C19 | P2 | C25 | C26 | 106.9(10) |
| O2 | P1 | C11 | C16 | 162.3(9) | C19 | P2 | C25 | C30 | -69.6(12) |
| O3 | P2 | C18 | C17 | -21.6(13) | C19 | C20 | C21 | C22 | 1.3(17) |
| O3 | P2 | C19 | C20 | 30.2(10) | C20 | C19 | C24 | C23 | 3.5(18) |
| O3 | P2 | C19 | C24 | -151.1(9) | C20 | C21 | C22 | C23 | 0.9(18) |
| O3 | P2 | C25 | C26 | -13.9(11) | C21 | C22 | C23 | C24 | -1.0(19) |
| O3 | P2 | C25 | C30 | 169.7(11) | C22 | C23 | C24 | C19 | -1.3(19) |
| O4 | C17 | C18 | P2 | 48.4(16) | C24 | C19 | C20 | C21 | -3.5(17) |
| N1 | C1 | C2 | P1 | -128.9(9) | C25 | P2 | O3 | Sm1 | -124.1(7) |
| N1 | C3 | C4 | N2 | -65.8(11) | C25 | P2 | C18 | C17 | 102.9(11) |
| N2[1] | C17 | C18 | P2 | -138.1(10) | C25 | P2 | C19 | C20 | -88.8(9) |
| C1 | N1 | C3 | C4 | 106.9(11) | C25 | P2 | C19 | C24 | 89.8(11) |
| C2 | P1 | O2 | Sm1 | 32.6(8) | C25 | C26 | C27 | C28 | -3(3) |
| C2 | P1 | C5 | C6 | -69.9(13) | C26 | C25 | C30 | C29 | -2(2) |
| C2 | P1 | C5 | C10 | 114.9(13) | C26 | C27 | C28 | C29 | -1(4) |
| C2 | P1 | C11 | C12 | -144.0(8) | C27 | C28 | C29 | C30 | 3(4) |
| C2 | P1 | C11 | C16 | 39.8(11) | C28 | C29 | C30 | C25 | -2(3) |
| C3 | N1 | C1 | O1 | -1.0(17) | C30 | C25 | C26 | C27 | 4(2) |
| C3 | N1 | C1 | C2 | 179.8(10) | | | | | |

[1]1-X,2-Y,1-Z

**Table 8 Hydrogen Atom Coordinates (Å×10[4]) and Isotropic Displacement Parameters (Å[2]×10[3]) for Sm(NO3)3-2.**

| **Atom** | ***x*** | ***y*** | ***z*** | **U(eq)** |
|---|---|---|---|---|
| H1 | 1264 | 8186.86 | 5251.34 | 54 |
| H2 | 2282.25 | 8973.16 | 3777.93 | 57 |
| H2A | 3380.18 | 7071.55 | 5010.08 | 53 |
| H2B | 2176.3 | 7106.36 | 5437.63 | 53 |
| H3A | 2603.61 | 9505.44 | 5403.13 | 56 |
| H3B | 1142.22 | 9414.25 | 5540.01 | 56 |
| H4A | 555.65 | 9233.84 | 4325.06 | 61 |
| H4B | 1139.49 | 10036.5 | 4499.67 | 61 |
| H6 | 2113.7 | 5811.83 | 6274.41 | 78 |
| H7 | 1999.58 | 4531.38 | 6147.34 | 89 |
| H8 | 3808.87 | 3897.14 | 5938.16 | 116 |
| H9 | 5683.62 | 4499.2 | 5878.03 | 142 |
| H10 | 5790.81 | 5791.4 | 5993.81 | 117 |
| H12 | 5184.06 | 7352.12 | 7493.49 | 81 |
| H13 | 4469.85 | 7737.99 | 8513.7 | 87 |
| H14 | 2326.1 | 7759.19 | 8629.23 | 88 |
| H15 | 779.67 | 7484.05 | 7676.5 | 89 |
| H16 | 1497.45 | 7102.73 | 6621.1 | 77 |
| H18A | 4926.09 | 10544.67 | 6074.31 | 91 |
| H18B | 5953.27 | 10803.07 | 6698.61 | 91 |
| H20 | 3012.09 | 8743.72 | 7117.93 | 55 |
| H21 | 846.58 | 8909 | 7194.27 | 62 |
| H22 | -78.69 | 10078.62 | 7024.58 | 60 |
| H23 | 1169.33 | 11067.54 | 6758.82 | 61 |
| H24 | 3327.45 | 10919.33 | 6680.55 | 67 |
| H26 | 6145.92 | 8720.74 | 8039.69 | 100 |
| H27 | 6672.47 | 8877.47 | 9233.66 | 148 |
| H28 | 6703.51 | 10003.82 | 9738.64 | 173 |
| H29 | 6251 | 11038.88 | 9090.85 | 157 |
| H30 | 5524.58 | 10936.03 | 7861.43 | 132 |
| H14A | 2225.14 | 7493.67 | 3301.68 | 164 |
| H14B | 3297.1 | 7899.97 | 3530.61 | 164 |

**Crystal structure determination of [Sm(NO3)3-2]**

**Crystal Data** for $C_{60}H_{64}N_{10}O_{28}P_4Sm_2$ ($M$ =1797.79 g/mol): monoclinic, space group $P2_1/n$ (no. 14), $a$ = 10.5235(15) Å, $b$ = 18.133(3) Å, $c$ = 19.488(3) Å, $\beta$ = 96.944(12)°, $V$ = 3691.6(9) Å$^3$, $Z$ = 2, $T$ = 173(2) K, μ(CuKα) = 13.393 mm$^{-1}$, $Dcalc$ = 1.617 g/cm$^3$, 24709 reflections measured (6.68° ≤ 2Θ ≤ 143.44°), 6996 unique ($R_{int}$ = 0.1460, $R_{sigma}$ = 0.1397) which were used in all calculations. The final $R_1$ was 0.0722 (I > 2σ(I)) and $wR_2$ was 0.1815 (all data).

**Refinement model description**

Number of restraints - 48, number of constraints - unknown.

Details:

1. Fixed Uiso

At 1.2 times of:

All C(H) groups, All C(H,H) groups, All N(H) groups

At 1.5 times of:

All O(H,H) groups

2. Rigid bond restraints

C5, C10, C9, C8, C7, C6
with sigma for 1-2 distances of 0.01 and sigma for 1-3 distances of 0.01
3. Uiso/Uaniso restraints and constraints
C5 ≈ C10 ≈ C9 ≈ C8 ≈ C7 ≈ C6: within 2A with sigma of 0.02
and sigma for terminal atoms of 0.04
4.a Free rotating group:
O14(H14A,H14B)
4.b Secondary CH2 refined with riding coordinates:
C2(H2A,H2B), C3(H3A,H3B), C4(H4A,H4B), C18(H18A,H18B)
4.c Aromatic/amide H refined with riding coordinates:
N1(H1), N2(H2), C6(H6), C7(H7), C8(H8), C9(H9), C10(H10), C12(H12), C13(H13), C14(H14), C15(H15), C16(H16), C20(H20), C21(H21), C22(H22), C23(H23), C24(H24), C26(H26), C27(H27), C28(H28), C29(H29), C30(H30)

**C. $Tb(NO_3)_3$-2 (MeOH solvate)**

The figure on the left depicts the atom numbering scheme of the 2:2 **$Tb(NO_3)_3$-2** complex using a ball and stick model with standard CPK colors (Tb atoms are colored green). Only one ligand of the major component is shown for clarity, although all atoms of the inner coordination sphere of each metal center are shown. Hydrogen atoms bonded to carbon atoms are not shown for clarity, and hydrogen bonds are depicted with blue, dashed lines.
The figure on the right depicts the thermal ellipsoids, which are shown at the 40% probability level.

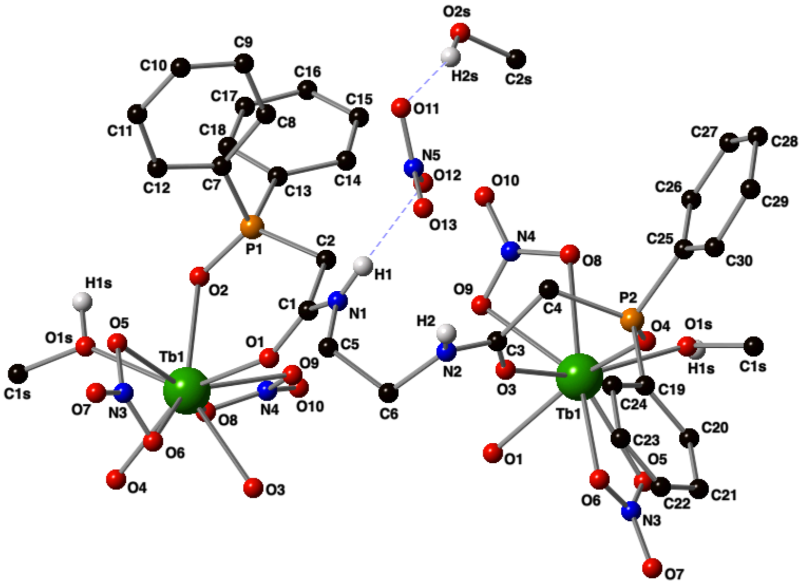


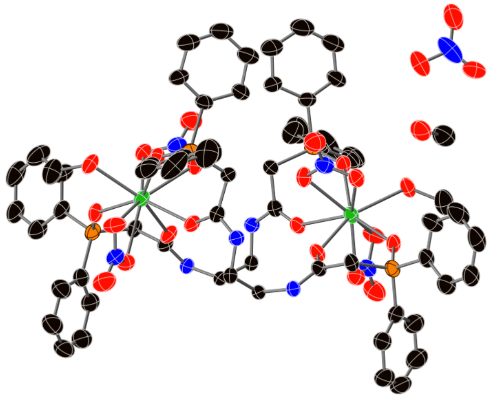

**Figure depicting atoms of inner-coordination sphere:**

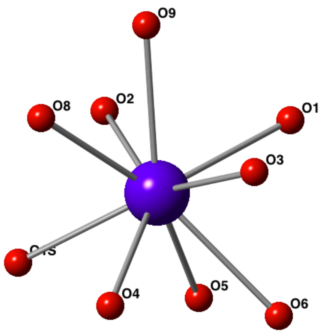


**Refinement notes**

(1) The electron density corresponding to the atoms of ring C13-C18, along with the ipso P1 atom, was positionally disordered. This electron density was modeled over two positions, and the occupancy of each component was found to be 0.733(17) and 0.267(17) by refining against a free variable. The FLAT command was applied to this ring to ensure a planar benzene ring, and EADP, SIMU and DELU commands were applied to the atoms of the rings to ensure more uniform thermal values.

(2) SIMU and DELU commands were applied to the atoms of ring C19-C24 to ensure more uniform thermal values.

**Experimental**

Single crystals of $C_{64}H_{76}N_{10}O_{30}P_4Tb_2$ ]**$Tb(NO_3)_3$-2 (MeOH solvate)]** were grown by slow diffusion of diethyl ether into a solution of the complex in methanol. A suitable crystal was selected and mounted on a nylon loop using a small amount of paratone oil on a Bruker APEX-II CCD diffractometer. The crystal was kept at 173(2) K during data collection. Using Olex2

[1], the structure was solved with the ShelXT [2] structure solution program using Intrinsic Phasing and refined with the ShelXL [3] refinement package using Least Squares minimization.


1. Dolomanov, O.V., Bourhis, L.J., Gildea, R.J, Howard, J.A.K. & Puschmann, H. (2009), J. Appl. Cryst. 42, 339-341.
2. Sheldrick, G.M. (2015). Acta Cryst. A71, 3-8.
3. Sheldrick, G.M. (2015). Acta Cryst. C71, 3-8.


**$Tb(NO_3)_3$-2 (MeOH solvate)**

**Table 1 Crystal data and structure refinement for $Tb(NO_3)_3$-2 (MeOH solvate).**

| | |
|---|---|
| CCDC number | 2003371 |
| Empirical formula | $C_{64}H_{76}N_{10}O_{30}P_4Tb_2$ |
| Formula weight | 1907.06 |
| Temperature/K | 173(2) |
| Crystal system | tetragonal |
| Space group | P-4$2_1$c |
| a/Å | 18.2898(4) |
| b/Å | 18.2898(4) |
| c/Å | 27.4073(6) |
| α/° | 90 |
| β/° | 90 |
| γ/° | 90 |
| Volume/Å$^3$ | 9168.2(4) |
| Z | 4 |
| $\rho_{calc}$g/cm$^3$ | 1.382 |
| μ/mm$^{-1}$ | 8.794 |
| F(000) | 3840.0 |
| Crystal size/mm$^3$ | 0.284 × 0.2 × 0.183 |
| Radiation | CuKα (λ = 1.54178) |
| 2Θ range for data collection/° | 5.81 to 136.676 |
| Index ranges | -22 ≤ h ≤ 22, -21 ≤ k ≤ 22, -33 ≤ l ≤ 33 |
| Reflections collected | 111590 |
| Independent reflections | 8408 [$R_{int}$ = 0.1335, $R_{sigma}$ = 0.0634] |
| Data/restraints/parameters | 8408/165/536 |
| Goodness-of-fit on F$^2$ | 1.094 |
| Final R indexes [I>=2σ (I)] | $R_1$ = 0.0638, w$R_2$ = 0.1895 |
| Final R indexes [all data] | $R_1$ = 0.0827, w$R_2$ = 0.2052 |
| Largest diff. peak/hole / e Å$^{-3}$ | 1.10/-0.77 |
| Flack parameter | 0.023(4) |

**Table 2 Fractional Atomic Coordinates (×10$^4$) and Equivalent Isotropic Displacement Parameters (Å$^2$×10$^3$) for TB(NO3)3-2 (MEOH SOLVATE). $U_{eq}$ is defined as 1/3 of of the trace of the 17rthogonalized $U_{IJ}$ tensor.**

| Atom | *x* | *y* | *z* | U(eq) |
|---|---|---|---|---|
| Tb1 | 3528.6(4) | 6587.3(4) | 6374.4(3) | 48.5(2) |
| P1 | 5005(13) | 6906(12) | 7168(4) | 45(2) |
| P1A | 4990(40) | 6890(40) | 7270(15) | 45(2) |

| | | | | |
|---|---|---|---|---|
| P2 | 8132.5(17) | 3889(2) | 5773.8(13) | 50.5(8) |
| O1 | 4745(4) | 6346(5) | 6130(3) | 51(2) |
| O2 | 4239(5) | 7023(5) | 7007(4) | 54(2) |
| O3 | 6562(5) | 4459(5) | 5867(3) | 54(2) |
| O4 | 7666(4) | 3387(5) | 6077(3) | 54(2) |
| O5 | 3922(7) | 7805(6) | 6051(4) | 76(3) |
| O6 | 3637(5) | 7033(6) | 5507(3) | 58(2) |
| O7 | 4024(8) | 8089(7) | 5277(5) | 89(4) |
| O8 | 2819(5) | 5841(6) | 6946(4) | 61(3) |
| O9 | 3937(5) | 5490(5) | 6869(4) | 58(2) |
| O10 | 3254(7) | 4946(8) | 7411(4) | 82(3) |
| N1 | 5978(6) | 6344(6) | 6033(4) | 46(2) |
| N2 | 6847(5) | 5437(5) | 5400(4) | 43(2) |
| N3 | 3866(7) | 7637(8) | 5605(5) | 66(3) |
| N4 | 3341(7) | 5413(7) | 7083(4) | 62(3) |
| C1 | 5367(7) | 6313(6) | 6312(5) | 46(3) |
| C2 | 5487(7) | 6210(8) | 6866(4) | 50(3) |
| C3 | 7019(6) | 4897(7) | 5690(4) | 45(3) |
| C4 | 7820(6) | 4818(7) | 5837(5) | 48(3) |
| C5 | 5939(7) | 6382(7) | 5513(4) | 44(3) |
| C6 | 6088(6) | 5635(7) | 5290(4) | 41(2) |
| C7 | 5523(9) | 7710(8) | 7133(5) | 60(4) |
| C8 | 6275(9) | 7700(10) | 7271(7) | 82(5) |
| C9 | 6700(14) | 8331(13) | 7268(9) | 114(8) |
| C10 | 6393(13) | 8950(13) | 7087(10) | 116(10) |
| C11 | 5688(15) | 8986(10) | 6973(7) | 96(7) |
| C12 | 5213(10) | 8346(8) | 6982(6) | 67(4) |
| C13 | 5060(13) | 6667(11) | 7835(6) | 61(5) |
| C14 | 5532(15) | 6101(13) | 8011(7) | 69(5) |
| C15 | 5586(18) | 6031(16) | 8514(8) | 90(6) |
| C16 | 5197(19) | 6477(17) | 8820(8) | 101(6) |
| C17 | 4772(19) | 6984(17) | 8635(9) | 104(6) |
| C18 | 4704(17) | 7109(14) | 8151(8) | 87(7) |
| C13A | 4770(30) | 6520(30) | 7773(13) | 61(5) |
| C14A | 5160(30) | 5900(30) | 7958(15) | 73(9) |
| C15A | 5000(40) | 5710(30) | 8437(17) | 90(6) |
| C16A | 4520(40) | 6100(40) | 8713(16) | 101(6) |
| C17A | 4170(40) | 6650(30) | 8520(17) | 104(6) |
| C18A | 4290(30) | 6880(30) | 8057(17) | 80(9) |
| C19 | 8141(8) | 3660(10) | 5144(6) | 66(3) |
| C20 | 8015(10) | 2932(12) | 5000(7) | 83(4) |
| C21 | 8022(11) | 2760(13) | 4514(7) | 96(5) |
| C22 | 8144(11) | 3327(16) | 4167(7) | 101(5) |
| C23 | 8281(9) | 4007(14) | 4285(7) | 91(5) |
| C24 | 8286(8) | 4179(12) | 4788(6) | 75(4) |
| C25 | 9058(7) | 3923(9) | 5997(6) | 59(4) |
| C26 | 9152(8) | 3940(11) | 6496(6) | 77(5) |
| C27 | 9874(10) | 3990(13) | 6682(8) | 97(7) |
| C28 | 10460(8) | 4019(11) | 6378(9) | 97(6) |
| C29 | 10356(8) | 3982(12) | 5864(8) | 88(6) |
| C30 | 9645(8) | 3903(11) | 5682(7) | 76(5) |

| | | | | |
|---|---|---|---|---|
| O1S | 2864(5) | 7515(6) | 6801(4) | 63(3) |
| C1S | 2235(10) | 7958(12) | 6647(10) | 95(6) |
| O11 | 1887(9) | 2846(9) | 6515(6) | 122(6) |
| O12 | 2612(6) | 3709(7) | 6547(4) | 71(3) |
| O13 | 2163(9) | 3437(9) | 5853(6) | 114(5) |
| N5 | 2249(8) | 3325(8) | 6301(6) | 101(6) |
| O2S | 1622(9) | 3353(6) | 7462(5) | 86(4) |
| C2S | 1605(14) | 4105(12) | 7541(8) | 101(6) |

**Table 3 Anisotropic Displacement Parameters (Å$^2$×10$^3$) for TB(NO3)3-2 (MEOH SOLVATE). The Anisotropic displacement factor exponent takes the form: $-2\pi^2[h^2a^{*2}U_{11}+2hka^*b^*U_{12}+\ldots]$.**

| Atom | $U_{11}$ | $U_{22}$ | $U_{33}$ | $U_{23}$ | $U_{13}$ | $U_{12}$ |
|---|---|---|---|---|---|---|
| Tb1 | 38.3(4) | 53.5(4) | 53.7(4) | -13.3(4) | 2.0(4) | -1.6(3) |
| P1 | 53(2) | 52(2) | 30(6) | -6(5) | 0(5) | 1.1(17) |
| P1A | 53(2) | 52(2) | 30(6) | -6(5) | 0(5) | 1.1(17) |
| P2 | 35.4(15) | 62(2) | 53.8(18) | 12.1(16) | -1.0(14) | 4.7(14) |
| O1 | 31(4) | 67(6) | 54(5) | -14(4) | 2(4) | 0(4) |
| O2 | 46(5) | 52(5) | 64(6) | -17(4) | -10(4) | -1(4) |
| O3 | 40(4) | 61(5) | 61(5) | 27(4) | -2(4) | -4(4) |
| O4 | 33(4) | 59(5) | 71(6) | 17(5) | 10(4) | 2(4) |
| O5 | 107(9) | 69(7) | 51(6) | -8(5) | 15(6) | -10(6) |
| O6 | 53(5) | 67(6) | 55(5) | -4(5) | 2(4) | -3(5) |
| O7 | 117(10) | 67(7) | 81(8) | -3(6) | 25(7) | -5(7) |
| O8 | 45(5) | 76(6) | 62(6) | 4(5) | 15(4) | 6(5) |
| O9 | 38(5) | 58(6) | 78(7) | 0(5) | 3(4) | 2(4) |
| O10 | 74(7) | 102(9) | 71(7) | 7(7) | 5(6) | -3(7) |
| N1 | 37(5) | 56(6) | 45(6) | -3(5) | -2(4) | -2(4) |
| N2 | 37(5) | 44(5) | 48(5) | 1(4) | -3(4) | 1(4) |
| N3 | 66(8) | 74(9) | 57(7) | -10(7) | 7(6) | 10(7) |
| N4 | 54(7) | 77(8) | 55(7) | -8(6) | 4(6) | -6(6) |
| C1 | 44(6) | 39(6) | 56(7) | -5(5) | 2(6) | -3(5) |
| C2 | 49(7) | 58(7) | 43(7) | -2(6) | 3(5) | 7(6) |
| C3 | 30(5) | 57(7) | 48(6) | 7(6) | -4(5) | -4(5) |
| C4 | 33(6) | 47(7) | 63(8) | 12(6) | -2(5) | -5(5) |
| C5 | 39(6) | 42(6) | 51(7) | -2(5) | -3(5) | 3(5) |
| C6 | 38(6) | 44(6) | 40(6) | 0(5) | -2(5) | 2(5) |
| C7 | 82(10) | 56(8) | 42(7) | -18(6) | -3(7) | 1(7) |
| C8 | 65(10) | 73(11) | 106(14) | -3(10) | -25(10) | -15(8) |
| C9 | 107(17) | 91(15) | 150(20) | -55(15) | -30(15) | -9(13) |
| C10 | 88(15) | 96(16) | 160(20) | -65(16) | 46(16) | -37(13) |
| C11 | 150(20) | 59(10) | 79(13) | -5(9) | 33(13) | 6(12) |
| C12 | 90(11) | 52(8) | 59(8) | -10(7) | 15(8) | -1(8) |
| C13 | 80(13) | 61(10) | 41(7) | -5(7) | -10(8) | 8(9) |
| C14 | 90(14) | 69(12) | 49(8) | 2(8) | -10(10) | 16(10) |
| C15 | 120(15) | 96(13) | 55(8) | 17(8) | 0(10) | 15(10) |
| C16 | 144(16) | 106(13) | 53(8) | 7(9) | 10(10) | 14(11) |
| C17 | 146(16) | 112(13) | 55(8) | -9(10) | 14(11) | 28(11) |
| C18 | 125(17) | 86(14) | 50(9) | -5(9) | 16(11) | 32(12) |
| C13A | 80(13) | 61(10) | 41(7) | -5(7) | -10(8) | 8(9) |

| | | | | | | |
|---|---|---|---|---|---|---|
| C14A | 100(20) | 78(19) | 45(13) | 4(15) | -10(15) | 15(15) |
| C15A | 120(15) | 96(13) | 55(8) | 17(8) | 0(10) | 15(10) |
| C16A | 144(16) | 106(13) | 53(8) | 7(9) | 10(10) | 14(11) |
| C17A | 146(16) | 112(13) | 55(8) | -9(10) | 14(11) | 28(11) |
| C18A | 110(20) | 83(19) | 48(14) | -12(13) | 0(16) | 17(16) |
| C19 | 46(7) | 89(9) | 64(7) | 1(6) | 7(6) | 14(7) |
| C20 | 77(10) | 99(9) | 72(8) | -14(8) | 2(8) | 13(9) |
| C21 | 86(11) | 119(12) | 83(9) | -33(8) | -3(9) | 21(10) |
| C22 | 73(10) | 168(14) | 61(8) | -19(8) | 7(8) | 19(12) |
| C23 | 52(8) | 154(13) | 67(8) | 8(9) | 11(7) | 12(10) |
| C24 | 47(7) | 111(10) | 67(8) | 11(7) | 4(7) | 9(8) |
| C25 | 28(6) | 76(10) | 72(10) | 17(8) | 0(6) | -1(6) |
| C26 | 49(8) | 112(14) | 68(10) | 27(10) | -7(7) | -14(8) |
| C27 | 59(10) | 141(19) | 91(13) | 29(13) | -24(9) | 6(11) |
| C28 | 37(7) | 123(16) | 130(17) | 48(15) | 1(10) | 6(8) |
| C29 | 39(8) | 129(17) | 96(14) | 33(12) | 15(8) | 14(9) |
| C30 | 46(8) | 104(13) | 78(11) | 15(10) | -3(7) | 1(8) |
| O1S | 54(5) | 61(6) | 75(7) | -30(5) | 3(5) | 10(4) |
| C1S | 68(11) | 91(13) | 126(18) | -20(12) | 4(11) | 8(10) |
| O11 | 126(12) | 128(13) | 113(11) | 32(10) | 27(10) | -46(11) |
| O12 | 58(6) | 100(9) | 54(6) | 5(6) | 13(5) | 27(6) |
| O13 | 120(12) | 96(10) | 125(12) | 19(10) | 30(10) | -20(9) |
| N5 | 82(11) | 103(13) | 118(15) | 43(12) | 57(11) | 27(9) |
| O2S | 114(10) | 64(7) | 80(8) | 14(6) | 3(8) | -7(7) |
| C2S | 120(18) | 96(14) | 87(13) | 13(11) | 0(13) | -8(13) |

**Table 4 Bond Lengths for TB(NO3)3-2 (MEOH SOLVATE).**

| Atom | Atom | Length/Å | Atom | Atom | Length/Å |
|---|---|---|---|---|---|
| Tb1 | O1 | 2.365(8) | C5 | C6 | 1.519(17) |
| Tb1 | O2 | 2.308(9) | C7 | C8 | 1.43(2) |
| Tb1 | O3[1] | 2.372(8) | C7 | C12 | 1.36(2) |
| Tb1 | O4[1] | 2.332(8) | C8 | C9 | 1.39(3) |
| Tb1 | O5 | 2.503(11) | C9 | C10 | 1.36(4) |
| Tb1 | O6 | 2.521(9) | C10 | C11 | 1.33(3) |
| Tb1 | O8 | 2.450(10) | C11 | C12 | 1.46(3) |
| Tb1 | O9 | 2.534(10) | C13 | C14 | 1.43(3) |
| Tb1 | N3 | 2.919(14) | C13 | C18 | 1.35(3) |
| Tb1 | N4 | 2.916(14) | C14 | C15 | 1.39(3) |
| Tb1 | O1S | 2.392(9) | C15 | C16 | 1.37(3) |
| P1 | O2 | 1.48(2) | C16 | C17 | 1.31(4) |
| P1 | C2 | 1.76(2) | C17 | C18 | 1.35(3) |
| P1 | C7 | 1.75(3) | C13A | C14A | 1.43(3) |
| P1 | C13 | 1.88(2) | C13A | C18A | 1.35(3) |
| P1A | O2 | 1.57(7) | C14A | C15A | 1.39(3) |
| P1A | C2 | 1.89(6) | C15A | C16A | 1.37(4) |
| P1A | C7 | 1.83(7) | C16A | C17A | 1.31(4) |
| P1A | C13A | 1.59(6) | C17A | C18A | 1.35(3) |
| P2 | O4 | 1.505(9) | C19 | C20 | 1.41(3) |
| P2 | C4 | 1.800(13) | C19 | C24 | 1.39(2) |

| | | | | | |
|---|---|---|---|---|---|
| P2 | C19 | 1.777(16) | C20 | C21 | 1.37(2) |
| P2 | C25 | 1.801(13) | C21 | C22 | 1.42(3) |
| O1 | C1 | 1.245(15) | C22 | C23 | 1.31(3) |
| O3 | C3 | 1.255(14) | C23 | C24 | 1.42(2) |
| O5 | N3 | 1.267(15) | C25 | C26 | 1.38(2) |
| O6 | N3 | 1.212(16) | C25 | C30 | 1.38(2) |
| O7 | N3 | 1.254(17) | C26 | C27 | 1.42(2) |
| O8 | N4 | 1.291(15) | C27 | C28 | 1.36(3) |
| O9 | N4 | 1.245(15) | C28 | C29 | 1.42(3) |
| O10 | N4 | 1.250(17) | C29 | C30 | 1.40(2) |
| N1 | C1 | 1.355(15) | O1S | C1S | 1.47(2) |
| N1 | C5 | 1.429(15) | O11 | N5 | 1.246(11) |
| N2 | C3 | 1.307(15) | O12 | N5 | 1.178(12) |
| N2 | C6 | 1.464(14) | O13 | N5 | 1.255(12) |
| C1 | C2 | 1.545(17) | O2S | C2S | 1.39(2) |
| C3 | C4 | 1.527(15) | | | |

[1]1-X,1-Y,+Z

**Table 5 Bond Angles for TB(NO3)3-2 (MEOH SOLVATE).**

| Atom | Atom | Atom | Angle/° | Atom | Atom | Atom | Angle/° |
|---|---|---|---|---|---|---|---|
| O1 | Tb1 | O3[1] | 75.4(3) | P2 | O4 | Tb1[1] | 135.4(5) |
| O1 | Tb1 | O5 | 78.2(4) | N3 | O5 | Tb1 | 95.9(9) |
| O1 | Tb1 | O6 | 73.7(3) | N3 | O6 | Tb1 | 96.5(8) |
| O1 | Tb1 | O8 | 125.2(3) | N4 | O8 | Tb1 | 97.5(7) |
| O1 | Tb1 | O9 | 74.1(3) | N4 | O9 | Tb1 | 94.8(8) |
| O1 | Tb1 | N3 | 73.7(3) | C1 | N1 | C5 | 121.7(11) |
| O1 | Tb1 | N4 | 99.3(3) | C3 | N2 | C6 | 122.6(10) |
| O1 | Tb1 | O1S | 138.5(3) | O5 | N3 | Tb1 | 58.5(8) |
| O2 | Tb1 | O1 | 75.4(3) | O6 | N3 | Tb1 | 59.1(7) |
| O2 | Tb1 | O3[1] | 139.3(3) | O6 | N3 | O5 | 117.6(13) |
| O2 | Tb1 | O4[1] | 141.5(3) | O6 | N3 | O7 | 121.4(13) |
| O2 | Tb1 | O5 | 78.3(4) | O7 | N3 | Tb1 | 178.9(12) |
| O2 | Tb1 | O6 | 123.5(4) | O7 | N3 | O5 | 121.0(14) |
| O2 | Tb1 | O8 | 90.6(4) | O8 | N4 | Tb1 | 56.4(7) |
| O2 | Tb1 | O9 | 72.9(3) | O9 | N4 | Tb1 | 60.0(7) |
| O2 | Tb1 | N3 | 101.4(4) | O9 | N4 | O8 | 116.2(12) |
| O2 | Tb1 | N4 | 79.6(4) | O9 | N4 | O10 | 121.9(13) |
| O2 | Tb1 | O1S | 71.0(3) | O10 | N4 | Tb1 | 175.7(10) |
| O3[1] | Tb1 | O5 | 122.1(3) | O10 | N4 | O8 | 121.9(12) |
| O3[1] | Tb1 | O6 | 73.3(3) | O1 | C1 | N1 | 121.6(12) |
| O3[1] | Tb1 | O8 | 83.6(3) | O1 | C1 | C2 | 122.0(11) |
| O3[1] | Tb1 | O9 | 72.3(3) | N1 | C1 | C2 | 116.3(11) |
| O3[1] | Tb1 | N3 | 97.0(4) | C1 | C2 | P1 | 107.7(10) |
| O3[1] | Tb1 | N4 | 77.8(3) | C1 | C2 | P1A | 115.2(19) |
| O3[1] | Tb1 | O1S | 145.4(3) | O3 | C3 | N2 | 123.9(10) |
| O4[1] | Tb1 | O1 | 141.8(3) | O3 | C3 | C4 | 118.5(10) |
| O4[1] | Tb1 | O3[1] | 75.3(3) | N2 | C3 | C4 | 117.6(11) |
| O4[1] | Tb1 | O5 | 97.3(4) | C3 | C4 | P2 | 111.7(9) |

| | | | | | | | |
|---|---|---|---|---|---|---|---|
| O4[1] | Tb1 | O6 | 74.8(3) | N1 | C5 | C6 | 110.4(10) |
| O4[1] | Tb1 | O8 | 74.8(3) | N2 | C6 | C5 | 108.0(10) |
| O4[1] | Tb1 | O9 | 118.6(3) | C8 | C7 | P1 | 119.7(14) |
| O4[1] | Tb1 | N3 | 86.1(4) | C8 | C7 | P1A | 117(2) |
| O4[1] | Tb1 | N4 | 97.9(4) | C12 | C7 | P1 | 120.6(15) |
| O4[1] | Tb1 | O1S | 71.4(3) | C12 | C7 | P1A | 123(3) |
| O5 | Tb1 | O6 | 49.9(3) | C12 | C7 | C8 | 119.7(16) |
| O5 | Tb1 | O9 | 144.0(4) | C9 | C8 | C7 | 121.7(19) |
| O5 | Tb1 | N3 | 25.6(3) | C10 | C9 | C8 | 118(2) |
| O5 | Tb1 | N4 | 157.7(4) | C11 | C10 | C9 | 122(2) |
| O6 | Tb1 | O9 | 137.5(3) | C10 | C11 | C12 | 122(2) |
| O6 | Tb1 | N3 | 24.4(3) | C7 | C12 | C11 | 116.3(18) |
| O6 | Tb1 | N4 | 151.1(3) | C14 | C13 | P1 | 121.9(16) |
| O8 | Tb1 | O5 | 151.0(3) | C18 | C13 | P1 | 117.3(16) |
| O8 | Tb1 | O6 | 145.6(3) | C18 | C13 | C14 | 120.4(17) |
| O8 | Tb1 | O9 | 51.2(3) | C15 | C14 | C13 | 116(2) |
| O8 | Tb1 | N3 | 160.2(3) | C16 | C15 | C14 | 121(2) |
| O8 | Tb1 | N4 | 26.0(3) | C17 | C16 | C15 | 120(2) |
| O9 | Tb1 | N3 | 147.7(3) | C16 | C17 | C18 | 123(2) |
| O9 | Tb1 | N4 | 25.2(3) | C17 | C18 | C13 | 119(2) |
| N4 | Tb1 | N3 | 172.3(3) | C14A | C13A | P1A | 121(4) |
| O1S | Tb1 | O5 | 71.8(4) | C18A | C13A | P1A | 117(4) |
| O1S | Tb1 | O6 | 105.8(4) | C18A | C13A | C14A | 121(2) |
| O1S | Tb1 | O8 | 79.2(4) | C15A | C14A | C13A | 116(2) |
| O1S | Tb1 | O9 | 116.7(4) | C16A | C15A | C14A | 122(3) |
| O1S | Tb1 | N3 | 89.7(4) | C17A | C16A | C15A | 120(3) |
| O1S | Tb1 | N4 | 97.9(4) | C16A | C17A | C18A | 123(3) |
| O2 | P1 | C2 | 116.0(13) | C17A | C18A | C13A | 119(3) |
| O2 | P1 | C7 | 112.0(14) | C20 | C19 | P2 | 119.6(13) |
| O2 | P1 | C13 | 111.8(13) | C24 | C19 | P2 | 121.5(14) |
| C2 | P1 | C13 | 105.2(13) | C24 | C19 | C20 | 118.9(16) |
| C7 | P1 | C2 | 108.1(13) | C21 | C20 | C19 | 119(2) |
| C7 | P1 | C13 | 102.6(13) | C20 | C21 | C22 | 119(2) |
| O2 | P1A | C2 | 105(3) | C23 | C22 | C21 | 123.8(19) |
| O2 | P1A | C7 | 104(3) | C22 | C23 | C24 | 117(2) |
| O2 | P1A | C13A | 104(4) | C19 | C24 | C23 | 122(2) |
| C7 | P1A | C2 | 99(3) | C26 | C25 | P2 | 117.0(11) |
| C13A | P1A | C2 | 111(4) | C30 | C25 | P2 | 121.3(12) |
| C13A | P1A | C7 | 131(4) | C30 | C25 | C26 | 121.7(14) |
| O4 | P2 | C4 | 110.1(6) | C25 | C26 | C27 | 118.3(16) |
| O4 | P2 | C19 | 113.4(7) | C28 | C27 | C26 | 121.0(18) |
| O4 | P2 | C25 | 111.5(6) | C27 | C28 | C29 | 120.0(16) |
| C19 | P2 | C4 | 108.5(7) | C30 | C29 | C28 | 118.8(15) |
| C19 | P2 | C25 | 109.3(7) | C25 | C30 | C29 | 119.9(17) |
| C25 | P2 | C4 | 103.6(7) | C1S | O1S | Tb1 | 130.4(11) |
| C1 | O1 | Tb1 | 139.0(8) | O11 | N5 | O13 | 120.6(17) |
| P1 | O2 | Tb1 | 134.8(8) | O12 | N5 | O11 | 116.6(17) |
| P1A | O2 | Tb1 | 142(2) | O12 | N5 | O13 | 122.3(14) |
| C3 | O3 | Tb1[1] | 142.0(8) | | | | |

[1]1-X,1-Y,+Z

**Table 6 Hydrogen Bonds for TB(NO3)3-2 (MEOH SOLVATE).**

| D | H | A | d(D-H)/Å | d(H-A)/Å | d(D-A)/Å | D-H-A/° |
|---|---|---|---|---|---|---|
| N1 | H1 | O12[1] | 0.88 | 2.06 | 2.941(14) | 176.1 |
| N2 | H2 | O6[2] | 0.88 | 2.46 | 3.025(14) | 122.2 |
| N2 | H2 | O7[2] | 0.88 | 2.26 | 3.093(16) | 157.9 |
| N2 | H2 | O13[1] | 0.88 | 2.55 | 3.010(18) | 113.7 |
| C6 | H6B | O6[2] | 0.99 | 2.57 | 3.087(15) | 112.2 |
| C17A | H17A | O11[3] | 0.95 | 2.24 | 2.92(5) | 127.3 |
| C18A | H18A | O11[3] | 0.95 | 2.47 | 3.02(5) | 116.1 |
| O1S | H1S | O2S[3] | 0.875(14) | 1.84(2) | 2.704(16) | 169(6) |
| O2S | H2S | O11 | 0.84 | 1.98 | 2.80(2) | 163.5 |

[1]1-X,1-Y,+Z; [2]+Y,1-X,1-Z; [3]1/2-X,1/2+Y,3/2-Z

**Table 7 Hydrogen Atom Coordinates (Å×10$^4$) and Isotropic Displacement Parameters (Å$^2$×10$^3$) for TB(NO3)3-2 (MEOH SOLVATE).**

| Atom | *x* | *y* | *z* | U(eq) |
|---|---|---|---|---|
| H1 | 6409.03 | 6340.5 | 6175.34 | 55 |
| H2 | 7200.79 | 5692.69 | 5264.66 | 52 |
| H2AA | 6014.52 | 6241.58 | 6944.39 | 60 |
| H2AB | 5305.06 | 5724.98 | 6970.01 | 60 |
| H2BC | 6017.43 | 6246.09 | 6934.13 | 60 |
| H2BD | 5328.7 | 5710.68 | 6956.19 | 60 |
| H4A | 7880.41 | 4975.97 | 6179.63 | 57 |
| H4B | 8123.23 | 5141.16 | 5629.21 | 57 |
| H5A | 6303.71 | 6737.73 | 5390.9 | 53 |
| H5B | 5447.79 | 6552.01 | 5413.16 | 53 |
| H6A | 5750.75 | 5267.41 | 5429.83 | 49 |
| H6B | 6012.15 | 5652.34 | 4932.82 | 49 |
| H8 | 6491.56 | 7250.81 | 7368.13 | 98 |
| H9 | 7186.85 | 8328.73 | 7388.46 | 137 |
| H10 | 6691.05 | 9369.23 | 7040.08 | 139 |
| H11 | 5486.32 | 9444.45 | 6881.47 | 116 |
| H12 | 4713.96 | 8371.98 | 6887.6 | 81 |
| H14 | 5793.63 | 5789.62 | 7795.57 | 83 |
| H15 | 5897.73 | 5668.03 | 8648.88 | 108 |
| H16 | 5235.25 | 6420.11 | 9163.23 | 122 |
| H17 | 4496.29 | 7279.21 | 8853.65 | 125 |
| H18 | 4412.07 | 7501.81 | 8035.14 | 104 |
| H14A | 5501.89 | 5633.73 | 7765.96 | 88 |
| H15A | 5240.82 | 5295.6 | 8577.04 | 108 |
| H16A | 4431.96 | 5964.32 | 9043.12 | 122 |
| H17A | 3821.57 | 6906.11 | 8713.4 | 125 |
| H18A | 4028.77 | 7292.16 | 7932.78 | 96 |
| H20 | 7926.46 | 2564.09 | 5237.39 | 99 |
| H21 | 7946.59 | 2270 | 4409.99 | 115 |
| H22 | 8125.79 | 3203.81 | 3830.39 | 121 |

| | | | | |
|---|---|---|---|---|
| H23 | 8372.34 | 4368.95 | 4043.63 | 109 |
| H24 | 8393.19 | 4666.04 | 4885.99 | 90 |
| H26 | 8743.05 | 3920.14 | 6709.44 | 92 |
| H27 | 9949.37 | 4002.55 | 7024.93 | 116 |
| H28 | 10939.47 | 4063.49 | 6507.88 | 116 |
| H29 | 10761.57 | 4009.86 | 5647.83 | 106 |
| H30 | 9567.55 | 3836.88 | 5342.27 | 91 |
| H1S | 3090(30) | 7770(50) | 7030(30) | 95 |
| H1SA | 2350.95 | 8206.34 | 6340.22 | 143 |
| H1SB | 2125.58 | 8322.03 | 6899.12 | 143 |
| H1SC | 1809.47 | 7640.1 | 6600.2 | 143 |
| H2S | 1644.11 | 3269.4 | 7161.3 | 129 |
| H2SA | 1207.63 | 4224.42 | 7767.46 | 151 |
| H2SB | 1524.82 | 4357.92 | 7230.61 | 151 |
| H2SC | 2072.26 | 4263.24 | 7681.71 | 151 |

**Table 8 Atomic Occupancy for TB(NO3)3-2 (MEOH SOLVATE).**

| Atom | *Occupancy* | Atom | *Occupancy* | Atom | *Occupancy* |
|---|---|---|---|---|---|
| P1 | 0.733(17) | P1A | 0.267(17) | H2AA | 0.733(17) |
| H2AB | 0.733(17) | H2BC | 0.267(17) | H2BD | 0.267(17) |
| C13 | 0.733(17) | C14 | 0.733(17) | H14 | 0.733(17) |
| C15 | 0.733(17) | H15 | 0.733(17) | C16 | 0.733(17) |
| H16 | 0.733(17) | C17 | 0.733(17) | H17 | 0.733(17) |
| C18 | 0.733(17) | H18 | 0.733(17) | C13A | 0.267(17) |
| C14A | 0.267(17) | H14A | 0.267(17) | C15A | 0.267(17) |
| H15A | 0.267(17) | C16A | 0.267(17) | H16A | 0.267(17) |
| C17A | 0.267(17) | H17A | 0.267(17) | C18A | 0.267(17) |
| H18A | 0.267(17) | | | | |

**Crystal structure determination of [TB(NO3)3-2 (MEOH SOLVATE)]**

**Crystal Data** for $C_{64}H_{76}N_{10}O_{30}P_4Tb_2$ ($M$ =1907.06 g/mol): tetragonal, space group P-42₁c (no. 114), $a$ = 18.2898(4) Å, $c$ = 27.4073(6) Å, $V$ = 9168.2(4) Å$^3$, $Z$ = 4, $T$ = 172.97 K, μ(CuKα) = 8.794 mm$^{-1}$, $Dcalc$ = 1.382 g/cm$^3$, 111590 reflections measured (5.81° ≤ 2Θ ≤ 136.676°), 8408 unique ($R_{int}$ = 0.1335, $R_{sigma}$ = 0.0634) which were used in all calculations. The final $R_1$ was 0.0638 (I > 2σ(I)) and $wR_2$ was 0.2052 (all data).

**Refinement model description**

Number of restraints – 165, number of constraints – unknown.
Details:
1. Fixed Uiso
At 1.2 times of:
All C(H) groups, All C(H,H) groups, All C(H,H,H,H) groups, All N(H) groups
At 1.5 times of:
All C(H,H,H) groups, All O(H) groups
2. Restrained distances
O12-N5 = O11-N5 = O13-N5
1.25 with sigma of 0.01
O1S-H1S
0.87 with sigma of 0.01
C1S-H1S
1.931945 with sigma of 0.02
Tb1-H1S

2.938614 with sigma of 0.02
3. Restrained planarity
C13A, C14A, C15A, C16A, C17A, C18A
with sigma of 0.1
4. Rigid bond restraints
C13A, C18A, C17A, C16A, C15A, C14A
with sigma for 1-2 distances of 0.01 and sigma for 1-3 distances of 0.01
C13, C18, C17, C16, C15, C14
with sigma for 1-2 distances of 0.01 and sigma for 1-3 distances of 0.01
C19, C20, C21, C22, C23, C24
with sigma for 1-2 distances of 0.01 and sigma for 1-3 distances of 0.01
5. Uiso/Uaniso restraints and constraints
C13A ≈ C18A ≈ C17A ≈ C16A ≈ C15A ≈ C14A: within 2A with sigma of 0.02 and sigma for terminal atoms of 0.04
C13 ≈ C18 ≈ C17 ≈ C16 ≈ C15 ≈ C14: within 2A with sigma of 0.02 and sigma for terminal atoms of 0.04
C19 ≈ C20 ≈ C21 ≈ C22 ≈ C23 ≈ C24: within 2A with sigma of 0.02 and sigma for terminal atoms of 0.04
Uanis(C13) = Uanis(C13A)
Uanis(C17) = Uanis(C17A)
Uanis(C16) = Uanis(C16A)
Uanis(C15) = Uanis(C15A)
Uanis(P1A) = Uanis(P1)
6. Same fragment restrains
{C13, C14, C15, C16, C17, C18} sigma for 1-2: 0.01, 1-3: 0.02
as
{C13A, C14A, C15A, C16A, C17A, C18A}
7. Others
Sof(P1A)=Sof(H2BC)=Sof(H2BD)=Sof(C13A)=Sof(C14A)=Sof(H14A)=Sof(C15A)=
Sof(H15A)=Sof(C16A)=Sof(H16A)=Sof(C17A)=Sof(H17A)=Sof(C18A)=Sof(H18A)=1-FVAR(1)
Sof(P1)=Sof(H2AA)=Sof(H2AB)=Sof(C13)=Sof(C14)=Sof(H14)=Sof(C15)=Sof(H15)=
Sof(C16)=Sof(H16)=Sof(C17)=Sof(H17)=Sof(C18)=Sof(H18)=FVAR(1)
8.a Secondary CH2 refined with riding coordinates:
C2(H2AA,H2AB), C2(H2BC,H2BD), C4(H4A,H4B), C5(H5A,H5B), C6(H6A,H6B)
8.b Aromatic/amide H refined with riding coordinates:
N1(H1), N2(H2), C8(H8), C9(H9), C10(H10), C11(H11), C12(H12), C14(H14),
C15(H15), C16(H16), C17(H17), C18(H18), C14A(H14A), C15A(H15A), C16A(H16A),
C17A(H17A), C18A(H18A), C20(H20), C21(H21), C22(H22), C23(H23), C24(H24),
C26(H26), C27(H27), C28(H28), C29(H29), C30(H30)
8.c Idealised Me refined as rotating group:
C1S(H1SA,H1SB,H1SC), C2S(H2SA,H2SB,H2SC)
8.d Idealised tetrahedral OH refined as rotating group:
O2S(H2S)

**D. $Tb(NO_3)_3$-1**

The figure on the left depicts the atom numbering scheme of the 2:2 $Tb(NO_3)_3$-1 complex using a ball and stick model with standard CPK colors (Tb atoms are colored green). Only one ligand of the major component is shown for clarity, although all atoms of the inner coordination sphere of each metal center are shown. Hydrogen atoms bonded to carbon atoms are not shown for clarity, and hydrogen bonds are depicted with blue, dashed lines.
The figure on the right depicts the thermal ellipsoids of all atoms of the 2:2 complex, which are shown at the 40% probability level.

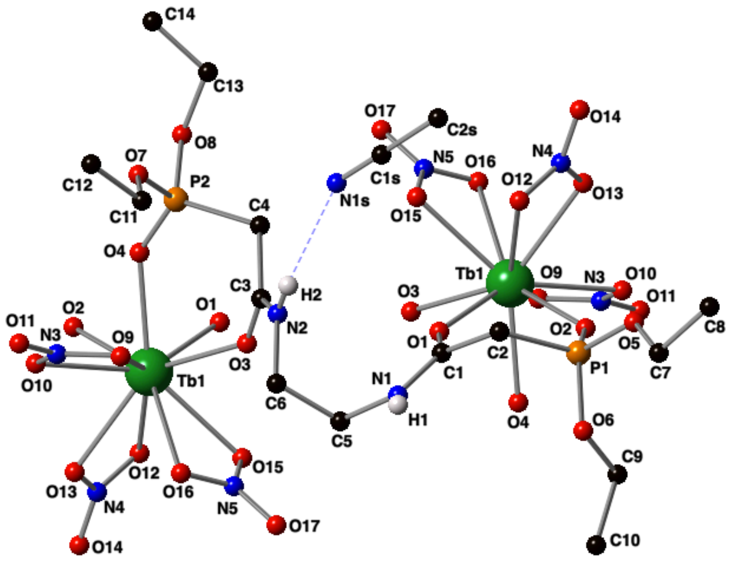


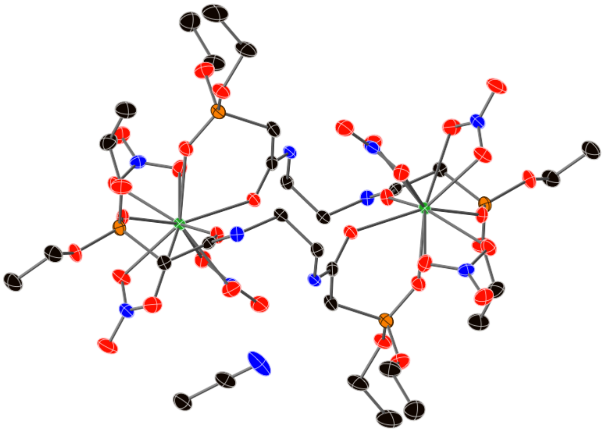

**Refinement notes**

(1) The electron density corresponding to C9 of one ethyl group was positionally disordered. This disordered was modeled over two positions, and the relative occupancy of each component was determined to be 0.610(12) to 0.390(12) by refining against a free variable.

**Experimental**

Single crystals of $C_{32}H_{66}N_{12}O_{34}P_4Tb_2$ (**$Tb(NO_3)_3$-1)** were grown by vapor diffusion of diethyl ether into a solution of the complex in acetonitrile. A suitable crystal was selected and mounted on a nylon loop using a small amount of paratone oil on a Bruker APEX-II CCD diffractometer. The crystal was kept at 173(2) K during data collection. Using Olex2 [1], the structure was solved with the ShelXT [2] structure solution program using Intrinsic Phasing and refined with the ShelXL [3] refinement package using Least Squares minimization.

1. Dolomanov, O.V., Bourhis, L.J., Gildea, R.J, Howard, J.A.K. & Puschmann, H. (2009), J. Appl. Cryst. 42, 339-341.
2. Sheldrick, G.M. (2015). Acta Cryst. A71, 3-8.
3. Sheldrick, G.M. (2015). Acta Cryst. C71, 3-8.

**$Tb(NO_3)_3$-1**

**Table 1 Crystal data and structure refinement for $Tb(NO_3)_3$-1**

| | |
|---|---|
| CCDC number | 2003373 |
| Empirical formula | $C_{32}H_{66}N_{12}O_{34}P_4Tb_2$ |
| Formula weight | 1604.68 |

| | |
|---|---|
| Temperature/K | 173(2) |
| Crystal system | monoclinic |
| Space group | C2/c |
| a/Å | 32.3189(5) |
| b/Å | 12.5255(2) |
| c/Å | 16.4158(3) |
| α/° | 90 |
| β/° | 116.1620(10) |
| γ/° | 90 |
| Volume/Å$^3$ | 5964.48(18) |
| Z | 4 |
| $\rho_{calc}$g/cm$^3$ | 1.787 |
| μ/mm$^{-1}$ | 13.430 |
| F(000) | 3216.0 |
| Crystal size/mm$^3$ | 0.264 × 0.173 × 0.103 |
| Radiation | CuKα (λ = 1.54178) |
| 2Θ range for data collection/° | 6.094 to 140.374 |
| Index ranges | -39 ≤ h ≤ 39, -15 ≤ k ≤ 15, -17 ≤ l ≤ 19 |
| Reflections collected | 44653 |
| Independent reflections | 5582 [$R_{int}$ = 0.0588, $R_{sigma}$ = 0.0348] |
| Data/restraints/parameters | 5582/0/402 |
| Goodness-of-fit on F$^2$ | 1.086 |
| Final R indexes [I>=2σ (I)] | $R_1$ = 0.0287, w$R_2$ = 0.0636 |
| Final R indexes [all data] | $R_1$ = 0.0336, w$R_2$ = 0.0653 |
| Largest diff. peak/hole / e Å$^{-3}$ | 0.57/-0.75 |

**Table 2 Fractional Atomic Coordinates (×10$^4$) and Equivalent Isotropic Displacement Parameters (Å$^2$×10$^3$) for TB(NO3)3-1. $U_{eq}$ is defined as 1/3 of of the trace of the orthogonalised $U_{IJ}$ tensor.**

| Atom | *x* | *y* | *z* | U(eq) |
|---|---|---|---|---|
| Tb1 | 3494.3(2) | 3928.6(2) | 6790.8(2) | 16.92(6) |
| P1 | 4369.2(3) | 4582.5(7) | 6087.1(6) | 22.55(18) |
| P2 | 1288.3(3) | 3916.2(7) | 3394.1(6) | 25.04(18) |
| O1 | 3354.7(8) | 3753.4(19) | 5248.3(15) | 24.4(5) |
| O2 | 4217.2(8) | 4340.2(19) | 6793.9(16) | 24.5(5) |
| O3 | 2124.9(8) | 2341.3(18) | 3770.2(16) | 24.9(5) |
| O4 | 1154.7(8) | 2781.3(19) | 3209.2(17) | 29.6(6) |
| O5 | 4691.2(8) | 5574(2) | 6365.2(17) | 29.2(6) |
| O6 | 4628.0(10) | 3658(2) | 5868(2) | 38.4(7) |
| O7 | 1098.5(10) | 4649(2) | 2535.1(19) | 38.4(6) |
| O8 | 1066.0(9) | 4411(2) | 3980.4(19) | 32.9(6) |
| O9 | 3491.2(8) | 2762(2) | 8087.8(17) | 32.9(6) |
| O10 | 4086.9(9) | 3749(2) | 8428.6(17) | 31.7(6) |
| O11 | 4063.5(10) | 2649(2) | 9432.3(18) | 40.1(7) |
| O12 | 3504.6(10) | 5948(2) | 6201.3(18) | 37.7(6) |
| O13 | 3690.3(10) | 5689.1(19) | 7612.7(18) | 31.4(6) |
| O14 | 3596.9(11) | 7315(2) | 7084(2) | 46.0(8) |
| O15 | 2711.2(9) | 4819(2) | 5922.6(19) | 38.2(6) |

| | | | | |
|---|---|---|---|---|
| O16 | 2883.4(9) | 4493(2) | 7319.8(18) | 33.1(6) |
| O17 | 2176.0(9) | 4888(2) | 6376(2) | 43.9(7) |
| N1 | 3433.4(10) | 3503(2) | 3969(2) | 23.1(6) |
| N2 | 2405.3(10) | 3725(2) | 3300(2) | 23.9(6) |
| N3 | 3885.2(10) | 3039(2) | 8674(2) | 26.7(6) |
| N4 | 3597.7(10) | 6350(2) | 6960(2) | 28.1(7) |
| N5 | 2579.5(11) | 4734(2) | 6538(2) | 29.9(7) |
| C1 | 3535.0(11) | 3993(3) | 4741(2) | 21.3(7) |
| C2 | 3895.3(12) | 4871(3) | 5005(2) | 24.0(7) |
| C3 | 2156.9(11) | 3322(3) | 3687(2) | 23.3(7) |
| C4 | 1902.6(12) | 4087(3) | 4014(3) | 28.0(8) |
| C5 | 3098.8(12) | 2641(3) | 3619(2) | 23.1(7) |
| C6 | 2634.7(12) | 3027(3) | 2917(2) | 23.6(7) |
| C7 | 5031.0(14) | 5794(3) | 6028(3) | 37.1(9) |
| C8 | 5257.3(15) | 6821(3) | 6429(4) | 48.5(12) |
| C9 | 4884(2) | 2830(6) | 6503(5) | 39(2) |
| C10 | 4979.3(17) | 1941(4) | 6010(4) | 55.0(13) |
| C11 | 1316.7(16) | 4895(3) | 1956(3) | 43.1(10) |
| C12 | 979.7(17) | 5530(4) | 1181(3) | 51.5(12) |
| C13 | 1088.6(16) | 5571(3) | 4144(3) | 44.9(11) |
| C14 | 624(2) | 6041(4) | 3674(5) | 74.7(18) |
| C9A | 4582(4) | 2605(8) | 5893(8) | 37(3) |
| N1S | 2481.1(18) | 6111(3) | 3770(3) | 62.1(12) |
| C1S | 2702.8(16) | 6812(3) | 4143(3) | 37.4(9) |
| C2S | 2983.7(16) | 7722(3) | 4599(3) | 42.8(10) |

**Table 3 Anisotropic Displacement Parameters (Å$^2$×10$^3$) for TB(NO3)3-1. The Anisotropic displacement factor exponent takes the form: $-2\pi^2[h^2a^{*2}U_{11}+2hka^*b^*U_{12}+\ldots]$.**

| Atom | $U_{11}$ | $U_{22}$ | $U_{33}$ | $U_{23}$ | $U_{13}$ | $U_{12}$ |
|---|---|---|---|---|---|---|
| Tb1 | 19.61(10) | 16.49(10) | 17.27(10) | -1.45(7) | 10.53(7) | -1.74(8) |
| P1 | 20.6(4) | 24.7(4) | 24.2(5) | -2.8(3) | 11.6(3) | -4.5(3) |
| P2 | 25.9(4) | 21.5(4) | 31.8(5) | -2.9(4) | 16.4(4) | -0.4(4) |
| O1 | 27.4(12) | 29.8(13) | 20.1(12) | -2.9(9) | 14.1(10) | -7.8(10) |
| O2 | 20.3(12) | 31.2(13) | 22.0(12) | -1.1(9) | 9.2(9) | -5.1(10) |
| O3 | 24.4(12) | 21.8(13) | 30.2(13) | -1.7(10) | 13.7(10) | -3.4(10) |
| O4 | 24.7(13) | 26.4(13) | 39.5(15) | -6.4(10) | 15.7(11) | -1.2(10) |
| O5 | 26.6(13) | 33.3(14) | 33.3(14) | -8.5(11) | 18.3(11) | -11.8(11) |
| O6 | 48.2(17) | 32.2(15) | 45.3(17) | 3.7(12) | 30.2(14) | 9.6(12) |
| O7 | 37.8(15) | 41.2(16) | 40.6(16) | 9.5(12) | 21.2(13) | 7.6(13) |
| O8 | 36.5(14) | 25.6(13) | 45.7(17) | -7.7(11) | 26.4(13) | -2.5(11) |
| O9 | 24.5(13) | 40.8(16) | 30.4(14) | 4.6(11) | 9.5(11) | -3.2(11) |
| O10 | 38.5(15) | 28.4(14) | 27.7(14) | 0.6(10) | 14.0(11) | -8.6(11) |
| O11 | 46.6(17) | 44.3(17) | 24.4(14) | 13.5(12) | 11.3(12) | 3.0(13) |
| O12 | 47.1(16) | 35.4(15) | 29.0(15) | -0.8(12) | 15.3(12) | 6.3(13) |
| O13 | 48.1(16) | 23.2(12) | 33.4(15) | -2.4(10) | 27.5(12) | -4.1(11) |
| O14 | 62(2) | 21.4(14) | 53.3(19) | -3.3(12) | 24.7(15) | 0.8(13) |
| O15 | 34.5(15) | 39.1(16) | 43.5(17) | 11.1(12) | 19.4(13) | 6.6(12) |
| O16 | 33.4(14) | 34.2(14) | 33.3(15) | -6.0(11) | 16.0(12) | 2.8(11) |
| O17 | 27.9(15) | 33.5(15) | 75(2) | -1.2(14) | 26.8(14) | 2.7(12) |

| | | | | | | |
|---|---|---|---|---|---|---|
| N1 | 25.4(15) | 28.2(15) | 20.2(16) | -1.2(12) | 14.2(12) | -2.8(12) |
| N2 | 24.8(15) | 17.5(15) | 29.5(16) | -1.0(11) | 11.8(12) | -2.6(12) |
| N3 | 31.4(16) | 26.5(16) | 24.1(16) | 2.7(12) | 13.9(13) | 6.3(13) |
| N4 | 30.2(16) | 21.3(15) | 36.2(19) | -3.6(12) | 17.9(14) | -2.7(12) |
| N5 | 29.7(17) | 17.3(14) | 46(2) | -1.9(13) | 19.3(15) | -1.3(12) |
| C1 | 20.8(16) | 25.1(17) | 16.7(16) | 2.7(13) | 7.1(12) | 2.6(14) |
| C2 | 27.4(18) | 26.2(18) | 21.7(18) | 0.2(13) | 13.8(14) | -2.5(14) |
| C3 | 20.1(16) | 24.4(18) | 23.7(18) | -4.4(13) | 7.9(13) | -3.6(14) |
| C4 | 27.5(18) | 24.2(18) | 35(2) | -8.8(15) | 16.7(15) | -6.7(15) |
| C5 | 27.2(18) | 19.8(17) | 25.7(18) | -2.3(13) | 14.8(14) | -2.4(14) |
| C6 | 25.5(17) | 24.4(18) | 22.6(18) | -1.6(13) | 12.0(14) | -4.3(14) |
| C7 | 33(2) | 35(2) | 54(3) | 1.4(18) | 29.2(19) | -2.6(17) |
| C8 | 34(2) | 35(2) | 84(4) | -1(2) | 32(2) | -8.8(19) |
| C9 | 26(4) | 42(4) | 48(5) | 4(3) | 17(4) | 6(3) |
| C10 | 51(3) | 35(2) | 75(4) | -4(2) | 24(3) | 9(2) |
| C11 | 54(3) | 35(2) | 48(3) | 11.0(19) | 30(2) | 6(2) |
| C12 | 55(3) | 53(3) | 44(3) | 10(2) | 20(2) | 1(2) |
| C13 | 54(3) | 27(2) | 63(3) | -14.2(19) | 35(2) | -2.8(19) |
| C14 | 71(4) | 41(3) | 113(5) | -8(3) | 41(4) | 21(3) |
| C9A | 40(7) | 25(5) | 50(8) | -6(4) | 25(6) | -2(4) |
| N1S | 103(4) | 40(2) | 58(3) | -14(2) | 49(3) | -31(2) |
| C1S | 60(3) | 27(2) | 36(2) | 3.6(17) | 31(2) | -1.8(19) |
| C2S | 58(3) | 31(2) | 38(2) | -0.5(17) | 19(2) | -6.4(19) |

**Table 4 Bond Lengths for TB(NO3)3-1.**

| Atom | Atom | Length/Å | Atom | Atom | Length/Å |
|---|---|---|---|---|---|
| Tb1 | O1 | 2.378(2) | O7 | C11 | 1.445(5) |
| Tb1 | O2 | 2.390(2) | O8 | C13 | 1.472(5) |
| Tb1 | $O3^1$ | 2.400(2) | O9 | N3 | 1.260(4) |
| Tb1 | $O4^1$ | 2.424(2) | O10 | N3 | 1.268(4) |
| Tb1 | O9 | 2.586(3) | O11 | N3 | 1.220(4) |
| Tb1 | O10 | 2.531(3) | O12 | N4 | 1.251(4) |
| Tb1 | O12 | 2.714(3) | O13 | N4 | 1.281(4) |
| Tb1 | O13 | 2.516(2) | O14 | N4 | 1.226(4) |
| Tb1 | O15 | 2.549(3) | O15 | N5 | 1.262(4) |
| Tb1 | O16 | 2.581(3) | O16 | N5 | 1.261(4) |
| P1 | O2 | 1.477(2) | O17 | N5 | 1.226(4) |
| P1 | O5 | 1.554(2) | N1 | C1 | 1.312(4) |
| P1 | O6 | 1.560(3) | N1 | C5 | 1.455(4) |
| P1 | C2 | 1.797(3) | N2 | C3 | 1.324(5) |
| P2 | O4 | 1.478(3) | N2 | C6 | 1.455(4) |
| P2 | O7 | 1.564(3) | C1 | C2 | 1.519(5) |
| P2 | O8 | 1.561(3) | C3 | C4 | 1.507(5) |
| P2 | C4 | 1.800(4) | C5 | C6 | 1.513(5) |
| O1 | C1 | 1.246(4) | C7 | C8 | 1.483(6) |
| O3 | $Tb1^1$ | 2.400(2) | C9 | C10 | 1.487(8) |
| O3 | C3 | 1.245(4) | C10 | C9A | 1.468(11) |
| O4 | $Tb1^1$ | 2.424(2) | C11 | C12 | 1.488(6) |
| O5 | C7 | 1.458(4) | C13 | C14 | 1.476(7) |

| | | | | | |
|---|---|---|---|---|---|
| O6 | C9 | 1.444(7) | N1S | C1S | 1.129(6) |
| O6 | C9A | 1.331(10) | C1S | C2S | 1.443(6) |

[1]1/2-X,1/2-Y,1-Z

**Table 5 Bond Angles for TB(NO3)3-1.**

| Atom | Atom | Atom | Angle/° | Atom | Atom | Atom | Angle/° |
|---|---|---|---|---|---|---|---|
| O1 | Tb1 | O2 | 75.44(8) | O6 | P1 | C2 | 103.39(16) |
| O1 | Tb1 | O3[1] | 78.21(8) | O4 | P2 | O7 | 114.63(16) |
| O1 | Tb1 | O4[1] | 77.73(8) | O4 | P2 | O8 | 109.67(14) |
| O1 | Tb1 | O9 | 139.17(8) | O4 | P2 | C4 | 112.32(16) |
| O1 | Tb1 | O10 | 145.31(9) | O7 | P2 | C4 | 109.64(17) |
| O1 | Tb1 | O12 | 74.50(8) | O8 | P2 | O7 | 102.62(15) |
| O1 | Tb1 | O13 | 122.45(8) | O8 | P2 | C4 | 107.29(16) |
| O1 | Tb1 | O15 | 76.68(9) | C1 | O1 | Tb1 | 139.9(2) |
| O1 | Tb1 | O16 | 124.70(8) | P1 | O2 | Tb1 | 134.89(14) |
| O2 | Tb1 | O3[1] | 142.45(8) | C3 | O3 | Tb1[1] | 135.0(2) |
| O2 | Tb1 | O4[1] | 74.55(8) | P2 | O4 | Tb1[1] | 139.09(14) |
| O2 | Tb1 | O9 | 118.60(8) | C7 | O5 | P1 | 124.0(2) |
| O2 | Tb1 | O10 | 74.69(8) | C9 | O6 | P1 | 124.2(3) |
| O2 | Tb1 | O12 | 68.53(8) | C9A | O6 | P1 | 130.6(5) |
| O2 | Tb1 | O13 | 78.18(8) | C11 | O7 | P2 | 127.4(3) |
| O2 | Tb1 | O15 | 129.45(8) | C13 | O8 | P2 | 120.3(3) |
| O2 | Tb1 | O16 | 146.31(8) | N3 | O9 | Tb1 | 95.92(19) |
| O3[1] | Tb1 | O4[1] | 74.06(8) | N3 | O10 | Tb1 | 98.38(19) |
| O3[1] | Tb1 | O9 | 68.65(8) | N4 | O12 | Tb1 | 93.20(19) |
| O3[1] | Tb1 | O10 | 117.45(8) | N4 | O13 | Tb1 | 101.96(19) |
| O3[1] | Tb1 | O12 | 128.34(8) | N5 | O15 | Tb1 | 96.8(2) |
| O3[1] | Tb1 | O13 | 139.19(8) | N5 | O16 | Tb1 | 95.2(2) |
| O3[1] | Tb1 | O15 | 67.72(9) | C1 | N1 | C5 | 123.7(3) |
| O3[1] | Tb1 | O16 | 71.24(8) | C3 | N2 | C6 | 120.7(3) |
| O4[1] | Tb1 | O9 | 70.87(9) | O9 | N3 | O10 | 116.0(3) |
| O4[1] | Tb1 | O10 | 77.66(8) | O11 | N3 | O9 | 122.2(3) |
| O4[1] | Tb1 | O12 | 138.10(9) | O11 | N3 | O10 | 121.8(3) |
| O4[1] | Tb1 | O13 | 140.11(9) | O12 | N4 | O13 | 115.9(3) |
| O4[1] | Tb1 | O15 | 137.37(9) | O14 | N4 | O12 | 123.0(3) |
| O4[1] | Tb1 | O16 | 132.01(9) | O14 | N4 | O13 | 121.0(3) |
| O9 | Tb1 | O12 | 145.63(8) | O16 | N5 | O15 | 116.6(3) |
| O10 | Tb1 | O9 | 49.53(8) | O17 | N5 | O15 | 121.0(3) |
| O10 | Tb1 | O12 | 109.83(8) | O17 | N5 | O16 | 122.4(3) |
| O10 | Tb1 | O15 | 137.10(9) | O1 | C1 | N1 | 122.7(3) |
| O10 | Tb1 | O16 | 89.98(9) | O1 | C1 | C2 | 120.6(3) |
| O13 | Tb1 | O9 | 98.35(8) | N1 | C1 | C2 | 116.7(3) |
| O13 | Tb1 | O10 | 67.34(8) | C1 | C2 | P1 | 110.4(2) |
| O13 | Tb1 | O12 | 48.28(8) | O3 | C3 | N2 | 121.7(3) |
| O13 | Tb1 | O15 | 82.51(9) | O3 | C3 | C4 | 120.2(3) |
| O13 | Tb1 | O16 | 68.20(8) | N2 | C3 | C4 | 118.0(3) |
| O15 | Tb1 | O9 | 110.13(8) | C3 | C4 | P2 | 111.2(2) |
| O15 | Tb1 | O12 | 63.79(9) | N1 | C5 | C6 | 112.2(3) |

| | | | | | | | |
|---|---|---|---|---|---|---|---|
| O15 | Tb1 | O16 | 49.48(9) | N2 | C6 | C5 | 112.6(3) |
| O16 | Tb1 | O9 | 66.20(8) | O5 | C7 | C8 | 107.6(3) |
| O16 | Tb1 | O12 | 89.79(9) | O6 | C9 | C10 | 109.6(5) |
| O2 | P1 | O5 | 110.19(14) | O7 | C11 | C12 | 106.9(4) |
| O2 | P1 | O6 | 115.22(15) | O8 | C13 | C14 | 109.9(4) |
| O2 | P1 | C2 | 112.55(15) | O6 | C9A | C10 | 117.6(8) |
| O5 | P1 | O6 | 107.63(15) | N1S | C1S | C2S | 178.6(5) |
| O5 | P1 | C2 | 107.36(15) | | | | |

[1]1/2-X,1/2-Y,1-Z

**Table 6 Hydrogen Bonds for TB(NO3)3-1.**

| D | H | A | d(D-H)/Å | d(H-A)/Å | d(D-A)/Å | D-H-A/° |
|---|---|---|---|---|---|---|
| C2 | H2A | O12 | 0.99 | 2.43 | 3.075(4) | 122.4 |
| C4 | H4A | N1S | 0.99 | 2.37 | 3.277(5) | 152.3 |
| C4 | H4B | O15 | 0.99 | 2.55 | 3.204(5) | 123.3 |
| C2S | H2SB | O12 | 0.98 | 2.50 | 3.284(5) | 137.0 |
| N1 | H1 | O13[1] | 0.80(4) | 2.09(4) | 2.881(4) | 172(4) |
| N2 | H2 | N1S | 0.81(4) | 2.33(4) | 3.070(5) | 152(4) |

[1]+X,1-Y,-1/2+Z

**Table 7 Hydrogen Atom Coordinates (Å×10$^4$) and Isotropic Displacement Parameters (Å$^2$×10$^3$) for TB(NO3)3-1.**

| Atom | *x* | *y* | *z* | U(eq) |
|---|---|---|---|---|
| H2A | 3754 | 5562 | 5037 | 29 |
| H2B | 4009 | 4932 | 4538 | 29 |
| H4A | 1985 | 4829 | 3937 | 34 |
| H4B | 1998 | 3966 | 4668 | 34 |
| H5A | 3061 | 2307 | 4128 | 28 |
| H5B | 3217 | 2090 | 3344 | 28 |
| H6A | 2677 | 3417 | 2434 | 28 |
| H6B | 2435 | 2402 | 2634 | 28 |
| H7A | 4878 | 5845 | 5357 | 45 |
| H7B | 5262 | 5214 | 6207 | 45 |
| H8A | 5031 | 7401 | 6197 | 73 |
| H8B | 5510 | 6956 | 6265 | 73 |
| H8C | 5380 | 6786 | 7092 | 73 |
| H9A | 4704 | 2561 | 6816 | 46 |
| H9B | 5178 | 3124 | 6967 | 46 |
| H10A | 4687 | 1646 | 5558 | 83 |
| H10B | 5154 | 1382 | 6442 | 83 |
| H10C | 5159 | 2210 | 5705 | 83 |
| H10D | 5075 | 2115 | 5536 | 83 |
| H10E | 4893 | 1186 | 5965 | 83 |
| H10F | 5235 | 2080 | 6608 | 83 |
| H11A | 1397 | 4230 | 1733 | 52 |
| H11B | 1602 | 5311 | 2294 | 52 |

| | | | | |
|---|---|---|---|---|
| H12A | 1119 | 5735 | 781 | 77 |
| H12B | 895 | 6174 | 1412 | 77 |
| H12C | 703 | 5100 | 841 | 77 |
| H13A | 1296 | 5905 | 3918 | 54 |
| H13B | 1216 | 5710 | 4803 | 54 |
| H14A | 506 | 5936 | 3018 | 112 |
| H14B | 639 | 6806 | 3807 | 112 |
| H14C | 417 | 5692 | 3884 | 112 |
| H9AA | 4318 | 2386 | 5321 | 44 |
| H9AB | 4502 | 2438 | 6395 | 44 |
| H2SA | 3196 | 7880 | 4336 | 64 |
| H2SB | 3160 | 7562 | 5247 | 64 |
| H2SC | 2785 | 8342 | 4525 | 64 |
| H1 | 3531(13) | 3720(30) | 3630(30) | 18(10) |
| H2 | 2434(14) | 4360(30) | 3250(30) | 30(11) |

**Table 8 Atomic Occupancy for TB(NO3)3-1.**

| **Atom** | ***Occupancy*** | **Atom** | ***Occupancy*** | **Atom** | ***Occupancy*** |
|---|---|---|---|---|---|
| C9 | 0.610(12) | H9A | 0.610(12) | H9B | 0.610(12) |
| H10A | 0.610(12) | H10B | 0.610(12) | H10C | 0.610(12) |
| H10D | 0.390(12) | H10E | 0.390(12) | H10F | 0.390(12) |
| C9A | 0.390(12) | H9AA | 0.390(12) | H9AB | 0.390(12) |

**Crystal structure determination of [TB(NO3)3-1]**

**Crystal Data** for $C_{32}H_{66}N_{12}O_{34}P_4Tb_2$ ($M$ =1604.68 g/mol): monoclinic, space group C2/c (no. 15), $a$ = 32.3189(5) Å, $b$ = 12.5255(2) Å, $c$ = 16.4158(3) Å, $\beta$ = 116.1620(10)°, $V$ = 5964.48(18) Å$^3$, $Z$ = 4, $T$ = 173.02 K, μ(CuKα) = 13.430 mm$^{-1}$, $Dcalc$ = 1.787 g/cm$^3$, 44653 reflections measured (6.094° ≤ 2Θ ≤ 140.374°), 5582 unique ($R_{int}$ = 0.0588, $R_{sigma}$ = 0.0348) which were used in all calculations. The final $R_1$ was 0.0287 (I > 2σ(I)) and $wR_2$ was 0.0653 (all data).

**Refinement model description**

Number of restraints - 0, number of constraints - unknown.

Details:

1. Fixed Uiso

At 1.2 times of:

All C(H,H) groups

At 1.5 times of:

All C(H,H,H) groups, All C(H,H,H,H,H,H) groups

2. Others

Sof(H10D)=Sof(H10E)=Sof(H10F)=Sof(C9A)=Sof(H9AA)=Sof(H9AB)=1-FVAR(1)

Sof(C9)=Sof(H9A)=Sof(H9B)=Sof(H10A)=Sof(H10B)=Sof(H10C)=FVAR(1)

3.a Secondary CH2 refined with riding coordinates:

C2(H2A,H2B), C4(H4A,H4B), C5(H5A,H5B), C6(H6A,H6B), C7(H7A,H7B), C9(H9A,H9B), C11(H11A,H11B), C13(H13A,H13B), C9A(H9AA,H9AB)

3.b Idealised Me refined as rotating group:

C8(H8A,H8B,H8C), C10(H10A,H10B,H10C), C10(H10D,H10E,H10F), C12(H12A,H12B, H12C), C14(H14A,H14B,H14C), C2S(H2SA,H2SB,H2SC)

**E. $Sm(NO_3)_3$-1**

The figure on the left depicts the atom numbering scheme of the 2:2 **$Sm(NO_3)_3$-1** complex using a ball and stick model with standard CPK colors (Sm atoms are colored purple). The electron density corresponding to the methylene carbon C9 was disordered, and modeled over two positions. The occupancy of this atom was refined against a free variable to give a ratio of 62.9:37.1. The N1-H1 bond was restrained using a DFIX in ShelXL command to give a bond length that agrees with known values. Hydrogen atoms bonded to carbon atoms are not shown for clarity, and hydrogen bonds are depicted with green, dashed lines. The figure on the right depicts the thermal ellipsoids of all atoms of the 2:2 complex, which are shown at the 40% probability level.

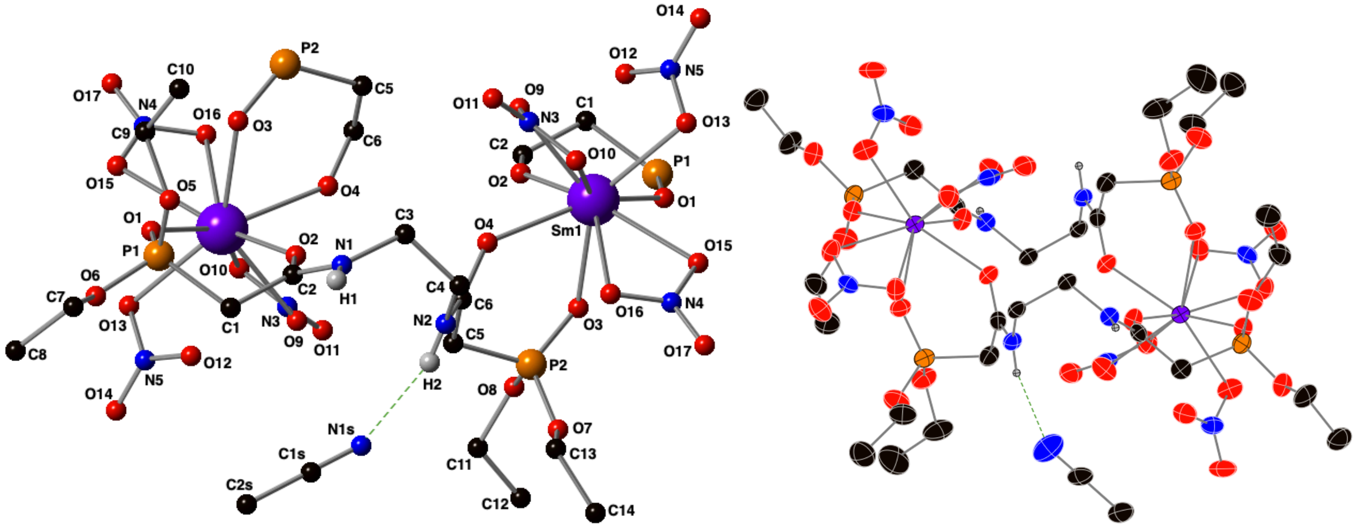


## Experimental

Single crystals of $C_{32}H_{66}N_{12}O_{34}P_4Sm_2$ **[SM(NO3)3-1]** were grown by slow diffusion of diethyl ether into an acetonitrile solution of the complex. A suitable crystal was selected and mounted using a small amount of paratone oil on a Bruker APEX-II CCD diffractometer. The crystal was kept at 173(2) K during data collection. Using Olex2 [1], the structure was solved with the ShelXT [2] structure solution program using Intrinsic Phasing and refined with the ShelXL [3] refinement package using Least Squares minimization.

1. Dolomanov, O.V., Bourhis, L.J., Gildea, R.J, Howard, J.A.K. & Puschmann, H. (2009), J. Appl. Cryst. 42, 339-341.
2. Sheldrick, G.M. (2015). Acta Cryst. A71, 3-8.
3. Sheldrick, G.M. (2015). Acta Cryst. C71, 3-8.

## Crystal structure determination of [SM(NO3)3-1]

**Crystal Data** for $C_{32}H_{66}N_{12}O_{34}P_4Sm_2$ ($M$ =1587.54 g/mol): monoclinic, space group C2/c (no. 15), $a$ = 32.318(11) Å, $b$ = 12.538(4) Å, $c$ = 16.447(6) Å, $\beta$ = 116.217(4)°, $V$ = 5979(4) Å$^3$, $Z$ = 4, $T$ = 173(2) K, μ(MoK$\alpha$) = 2.154 mm$^{-1}$, $Dcalc$ = 1.764 g/cm$^3$, 26038 reflections measured (3.54° ≤ 2Θ ≤ 52.802°), 6120 unique ($R_{int}$ = 0.0770, $R_{sigma}$ = 0.0704) which were used in all calculations. The final $R_1$ was 0.0532 (I > 2$\sigma$(I)) and $wR_2$ was 0.1487 (all data).

**Table 1 Crystal data and structure refinement for SM(NO3)3-1.**

| | |
|---|---|
| CCDC code | 2003370 |
| Empirical formula | $C_{32}H_{66}N_{12}O_{34}P_4Sm_2$ |
| Formula weight | 1587.54 |
| Temperature/K | 173(2) |

| | |
|---|---|
| Crystal system | monoclinic |
| Space group | C2/c |
| a/Å | 32.318(11) |
| b/Å | 12.538(4) |
| c/Å | 16.447(6) |
| $\alpha$/° | 90 |
| β/° | 116.217(4) |
| $\gamma$/° | 90 |
| Volume/Å$^3$ | 5979(4) |
| Z | 4 |
| $\rho_{calc}$g/cm$^3$ | 1.764 |
| μ/mm$^{-1}$ | 2.154 |
| F(000) | 3192.0 |
| Crystal size/mm$^3$ | 0.279 × 0.218 × 0.082 |
| Radiation | MoK$\alpha$ ($\lambda$ = 0.71073) |
| 2Θ range for data collection/° | 3.54 to 52.802 |
| Index ranges | -40 ≤ h ≤ 40, -15 ≤ k ≤ 15, -20 ≤ l ≤ 20 |
| Reflections collected | 26038 |
| Independent reflections | 6120 [$R_{int}$ = 0.0770, $R_{sigma}$ = 0.0704] |
| Data/restraints/parameters | 6120/1/402 |
| Goodness-of-fit on F$^2$ | 1.058 |
| Final R indexes [I>=2σ (I)] | $R_1$ = 0.0532, w$R_2$ = 0.1296 |
| Final R indexes [all data] | $R_1$ = 0.0776, w$R_2$ = 0.1487 |
| Largest diff. peak/hole / e Å$^{-3}$ | 3.54/-0.79 |

**Table 2 Fractional Atomic Coordinates (×10$^4$) and Equivalent Isotropic Displacement Parameters (Å$^2$×10$^3$) for SM(NO3)3-1. $U_{eq}$ is defined as 1/3 of of the trace of the 34rthogonalized $U_{IJ}$ tensor.**

| Atom | *x* | *y* | *z* | U(eq) |
|---|---|---|---|---|
| Sm1 | 3493.7(2) | 6067.2(2) | 6793.8(2) | 26.46(13) |
| P1 | 4370.3(6) | 5416.9(14) | 6087.5(12) | 32.6(4) |
| P2 | 1288.1(6) | 6079.8(12) | 3387.0(13) | 33.1(4) |
| O1 | 4219.4(15) | 5659(4) | 6795(3) | 34.2(10) |
| O2 | 3353.9(16) | 6242(3) | 5248(3) | 33.5(11) |
| O3 | 1157.7(15) | 7211(3) | 3204(3) | 38.0(11) |
| O4 | 2128.7(15) | 7651(3) | 3774(3) | 36.1(10) |
| O5 | 4626.9(18) | 6338(4) | 5873(3) | 46.4(13) |
| O6 | 4692.7(16) | 4429(4) | 6367(3) | 38.7(11) |
| O7 | 1104.8(18) | 5343(4) | 2543(3) | 50.0(13) |
| O8 | 1071.7(17) | 5581(4) | 3982(3) | 46.2(13) |
| O9 | 2706.5(18) | 5181(4) | 5924(3) | 51.0(13) |
| O10 | 2882.1(17) | 5486(4) | 7308(3) | 45.3(12) |

| | | | | |
|---|---|---|---|---|
| O11 | 2170.7(17) | 5111(4) | 6370(4) | 51.7(14) |
| O12 | 3504.9(19) | 4050(4) | 6209(3) | 45.3(13) |
| O13 | 3694.6(18) | 4297(4) | 7623(3) | 41.7(12) |
| O14 | 3593.9(19) | 2701(4) | 7091(4) | 54.8(14) |
| O15 | 4088.9(16) | 6248(4) | 8440(3) | 39.0(11) |
| O16 | 3487.0(16) | 7236(4) | 8092(3) | 41.8(12) |
| O17 | 4061.9(17) | 7346(4) | 9429(3) | 49.1(13) |
| N1 | 3438.7(18) | 6505(4) | 3976(3) | 29.6(12) |
| N2 | 2409.2(19) | 6265(5) | 3297(4) | 31.8(13) |
| N3 | 2575(2) | 5262(4) | 6537(5) | 40.0(14) |
| N4 | 3884.1(19) | 6961(4) | 8673(3) | 32.6(12) |
| N5 | 3595(2) | 3649(5) | 6958(4) | 36.6(14) |
| C1 | 3897(2) | 5131(5) | 5011(4) | 34.4(15) |
| C2 | 3535(2) | 6012(5) | 4744(4) | 27.8(14) |
| C3 | 3100(2) | 7363(5) | 3620(4) | 30.5(14) |
| C4 | 2637(2) | 6966(5) | 2920(4) | 30.4(14) |
| C5 | 1900(2) | 5919(5) | 4000(5) | 36.9(16) |
| C6 | 2167(2) | 6676(5) | 3693(4) | 30.6(14) |
| C7 | 5038(3) | 4188(6) | 6042(6) | 49(2) |
| C8 | 5253(3) | 3171(6) | 6408(6) | 61(2) |
| C9 | 4894(4) | 7158(10) | 6509(9) | 47(4) |
| C10 | 4979(3) | 8057(6) | 6010(6) | 61(2) |
| C11 | 1087(3) | 4445(6) | 4135(6) | 61(2) |
| C12 | 626(4) | 3967(7) | 3667(9) | 91(4) |
| C13 | 1320(3) | 5111(6) | 1956(5) | 51(2) |
| C14 | 974(3) | 4454(7) | 1175(6) | 66(2) |
| C9A | 4576(7) | 7409(14) | 5892(15) | 41(6) |
| N1S | 2480(3) | 3888(6) | 3780(6) | 70(2) |
| C1S | 2704(3) | 3185(6) | 4140(5) | 45.1(18) |
| C2S | 2985(3) | 2286(6) | 4603(5) | 54(2) |

**Table 3 Anisotropic Displacement Parameters (Å$^2$×10$^3$) for SM(NO3)3-1. The Anisotropic displacement factor exponent takes the form: $-2\pi^2[h^2a^{*2}U_{11}+2hka^*b^*U_{12}+\ldots]$.**

| **Atom** | **$U_{11}$** | **$U_{22}$** | **$U_{33}$** | **$U_{23}$** | **$U_{13}$** | **$U_{12}$** |
|---|---|---|---|---|---|---|
| Sm1 | 29.8(2) | 26.4(2) | 26.9(2) | 1.48(13) | 15.88(16) | 1.64(13) |
| P1 | 30.5(9) | 36.6(9) | 33.4(9) | 3.6(7) | 16.5(8) | 4.3(8) |
| P2 | 34.6(10) | 29.3(9) | 40.8(10) | 2.3(7) | 21.5(8) | 1.4(7) |
| O1 | 35(3) | 41(3) | 29(2) | 1(2) | 17(2) | 5(2) |
| O2 | 35(3) | 43(3) | 31(2) | 5(2) | 22(2) | 10(2) |
| O3 | 35(3) | 31(2) | 53(3) | 3(2) | 23(2) | -1(2) |
| O4 | 39(3) | 31(3) | 38(3) | 4(2) | 18(2) | 9(2) |
| O5 | 56(3) | 46(3) | 50(3) | 2(2) | 34(3) | -8(3) |

| | | | | | | |
|---|---|---|---|---|---|---|
| O6 | 38(3) | 47(3) | 40(3) | 7(2) | 25(2) | 9(2) |
| O7 | 49(3) | 55(3) | 55(3) | -12(3) | 31(3) | -11(3) |
| O8 | 49(3) | 40(3) | 62(3) | 13(2) | 37(3) | 6(2) |
| O9 | 51(3) | 55(3) | 54(3) | -16(3) | 28(3) | -13(3) |
| O10 | 42(3) | 48(3) | 47(3) | 5(2) | 19(3) | -3(2) |
| O11 | 33(3) | 41(3) | 83(4) | -3(3) | 28(3) | -5(2) |
| O12 | 59(3) | 43(3) | 34(3) | -1(2) | 21(3) | -8(2) |
| O13 | 60(3) | 40(3) | 41(3) | 6(2) | 37(3) | 5(2) |
| O14 | 78(4) | 28(3) | 59(3) | -5(2) | 31(3) | -5(3) |
| O15 | 41(3) | 44(3) | 31(3) | 1(2) | 15(2) | 12(2) |
| O16 | 34(3) | 51(3) | 36(3) | -5(2) | 12(2) | 4(2) |
| O17 | 48(3) | 63(3) | 36(3) | -14(2) | 18(3) | -5(3) |
| N1 | 34(3) | 33(3) | 28(3) | -1(2) | 20(3) | 1(2) |
| N2 | 28(3) | 31(3) | 40(3) | 0(3) | 18(3) | 4(2) |
| N3 | 39(4) | 26(3) | 64(4) | 2(3) | 31(3) | 1(3) |
| N4 | 39(3) | 35(3) | 27(3) | -5(2) | 17(3) | -8(3) |
| N5 | 43(4) | 30(3) | 46(4) | -1(3) | 28(3) | -3(3) |
| C1 | 43(4) | 39(4) | 28(3) | -1(3) | 22(3) | 3(3) |
| C2 | 29(3) | 33(3) | 22(3) | -2(3) | 11(3) | -4(3) |
| C3 | 34(4) | 27(3) | 35(4) | 3(3) | 19(3) | 5(3) |
| C4 | 35(4) | 28(3) | 29(3) | 1(3) | 15(3) | 8(3) |
| C5 | 34(4) | 36(4) | 45(4) | 10(3) | 21(3) | 4(3) |
| C6 | 26(3) | 34(4) | 29(3) | 7(3) | 10(3) | 1(3) |
| C7 | 36(4) | 51(4) | 72(6) | 6(4) | 35(4) | 14(4) |
| C8 | 43(5) | 46(5) | 97(7) | 4(5) | 32(5) | 9(4) |
| C9 | 38(8) | 50(8) | 56(9) | -5(6) | 23(7) | -7(6) |
| C10 | 58(5) | 46(5) | 78(6) | 10(4) | 28(5) | -7(4) |
| C11 | 71(6) | 41(5) | 88(7) | 21(4) | 52(6) | 8(4) |
| C12 | 82(8) | 72(7) | 121(10) | 11(6) | 45(7) | -30(6) |
| C13 | 66(6) | 44(4) | 53(5) | -9(4) | 36(4) | -6(4) |
| C14 | 76(7) | 64(6) | 48(5) | -8(4) | 20(5) | -1(5) |
| C9A | 46(14) | 36(11) | 47(13) | 29(9) | 25(12) | 16(9) |
| N1S | 93(6) | 64(5) | 72(5) | 14(4) | 54(5) | 25(4) |
| C1S | 69(5) | 33(4) | 38(4) | -8(3) | 28(4) | 4(4) |
| C2S | 67(6) | 37(4) | 58(5) | -1(4) | 29(5) | 5(4) |

**Table 4 Bond Lengths for SM(NO3)3-1.**

| Atom | Atom | Length/Å | Atom | Atom | Length/Å |
|---|---|---|---|---|---|
| Sm1 | O1 | 2.400(4) | O7 | C13 | 1.447(8) |
| Sm1 | O2 | 2.387(4) | O8 | C11 | 1.444(9) |
| Sm1 | O3[1] | 2.434(4) | O9 | N3 | 1.260(7) |
| Sm1 | O4[1] | 2.417(4) | O10 | N3 | 1.249(7) |

| | | | | | |
|---|---|---|---|---|---|
| Sm1 | O9 | 2.557(5) | O11 | N3 | 1.225(7) |
| Sm1 | O10 | 2.574(5) | O12 | N5 | 1.239(7) |
| Sm1 | O12 | 2.712(5) | O13 | N5 | 1.284(7) |
| Sm1 | O13 | 2.535(5) | O14 | N5 | 1.209(7) |
| Sm1 | O15 | 2.546(5) | O15 | N4 | 1.268(7) |
| Sm1 | O16 | 2.598(5) | O16 | N4 | 1.263(7) |
| Sm1 | N3 | 2.984(6) | O17 | N4 | 1.216(6) |
| Sm1 | N4 | 2.992(5) | N1 | C2 | 1.314(8) |
| P1 | O1 | 1.478(4) | N1 | C3 | 1.460(8) |
| P1 | O5 | 1.551(5) | N2 | C4 | 1.449(8) |
| P1 | O6 | 1.552(5) | N2 | C6 | 1.323(8) |
| P1 | C1 | 1.792(7) | C1 | C2 | 1.525(9) |
| P2 | O3 | 1.473(4) | C3 | C4 | 1.514(8) |
| P2 | O7 | 1.551(5) | C5 | C6 | 1.511(9) |
| P2 | O8 | 1.563(5) | C7 | C8 | 1.450(10) |
| P2 | C5 | 1.792(7) | C9 | C10 | 1.489(14) |
| O2 | C2 | 1.242(7) | C10 | C9A | 1.47(2) |
| O4 | C6 | 1.242(7) | C11 | C12 | 1.468(12) |
| O5 | C9 | 1.448(12) | C13 | C14 | 1.519(11) |
| O5 | C9A | 1.355(18) | N1S | C1S | 1.128(9) |
| O6 | C7 | 1.465(8) | C1S | C2S | 1.437(10) |

[1]1/2-X,3/2-Y,1-Z

**Table 5 Bond Angles for SM(NO3)3-1.**

| Atom | Atom | Atom | Angle/° | Atom | Atom | Atom | Angle/° |
|---|---|---|---|---|---|---|---|
| O1 | Sm1 | O3[1] | 74.78(15) | O16 | Sm1 | N3 | 86.45(16) |
| O1 | Sm1 | O4[1] | 142.01(15) | O16 | Sm1 | N4 | 24.85(14) |
| O1 | Sm1 | O9 | 129.64(16) | N3 | Sm1 | N4 | 102.25(16) |
| O1 | Sm1 | O10 | 146.23(16) | O1 | P1 | O5 | 114.9(3) |
| O1 | Sm1 | O12 | 68.60(16) | O1 | P1 | O6 | 110.0(3) |
| O1 | Sm1 | O13 | 78.17(15) | O1 | P1 | C1 | 112.6(3) |
| O1 | Sm1 | O15 | 74.89(15) | O5 | P1 | O6 | 107.8(3) |
| O1 | Sm1 | O16 | 118.88(14) | O5 | P1 | C1 | 103.5(3) |
| O1 | Sm1 | N3 | 147.02(16) | O6 | P1 | C1 | 107.6(3) |
| O1 | Sm1 | N4 | 96.50(15) | O3 | P2 | O7 | 115.4(3) |
| O2 | Sm1 | O1 | 75.24(14) | O3 | P2 | O8 | 110.3(3) |
| O2 | Sm1 | O3[1] | 77.88(15) | O3 | P2 | C5 | 111.6(3) |
| O2 | Sm1 | O4[1] | 78.09(14) | O7 | P2 | O8 | 102.9(3) |
| O2 | Sm1 | O9 | 76.73(16) | O7 | P2 | C5 | 109.1(3) |
| O2 | Sm1 | O10 | 124.33(16) | O8 | P2 | C5 | 106.9(3) |
| O2 | Sm1 | O12 | 74.57(15) | P1 | O1 | Sm1 | 134.9(3) |
| O2 | Sm1 | O13 | 122.43(14) | C2 | O2 | Sm1 | 140.0(4) |

| | | | | | | | |
|---|---|---|---|---|---|---|---|
| O2 | Sm1 | O15 | 145.37(16) | P2 | O3 | Sm1$^1$ | 139.8(3) |
| O2 | Sm1 | O16 | 139.22(15) | C6 | O4 | Sm1$^1$ | 135.9(4) |
| O2 | Sm1 | N3 | 99.90(17) | C9 | O5 | P1 | 124.9(6) |
| O2 | Sm1 | N4 | 149.37(15) | C9A | O5 | P1 | 130.6(9) |
| O3$^1$ | Sm1 | O9 | 137.03(16) | C7 | O6 | P1 | 125.1(4) |
| O3$^1$ | Sm1 | O10 | 132.27(15) | C13 | O7 | P2 | 127.7(5) |
| O3$^1$ | Sm1 | O12 | 138.50(15) | C11 | O8 | P2 | 120.9(5) |
| O3$^1$ | Sm1 | O13 | 140.10(16) | N3 | O9 | Sm1 | 97.0(4) |
| O3$^1$ | Sm1 | O15 | 77.73(15) | N3 | O10 | Sm1 | 96.4(4) |
| O3$^1$ | Sm1 | O16 | 70.76(15) | N5 | O12 | Sm1 | 93.4(4) |
| O3$^1$ | Sm1 | N3 | 136.99(15) | N5 | O13 | Sm1 | 100.8(4) |
| O3$^1$ | Sm1 | N4 | 71.49(15) | N4 | O15 | Sm1 | 97.7(3) |
| O4$^1$ | Sm1 | O3$^1$ | 73.53(15) | N4 | O16 | Sm1 | 95.3(3) |
| O4$^1$ | Sm1 | O9 | 67.69(16) | C2 | N1 | C3 | 123.4(5) |
| O4$^1$ | Sm1 | O10 | 71.75(15) | C6 | N2 | C4 | 119.8(6) |
| O4$^1$ | Sm1 | O12 | 128.50(16) | O9 | N3 | Sm1 | 58.3(3) |
| O4$^1$ | Sm1 | O13 | 139.64(15) | O10 | N3 | Sm1 | 59.0(3) |
| O4$^1$ | Sm1 | O15 | 117.43(14) | O10 | N3 | O9 | 115.6(6) |
| O4$^1$ | Sm1 | O16 | 68.59(15) | O11 | N3 | Sm1 | 168.5(4) |
| O4$^1$ | Sm1 | N3 | 64.17(15) | O11 | N3 | O9 | 120.8(7) |
| O4$^1$ | Sm1 | N4 | 92.83(15) | O11 | N3 | O10 | 123.6(6) |
| O9 | Sm1 | O10 | 48.88(16) | O15 | N4 | Sm1 | 57.5(3) |
| O9 | Sm1 | O12 | 64.03(16) | O16 | N4 | Sm1 | 59.8(3) |
| O9 | Sm1 | O16 | 109.74(15) | O16 | N4 | O15 | 117.1(5) |
| O9 | Sm1 | N3 | 24.78(15) | O17 | N4 | Sm1 | 176.5(4) |
| O9 | Sm1 | N4 | 127.03(15) | O17 | N4 | O15 | 121.0(6) |
| O10 | Sm1 | O12 | 89.12(16) | O17 | N4 | O16 | 121.8(5) |
| O10 | Sm1 | O16 | 66.64(16) | O12 | N5 | O13 | 116.8(6) |
| O10 | Sm1 | N3 | 24.57(15) | O14 | N5 | O12 | 124.1(6) |
| O10 | Sm1 | N4 | 78.56(15) | O14 | N5 | O13 | 119.1(6) |
| O12 | Sm1 | N3 | 78.61(16) | C2 | C1 | P1 | 110.6(4) |
| O12 | Sm1 | N4 | 130.53(15) | O2 | C2 | N1 | 123.4(6) |
| O13 | Sm1 | O9 | 82.86(18) | O2 | C2 | C1 | 120.2(5) |
| O13 | Sm1 | O10 | 68.09(15) | N1 | C2 | C1 | 116.3(6) |
| O13 | Sm1 | O12 | 48.22(16) | N1 | C3 | C4 | 112.1(5) |
| O13 | Sm1 | O15 | 67.18(15) | N2 | C4 | C3 | 113.1(5) |
| O13 | Sm1 | O16 | 98.32(15) | C6 | C5 | P2 | 113.1(4) |
| O13 | Sm1 | N3 | 77.43(16) | O4 | C6 | N2 | 123.0(6) |
| O13 | Sm1 | N4 | 83.18(15) | O4 | C6 | C5 | 118.9(6) |
| O15 | Sm1 | O9 | 137.02(16) | N2 | C6 | C5 | 117.9(6) |
| O15 | Sm1 | O10 | 90.30(16) | C8 | C7 | O6 | 109.5(6) |
| O15 | Sm1 | O12 | 109.75(15) | O5 | C9 | C10 | 109.6(10) |
| O15 | Sm1 | O16 | 49.63(14) | O8 | C11 | C12 | 111.3(7) |
| O15 | Sm1 | N3 | 114.70(17) | O7 | C13 | C14 | 106.5(7) |

| | | | | | | | |
|---|---|---|---|---|---|---|---|
| O15 | Sm1 | N4 | 24.83(14) | O5 | C9A | C10 | 116.2(14) |
| O16 | Sm1 | O12 | 145.46(15) | N1S | C1S | C2S | 179.4(9) |

[1]1/2-X,3/2-Y,1-Z

**Table 6 Hydrogen Bonds for SM(NO3)3-1.**

| D | H | A | d(D-H)/Å | d(H-A)/Å | d(D-A)/Å | D-H-A/° |
|---|---|---|---|---|---|---|
| N1 | H1 | O13[1] | 0.877(10) | 2.03(2) | 2.877(7) | 161(6) |
| N2 | H2 | N1S | 0.85(10) | 2.24(10) | 3.067(9) | 163(9) |

[1]+X,1-Y,-1/2+Z

**Table 7 Hydrogen Atom Coordinates (Å×10[4]) and Isotropic Displacement Parameters (Å[2]×10[3]) for SM(NO3)3-1.**

| Atom | *x* | *y* | *z* | U(eq) |
|---|---|---|---|---|
| H1 | 3580(19) | 6300(50) | 3650(40) | 30(18) |
| H2 | 2390(30) | 5590(80) | 3310(70) | 100(40) |
| H1A | 4010.68 | 5068.88 | 4544.12 | 41 |
| H1B | 3755.57 | 4441.7 | 5042.96 | 41 |
| H3A | 3216.68 | 7913.58 | 3341.24 | 37 |
| H3B | 3060.79 | 7700.63 | 4125.33 | 37 |
| H4A | 2434.54 | 7586.16 | 2635.43 | 37 |
| H4B | 2680.82 | 6579.61 | 2438.81 | 37 |
| H5A | 1993.77 | 6035.26 | 4652.94 | 44 |
| H5B | 1982.26 | 5176.94 | 3923.81 | 44 |
| H7A | 4888.09 | 4159.52 | 5371.52 | 59 |
| H7B | 5274.46 | 4756.94 | 6236.14 | 59 |
| H8A | 5479.72 | 3003.69 | 6180.79 | 92 |
| H8B | 5017.1 | 2612.03 | 6218.02 | 92 |
| H8C | 5408.32 | 3210.08 | 7070.74 | 92 |
| H9A | 5191.63 | 6858.09 | 6952.66 | 56 |
| H9B | 4724.07 | 7420.11 | 6843.87 | 56 |
| H10A | 4690.72 | 8447.77 | 5669.08 | 92 |
| H10B | 5092.49 | 7776.02 | 5590.16 | 92 |
| H10C | 5209.67 | 8539.84 | 6443.57 | 92 |
| H10D | 4948.71 | 8281.34 | 5415.63 | 92 |
| H10E | 5260.57 | 7633.42 | 6319.26 | 92 |
| H10F | 4995.61 | 8688.6 | 6374.79 | 92 |
| H11A | 1212.9 | 4304.11 | 4793.1 | 73 |
| H11B | 1295.44 | 4109.21 | 3913.26 | 73 |
| H12A | 488.74 | 4164.17 | 3024.83 | 137 |
| H12B | 430.75 | 4228.82 | 3940.89 | 137 |

| | | | | |
|---|---|---|---|---|
| H12C | 651.61 | 3188.87 | 3724.36 | 137 |
| H13A | 1608.61 | 4703.97 | 2287.86 | 61 |
| H13B | 1393.47 | 5780.03 | 1727.73 | 61 |
| H14A | 867.77 | 3854.43 | 1414.49 | 98 |
| H14B | 1121.85 | 4182.54 | 808.25 | 98 |
| H14C | 710.62 | 4902.88 | 797.26 | 98 |
| H9AA | 4495.01 | 7580.3 | 6391.05 | 49 |
| H9AB | 4312.42 | 7626.22 | 5318.13 | 49 |
| H2SA | 3208.07 | 2144.49 | 4362.68 | 80 |
| H2SB | 2788.43 | 1657.56 | 4510.03 | 80 |
| H2SC | 3149.62 | 2441.64 | 5252.61 | 80 |

**Table 8 Atomic Occupancy for SM(NO3)3-1.**

| **Atom** | *Occupancy* | **Atom** | *Occupancy* | **Atom** | *Occupancy* |
|---|---|---|---|---|---|
| C9 | 0.629(19) | H9A | 0.629(19) | H9B | 0.629(19) |
| H10A | 0.5 | H10B | 0.5 | H10C | 0.5 |
| H10D | 0.5 | H10E | 0.5 | H10F | 0.5 |
| C9A | 0.371(19) | H9AA | 0.371(19) | H9AB | 0.371(19) |

**Refinement model description**

Number of restraints – 1, number of constraints – unknown.

Details:

1. Fixed Uiso
 At 1.2 times of:
  All C(H,H) groups
 At 1.5 times of:
  All C(H,H,H) groups, All C(H,H,H,H,H,H) groups

2. Restrained distances
 N1-H1
 0.88 with sigma of 0.01

3. Others
 Sof(C9A)=Sof(H9AA)=Sof(H9AB)=1-FVAR(1)
 Sof(C9)=Sof(H9A)=Sof(H9B)=FVAR(1)
 Fixed Sof: H10A(0.5) H10B(0.5) H10C(0.5) H10D(0.5) H10E(0.5) H10F(0.5)

4.a Secondary CH2 refined with riding coordinates:
 C1(H1A,H1B), C3(H3A,H3B), C4(H4A,H4B), C5(H5A,H5B), C7(H7A,H7B), C9(H9A,H9B), C11(H11A,H11B), C13(H13A,H13B), C9A(H9AA,H9AB)

4.b Idealised Me refined as rotating group:
 C8(H8A,H8B,H8C), C10(H10A,H10B,H10C), C10(H10D,H10E,H10F), C12(H12A,H12B, H12C), C14(H14A,H14B,H14C), C2S(H2SA,H2SB,H2SC)

**F. $Tb(NO_3)_3$-2 (water solvate)**

The figure on the left depicts the atom numbering scheme of the 2:2 **$Tb(NO_3)_3$-2 • (3 $H_2O$)** complex using a ball and stick model with standard CPK colors (Tb atoms are colored green). All phenyl rings and hydrogen atoms bonded to carbon atoms are not shown for clarity. Only one complete ligand (except for the pendant phenyl rings) is shown, although every atom in the inner coordination sphere of both metals has been included. The figure on the right depicts the thermal ellipsoids of all atoms of the 2:2 complex, which are shown at the 40% probability level. Hydrogen bonds are depicted with green, dashed lines.

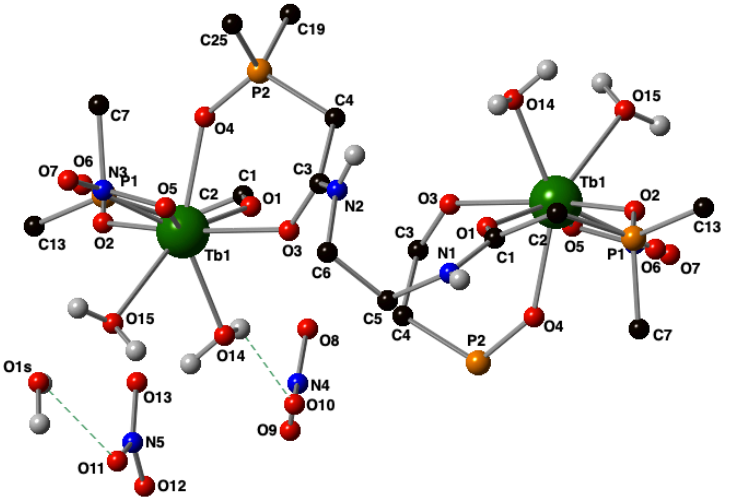

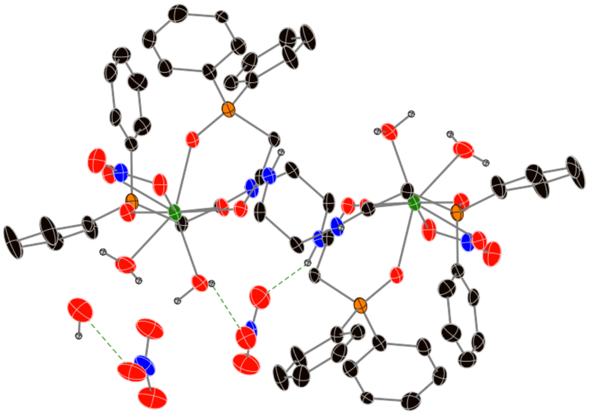

**Figure showing only atoms of inner coordination sphere:**

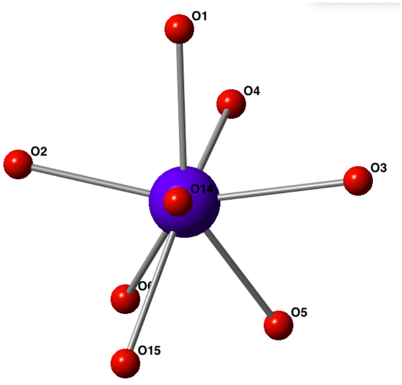


## Experimental

Single crystals of $C_{60}H_{72}N_{10}O_{32}P_4Tb_2$ **[$Tb(NO_3)_3$-2 (water solvate)]** were grown by slow evaporation of an acetonitrile solution of the complex. A suitable crystal was selected and mounted on a nylon loop using a small amount of paratone oil on a Bruker APEX-II CCD diffractometer. The crystal was kept at 173(2) K during data collection. Using Olex2 [1], the structure was solved with the ShelXT [2] structure solution program using Intrinsic Phasing and refined with the ShelXL [3] refinement package using Least Squares minimization.

1. Dolomanov, O.V., Bourhis, L.J., Gildea, R.J, Howard, J.A.K. & Puschmann, H. (2009), J. Appl. Cryst. 42, 339-341.
2. Sheldrick, G.M. (2015). Acta Cryst. A71, 3-8.
3. Sheldrick, G.M. (2015). Acta Cryst. C71, 3-8.

**Crystal structure determination of $Tb(NO_3)_3$-2 (water solvate)**

**Crystal Data** for $C_{60}H_{72}N_{10}O_{32}P_4Tb_2$ (*M* =1886.99 g/mol): triclinic, space group P-1 (no. 2), *a* = 11.438(2) Å, *b* = 12.724(2) Å, *c* = 14.681(3) Å, *α* = 91.784(2)°, *β* = 106.593(2)°, *γ* = 113.139(2)°, *V* = 1857.8(6) Å$^3$, *Z* = 1, *T* = 173(2) K, μ(MoK*α*) = 2.068 mm$^{-1}$, *Dcalc* = 1.687 g/cm$^3$, 18678 reflections measured (3.528° ≤ 2Θ ≤ 50.95°), 6825 unique ($R_{int}$ = 0.0585, $R_{sigma}$ = 0.0735) which were used in all calculations. The final $R_1$ was 0.0626 (I > 2σ(I)) and $wR_2$ was 0.1717 (all data).

**Table 1 Crystal data and structure refinement for $Tb(NO_3)_3$-2 (water solvate).**

| | |
|---|---|
| CCDC code | 2003374 |
| Empirical formula | $C_{60}H_{72}N_{10}O_{32}P_4Tb_2$ |
| Formula weight | 1886.99 |
| Temperature/K | 173(2) |
| Crystal system | triclinic |
| Space group | P-1 |
| a/Å | 11.438(2) |
| b/Å | 12.724(2) |
| c/Å | 14.681(3) |
| α/° | 91.784(2) |
| β/° | 106.593(2) |
| γ/° | 113.139(2) |
| Volume/Å$^3$ | 1857.8(6) |
| Z | 1 |
| $\rho_{calc}$g/cm$^3$ | 1.687 |
| μ/mm$^{-1}$ | 2.068 |
| F(000) | 948.0 |
| Crystal size/mm$^3$ | 0.187 × 0.11 × 0.038 |
| Radiation | MoK*α* (*λ* = 0.71073) |
| 2Θ range for data collection/° | 3.528 to 50.95 |
| Index ranges | -13 ≤ h ≤ 13, -12 ≤ k ≤ 15, -17 ≤ l ≤ 16 |
| Reflections collected | 18678 |
| Independent reflections | 6825 [$R_{int}$ = 0.0585, $R_{sigma}$ = 0.0735] |
| Data/restraints/parameters | 6825/0/492 |
| Goodness-of-fit on F$^2$ | 1.024 |
| Final R indexes [I>=2σ (I)] | $R_1$ = 0.0626, $wR_2$ = 0.1578 |
| Final R indexes [all data] | $R_1$ = 0.0791, $wR_2$ = 0.1717 |
| Largest diff. peak/hole / e Å$^{-3}$ | 3.52/-1.60 |

**Table 2 Fractional Atomic Coordinates (×10$^4$) and Equivalent Isotropic Displacement Parameters (Å$^2$×10$^3$) for TB(NO3)3-2 (WATER SOLVATE). $U_{eq}$ is defined as 1/3 of of the trace of the orthogonalized $U_{IJ}$ tensor.**

| Atom | *x* | *y* | *z* | U(eq) |
|---|---|---|---|---|
| Tb1 | 6061.6(3) | 1484.1(3) | 3059.3(3) | 26.32(16) |
| P1 | 4885.7(19) | 3581.4(16) | 2350.5(14) | 25.8(4) |
| P2 | 7121.3(18) | 942.7(16) | 7505.1(14) | 24.7(4) |
| O4 | 6159(5) | -199(4) | 7663(3) | 26.7(11) |

| | | | | |
|---|---|---|---|---|
| O1 | 4765(5) | 1973(5) | 3897(4) | 30.5(12) |
| O2 | 5786(5) | 2976(4) | 2353(4) | 32.5(12) |
| O3 | 4135(5) | -65(5) | 5934(4) | 30.6(12) |
| O6 | 5911(5) | 1047(5) | 1380(4) | 34.6(12) |
| N4 | 7303(6) | 4569(5) | 6193(5) | 26.0(13) |
| O14 | 7569(5) | 2715(5) | 4513(4) | 42.4(14) |
| O7 | 6482(7) | -242(6) | 867(5) | 52.3(17) |
| O5 | 6335(6) | -118(5) | 2307(4) | 39.8(14) |
| N1 | 3219(6) | 2474(6) | 4216(4) | 29.7(14) |
| N2 | 4568(6) | 1827(5) | 6013(4) | 27.6(13) |
| N3 | 6251(6) | 213(6) | 1483(5) | 31.9(15) |
| O10 | 8039(7) | 4075(6) | 6156(5) | 56.3(17) |
| O15 | 8290(6) | 2357(7) | 2930(5) | 63(2) |
| O11 | 281(7) | 3827(6) | 5259(6) | 69(2) |
| O8 | 6158(8) | 4187(6) | 5896(5) | 62.9(19) |
| O13 | 2227(6) | 3845(6) | 5490(6) | 70(2) |
| O12 | 502(7) | 2513(6) | 4447(6) | 69(2) |
| C4 | 6439(7) | 1295(6) | 6340(5) | 26.1(16) |
| C5 | 2586(7) | 1443(6) | 4610(5) | 28.5(16) |
| C6 | 3154(8) | 1626(7) | 5702(5) | 32.2(17) |
| N5 | 1028(7) | 3396(7) | 5073(6) | 51(2) |
| C1 | 4254(7) | 2687(7) | 3906(5) | 27.4(16) |
| C13 | 5475(7) | 4964(6) | 1967(5) | 27.5(16) |
| C25 | 7492(7) | 2091(6) | 8422(5) | 27.3(16) |
| C19 | 8654(7) | 903(7) | 7456(5) | 28.9(16) |
| C7 | 3223(7) | 2737(7) | 1527(5) | 30.5(17) |
| O9 | 7966(8) | 5643(7) | 6633(6) | 73(2) |
| C12 | 2162(8) | 3024(8) | 1520(6) | 40(2) |
| C2 | 4790(7) | 3817(7) | 3537(5) | 27.8(16) |
| C18 | 5043(9) | 5799(7) | 2151(6) | 38.6(19) |
| C24 | 8902(8) | -76(7) | 7707(5) | 32.2(17) |
| C23 | 10047(8) | -138(8) | 7635(6) | 39(2) |
| C3 | 4964(7) | 986(7) | 6076(5) | 27.2(16) |
| C9 | 1778(9) | 1291(7) | 129(6) | 42(2) |
| C8 | 3023(8) | 1886(7) | 831(5) | 34.0(18) |
| C17 | 5403(9) | 6796(7) | 1758(6) | 39(2) |
| C29 | 7411(8) | 2678(8) | 9965(6) | 40(2) |
| C11 | 916(8) | 2435(8) | 826(7) | 45(2) |
| C10 | 738(9) | 1561(8) | 123(6) | 48(2) |
| C30 | 7124(7) | 1821(8) | 9238(6) | 36.1(19) |
| C26 | 8141(8) | 3258(7) | 8334(6) | 40(2) |
| C16 | 6159(9) | 6972(7) | 1173(7) | 44(2) |
| C20 | 9560(8) | 1759(9) | 7139(7) | 49(2) |
| C22 | 10949(9) | 739(9) | 7331(7) | 50(2) |

| | | | | |
|---|---|---|---|---|
| C28 | 8050(9) | 3840(8) | 9879(6) | 47(2) |
| C14 | 6249(11) | 5135(9) | 1369(8) | 60(3) |
| C27 | 8396(8) | 4161(8) | 9085(6) | 46(2) |
| C21 | 10686(9) | 1670(10) | 7072(8) | 59(3) |
| O1S | 872(11) | 5275(9) | 7092(8) | 112(4) |
| C15 | 6584(13) | 6144(10) | 963(10) | 78(4) |

**Table 3 Anisotropic Displacement Parameters (Å$^2$×10$^3$) for TB(NO3)3-2 (WATER SOLVATE). The Anisotropic displacement factor exponent takes the form: $-2\pi^2[h^2a^{*2}U_{11}+2hka^*b^*U_{12}+\ldots]$.**

| Atom | $U_{11}$ | $U_{22}$ | $U_{33}$ | $U_{23}$ | $U_{13}$ | $U_{12}$ |
|---|---|---|---|---|---|---|
| Tb1 | 28.6(2) | 23.1(2) | 34.5(2) | 14.24(15) | 15.03(15) | 14.07(16) |
| P1 | 31.9(9) | 21.8(10) | 30.2(10) | 14.9(8) | 13.2(8) | 14.9(8) |
| P2 | 25.4(9) | 24.3(10) | 29.7(10) | 13.2(8) | 11.9(7) | 13.0(8) |
| O4 | 28(2) | 25(3) | 31(3) | 15(2) | 12(2) | 12(2) |
| O1 | 32(3) | 32(3) | 37(3) | 19(2) | 19(2) | 16(2) |
| O2 | 38(3) | 26(3) | 44(3) | 18(2) | 17(2) | 20(2) |
| O3 | 37(3) | 28(3) | 32(3) | 14(2) | 12(2) | 17(2) |
| O6 | 41(3) | 31(3) | 42(3) | 19(2) | 17(2) | 22(2) |
| N4 | 36(4) | 24(3) | 36(4) | 17(3) | 23(3) | 21(3) |
| O14 | 31(3) | 47(4) | 46(3) | 8(3) | 18(3) | 9(3) |
| O7 | 79(5) | 53(4) | 50(4) | 11(3) | 36(3) | 42(4) |
| O5 | 57(4) | 39(3) | 46(3) | 26(3) | 27(3) | 33(3) |
| N1 | 33(3) | 33(4) | 40(4) | 20(3) | 21(3) | 23(3) |
| N2 | 33(3) | 24(3) | 35(3) | 12(3) | 16(3) | 17(3) |
| N3 | 40(4) | 31(4) | 37(4) | 14(3) | 19(3) | 23(3) |
| O10 | 61(4) | 50(4) | 68(5) | 13(3) | 31(4) | 26(3) |
| O15 | 35(3) | 89(6) | 53(4) | -11(4) | 22(3) | 10(3) |
| O11 | 40(4) | 60(5) | 103(6) | -12(4) | 20(4) | 21(3) |
| O8 | 73(5) | 44(4) | 70(5) | 21(4) | 26(4) | 21(4) |
| O13 | 39(4) | 55(5) | 108(6) | 19(4) | 12(4) | 20(3) |
| O12 | 60(4) | 41(4) | 101(6) | 0(4) | 15(4) | 27(4) |
| C4 | 31(4) | 22(4) | 28(4) | 14(3) | 12(3) | 12(3) |
| C5 | 27(4) | 22(4) | 44(5) | 16(3) | 18(3) | 13(3) |
| C6 | 45(4) | 37(5) | 34(4) | 20(4) | 25(4) | 28(4) |
| N5 | 33(4) | 45(5) | 81(6) | 30(4) | 20(4) | 18(4) |
| C1 | 26(4) | 32(4) | 29(4) | 12(3) | 8(3) | 17(3) |
| C13 | 34(4) | 22(4) | 31(4) | 15(3) | 14(3) | 13(3) |
| C25 | 29(4) | 21(4) | 39(4) | 13(3) | 15(3) | 15(3) |
| C19 | 27(4) | 37(5) | 31(4) | 13(3) | 15(3) | 18(3) |
| C7 | 30(4) | 30(4) | 31(4) | 19(3) | 9(3) | 11(3) |
| O9 | 67(5) | 50(5) | 96(6) | -8(4) | 28(4) | 19(4) |
| C12 | 46(5) | 35(5) | 41(5) | 12(4) | 11(4) | 21(4) |

| | | | | | | |
|---|---|---|---|---|---|---|
| C2 | 32(4) | 32(4) | 27(4) | 9(3) | 14(3) | 18(3) |
| C18 | 56(5) | 23(4) | 53(5) | 17(4) | 34(4) | 22(4) |
| C24 | 33(4) | 36(5) | 32(4) | 10(3) | 12(3) | 17(3) |
| C23 | 45(5) | 56(6) | 31(4) | 14(4) | 13(4) | 34(4) |
| C3 | 35(4) | 28(4) | 25(4) | 11(3) | 15(3) | 16(3) |
| C9 | 60(5) | 30(5) | 35(5) | 19(4) | 19(4) | 14(4) |
| C8 | 46(4) | 28(4) | 28(4) | 16(3) | 16(3) | 11(4) |
| C17 | 54(5) | 23(4) | 47(5) | 11(4) | 18(4) | 19(4) |
| C29 | 48(5) | 48(6) | 31(4) | 7(4) | 23(4) | 21(4) |
| C11 | 36(4) | 44(5) | 58(6) | 22(5) | 8(4) | 24(4) |
| C10 | 48(5) | 37(5) | 40(5) | 12(4) | 1(4) | 8(4) |
| C30 | 32(4) | 39(5) | 40(5) | 16(4) | 18(3) | 13(4) |
| C26 | 44(5) | 30(5) | 41(5) | 12(4) | 15(4) | 7(4) |
| C16 | 66(6) | 23(4) | 54(6) | 26(4) | 34(5) | 17(4) |
| C20 | 38(5) | 53(6) | 78(7) | 45(5) | 34(5) | 27(4) |
| C22 | 37(4) | 80(7) | 52(6) | 27(5) | 22(4) | 35(5) |
| C28 | 58(5) | 38(5) | 40(5) | -4(4) | 13(4) | 18(4) |
| C14 | 86(7) | 37(5) | 97(8) | 39(6) | 65(7) | 39(5) |
| C27 | 33(4) | 45(5) | 28(4) | 15(4) | 1(3) | -11(4) |
| C21 | 50(5) | 70(7) | 83(8) | 56(6) | 41(5) | 34(5) |
| O1S | 96(7) | 93(8) | 99(8) | 28(6) | 24(6) | -4(6) |
| C15 | 108(9) | 57(7) | 126(11) | 58(7) | 95(9) | 48(7) |

**Table 4 Bond Lengths for TB(NO3)3-2 (WATER SOLVATE).**

| Atom | Atom | Length/Å | Atom | Atom | Length/Å |
|---|---|---|---|---|---|
| Tb1 | O4[1] | 2.310(5) | O13 | N5 | 1.210(9) |
| Tb1 | O1 | 2.412(5) | O12 | N5 | 1.247(11) |
| Tb1 | O2 | 2.277(5) | C4 | C3 | 1.499(10) |
| Tb1 | O3[1] | 2.352(5) | C5 | C6 | 1.521(11) |
| Tb1 | O6 | 2.452(6) | C1 | C2 | 1.510(10) |
| Tb1 | O14 | 2.356(5) | C13 | C18 | 1.384(11) |
| Tb1 | O5 | 2.455(6) | C13 | C14 | 1.381(12) |
| Tb1 | N3 | 2.889(7) | C25 | C30 | 1.389(11) |
| Tb1 | O15 | 2.414(6) | C25 | C26 | 1.405(11) |
| P1 | O2 | 1.509(5) | C19 | C24 | 1.418(11) |
| P1 | C13 | 1.792(7) | C19 | C20 | 1.377(11) |
| P1 | C7 | 1.798(7) | C7 | C12 | 1.396(11) |
| P1 | C2 | 1.799(7) | C7 | C8 | 1.369(11) |
| P2 | O4 | 1.511(5) | C12 | C11 | 1.384(11) |
| P2 | C4 | 1.815(7) | C18 | C17 | 1.372(11) |
| P2 | C25 | 1.786(8) | C24 | C23 | 1.376(11) |
| P2 | C19 | 1.796(7) | C23 | C22 | 1.378(13) |

| | | | | | |
|---|---|---|---|---|---|
| O1 | C1 | 1.261(8) | C9 | C8 | 1.388(11) |
| O3 | C3 | 1.266(9) | C9 | C10 | 1.360(13) |
| O6 | N3 | 1.265(8) | C17 | C16 | 1.350(12) |
| N4 | O10 | 1.244(8) | C29 | C30 | 1.371(12) |
| N4 | O8 | 1.141(9) | C29 | C28 | 1.397(13) |
| N4 | O9 | 1.308(9) | C11 | C10 | 1.405(13) |
| O7 | N3 | 1.202(8) | C26 | C27 | 1.447(13) |
| O5 | N3 | 1.284(8) | C16 | C15 | 1.382(14) |
| N1 | C5 | 1.456(9) | C20 | C21 | 1.367(12) |
| N1 | C1 | 1.320(9) | C22 | C21 | 1.372(13) |
| N2 | C6 | 1.462(9) | C28 | C27 | 1.360(12) |
| N2 | C3 | 1.312(9) | C14 | C15 | 1.394(13) |
| O11 | N5 | 1.264(10) | | | |

[1]1-X,-Y,1-Z

**Table 5 Bond Angles for TB(NO3)3-2 (WATER SOLVATE).**

| Atom | Atom | Atom | Angle/° | Atom | Atom | Atom | Angle/° |
|---|---|---|---|---|---|---|---|
| O4[1] | Tb1 | O1 | 72.89(18) | O8 | N4 | O9 | 119.2(7) |
| O4[1] | Tb1 | O3[1] | 79.05(17) | N3 | O5 | Tb1 | 96.1(4) |
| O4[1] | Tb1 | O6 | 76.62(18) | C1 | N1 | C5 | 124.9(6) |
| O4[1] | Tb1 | O14 | 142.21(19) | C3 | N2 | C6 | 123.1(7) |
| O4[1] | Tb1 | O5 | 79.35(19) | O6 | N3 | Tb1 | 57.4(4) |
| O4[1] | Tb1 | N3 | 78.85(18) | O6 | N3 | O5 | 114.5(6) |
| O4[1] | Tb1 | O15 | 147.0(2) | O7 | N3 | Tb1 | 172.7(6) |
| O1 | Tb1 | O6 | 136.89(16) | O7 | N3 | O6 | 123.5(7) |
| O1 | Tb1 | O5 | 144.65(19) | O7 | N3 | O5 | 122.0(6) |
| O1 | Tb1 | N3 | 151.38(18) | O5 | N3 | Tb1 | 57.7(4) |
| O1 | Tb1 | O15 | 137.8(2) | C3 | C4 | P2 | 112.8(5) |
| O2 | Tb1 | O4[1] | 93.84(17) | N1 | C5 | C6 | 112.3(6) |
| O2 | Tb1 | O1 | 77.64(18) | N2 | C6 | C5 | 110.7(6) |
| O2 | Tb1 | O3[1] | 159.75(19) | O13 | N5 | O11 | 120.1(9) |
| O2 | Tb1 | O6 | 74.75(18) | O13 | N5 | O12 | 121.4(8) |
| O2 | Tb1 | O14 | 92.6(2) | O12 | N5 | O11 | 118.5(7) |
| O2 | Tb1 | O5 | 126.38(19) | O1 | C1 | N1 | 121.9(7) |
| O2 | Tb1 | N3 | 100.16(19) | O1 | C1 | C2 | 120.7(6) |
| O2 | Tb1 | O15 | 84.6(2) | N1 | C1 | C2 | 117.4(6) |
| O3[1] | Tb1 | O1 | 82.14(17) | C18 | C13 | P1 | 122.1(6) |
| O3[1] | Tb1 | O6 | 120.99(18) | C14 | C13 | P1 | 118.1(6) |
| O3[1] | Tb1 | O14 | 82.15(19) | C14 | C13 | C18 | 119.3(7) |
| O3[1] | Tb1 | O5 | 71.32(18) | C30 | C25 | P2 | 119.2(6) |
| O3[1] | Tb1 | N3 | 97.04(18) | C30 | C25 | C26 | 119.4(8) |
| O3[1] | Tb1 | O15 | 111.7(2) | C26 | C25 | P2 | 121.5(6) |

| | | | | | | | |
|---|---|---|---|---|---|---|---|
| O6 | Tb1 | O5 | 51.82(17) | C24 | C19 | P2 | 118.5(6) |
| O6 | Tb1 | N3 | 25.77(17) | C20 | C19 | P2 | 122.2(6) |
| O14 | Tb1 | O1 | 72.27(18) | C20 | C19 | C24 | 119.2(7) |
| O14 | Tb1 | O6 | 140.73(19) | C12 | C7 | P1 | 120.6(6) |
| O14 | Tb1 | O5 | 124.5(2) | C8 | C7 | P1 | 119.7(6) |
| O14 | Tb1 | N3 | 136.18(19) | C8 | C7 | C12 | 119.3(7) |
| O14 | Tb1 | O15 | 70.7(2) | C11 | C12 | C7 | 120.0(8) |
| O5 | Tb1 | N3 | 26.23(17) | C1 | C2 | P1 | 111.3(5) |
| O15 | Tb1 | O6 | 71.2(2) | C17 | C18 | C13 | 120.3(8) |
| O15 | Tb1 | O5 | 75.4(2) | C23 | C24 | C19 | 119.1(8) |
| O15 | Tb1 | N3 | 69.1(2) | C24 | C23 | C22 | 120.7(8) |
| O2 | P1 | C13 | 112.2(3) | O3 | C3 | N2 | 121.6(7) |
| O2 | P1 | C7 | 110.5(3) | O3 | C3 | C4 | 120.0(6) |
| O2 | P1 | C2 | 110.7(3) | N2 | C3 | C4 | 118.3(7) |
| C13 | P1 | C7 | 106.5(3) | C10 | C9 | C8 | 119.8(9) |
| C13 | P1 | C2 | 107.6(4) | C7 | C8 | C9 | 121.1(8) |
| C7 | P1 | C2 | 109.2(4) | C16 | C17 | C18 | 120.9(8) |
| O4 | P2 | C4 | 111.5(3) | C30 | C29 | C28 | 120.0(8) |
| O4 | P2 | C25 | 110.8(3) | C12 | C11 | C10 | 119.4(8) |
| O4 | P2 | C19 | 112.4(3) | C9 | C10 | C11 | 120.3(8) |
| C25 | P2 | C4 | 108.7(3) | C29 | C30 | C25 | 120.9(8) |
| C25 | P2 | C19 | 109.8(4) | C25 | C26 | C27 | 119.6(8) |
| C19 | P2 | C4 | 103.4(3) | C17 | C16 | C15 | 120.1(8) |
| P2 | O4 | Tb1[1] | 137.3(3) | C21 | C20 | C19 | 120.4(8) |
| C1 | O1 | Tb1 | 139.1(5) | C21 | C22 | C23 | 119.7(8) |
| P1 | O2 | Tb1 | 133.7(3) | C27 | C28 | C29 | 121.9(8) |
| C3 | O3 | Tb1[1] | 131.3(4) | C13 | C14 | C15 | 119.5(9) |
| N3 | O6 | Tb1 | 96.8(4) | C28 | C27 | C26 | 118.1(8) |
| O10 | N4 | O9 | 113.6(7) | C20 | C21 | C22 | 120.9(9) |
| O8 | N4 | O10 | 127.3(8) | C16 | C15 | C14 | 119.9(9) |

[1]1-X,-Y,1-Z

**Table 6 Hydrogen Bonds for TB(NO3)3-2 (WATER SOLVATE).**

| D | H | A | d(D-H)/Å | d(H-A)/Å | d(D-A)/Å | D-H-A/° |
|---|---|---|---|---|---|---|
| O14 | H14A | O1 | 0.88 | 2.44 | 2.811(7) | 105.6 |
| O14 | H14A | O10 | 0.88 | 2.13 | 2.714(9) | 123.5 |
| O14 | H14B | O11[1] | 0.88 | 2.15 | 2.709(9) | 121.3 |
| N1 | H1 | O13 | 0.88 | 2.60 | 3.225(10) | 128.5 |
| N1 | H1 | O12 | 0.88 | 2.70 | 3.240(9) | 121.0 |
| N1 | H1 | O9[2] | 0.88 | 2.48 | 3.301(10) | 154.6 |
| N2 | H2 | O8 | 0.88 | 2.06 | 2.876(10) | 154.4 |
| O15 | H15B | O12[1] | 0.91 | 1.97 | 2.785(10) | 147.9 |

| | | | | |
|---|---|---|---|---|
| O1S H1SA O11 | 0.87 | 2.41 | 2.960(14) | 121.6 |
| O1S H1SB O10[3] | 0.87 | 2.00 | 2.856(13) | 165.8 |

[1]1+X,+Y,+Z; [2]1-X,1-Y,1-Z; [3]-1+X,+Y,+Z

**Table 7 Hydrogen Atom Coordinates (Å×10⁴) and Isotropic Displacement Parameters (Å²×10³) for TB(NO3)3-2 (WATER SOLVATE).**

| Atom | *x* | *y* | *z* | U(eq) |
|---|---|---|---|---|
| H14A | 7165.98 | 3054.23 | 4759.97 | 64 |
| H14B | 8218.12 | 3289.63 | 4396.14 | 64 |
| H1 | 2887.25 | 2994.65 | 4181.49 | 36 |
| H2 | 5183.99 | 2546.62 | 6166.14 | 33 |
| H15A | 8305.09 | 2113.97 | 2341.63 | 95 |
| H15B | 8860.39 | 2110.68 | 3342.33 | 95 |
| H4A | 6921.91 | 2136.1 | 6349.55 | 31 |
| H4B | 6596.63 | 877.44 | 5840.89 | 31 |
| H5A | 2720.64 | 794.3 | 4335.57 | 34 |
| H5B | 1607.58 | 1224.07 | 4415.22 | 34 |
| H6A | 3065.26 | 2299.58 | 5979.42 | 39 |
| H6B | 2633.57 | 934.55 | 5942.93 | 39 |
| H12 | 2294.75 | 3624.43 | 1992.53 | 48 |
| H2A | 5696.01 | 4324.56 | 3982.95 | 33 |
| H2B | 4195.8 | 4214.41 | 3518.54 | 33 |
| H18 | 4495.77 | 5681.05 | 2551.65 | 46 |
| H24 | 8285.38 | -679.41 | 7921.72 | 39 |
| H23 | 10217.19 | -793.37 | 7796.95 | 47 |
| H9 | 1652.99 | 696.52 | -344.85 | 50 |
| H8 | 3748.42 | 1698.69 | 827.12 | 41 |
| H17 | 5115.66 | 7369.99 | 1900.37 | 47 |
| H29 | 7175.49 | 2481.15 | 10526.52 | 47 |
| H11 | 187.51 | 2619.3 | 824.87 | 54 |
| H10 | -112.84 | 1157.49 | -359.04 | 57 |
| H30 | 6667.31 | 1033.31 | 9293.11 | 43 |
| H26 | 8409.8 | 3453.37 | 7784.01 | 49 |
| H16 | 6398.93 | 7666.13 | 904.59 | 53 |
| H20 | 9400.08 | 2415.53 | 6966.05 | 59 |
| H22 | 11749.55 | 698.71 | 7300.31 | 60 |
| H28 | 8247.82 | 4423.91 | 10390.7 | 56 |
| H14 | 6551.37 | 4568.68 | 1235.56 | 72 |
| H27 | 8795.21 | 4954.62 | 9024.77 | 56 |
| H21 | 11294.83 | 2261.97 | 6842.55 | 71 |

| | | | | |
|---|---|---|---|---|
| H1SA | 1102.15 | 4746.8 | 6913.51 | 168 |
| H1SB | 20.08 | 5020.92 | 6769.86 | 168 |
| H15 | 7103.74 | 6262.52 | 542.15 | 94 |

**Refinement model description**

Number of restraints - 0, number of constraints - unknown.

Details:

1. Fixed Uiso

At 1.2 times of:

All C(H) groups, All C(H,H) groups, All N(H) groups

At 1.5 times of:

All O(H,H) groups

2.a Free rotating group:

O1S(H1SA,H1SB)

2.b Rotating group:

O14(H14A,H14B), O15(H15A,H15B)

2.c Secondary CH2 refined with riding coordinates:

C4(H4A,H4B), C5(H5A,H5B), C6(H6A,H6B), C2(H2A,H2B)

2.d Aromatic/amide H refined with riding coordinates:

N1(H1), N2(H2), C12(H12), C18(H18), C24(H24), C23(H23), C9(H9), C8(H8), C17(H17), C29(H29), C11(H11), C10(H10), C30(H30), C26(H26), C16(H16), C20(H20), C22(H22), C28(H28), C14(H14), C27(H27), C21(H21), C15(H15)

## II. Luminescence Data

**A. Diagram of a possible mechanism for the sensitization process** for lanthanide luminescence using an abbreviated Jablonski diagram: dashed arrows = fluorescence (from $^{1}S$) and phosphorescence (from $^{3}T$) of ligand, squiggly arrows = non-radiative decay pathways, IC = internal conversion, ISC = inter-system crossing, ET = energy transfer.

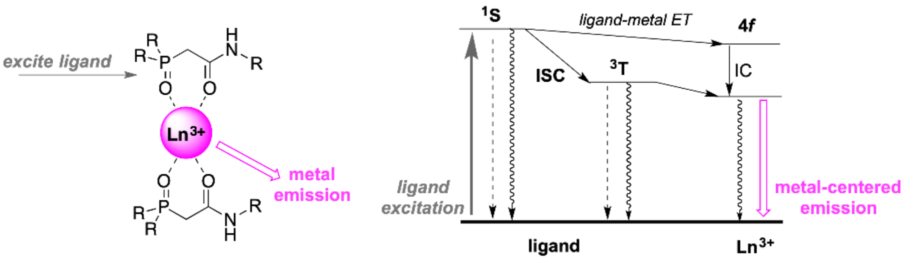


**B. Absorption and Excitation spectra**

The absorption and excitation spectra for each Ln-ligand complex are shown below. For a given complex, the spectra are presented on the same set of axes and the intensity values have been normalized to 1 for ease of comparison. Absorption spectra are shown with solid lines, while excitation spectra are shown with dashed lines.

**1.** Absorption spectra of free ligands **1** and **2** (acetonitrile, 1.0 mM, 1.0 nm slits). Note: ligand **2** has quite poor solubility in acetonitrile, and there was some undissolved ligand in the cuvette. We attribute the high baseline of the UV-VIS spectrum to this feature. No attempt was made to correct for this. Purple dashed line: ethoxy-substituted ligand **1**, orange solid line: phenyl-substituted ligand **2**.

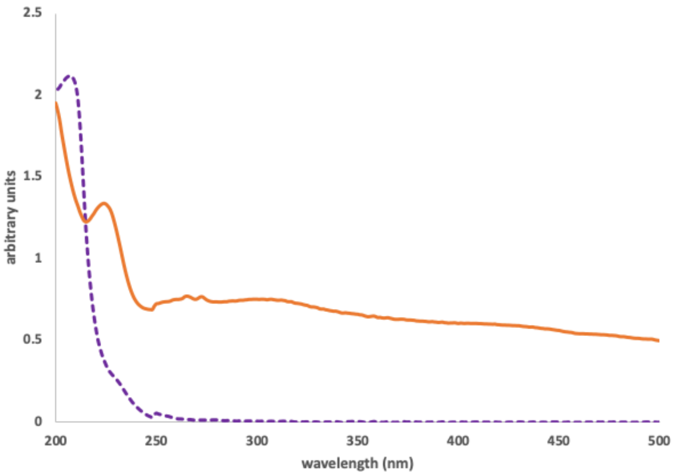

**2. Eu(NO$_3$)$_3$-1** (excitation spectrum monitored at 620 nm, 2.0 nm excitation and emission slits)

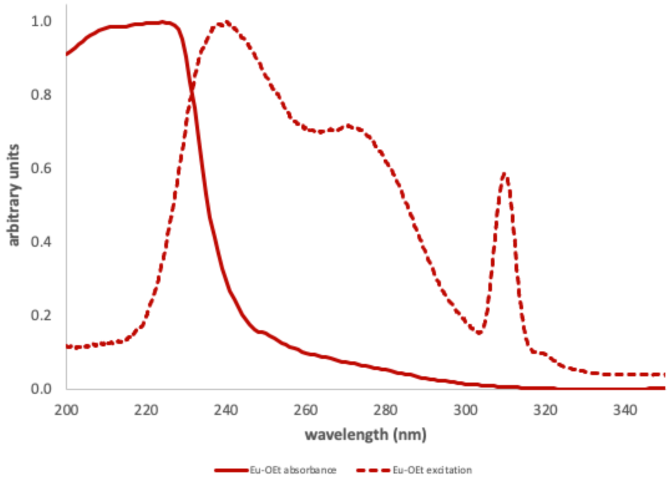


**3. Tb(NO$_3$)$_3$-1** (excitation spectrum monitored at 545 nm, 2.0 nm excitation and emission slits)

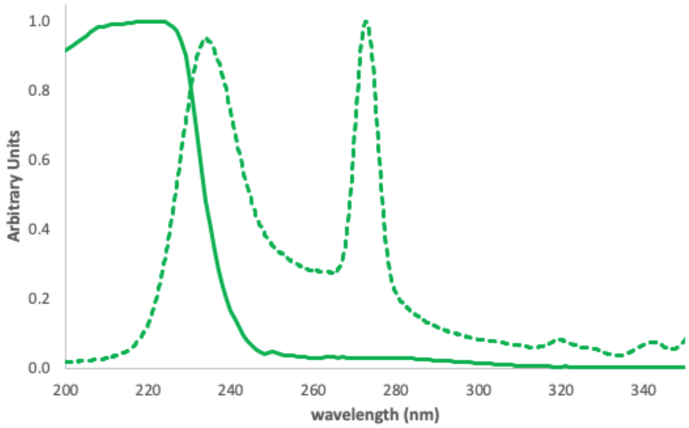


**4. Eu(NO$_3$)$_3$-2** (excitation spectrum monitored at 617 nm, 2.0 nm excitation and emission slits)

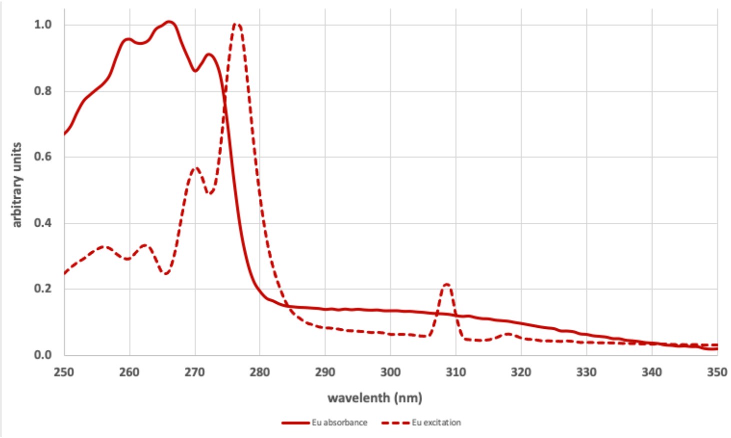

**5. Tb(NO$_3$)$_3$-2** (excitation spectrum monitored at 544 nm, 2.0 mm excitation and emission slits)

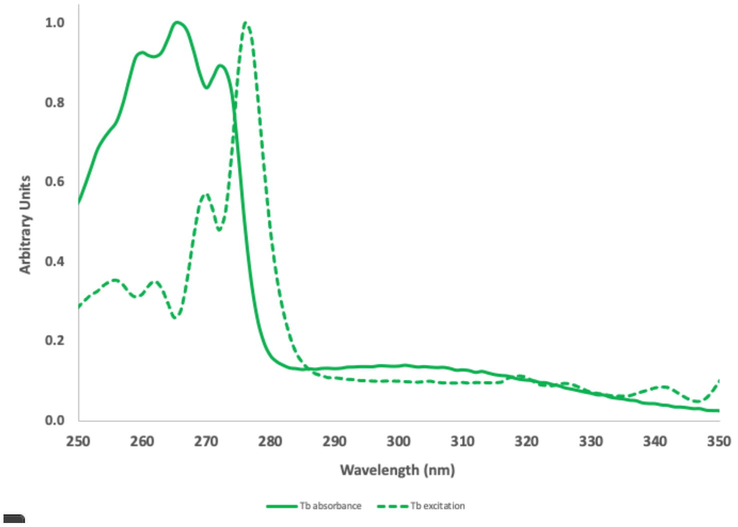

## C. Lifetime decay curves

### 1. Ligand 2-Eu complex

Fluorimeter settings for all trials:
excitation: 260 nm; emission: 616 nm; slits: 2.0 nm; sample window: 1.5 ms; time between flashes: 41 ms; count (averages): 100; initial delay: 0.05 ms; max delay: 15 ms; delay increment: 0.05 ms

**Trial #1**

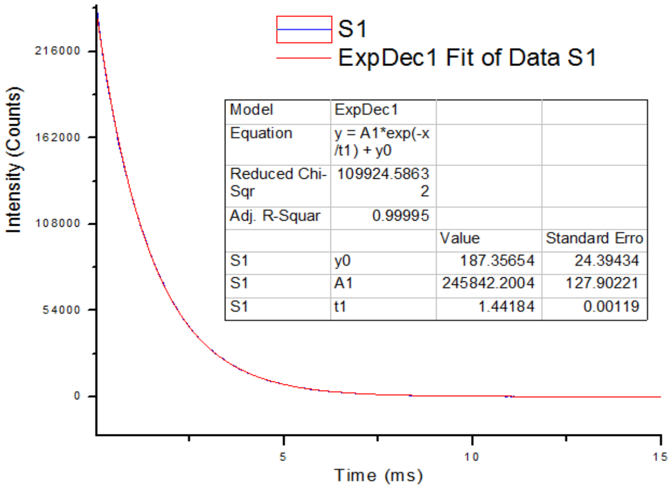


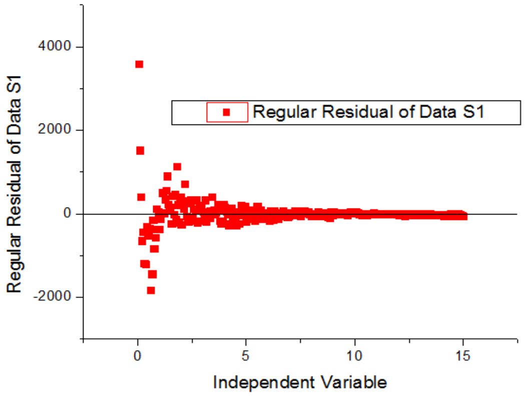


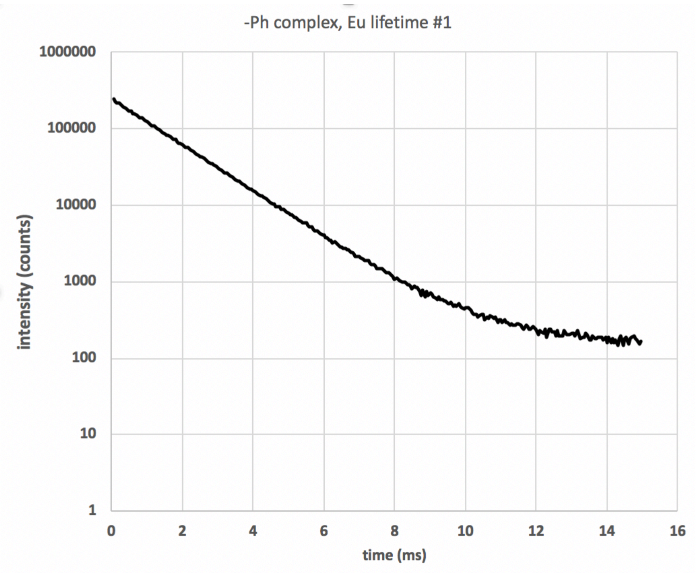

## Trial #2

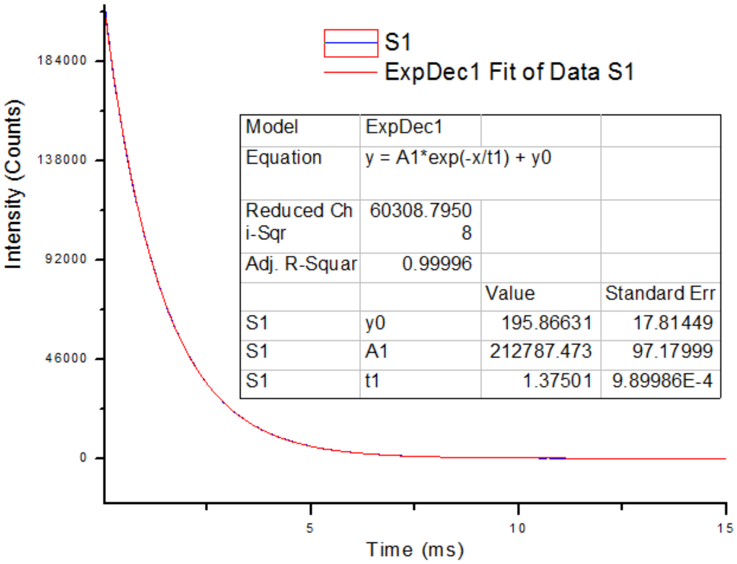
S1
ExpDec1 Fit of Data S1
Model | ExpDec1
Equation | y = A1*exp(-x/t1) + y0
Reduced Chi-Sqr | 60308.79508
Adj. R-Square | 0.99996
Value | Standard Err
S1 | y0 | 195.86631 | 17.81449
S1 | A1 | 212787.473 | 97.17999
S1 | t1 | 1.37501 | 9.89986E-4
Intensity (Counts)
184000
138000
92000
46000
0
5
10
15
Time (ms)

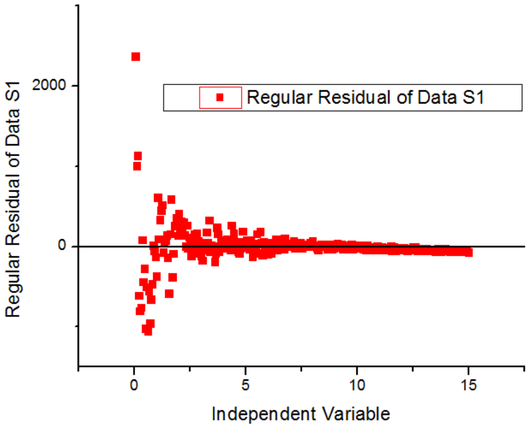
Regular Residual of Data S1
Regular Residual of Data S1
2000
0
0
5
10
15
Independent Variable

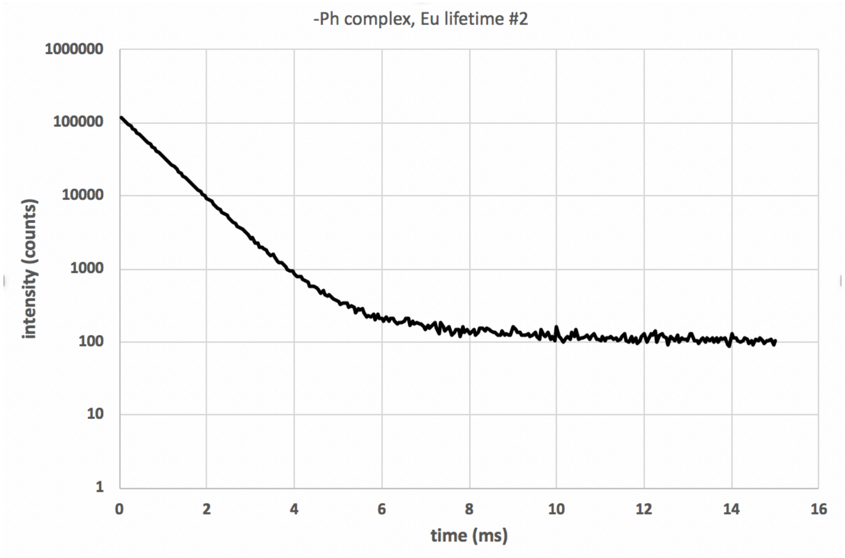
-Ph complex, Eu lifetime #2
intensity (counts)
1000000
100000
10000
1000
100
10
1
0
2
4
6
8
10
12
14
16
time (ms)

# Trial #3

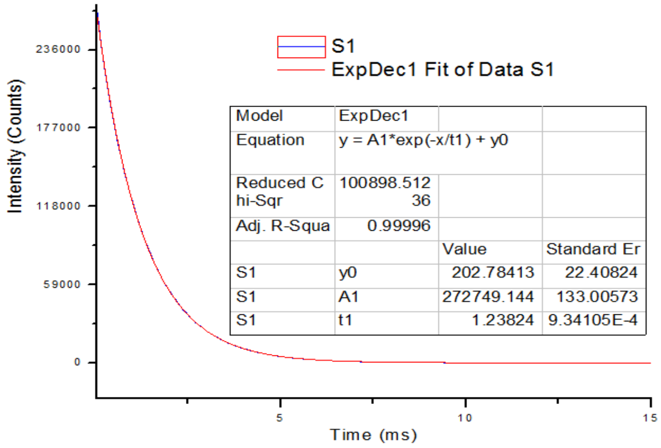
S1
ExpDec1 Fit of Data S1
Model ExpDec1
Equation y = A1*exp(-x/t1) + y0
Reduced Chi-Sqr 100898.51236
Adj. R-Squa 0.99996
Value Standard Er
S1 y0 202.78413 22.40824
S1 A1 272749.144 133.00573
S1 t1 1.23824 9.34105E-4
Intensity (Counts)
236000
177000
118000
59000
0
5
10
15
Time (ms)

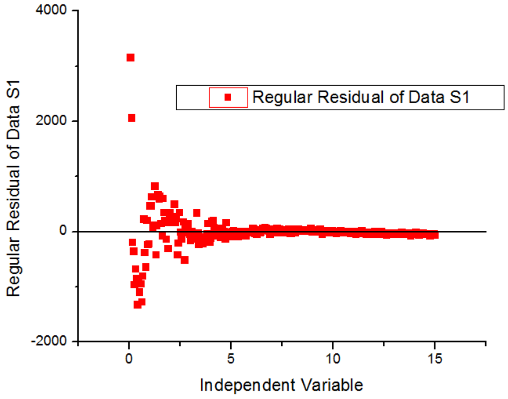
Regular Residual of Data S1
4000
2000
0
-2000
0
5
10
15
Regular Residual of Data S1
Independent Variable

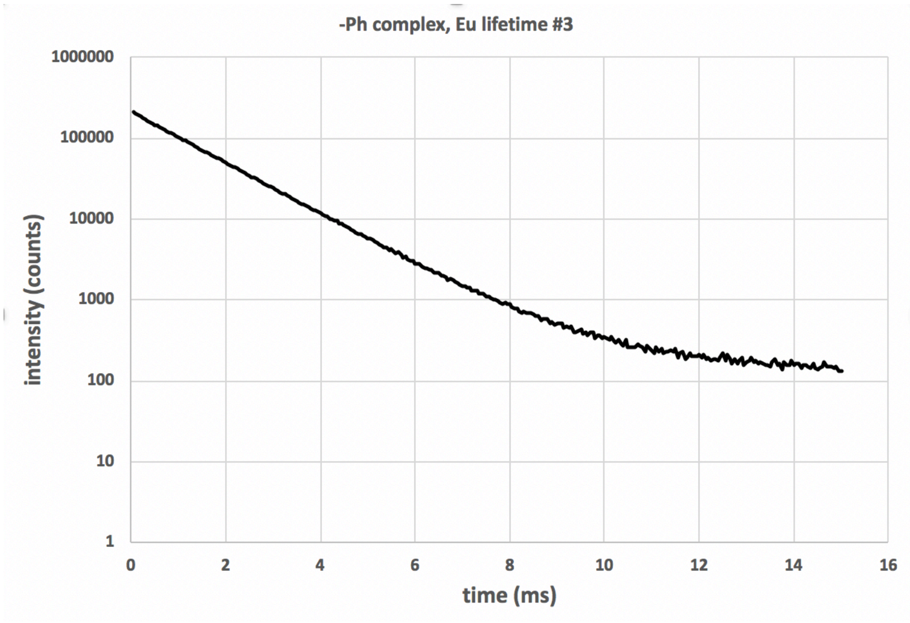
-Ph complex, Eu lifetime #3
1000000
100000
10000
1000
100
10
1
0
2
4
6
8
10
12
14
16
intensity (counts)
time (ms)

## 2. Ligand 2-Tb complex

Fluorimeter settings for all trials:

excitation: 260 nm; emission: 545 nm; slits: 2.0 nm; sample window: 1.9 ms; time between flashes: 41 ms; count (averages): 100; initial delay: 0.05 ms; max delay: 19 ms; delay increment: 0.05 ms

### <u>Trial #1</u>

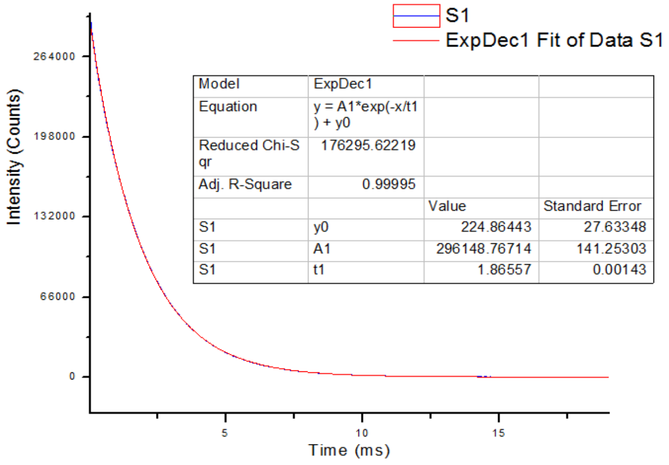


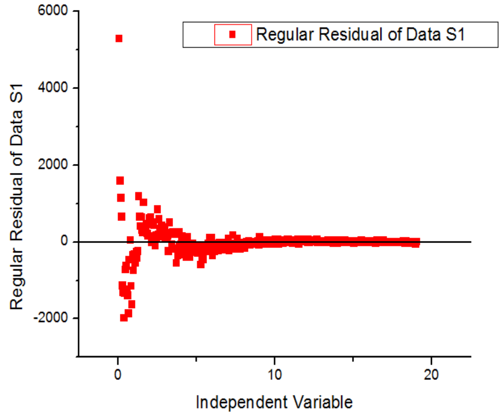


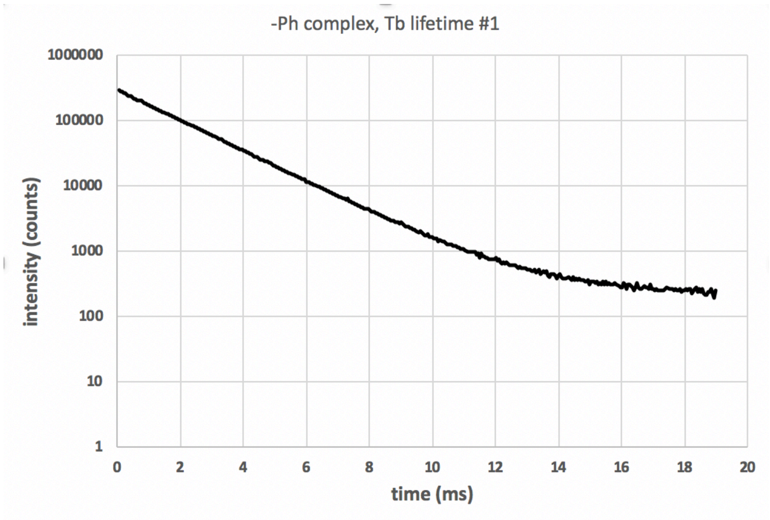

# Trial #2

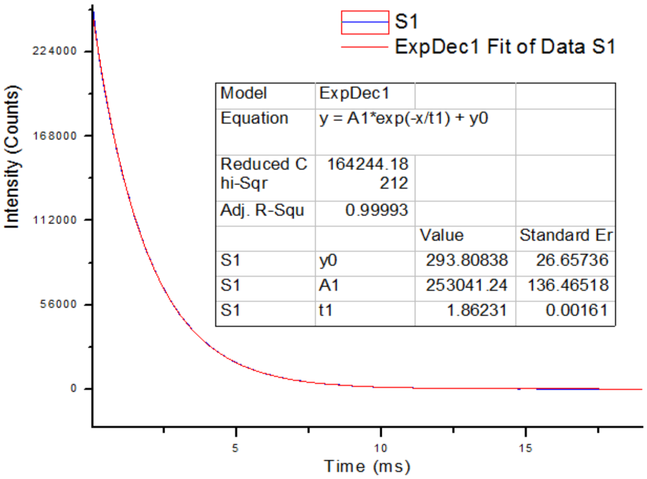
S1
ExpDec1 Fit of Data S1
Model ExpDec1
Equation y = A1*exp(-x/t1) + y0
Reduced Chi-Sqr 164244.18212
Adj. R-Squ 0.99993
Value Standard Er
S1 y0 293.80838 26.65736
S1 A1 253041.24 136.46518
S1 t1 1.86231 0.00161
Intensity (Counts)
224000
168000
112000
56000
0
5
10
15
Time (ms)

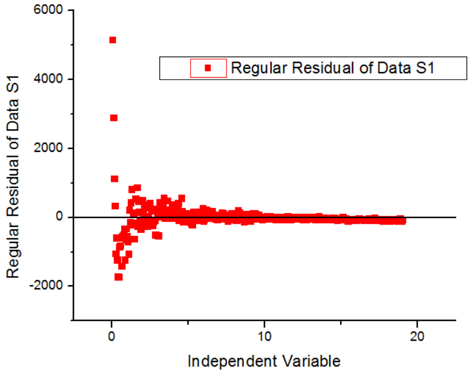
Regular Residual of Data S1
Regular Residual of Data S1
6000
4000
2000
0
-2000
0
10
20
Independent Variable

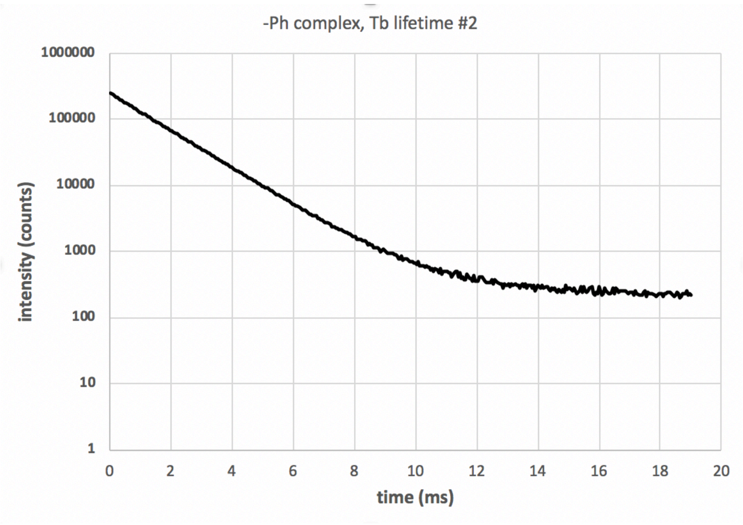
-Ph complex, Tb lifetime #2
intensity (counts)
1000000
100000
10000
1000
100
10
1
0
2
4
6
8
10
12
14
16
18
20
time (ms)

**Trial #3**

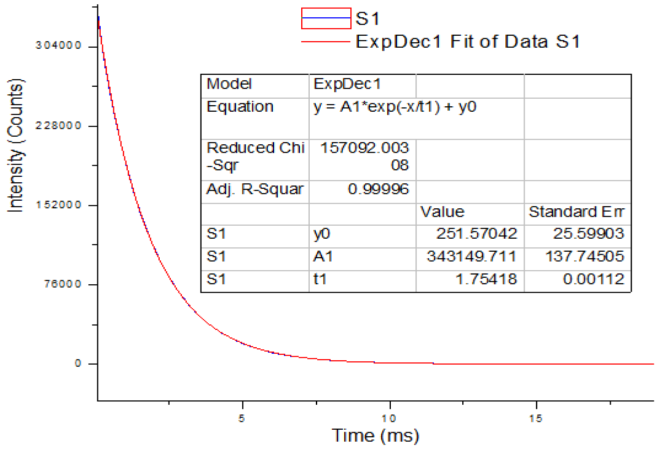
S1
ExpDec1 Fit of Data S1
Model ExpDec1
Equation y = A1*exp(-x/t1) + y0
Reduced Chi-Sqr 157092.00308
Adj. R-Squar 0.99996
Value Standard Err
S1 y0 251.57042 25.59903
S1 A1 343149.711 137.74505
S1 t1 1.75418 0.00112
Intensity (Counts)
304000
228000
152000
76000
0
5
10
15
Time (ms)

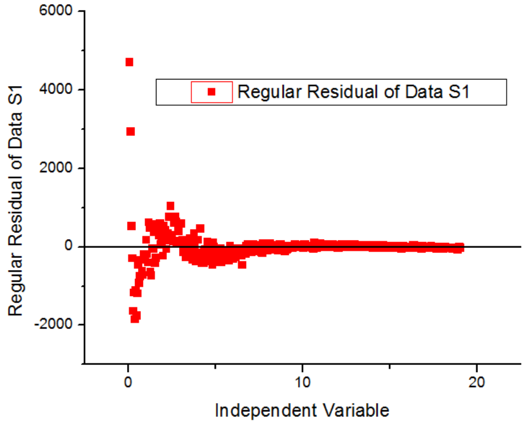
Regular Residual of Data S1
Regular Residual of Data S1
6000
4000
2000
0
-2000
0
10
20
Independent Variable

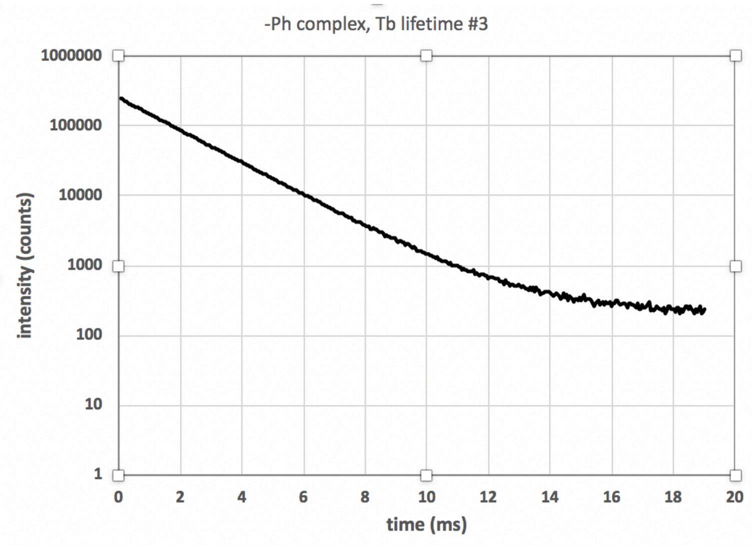
-Ph complex, Tb lifetime #3
intensity (counts)
1000000
100000
10000
1000
100
10
1
0
2
4
6
8
10
12
14
16
18
20
time (ms)
