# Supplementary material for: Two Beta-Phosphorylamide Compounds as Ligands for Sm3+, Eu3+, and Tb3+: X-ray Crystallography and Luminescence Properties
Source: Molecules. 2020 Jun 28;25(13):2971. doi: 10.3390/molecules25132971 (PMC7411983; doi:10.3390/molecules25132971)

checkCIF/PLATON report

Structure factors have been supplied for datablock(s) gvsu819h

THIS REPORT IS FOR GUIDANCE ONLY. IF USED AS PART OF A REVIEW PROCEDURE FOR PUBLICATION, IT SHOULD NOT REPLACE THE EXPERTISE OF AN EXPERIENCED CRYSTALLOGRAPHIC REFEREE.

No syntax errors found. CIF dictionary Interpreting this report

Datablock: gvsu819h

```
Bond precision:  C-C = 0.0263 A          Wavelength=1.54178

Cell:            a=18.2898(4)     b=18.2898(4)     c=27.4073(6)
                 alpha=90         beta=90          gamma=90
Temperature:     173 K
```

	Calculated	Reported
Volume	9168.2(4)	9168.2(4)
Space group	P -4 21 c	P -4 21 c
Hall group	P -4 2n	P -4 2n
Moiety formula	C62 H68 N8 O22 P4 Tb2, 2(N O3), 2(C H4 O)	C62 H68 N8 O22 P4 Tb2, 2(N O3), 2(C H4 O)
Sum formula	C64 H76 N10 O30 P4 Tb2	C64 H76 N10 O30 P4 Tb2
Mr	1907.09	1907.06
Dx,g cm-3	1.382	1.382
Z	4	4
Mu (mm-1)	8.794	8.794
F000	3840.0	3840.0
F000’	3783.58	
h,k,lmax	22,22,33	22,22,33
Nref	8428[4594]	8408
Tmin,Tmax	0.182,0.200	0.214,0.428
Tmin’	0.055	

```
Correction method= # Reported T Limits: Tmin=0.214 Tmax=0.428
AbsCorr = MULTI-SCAN

Data completeness= 1.83/1.00      Theta(max)= 68.338

R(reflections)= 0.0638( 6739)     wR2(reflections)= 0.2052( 8408)

S = 1.094                  Npar= 536
```

The following ALERTS were generated. Each ALERT has the format
test-name_ALERT_alert-type_alert-level.
Click on the hyperlinks for more details of the test.

Alert level A

```
PLAT602_ALERT_2_A VERY LARGE Solvent Accessible VOID(S) in Structure        ! Info
```

Author Response: There is a minimal amount of electron density left in this structure; we propose that extensive hydrogen bonding network holding these compounds together also supports the relatively large void.

Alert level B

```
PLAT342_ALERT_3_B Low Bond Precision on  C-C Bonds ...............    0.02629 Ang.
PLAT934_ALERT_3_B Number of (Iobs-Icalc)/Sigma(W) > 10 Outliers ..          3 Check
```

Author Response: This is a minimal number of outliers.

```
PLAT987_ALERT_1_B The Flack x is >> 0 -  Do a BASF/TWIN Refinement     Please Check
```

Author Response: A TWIN refinement revealed no applicable TWIN laws.

Alert level C

```
RINTA01_ALERT_3_C  The value of Rint is greater than 0.12
            Rint given   0.133
PLAT234_ALERT_4_C Large Hirshfeld Difference C9       --C10      .       0.22 Ang.
PLAT241_ALERT_2_C High   'MainMol' Ueq as Compared to Neighbors of         O5 Check
PLAT241_ALERT_2_C High   'MainMol' Ueq as Compared to Neighbors of         C9 Check
PLAT260_ALERT_2_C Large Average Ueq of Residue Including       O11      0.102 Check
PLAT331_ALERT_2_C Small Aver Phenyl C-C Dist C13A      -C18A     .       1.37 Ang.
PLAT918_ALERT_3_C Reflection(s) with I(obs) much Smaller I(calc) .          2 Check
PLAT976_ALERT_2_C Check Calcd Resid. Dens.  0.86A   From O1             -0.41 eA-3
PLAT976_ALERT_2_C Check Calcd Resid. Dens.  0.63A   From O13            -0.40 eA-3
PLAT978_ALERT_2_C Number C-C Bonds with Positive Residual Density.          0 Info
```

Alert level G

```
PLAT002_ALERT_2_G Number of Distance or Angle Restraints on AtSite         20 Note
PLAT003_ALERT_2_G Number of Uiso or Uij Restrained non-H Atoms ...         18 Report
PLAT007_ALERT_5_G Number of Unrefined Donor-H Atoms .............           3 Report
PLAT020_ALERT_3_G The Value of Rint is Greater Than 0.12 .........      0.133 Report
PLAT072_ALERT_2_G SHELXL First  Parameter in WGHT  Unusually Large       0.13 Report
PLAT171_ALERT_4_G The CIF-Embedded .res File Contains EADP Records          5 Report
PLAT172_ALERT_4_G The CIF-Embedded .res File Contains DFIX Records          2 Report
PLAT173_ALERT_4_G The CIF-Embedded .res File Contains DANG Records          2 Report
PLAT174_ALERT_4_G The CIF-Embedded .res File Contains FLAT Records          1 Report
PLAT175_ALERT_4_G The CIF-Embedded .res File Contains SAME Records          1 Report
PLAT177_ALERT_4_G The CIF-Embedded .res File Contains DELU Records          3 Report
PLAT178_ALERT_4_G The CIF-Embedded .res File Contains SIMU Records          3 Report
PLAT301_ALERT_3_G Main Residue  Disorder ..............(Resd  1  )        14% Note
PLAT432_ALERT_2_G Short Inter X...Y Contact  O11      ..C17A             2.92 Ang.
                                        1/2-x,-1/2+y,3/2-z  =      5_546 Check
```

```
PLAT432_ALERT_2_G Short Inter X...Y Contact  O11      ..C18A            3.02 Ang.
                                         1/2-x,-1/2+y,3/2-z  =      5_546 Check
PLAT720_ALERT_4_G Number of Unusual/Non-Standard Labels ..........         10 Note
PLAT794_ALERT_5_G Tentative Bond Valency for Tb1       (III)     .       3.52 Info
PLAT860_ALERT_3_G Number of Least-Squares Restraints .............        165 Note
PLAT912_ALERT_4_G Missing # of FCF Reflections Above STh/L=  0.600          2 Note
```

```
  1 ALERT level A = Most likely a serious problem - resolve or explain
  3 ALERT level B = A potentially serious problem, consider carefully
 10 ALERT level C = Check. Ensure it is not caused by an omission or oversight
 19 ALERT level G = General information/check it is not something unexpected

  1 ALERT type 1 CIF construction/syntax error, inconsistent or missing data
 13 ALERT type 2 Indicator that the structure model may be wrong or deficient
  7 ALERT type 3 Indicator that the structure quality may be low
 10 ALERT type 4 Improvement, methodology, query or suggestion
  2 ALERT type 5 Informative message, check
```

It is advisable to attempt to resolve as many as possible of the alerts in all categories. Often the minor alerts point to easily fixed oversights, errors and omissions in your CIF or refinement strategy, so attention to these fine details can be worthwhile. In order to resolve some of the more serious problems it may be necessary to carry out additional measurements or structure refinements. However, the purpose of your study may justify the reported deviations and the more serious of these should normally be commented upon in the discussion or experimental section of a paper or in the "special_details" fields of the CIF. checkCIF was carefully designed to identify outliers and unusual parameters, but every test has its limitations and alerts that are not important in a particular case may appear. Conversely, the absence of alerts does not guarantee there are no aspects of the results needing attention. It is up to the individual to critically assess their own results and, if necessary, seek expert advice.

Publication of your CIF in IUCr journals

A basic structural check has been run on your CIF. These basic checks will be run on all CIFs submitted for publication in IUCr journals (*Acta Crystallographica*, *Journal of Applied Crystallography*, *Journal of Synchrotron Radiation*); however, if you intend to submit to *Acta Crystallographica Section C* or *E* or *IUCrData*, you should make sure that full publication checks are run on the final version of your CIF prior to submission.

Publication of your CIF in other journals

Please refer to the *Notes for Authors* of the relevant journal for any special instructions relating to CIF submission.

PLATON version of 07/08/2019; check.def file version of 30/07/2019

Datablock gvsu819h - ellipsoid plot

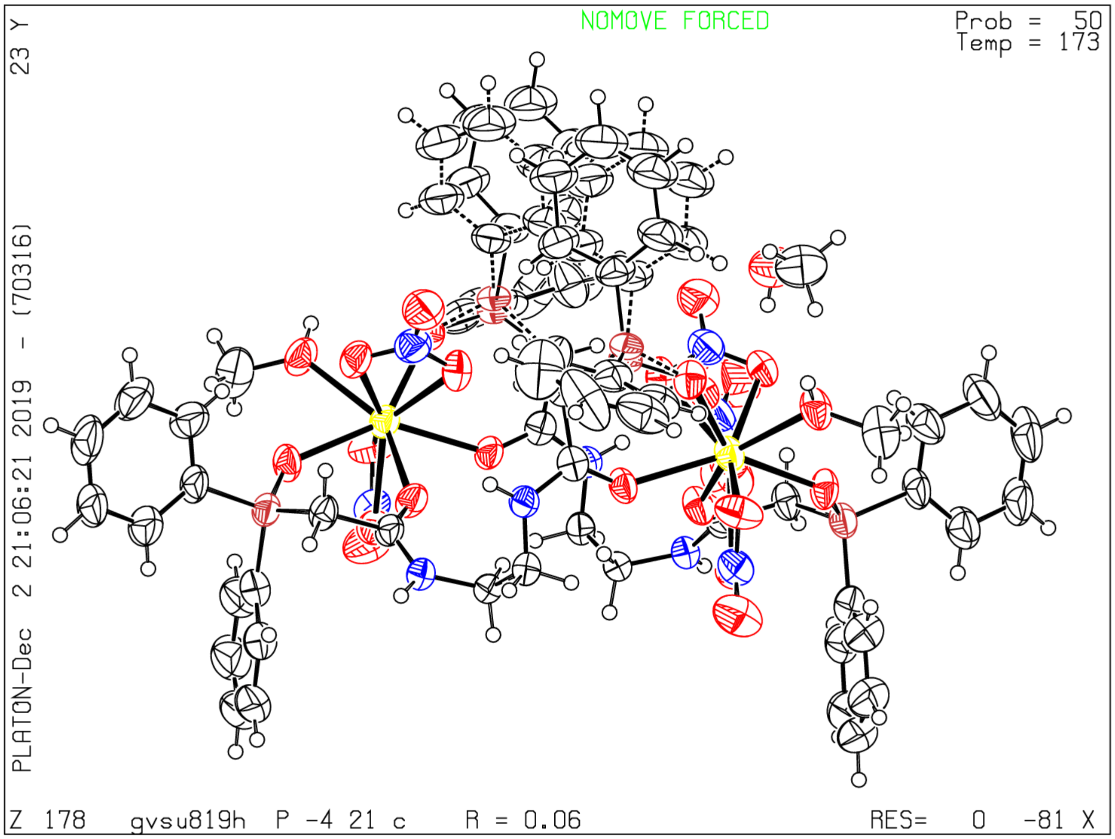

Supplement: Supplementary file 1 [file molecules-25-02971-s001.zip › supp_files_rev2/check_cif_reports/GVSU819h_checkcif.pdf]
