# Supplementary material for: Two Beta-Phosphorylamide Compounds as Ligands for Sm3+, Eu3+, and Tb3+: X-ray Crystallography and Luminescence Properties
Source: Molecules. 2020 Jun 28;25(13):2971. doi: 10.3390/molecules25132971 (PMC7411983; doi:10.3390/molecules25132971)

# checkCIF/PLATON report

Structure factors have been supplied for datablock(s) gvsusb919a

THIS REPORT IS FOR GUIDANCE ONLY. IF USED AS PART OF A REVIEW PROCEDURE FOR PUBLICATION, IT SHOULD NOT REPLACE THE EXPERTISE OF AN EXPERIENCED CRYSTALLOGRAPHIC REFEREE.

No syntax errors found. CIF dictionary Interpreting this report

## Datablock: gvsusb919a

---

```
Bond precision:  C-C = 0.0134 A          Wavelength=0.71073

Cell:           a=11.438(2)       b=12.724(2)       c=14.681(3)
                alpha=91.784(2)   beta=106.593(2)   gamma=113.139(2)
Temperature:    173 K
```

| | Calculated | Reported |
|---|---|---|
| Volume | 1857.9(6) | 1857.8(6) |
| Space group | P -1 | P -1 |
| Hall group | -P 1 | -P 1 |
| Moiety formula | C60 H68 N6 O18 P4 Tb2, 4(N O3), 2(H2 O) | C60 H68 N6 O18 P4 Tb2, 4(N O3), 2(H2 O) |
| Sum formula | C60 H72 N10 O32 P4 Tb2 | C60 H72 N10 O32 P4 Tb2 |
| Mr | 1887.02 | 1886.99 |
| Dx,g cm-3 | 1.687 | 1.687 |
| Z | 1 | 1 |
| Mu (mm-1) | 2.067 | 2.068 |
| F000 | 948.0 | 948.0 |
| F000’ | 948.52 | |
| h,k,lmax | 13,15,17 | 13,15,17 |
| Nref | 6898 | 6825 |
| Tmin,Tmax | 0.761,0.924 | 0.529,0.745 |
| Tmin’ | 0.679 | |

```
Correction method= # Reported T Limits: Tmin=0.529 Tmax=0.745
AbsCorr = MULTI-SCAN

Data completeness= 0.989          Theta(max)= 25.475

R(reflections)= 0.0626( 5493)     wR2(reflections)= 0.1717( 6825)

S = 1.024                   Npar= 492
```

---

The following ALERTS were generated. Each ALERT has the format
**test-name_ALERT_alert-type_alert-level**.
Click on the hyperlinks for more details of the test.

---

## Alert level A

PLAT971_ALERT_2_A Check Calcd Resid. Dens. 0.96A From Tb1 3.76 eA-3

**Author Response: This residual electron density is near the metal.**

PLAT973_ALERT_2_A Check Calcd Positive Resid. Density on Tb1 2.52 eA-3

**Author Response: This positive residual density is near the metal.**

---

## Alert level B

PLAT971_ALERT_2_B Check Calcd Resid. Dens. 0.92A From Tb1 3.40 eA-3

**Author Response: This residual electron density is near the metal.**

PLAT971_ALERT_2_B Check Calcd Resid. Dens. 1.07A From Tb1 3.20 eA-3

**Author Response: This residual electron density is near the metal.**

PLAT971_ALERT_2_B Check Calcd Resid. Dens. 1.09A From Tb1 3.12 eA-3

**Author Response: This residual electron density is near the metal.**

PLAT971_ALERT_2_B Check Calcd Resid. Dens. 0.94A From Tb1 2.56 eA-3

**Author Response: This residual electron density is near the metal.**

---

## Alert level C

```
PLAT094_ALERT_2_C Ratio of Maximum / Minimum Residual Density ....       2.20 Report
PLAT241_ALERT_2_C High   MainMol Ueq as Compared to Neighbors of        C15 Check
PLAT244_ALERT_4_C Low    Solvent Ueq as Compared to Neighbors of         N4 Check
PLAT244_ALERT_4_C Low    Solvent Ueq as Compared to Neighbors of         N5 Check
PLAT260_ALERT_2_C Large Average Ueq of Residue Including       O1S      0.112 Check
PLAT342_ALERT_3_C Low Bond Precision on  C-C Bonds ...............    0.01341 Ang.
PLAT480_ALERT_4_C Long H...A H-Bond Reported H1       ..O12       .      2.70 Ang.
PLAT911_ALERT_3_C Missing FCF Refl Between Thmin & STh/L=    0.600         19 Report
PLAT971_ALERT_2_C Check Calcd Resid. Dens.  0.98A   From Tb1             2.37 eA-3
```

**Author Response: This residual electron density is near the metal.**

PLAT971_ALERT_2_C Check Calcd Resid. Dens. 1.94A From O1S 1.56 eA-3

**Author Response: This residual electron density is near the metal.**

```
PLAT972_ALERT_2_C Check Calcd Resid. Dens.  0.94A   From Tb1            -1.82 eA-3
PLAT972_ALERT_2_C Check Calcd Resid. Dens.  0.90A   From Tb1            -1.79 eA-3
PLAT975_ALERT_2_C Check Calcd Resid. Dens.  0.95A   From O1              1.00 eA-3
PLAT975_ALERT_2_C Check Calcd Resid. Dens.  0.91A   From O1              0.92 eA-3
PLAT975_ALERT_2_C Check Calcd Resid. Dens.  0.96A   From O15             0.88 eA-3
PLAT975_ALERT_2_C Check Calcd Resid. Dens.  0.95A   From O6              0.75 eA-3
PLAT975_ALERT_2_C Check Calcd Resid. Dens.  1.03A   From O1S             0.75 eA-3
PLAT976_ALERT_2_C Check Calcd Resid. Dens.  0.80A   From O1S            -0.75 eA-3
PLAT977_ALERT_2_C Check Negative Difference Density on H14B             -0.44 eA-3
PLAT977_ALERT_2_C Check Negative Difference Density on H15A             -0.33 eA-3
```

---

## Alert level G

```
PLAT007_ALERT_5_G Number of Unrefined Donor-H Atoms ..............          8 Report
PLAT072_ALERT_2_G SHELXL First  Parameter in WGHT  Unusually Large       0.12 Report
PLAT154_ALERT_1_G The s.u.'s on the Cell Angles are Equal ..(Note)      0.002 Degree
PLAT720_ALERT_4_G Number of Unusual/Non-Standard Labels ..........          2 Note
PLAT794_ALERT_5_G Tentative Bond Valency for Tb1       (III)     .       3.33 Info
PLAT910_ALERT_3_G Missing # of FCF Reflection(s) Below Theta(Min).          1 Note
PLAT912_ALERT_4_G Missing # of FCF Reflections Above STh/L=  0.600         54 Note
PLAT978_ALERT_2_G Number C-C Bonds with Positive Residual Density.          1 Info
```

---

2 **ALERT level A** = Most likely a serious problem - resolve or explain
4 **ALERT level B** = A potentially serious problem, consider carefully
20 **ALERT level C** = Check. Ensure it is not caused by an omission or oversight
8 **ALERT level G** = General information/check it is not something unexpected

1 ALERT type 1 CIF construction/syntax error, inconsistent or missing data
23 ALERT type 2 Indicator that the structure model may be wrong or deficient
3 ALERT type 3 Indicator that the structure quality may be low
5 ALERT type 4 Improvement, methodology, query or suggestion
2 ALERT type 5 Informative message, check

---

It is advisable to attempt to resolve as many as possible of the alerts in all categories. Often the minor alerts point to easily fixed oversights, errors and omissions in your CIF or refinement strategy, so attention to these fine details can be worthwhile. In order to resolve some of the more serious problems it may be necessary to carry out additional measurements or structure refinements. However, the purpose of your study may justify the reported deviations and the more serious of these should normally be commented upon in the discussion or experimental section of a paper or in the "special_details" fields of the CIF. checkCIF was carefully designed to identify outliers and unusual parameters, but every test has its limitations and alerts that are not important in a particular case may appear. Conversely, the absence of alerts does not guarantee there are no aspects of the results needing attention. It is up to the individual to critically assess their own results and, if necessary, seek expert advice.

**Publication of your CIF in IUCr journals**

A basic structural check has been run on your CIF. These basic checks will be run on all CIFs submitted for publication in IUCr journals (*Acta Crystallographica*, *Journal of Applied Crystallography*, *Journal of Synchrotron Radiation*); however, if you intend to submit to *Acta Crystallographica Section C* or *E* or *IUCrData*, you should make sure that full publication checks are run on the final version of your CIF prior to submission.

**Publication of your CIF in other journals**

Please refer to the *Notes for Authors* of the relevant journal for any special instructions relating to CIF submission.

---

**PLATON version of 22/12/2019; check.def file version of 13/12/2019**

**Datablock gvsusb919a** - ellipsoid plot

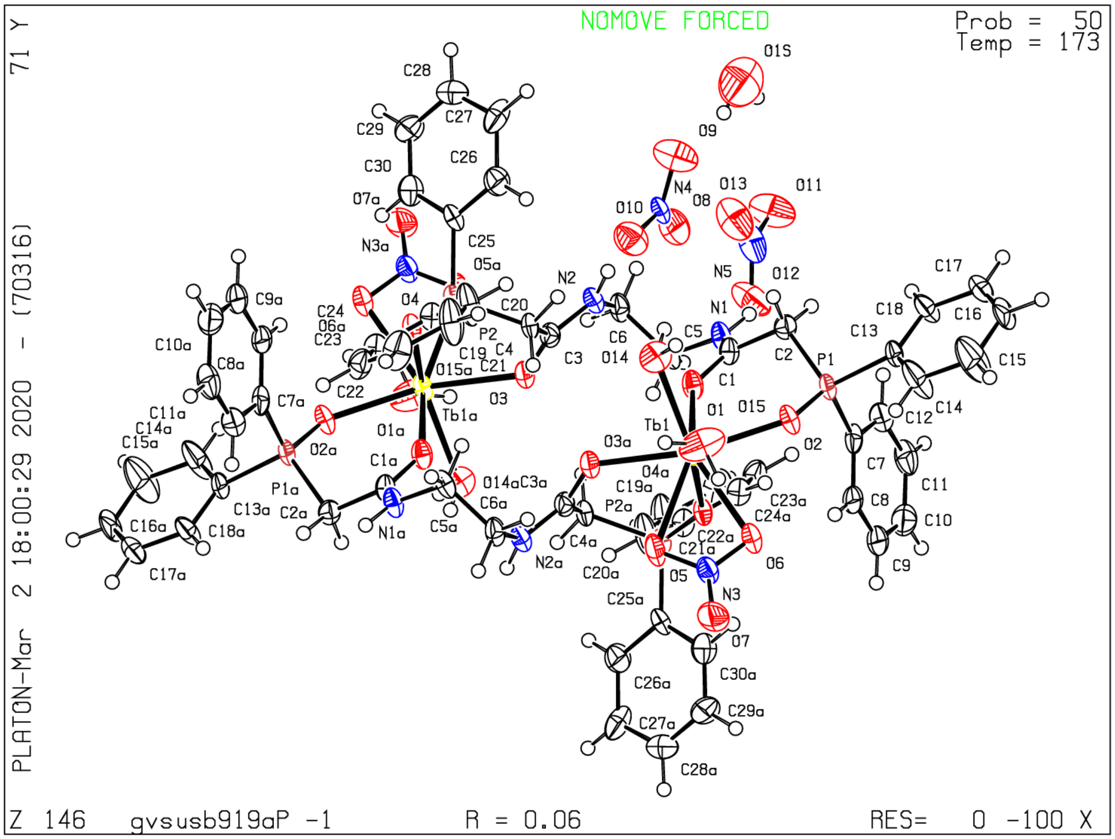

Supplement: Supplementary file 1 [file molecules-25-02971-s001.zip › supp_files_rev2/check_cif_reports/GVSUSB919A_checkcif.pdf]
