# Supplementary material for: Two Beta-Phosphorylamide Compounds as Ligands for Sm3+, Eu3+, and Tb3+: X-ray Crystallography and Luminescence Properties
Source: Molecules. 2020 Jun 28;25(13):2971. doi: 10.3390/molecules25132971 (PMC7411983; doi:10.3390/molecules25132971)

checkCIF/PLATON report

Structure factors have been supplied for datablock(s) gvs1014c_0m

THIS REPORT IS FOR GUIDANCE ONLY. IF USED AS PART OF A REVIEW PROCEDURE FOR PUBLICATION, IT SHOULD NOT REPLACE THE EXPERTISE OF AN EXPERIENCED CRYSTALLOGRAPHIC REFEREE.

No syntax errors found. CIF dictionary Interpreting this report

Datablock: gvs1014c_0m

```
Bond precision:  C-C = 0.0192 A          Wavelength=1.54178

Cell:           a=10.5235(15)     b=18.133(3)         c=19.488(3)
                alpha=90          beta=96.944(12)     gamma=90
Temperature:    173 K
```

	Calculated	Reported
Volume	3691.5(10)	3691.6(9)
Space group	P 21/n	P 1 21/n 1
Hall group	-P 2yn	-P 2yn
Moiety formula	C60 H60 N10 O26 P4 Sm2, 2(H2 O)	C60 H60 N10 O26 P4 Sm2, 2(H2 O)
Sum formula	C60 H64 N10 O28 P4 Sm2	C60 H64 N10 O28 P4 Sm2
Mr	1797.81	1797.79
Dx,g cm-3	1.617	1.617
Z	2	2
Mu (mm-1)	13.393	13.393
F000	1804.0	1804.0
F000’	1791.13	
h,k,lmax	12,22,23	12,22,23
Nref	7211	6996
Tmin,Tmax	0.165,0.430	0.486,0.753
Tmin’	0.024	

```
Correction method= # Reported T Limits: Tmin=0.486 Tmax=0.753
AbsCorr = MULTI-SCAN

Data completeness= 0.970          Theta(max)= 71.720

R(reflections)= 0.0722( 3911)     wR2(reflections)= 0.1815( 6996)

S = 1.020                  Npar= 472
```

The following ALERTS were generated. Each ALERT has the format
test-name_ALERT_alert-type_alert-level.
Click on the hyperlinks for more details of the test.

Alert level B

```
PLAT241_ALERT_2_B High   'MainMol' Ueq as Compared to Neighbors of         O9 Check
```

Author Response: This atom assignment was confirmed as an oxygen (of a nitrate group). O9 is bonded directly to the Sm metal center (which has a relatively small U(eq) value). This difference is likely what triggered this alert.

Alert level C

```
RINTA01_ALERT_3_C  The value of Rint is greater than 0.12
            Rint given   0.146
PLAT020_ALERT_3_C The Value of Rint is Greater Than 0.12 .........      0.146 Report
PLAT220_ALERT_2_C Non-Solvent  Resd 1  C   Ueq(max)/Ueq(min) Range        3.6 Ratio
PLAT234_ALERT_4_C Large Hirshfeld Difference Sm1      --O12      .       0.16 Ang.
PLAT234_ALERT_4_C Large Hirshfeld Difference P1       --C11      .       0.16 Ang.
PLAT234_ALERT_4_C Large Hirshfeld Difference C11      --C12      .       0.16 Ang.
PLAT234_ALERT_4_C Large Hirshfeld Difference C11      --C16      .       0.16 Ang.
PLAT234_ALERT_4_C Large Hirshfeld Difference C14      --C15      .       0.21 Ang.
PLAT241_ALERT_2_C High   'MainMol' Ueq as Compared to Neighbors of         O5 Check
```

Author Response: This atom assignment was confirmed as an oxygen (of a nitrate group). O9 is bonded directly to the Sm metal center (which has a relatively small U(eq) value). This difference is likely what triggered this alert.

```
PLAT241_ALERT_2_C High   'MainMol' Ueq as Compared to Neighbors of         O6 Check
```

Author Response: This atom assignment was confirmed as an oxygen (of a nitrate group). O9 is bonded directly to the Sm metal center (which has a relatively small U(eq) value). This difference is likely what triggered this alert.

```
PLAT241_ALERT_2_C High   'MainMol' Ueq as Compared to Neighbors of        O12 Check
```

Author Response: This atom assignment was confirmed as an oxygen (of a nitrate group). O9 is bonded directly to the Sm metal center (which has a relatively small U(eq) value). This difference is likely what triggered this alert.

```
PLAT241_ALERT_2_C High   'MainMol' Ueq as Compared to Neighbors of         C9 Check
```

Author Response: This atom assignment was confirmed as an oxygen (of a nitrate group). O9 is bonded directly to the Sm metal center (which has a relatively small U(eq) value). This difference is likely what triggered this alert.

```
PLAT241_ALERT_2_C High   'MainMol' Ueq as Compared to Neighbors of        C18 Check
```

Author Response: This atom assignment was confirmed as an oxygen (of a nitrate group). O9 is bonded directly to the Sm metal center (which has a relatively small U(eq) value). This difference is likely what triggered this alert.

```
PLAT241_ALERT_2_C High   'MainMol' Ueq as Compared to Neighbors of        C28 Check
```

Author Response: This atom assignment was confirmed as an oxygen (of a nitrate group). O9 is bonded directly to the Sm metal center (which has a relatively small U(eq) value). This difference is likely what triggered this alert.

```
PLAT241_ALERT_2_C High   'MainMol' Ueq as Compared to Neighbors of        C30 Check
```

Author Response: This atom assignment was confirmed as an oxygen (of a nitrate group). O9 is bonded directly to the Sm metal center (which has a relatively small U(eq) value). This difference is likely what triggered this alert.

```
PLAT242_ALERT_2_C Low    'MainMol' Ueq as Compared to Neighbors of        Sm1 Check
PLAT242_ALERT_2_C Low    'MainMol' Ueq as Compared to Neighbors of         N3 Check
PLAT242_ALERT_2_C Low    'MainMol' Ueq as Compared to Neighbors of         N4 Check
PLAT242_ALERT_2_C Low    'MainMol' Ueq as Compared to Neighbors of        C25 Check
PLAT260_ALERT_2_C Large Average Ueq of Residue Including       O14      0.109 Check
PLAT331_ALERT_2_C Small Aver Phenyl C-C Dist C5        -C10      .       1.37 Ang.
PLAT342_ALERT_3_C Low Bond Precision on  C-C Bonds ..............    0.01919 Ang.
PLAT906_ALERT_3_C Large K Value in the Analysis of Variance ......      5.488 Check
PLAT911_ALERT_3_C Missing FCF Refl Between Thmin & STh/L=    0.600         91 Report
PLAT934_ALERT_3_C Number of (Iobs-Icalc)/Sigma(W) > 10 Outliers ..          1 Check
PLAT975_ALERT_2_C Check Calcd Resid. Dens.  0.83A   From O1              0.45 eA-3
PLAT978_ALERT_2_C Number C-C Bonds with Positive Residual Density.          0 Info
```

Alert level G

```
PLAT003_ALERT_2_G Number of Uiso or Uij Restrained non-H Atoms ...          6 Report
PLAT007_ALERT_5_G Number of Unrefined Donor-H Atoms .............           4 Report
PLAT177_ALERT_4_G The CIF-Embedded .res File Contains DELU Records          1 Report
PLAT178_ALERT_4_G The CIF-Embedded .res File Contains SIMU Records          1 Report
PLAT232_ALERT_2_G Hirshfeld Test Diff (M-X)  Sm1      --O5       .        5.6 s.u.
PLAT793_ALERT_4_G Model has Chirality at P2          (Centro SPGR)          R Verify
PLAT794_ALERT_5_G Tentative Bond Valency for Sm1       (III)     .       3.46 Info
PLAT860_ALERT_3_G Number of Least-Squares Restraints ............          48 Note
PLAT912_ALERT_4_G Missing # of FCF Reflections Above STh/L=  0.600        125 Note
```

---

0 **ALERT level A** = Most likely a serious problem - resolve or explain
1 **ALERT level B** = A potentially serious problem, consider carefully

```
27 ALERT level C = Check. Ensure it is not caused by an omission or oversight
 9 ALERT level G = General information/check it is not something unexpected

 0 ALERT type 1 CIF construction/syntax error, inconsistent or missing data
19 ALERT type 2 Indicator that the structure model may be wrong or deficient
 7 ALERT type 3 Indicator that the structure quality may be low
 9 ALERT type 4 Improvement, methodology, query or suggestion
 2 ALERT type 5 Informative message, check
```

It is advisable to attempt to resolve as many as possible of the alerts in all categories. Often the minor alerts point to easily fixed oversights, errors and omissions in your CIF or refinement strategy, so attention to these fine details can be worthwhile. In order to resolve some of the more serious problems it may be necessary to carry out additional measurements or structure refinements. However, the purpose of your study may justify the reported deviations and the more serious of these should normally be commented upon in the discussion or experimental section of a paper or in the "special_details" fields of the CIF. checkCIF was carefully designed to identify outliers and unusual parameters, but every test has its limitations and alerts that are not important in a particular case may appear. Conversely, the absence of alerts does not guarantee there are no aspects of the results needing attention. It is up to the individual to critically assess their own results and, if necessary, seek expert advice.

Publication of your CIF in IUCr journals

A basic structural check has been run on your CIF. These basic checks will be run on all CIFs submitted for publication in IUCr journals (*Acta Crystallographica*, *Journal of Applied Crystallography*, *Journal of Synchrotron Radiation*); however, if you intend to submit to *Acta Crystallographica Section C* or *E* or *IUCrData*, you should make sure that full publication checks are run on the final version of your CIF prior to submission.

Publication of your CIF in other journals

Please refer to the *Notes for Authors* of the relevant journal for any special instructions relating to CIF submission.

PLATON version of 07/08/2019; check.def file version of 30/07/2019

Datablock gvs1014c_0m - ellipsoid plot

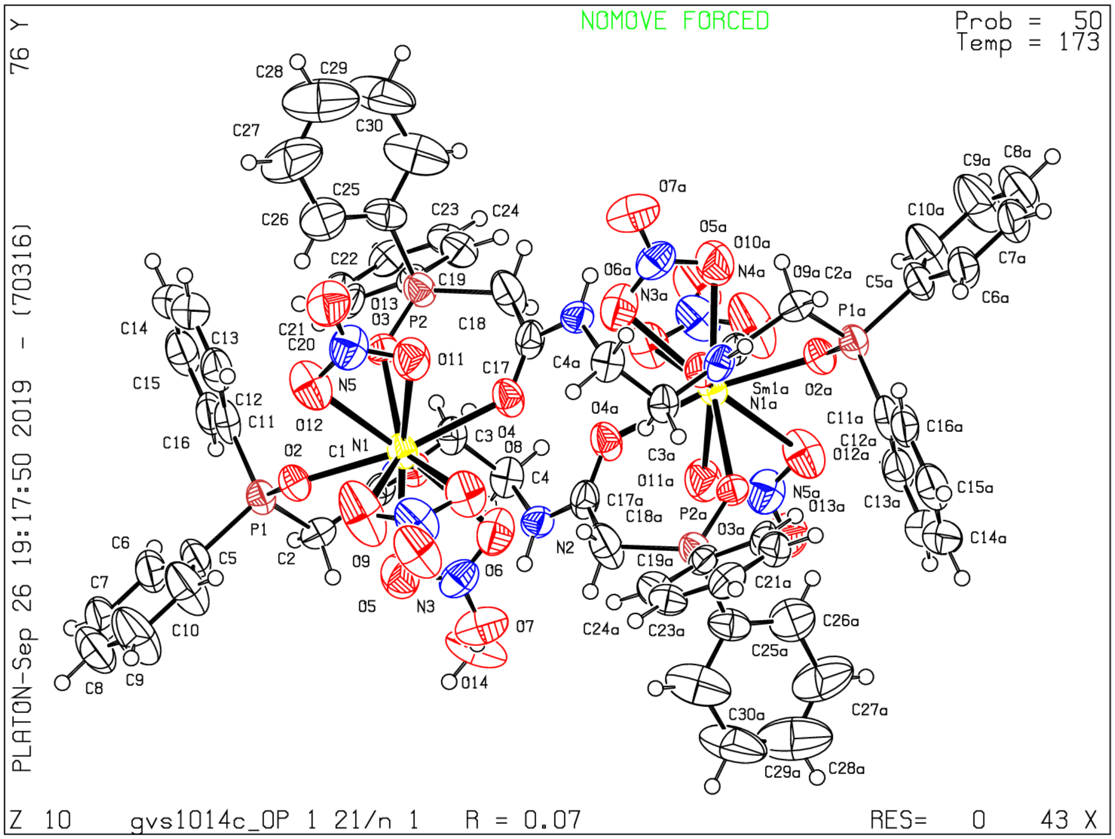

Supplement: Supplementary file 1 [file molecules-25-02971-s001.zip › supp_files_rev2/check_cif_reports/GVS1014c_checkcif.pdf]
