# Supplementary material for: Two Beta-Phosphorylamide Compounds as Ligands for Sm3+, Eu3+, and Tb3+: X-ray Crystallography and Luminescence Properties
Source: Molecules. 2020 Jun 28;25(13):2971. doi: 10.3390/molecules25132971 (PMC7411983; doi:10.3390/molecules25132971)

checkCIF/PLATON report

Structure factors have been supplied for datablock(s) gvsu819k

THIS REPORT IS FOR GUIDANCE ONLY. IF USED AS PART OF A REVIEW PROCEDURE FOR PUBLICATION, IT SHOULD NOT REPLACE THE EXPERTISE OF AN EXPERIENCED CRYSTALLOGRAPHIC REFEREE.

No syntax errors found. CIF dictionary Interpreting this report

Datablock: gvsu819k

```
Bond precision:  C-C = 0.0046 A          Wavelength=1.54178

Cell:            a=5.6555(1)     b=28.8994(5)        c=8.4517(2)
                 alpha=90        beta=109.429(1)     gamma=90
Temperature:     173 K

                Calculated                Reported
Volume          1302.69(5)                1302.69(5)
Space group     P 21/c                    P 1 21/c 1
Hall group      -P 2ybc                   -P 2ybc
Moiety formula  C30 H30 N2 O4 P2          C30 H30 N2 O4 P2
Sum formula     C30 H30 N2 O4 P2          C30 H30 N2 O4 P2
Mr              544.50                    544.50
Dx,g cm-3       1.388                     1.388
Z               2                         2
Mu (mm-1)       1.848                     1.848
F000            572.0                     572.0
F000'           574.71
h,k,lmax        6,34,10                   6,34,10
Nref            2390                      2388
Tmin,Tmax       0.814,0.937               0.635,0.753
Tmin'           0.732

Correction method= # Reported T Limits: Tmin=0.635 Tmax=0.753
AbsCorr = MULTI-SCAN

Data completeness= 0.999          Theta(max)= 68.305

R(reflections)= 0.0468( 1948)     wR2(reflections)= 0.1234( 2388)

S = 1.022                  Npar= 213
```

The following ALERTS were generated. Each ALERT has the format
test-name_ALERT_alert-type_alert-level.
Click on the hyperlinks for more details of the test.

Alert level C

```
PLAT241_ALERT_2_C High   'MainMol' Ueq as Compared to Neighbors of          C5 Check
PLAT242_ALERT_2_C Low    'MainMol' Ueq as Compared to Neighbors of          C4 Check
PLAT242_ALERT_2_C Low    'MainMol' Ueq as Compared to Neighbors of          C7 Check
PLAT332_ALERT_2_C Large Phenyl C-C Range     C10       -C15A     .       0.19 Ang.
PLAT340_ALERT_3_C Low Bond Precision on  C-C Bonds ...............    0.00462 Ang.
```

Alert level G

```
PLAT301_ALERT_3_G Main Residue  Disorder ..............(Resd  1  )        21% Note
PLAT410_ALERT_2_G Short Intra H...H Contact  H1B      ..H15      .       2.09 Ang.
                                                       x,y,z  =      1_555 Check
PLAT410_ALERT_2_G Short Intra H...H Contact  H9       ..H15A     .       2.05 Ang.
                                                       x,y,z  =      1_555 Check
PLAT793_ALERT_4_G Model has Chirality at P1          (Centro SPGR)          R Verify
PLAT912_ALERT_4_G Missing # of FCF Reflections Above STh/L=  0.600          2 Note
PLAT978_ALERT_2_G Number C-C Bonds with Positive Residual Density.          9 Info
```

```
   0 ALERT level A = Most likely a serious problem - resolve or explain
   0 ALERT level B = A potentially serious problem, consider carefully
   5 ALERT level C = Check. Ensure it is not caused by an omission or oversight
   6 ALERT level G = General information/check it is not something unexpected

   0 ALERT type 1 CIF construction/syntax error, inconsistent or missing data
   7 ALERT type 2 Indicator that the structure model may be wrong or deficient
   2 ALERT type 3 Indicator that the structure quality may be low
   2 ALERT type 4 Improvement, methodology, query or suggestion
   0 ALERT type 5 Informative message, check
```

It is advisable to attempt to resolve as many as possible of the alerts in all categories. Often the minor alerts point to easily fixed oversights, errors and omissions in your CIF or refinement strategy, so attention to these fine details can be worthwhile. In order to resolve some of the more serious problems it may be necessary to carry out additional measurements or structure refinements. However, the purpose of your study may justify the reported deviations and the more serious of these should normally be commented upon in the discussion or experimental section of a paper or in the "special_details" fields of the CIF. checkCIF was carefully designed to identify outliers and unusual parameters, but every test has its limitations and alerts that are not important in a particular case may appear. Conversely, the absence of alerts does not guarantee there are no aspects of the results needing attention. It is up to the individual to critically assess their own results and, if necessary, seek expert advice.

Publication of your CIF in IUCr journals

A basic structural check has been run on your CIF. These basic checks will be run on all CIFs submitted for publication in IUCr journals (*Acta Crystallographica*, *Journal of Applied Crystallography*, *Journal of Synchrotron Radiation*); however, if you intend to submit to *Acta Crystallographica Section C* or *E* or *IUCrData*, you should make sure that full publication checks are run on the final version of your CIF prior to submission.

Publication of your CIF in other journals

Please refer to the *Notes for Authors* of the relevant journal for any special instructions relating to CIF submission.

PLATON version of 07/08/2019; check.def file version of 30/07/2019

Datablock gvsu819k - ellipsoid plot

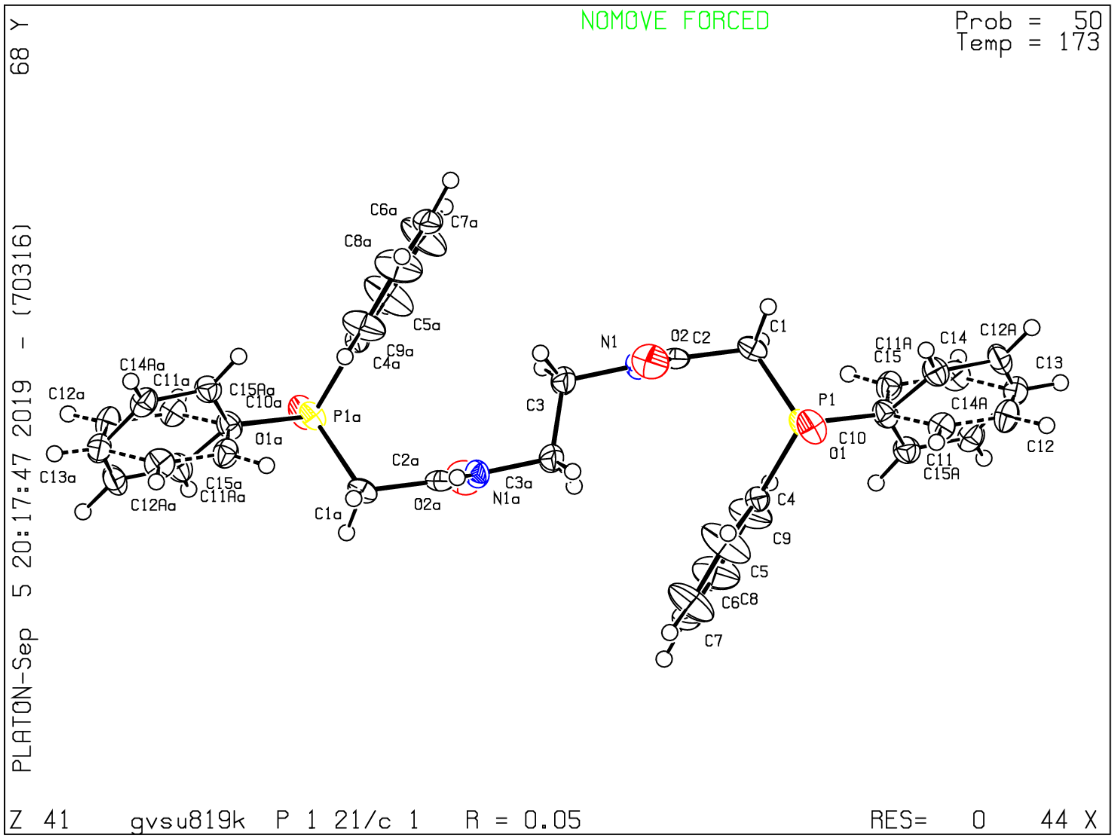

Supplement: Supplementary file 1 [file molecules-25-02971-s001.zip › supp_files_rev2/check_cif_reports/GVSU819K_checkcif.pdf]
