# Supplementary material for: Two Beta-Phosphorylamide Compounds as Ligands for Sm3+, Eu3+, and Tb3+: X-ray Crystallography and Luminescence Properties
Source: Molecules. 2020 Jun 28;25(13):2971. doi: 10.3390/molecules25132971 (PMC7411983; doi:10.3390/molecules25132971)

checkCIF/PLATON report

Structure factors have been supplied for datablock(s) gvsu819l

THIS REPORT IS FOR GUIDANCE ONLY. IF USED AS PART OF A REVIEW PROCEDURE FOR PUBLICATION, IT SHOULD NOT REPLACE THE EXPERTISE OF AN EXPERIENCED CRYSTALLOGRAPHIC REFEREE.

No syntax errors found. CIF dictionary Interpreting this report

Datablock: gvsu819l

Bond precision: C-C = 0.0063 A Wavelength=1.54178

Cell:	a=32.3189(5)	b=12.5255(2)	c=16.4158(3)
	alpha=90	beta=116.162(1)	gamma=90
Temperature:	173 K		

	Calculated	Reported
Volume	5964.48(18)	5964.48(18)
Space group	C 2/c	C 1 2/c 1
Hall group	-C 2yc	-C 2yc
Moiety formula	C28 H60 N10 O34 P4 Tb2, 2(C2 H3 N)	C28 H60 N10 O34 P4 Tb2, 2(C2 H3 N)
Sum formula	C32 H66 N12 O34 P4 Tb2	C32 H66 N12 O34 P4 Tb2
Mr	1604.71	1604.68
Dx,g cm-3	1.787	1.787
Z	4	4
Mu (mm-1)	13.430	13.430
F000	3216.0	3216.0
F000’	3158.54	
h,k,lmax	39,15,20	39,15,19
Nref	5677	5582
Tmin,Tmax	0.117,0.251	0.119,0.400
Tmin’	0.015	

Correction method= # Reported T Limits: Tmin=0.119 Tmax=0.400
AbsCorr = MULTI-SCAN

Data completeness= 0.983 Theta(max)= 70.187

R(reflections)= 0.0287(5026) wR2(reflections)= 0.0653(5582)

S = 1.086 Npar= 402

The following ALERTS were generated. Each ALERT has the format
      test-name_ALERT_alert-type_alert-level.
Click on the hyperlinks for more details of the test.

Alert level C

```
PLAT220_ALERT_2_C Non-Solvent  Resd 1  C   Ueq(max)/Ueq(min) Range        3.5 Ratio
PLAT222_ALERT_3_C Non-Solv.  Resd 1  H   Uiso(max)/Uiso(min) Range        6.2 Ratio
PLAT241_ALERT_2_C High   'MainMol' Ueq as Compared to Neighbors of        C10 Check
PLAT911_ALERT_3_C Missing FCF Refl Between Thmin & STh/L=    0.600         79 Report
```

Alert level G

```
PLAT012_ALERT_1_G No       _shelx_res_checksum Found in CIF ......     Please Check
PLAT083_ALERT_2_G SHELXL Second Parameter in WGHT  Unusually Large      25.88 Why ?
PLAT128_ALERT_4_G Alternate Setting for Input Space Group     C2/c       I2/a Note
PLAT232_ALERT_2_G Hirshfeld Test Diff (M-X)  Tb1      --O9       .        9.6 s.u.
PLAT232_ALERT_2_G Hirshfeld Test Diff (M-X)  Tb1      --O10      .        7.8 s.u.
PLAT232_ALERT_2_G Hirshfeld Test Diff (M-X)  Tb1      --O12      .       13.9 s.u.
PLAT232_ALERT_2_G Hirshfeld Test Diff (M-X)  Tb1      --O13      .        5.9 s.u.
PLAT232_ALERT_2_G Hirshfeld Test Diff (M-X)  Tb1      --O15      .        6.0 s.u.
PLAT232_ALERT_2_G Hirshfeld Test Diff (M-X)  Tb1      --O16      .        8.5 s.u.
PLAT301_ALERT_3_G Main Residue  Disorder ..............(Resd  1  )         3% Note
PLAT720_ALERT_4_G Number of Unusual/Non-Standard Labels ..........          5 Note
PLAT793_ALERT_4_G Model has Chirality at P1          (Centro SPGR)          S Verify
PLAT794_ALERT_5_G Tentative Bond Valency for Tb1       (III)     .       3.00 Info
PLAT912_ALERT_4_G Missing # of FCF Reflections Above STh/L=  0.600         15 Note
PLAT978_ALERT_2_G Number C-C Bonds with Positive Residual Density.          1 Info
```

---

   0 **ALERT level A** = Most likely a serious problem - resolve or explain
   0 **ALERT level B** = A potentially serious problem, consider carefully
   4 **ALERT level C** = Check. Ensure it is not caused by an omission or oversight
  15 **ALERT level G** = General information/check it is not something unexpected

```
 1 ALERT type 1 CIF construction/syntax error, inconsistent or missing data
10 ALERT type 2 Indicator that the structure model may be wrong or deficient
 3 ALERT type 3 Indicator that the structure quality may be low
 4 ALERT type 4 Improvement, methodology, query or suggestion
 1 ALERT type 5 Informative message, check
```

It is advisable to attempt to resolve as many as possible of the alerts in all categories. Often the minor alerts point to easily fixed oversights, errors and omissions in your CIF or refinement strategy, so attention to these fine details can be worthwhile. In order to resolve some of the more serious problems it may be necessary to carry out additional measurements or structure refinements. However, the purpose of your study may justify the reported deviations and the more serious of these should normally be commented upon in the discussion or experimental section of a paper or in the "special_details" fields of the CIF. checkCIF was carefully designed to identify outliers and unusual parameters, but every test has its limitations and alerts that are not important in a particular case may appear. Conversely, the absence of alerts does not guarantee there are no aspects of the results needing attention. It is up to the individual to critically assess their own results and, if necessary, seek expert advice.

Publication of your CIF in IUCr journals

A basic structural check has been run on your CIF. These basic checks will be run on all CIFs submitted for publication in IUCr journals (*Acta Crystallographica*, *Journal of Applied Crystallography*, *Journal of Synchrotron Radiation*); however, if you intend to submit to *Acta Crystallographica Section C* or *E* or *IUCrData*, you should make sure that full publication checks are run on the final version of your CIF prior to submission.

Publication of your CIF in other journals

Please refer to the *Notes for Authors* of the relevant journal for any special instructions relating to CIF submission.

PLATON version of 07/08/2019; check.def file version of 30/07/2019

Datablock gvsu819l - ellipsoid plot

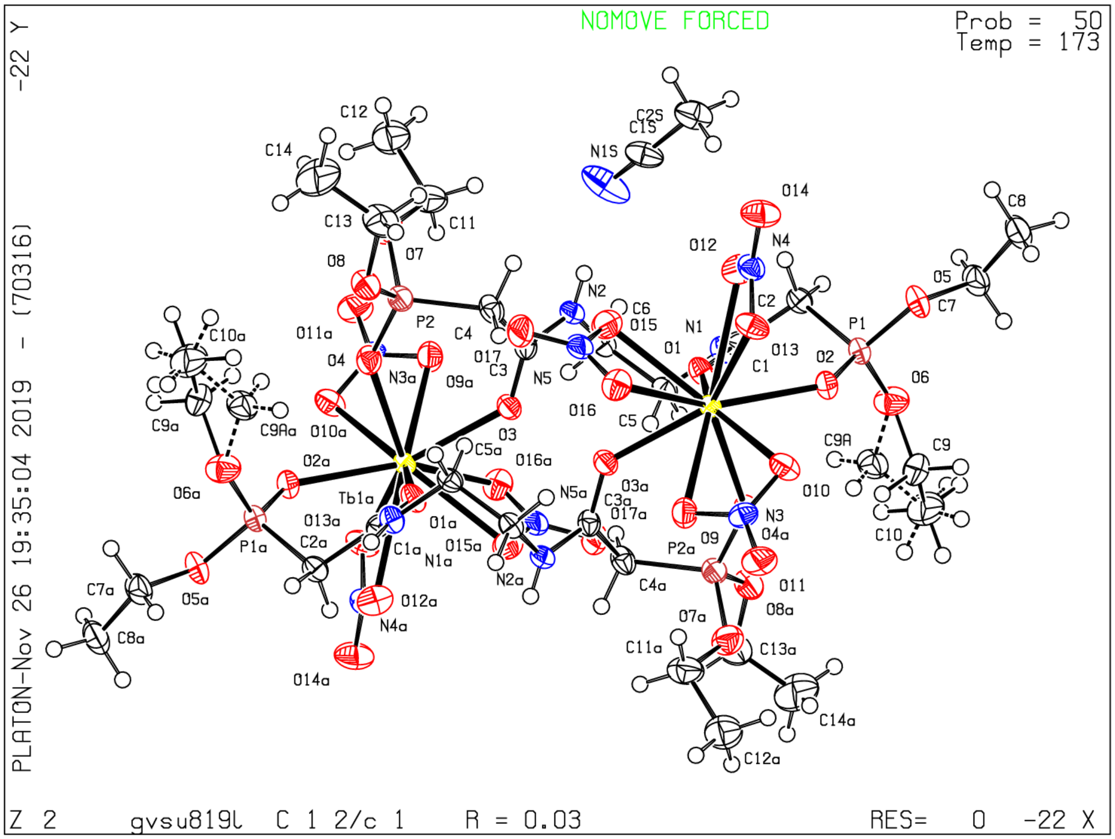

Supplement: Supplementary file 1 [file molecules-25-02971-s001.zip › supp_files_rev2/check_cif_reports/GVSU819L_checkcif.pdf]
