# Supplementary material for: Two Beta-Phosphorylamide Compounds as Ligands for Sm3+, Eu3+, and Tb3+: X-ray Crystallography and Luminescence Properties
Source: Molecules. 2020 Jun 28;25(13):2971. doi: 10.3390/molecules25132971 (PMC7411983; doi:10.3390/molecules25132971)
Supplement: Supplementary file 1 [file molecules-25-02971-s001.zip › supp_files_rev2/Lear_Molecules_rev2.docx]

Article

Two beta-phosphorylamide compounds as ligands for Sm^3+^, Eu^3+^ and Tb^3+^: X-ray crystallography and luminescence properties

Alan R. Lear^1^, Jonah Lenters^1^, Michael G. Patterson^2^, Richard J. Staples^3^, Eric J. Werner^2^, and Shannon M. Biros^1^*

^1^ Department of Chemistry, Grand Valley State University, 1 Campus Dr., Allendale, MI 49401, USA

^2^ Department of Chemistry, Biochemistry and Physics, The University of Tampa, 401 W. Kennedy Blvd., Tampa, FL 33606, USA

^3^ Center for Crystallographic Research, Department of Chemistry, Michigan State University, 578 S. Shaw Lane, East Lansing, MI 48824, USA

***** Correspondence: biross@gvsu.edu; Tel.: +1-616-331-8955

Received: date; Accepted: date; Published: date

**Abstract:** This paper describes the synthesis of two beta-phosphorylamide ligands and their coordination chemistry with the Ln ions Tb^3+^, Eu^3+^ and Sm^3+^. Both the ligands and Ln-complexes were characterized by IR, NMR, MS and X-Ray crystallography. The luminescence properties of the Tb^3+^- and Eu^3+^-complexes were also characterized, including the acquisition of lifetime decay curves. In the solid state, the Tb^3+^- and Sm^3+^-ligand complexes were found to have a 2:2 stoichiometry when analyzed by X-Ray diffraction. In these structures, the Ln ion was bound by both oxygen atoms of each beta-phosphorylamide moiety of the ligands. The Tb^3+^- and Eu^3+^-complexes were modestly emissive as solutions in acetonitrile, with lifetime values that fell within typical ranges.

**Keywords:** X-Ray crystallography; lanthanide luminescence; carbamoylmethylphosphine oxide (CMPO); lanthanide coordination chemistry

1. Introduction

Studies into the coordination chemistry of lanthanide (Ln) metals have gained attention due to their unique chemical and photophysical properties. The vast majority of Ln metals exhibit a stable +3 oxidation state and have coordination numbers that range from 5-10, with values of 8 and 9 being most common.[1] Since the Ln ions’ ­*f*-orbitals are minimally used for metal-ligand binding, the coordination geometries are varied and often dictated by the identity of the ligands.

Most Ln metals also emit light in the ultraviolet, visible, and near-infrared regions of the electromagnetic spectrum. This “lanthanide luminescence” is most often facilitated by the incorporation of an organic molecule that binds to the metal and acts as an antenna to harvest incident light[2-6]. As such, Ln-ligand complexes have found use in a wide variety of applications such as computer screens, probes[7-9], sensors[10, 11], imaging agents[12, 13], magnets[14], and other functional materials[15, 16]. Current efforts in the field of *f*-element coordination chemistry are focused on the development of new ligands that can bind these metals in both solution and in the solid-state and be useful as new materials, or in efforts to purify Ln ions from recycled materials[17, 18] and ground ores[19-21].

This paper describes the synthesis and characterization of two new organic ligands, along with their complexes with the Ln ions Sm^3+^, Tb^3+^, and Eu^3+^. One of these ligands contains the carbamoylmethylphosphine oxide (CMPO) chelating group, which was initially developed as a part of organic compounds used in the sequestration of transuranic elements from spent nuclear fuel[22]. The other ligand contains a similar chelating group, with a phosphate ester in place of the phosphine oxide. We also discuss here the characterization of the Ln-complexes of these ligands using IR and X-Ray crystallography, as well as the ability of these complexes to exhibit lanthanide luminescence.

2. Results and Discussion

2.1. Synthesis of the bis-CMPO ligands **1** and **2**, and the 1:1 Ln-complexes

Both organic ligands were synthesized following well-trodden paths (Figure 1), and were isolated in reasonable yields. The ethoxy-substituted ligand **1** was prepared in one step by direct condensation of ethylenediamine **3** and triethyl phosphonoacetate **4** at high concentration in methanol[23]. Analogously, the phenyl-substituted ligand **2** was synthesized by combining ethylenediamine and the p-nitrophenol ester **5**[24]. The Ln(NO_3_)_3_-ligand complexes were prepared by stirring a 1:1 ratio of lanthanide nitrate hydrate (Ln = Sm, Eu, Tb) and the ligand in acetonitrile. The complexes were purified by removal of the volatiles and trituration of the resultant solid film with diethyl ether to give beige powders. Scheme 1 depicts the stoichiometry of the product Ln-ligand complexes as 2:2 based on X-Ray structural data (vide infra).


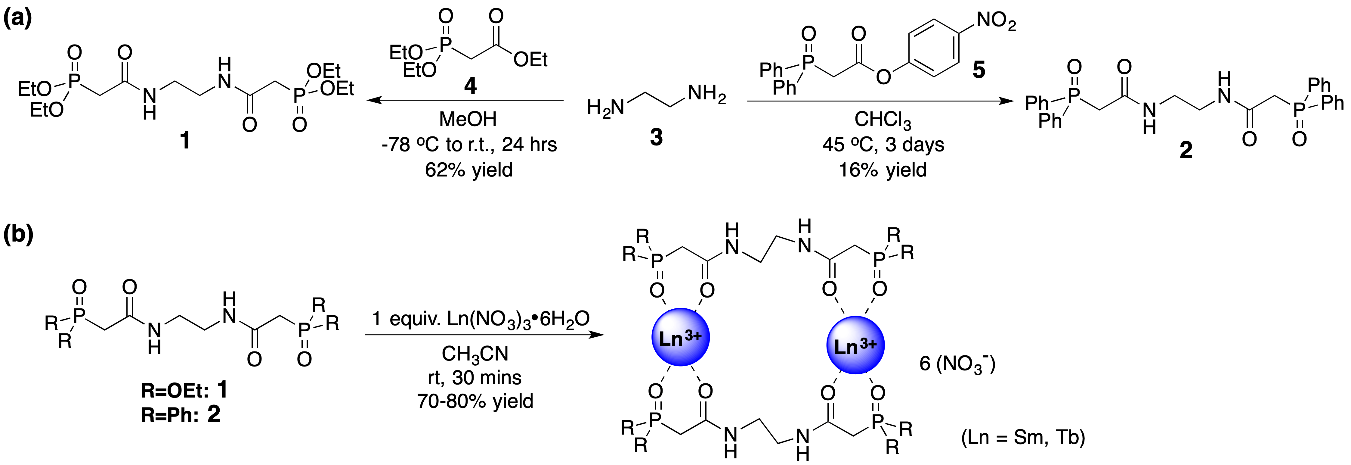


**Figure 1.** Synthetic pathways to (a) ligands **1** and **2**, along with (b) their 2:2 Ln-ligand complexes. The Ln-ligand complex shown in part (b) has been simplified for clarity; details regarding the precise coordination environments, including metal-bound nitrate groups and solvent molecues, are described with the X-Ray diffraction studies.

2.2 Characterization of the ligands and complexes in the solid state - IR spectra and single crystal X-Ray diffraction studies

Both the ligands and their Ln-complexes were characterized in the solid state by IR spectroscopy and single crystal X-Ray diffraction. IR spectroscopy revealed shifts in both the ligand C=O and P=O stretches to smaller wavenumbers for the Ln-complexes of both ligands (Δν ≈ 40 cm^-1^ and 15 cm^-1^, respectively; Table 1). This suggests that the oxygen atoms of both the carbonyl and phosphonate/phosphine oxide groups are coordinated to the metal in the solid state. For the Ln complexes of the ethoxy-substituted ligand, the frequency of the P-O stretch did not shift significantly compared to the free ligand indicating that it is not involved in metal binding.

**Table 1.** FT-IR data of ligands **1** and the resultant Ln-complexes; peak values reported in wavenumbers (cm^-1^).

| **stretch** | **Ligand** **1** | **Sm-1** | **Eu-1** | **Tb-1** | **Ligand 2** | **Sm-2** | **Eu-2** | **Tb-2** |
| --- | --- | --- | --- | --- | --- | --- | --- | --- |
| C=O | 1666 | 1626 | 1627 | 1627 | 1661 | 1623 | 1621 | 1621 |
| P=O | 1203 | 1191 | 1191 | 1191 | 1173 | 1157 | 1156 | 1156 |
| P-O | 1016 | 1014 | 1013 | 1014 | ----- | ----- | ----- | ----- |
| N-H | 3268 | 3317 | 3328 | 3321 | 3271 | weak | weak | weak |

Single crystals of both ligands **1**[25] and **2** have been isolated and analyzed by X-Ray diffraction (Figure 2). For the structure of compound **2**, the electron density corresponding to one of the phenyl rings was disordered and modeled as two orientations of the ring. Additionally, crystals of the Sm^3+^ and Tb^3+^ complexes of both ligands were also analyzed. Unfortunately, despite our best efforts, crystals of the Eu^3+^ complexes of either ligand were not isolated. Crystal data and structure refinement information for all new structures reported here are given in Table 2, and pertinent bond lengths and angles for ligands **1** and **2**, as well as their Sm(NO_3_)_3_ and Tb(NO_3_)_3_-complexes, are given in Tables 3 and 4. Additional structural and experimental details regarding all new crystal structures, along with figures depicting the thermal ellipsoids of every non-hydrogen atom, can be found in the Supplementary Materials.


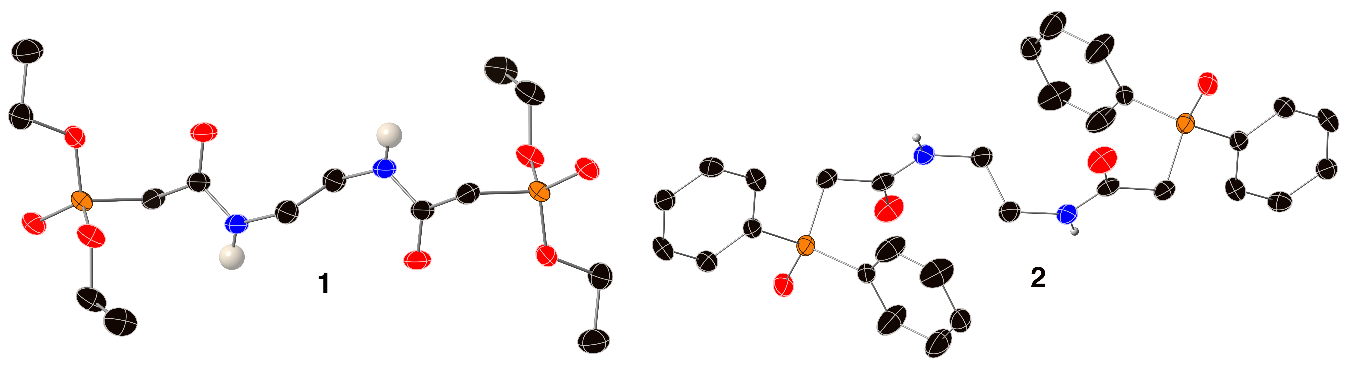


**Figure 2.** X-ray crystal structures of organic ligands **1**[25] and **2**. Thermal ellipsoids are depicted at the 50% probability level using standard CPK colors, and all hydrogen atoms bonded to carbon atoms have been omitted for clarity. Only the major component of compound **2** is shown here; details regarding the treatment of this disorder are given in the Supplementary Materials.

**Table 2.** Crystal data and structure refinement for free ligand **2**, and the metal complexes [Sm(NO_3_)_3_(**1**)]_2_•(CH_3_CN), [Tb(NO_3_)_3_(**1**)]_2_•(CH_3_CN), [Sm(NO_3_)_3_(**2)]_2_**•H_2_O, and [Tb(NO_3_)_3_(**2)**(H_2_O)_2_]_2_, [Tb(NO_3_)_3_(**2)**(MeOH)]_2_.

| **Structure number** | **2** | [Sm(NO_3_)_3_(**1**)]_2_• (CH_3_CN) | [Tb(NO_3_)_3_(**1**)]_2_•(CH_3_CN) | [Sm(NO_3_)_3_(**2**)]_2_•H_2_O | [Tb(NO_3_)_3_(**2)**(H_2_O)_2_]_2_ | [Tb(NO_3_)_3_(**2)**(MeOH)]**_2_** |
| --- | --- | --- | --- | --- | --- | --- |
| CCDC number | 2003372 | 2003370 | 2003373 | 2003369 | 2003374 | 2003371 |
| Empirical formula | C_30_H_30_N_2_O_4_P_2_ | C_32_H_66_N_12_O_34_P_4_Sm_2_ | C_32_H_66_N_12_O_34_P_4_Tb_2_ | C_60_H_64_N_10_O_28_P_4_Sm_2_ | C_60_H_72_N_10_O_32_P_4_Tb_2_ | C_64_H_76_N_10_O_30_P_4_Tb_2_ |
| Formula weight | 544.50 | 1587.54 | 1604.68 | 1797.79 | 1886.99 | 1907.06 |
| Temperature/K | 173(2) | 173(2) | 173(2) | 173(2) | 173(2) | 173(2) |
| Crystal system | monoclinic | monoclinic | monoclinic | monoclinic | triclinic | tetragonal |
| Space group | P2_1_/c | C2/c | C2/c | P2_1_/n | P-1 | P-42_1_c |
| a/Å | 5.65550(10) | 32.318(11) | 32.3189(5) | 10.5235(15) | 11.438(2) | 18.2898(4) |
| b/Å | 28.8994(5) | 12.538(4) | 12.5255(2) | 18.133(3) | 12.724(2) | 18.2898(4) |
| c/Å | 8.4517(2) | 16.447(6) | 16.4158(3) | 19.488(3) | 14.681(3) | 27.4073(6) |
| α/° | 90 | 90 | 90 | 90 | 91.784(2) | 90 |
| β/° | 109.4290(10) | 116.217(4) | 116.1620(10) | 96.944(12) | 106.593(2) | 90 |
| γ/° | 90 | 90 | 90 | 90 | 113.139(2) | 90 |
| Volume/Å^3^ | 1302.69(5) | 5979(4) | 5964.48(18) | 3691.6(9) | 1857.8(6) | 9168.2(4) |
| Z | 2 | 4 | 4 | 2 | 1 | 4 |
| ρ_calc_g/cm^3^ | 1.388 | 1.764 | 1.787 | 1.617 | 1.687 | 1.382 |
| μ/mm^‑1^ | 1.848 | 2.154 | 13.430 | 13.393 | 2.068 | 8.794 |
| F(000) | 572.0 | 3192.0 | 3216.0 | 1804.0 | 948.0 | 3840.0 |
| Crystal size/mm^3^ | 0.169 × 0.093 × 0.035 | 0.279 × 0.218 × 0.082 | 0.264 × 0.173 × 0.103 | 0.262 × 0.126 × 0.063 | 0.187 × 0.11 × 0.038 | 0.284 × 0.2 × 0.183 |
| Radiation | CuKα (λ = 1.54178) | MoKα (λ = 0.71073) | CuKα (λ = 1.54178) | CuKα (λ = 1.54178) | MoKα (λ = 0.71073) | CuKα (λ = 1.54178) |
| 2Θ range for data collection/° | 6.116 to 136.61 | 3.54 to 52.802 | 6.094 to 140.374 | 6.68 to 143.44 | 3.528 to 50.95 | 5.81 to 136.676 |
| Index ranges | -6 ≤ h ≤ 6, -34 ≤ k ≤ 34, -10 ≤ l ≤ 10 | -40 ≤ h ≤ 40, -15 ≤ k ≤ 15, -20 ≤ l ≤ 20 | -39 ≤ h ≤ 39, -15 ≤ k ≤ 15, -17 ≤ l ≤ 19 | -12 ≤ h ≤ 12, -22 ≤ k ≤ 22, -23 ≤ l ≤ 21 | -13 ≤ h ≤ 13, -12 ≤ k ≤ 15, -17 ≤ l ≤ 16 | -22 ≤ h ≤ 22, -21 ≤ k ≤ 22, -33 ≤ l ≤ 33 |
| Reflections collected | 17978 | 26038 | 44653 | 24709 | 18678 | 111590 |
| Independent reflections | 2388 [R_int_ = 0.0757, R_sigma_ = 0.0373] | 6120 [R_int_ = 0.0770, R_sigma_ = 0.0704] | 5582 [R_int_ = 0.0588, R_sigma_ = 0.0348] | 6996 [R_int_ = 0.1460, R_sigma_ = 0.1397] | 6825 [R_int_ = 0.0585, R_sigma_ = 0.0735] | 8408 [R_int_ = 0.1335, R_sigma_ = 0.0634] |
| Data/restraints/parameters | 2388/0/213 | 6120/1/402 | 5582/0/402 | 6996/48/472 | 6825/0/492 | 8408/165/536 |
| Goodness-of-fit on F^2^ | 1.022 | 1.058 | 1.086 | 1.020 | 1.024 | 1.094 |
| Final R indexes [I>=2σ (I)] | R_1_ = 0.0468, wR_2_ = 0.1144 | R_1_ = 0.0532, wR_2_ = 0.1296 | R_1_ = 0.0287, wR_2_ = 0.0636 | R_1_ = 0.0722, wR_2_ = 0.1524 | R_1_ = 0.0626, wR_2_ = 0.1578 | R_1_ = 0.0638, wR_2_ = 0.1895 |
| Final R indexes [all data] | R_1_ = 0.0610, wR_2_ = 0.1234 | R_1_ = 0.0776, wR_2_ = 0.1487 | R_1_ = 0.0336, wR_2_ = 0.0653 | R_1_ = 0.1453, wR_2_ = 0.1815 | R_1_ = 0.0791, wR_2_ = 0.1717 | R_1_ = 0.0827, wR_2_ = 0.2052 |
| Largest diff. peak/hole / e Å^-3^ | 0.67/-0.34 | 3.54/-0.79 | 0.57/-0.75 | 0.91/-0.91 | 3.52/-1.60 | 1.10/-0.77 |
|  |  |  |  |  |  | 0.023(4) |

The X-ray crystal structures of the Sm(NO_3_)_3_ and Tb(NO_3_)_3_ complexes with ethoxy-substituted ligand **1** reveal formation of 2:2 dimers in the solid state, where the Ln centers are bound by both the phosphonate P=O and amide C=O groups (Figure 3 shows the structure of the Sm(NO_3_)_3_ complex). In previous work, our group also reported the formation of a polymeric structure for the Sm^3+^ complex[26], which also shows bidendate binding of the phosphate ester and amide groups. The new structures reported here are isomorphs of one another, where the inner sphere of each metal is completed with three bidentate nitrate groups to give two, 10-coordinate metal centers. There is also one molecule of solvent acetonitrile that is not directly bonded to the Ln center, but engaged in a hydrogen bond with one of the ligand’s amide hydrogen atoms. For both structures, the electron density corresponding to the methylene group between the P=O and C=O was disordered. This disorder was modeled over two positions with occupancy ratios of ~0.62:~0.38 for both structures. More details about the treatment of this disorder are included in the Supplementary Materials.


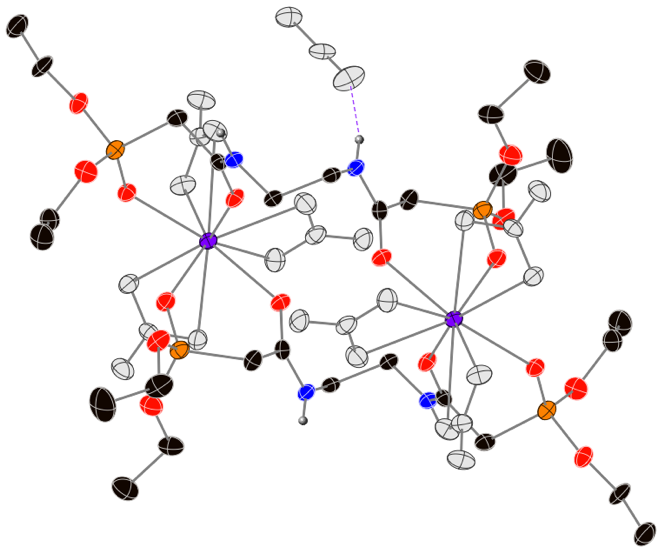


**Figure 3.** X-ray crystal structure of the [Sm(NO_3_)_3_(**1)]_2_**(CH_3_CN) complex, using standard CPK colors for the atoms of ligand **1** (Sm^3+^ = purple spheres). The atoms of the nitrate groups and solvent acetonitrile are depicted as grey spheres to make it easier to visualize the structure of the ligands and the metal’s coordination geometry. Thermal ellipsoids are drawn at the 40% probability level, only hydrogen atoms bonded to nitrogen atoms are shown for clarity, and the hydrogen bonding interaction is depicted with a purple, dashed line. Only the major component is shown for clarity. The Tb^3+^ complex is isomorphic to this structure, and shown in the Supplementary Materials.

Key bond lengths and angles for the free ligand **1** and its Sm(NO_3_)_3_ and Tb(NO_3_)_3_ complexes are summarized in Table 3. The length of the ligand’s carbonyl (C=O) bond increases about 0.02 Å upon metal complexation, indicating that this group is donating electron density to the Ln center. This increase in bond length, and therefore weakening of the C=O bond, is in agreement with the observed decrease in absorption frequency for this bond in the IR data (*vide supra*). For the phosphonate functional group, the length of the P=O bond of the ligand remains more or less the same upon Ln complexation with a maximum increase of 0.01 Å. This slight increase in length also agrees with the IR data discussed above. The bond angle for the beta-phosphonate amide group [C(O)-C-P(O)] is largely unchanged between free and complexed ligand, and the O(C)-Ln-O(P) bond angles are also consistent between the Sm(NO_3_)_3_ and Tb(NO_3_)_3_ complexes at ~74°. Finally, the O-Ln bonds of the ligand are longer for the Sm(NO_3_)_3_ complex than for the Tb(NO_3_)_3_ complex, which is expected due to the larger ionic radius of Sm^3+^.

**Table 3.** Selected bond distances (Å) and angles (°) for ligand **1**[25] and its Sm(NO_3_)_3_ (dimeric), Sm(NO_3_)_3_ (polymeric),[26] and Tb(NO_3_)_3_ complexes.

|  | **Ligand 1**[25] | [Sm(NO_3_)_3_(**1**)]_2_• (CH_3_CN) | [Sm(NO_3_)_3_(**1**)]_2_  (polymer)[26] | [Tb(NO_3_)_3_(**1**)]_2_• (CH_3_CN) |
| --- | --- | --- | --- | --- |
| *ligand* |  |  |  |  |
| C=O | 1.226(2) | 1.242(7), 1.242(7) | 1.245(5), 1.249(4) | 1.246(4), 1.245(4) |
| P=O | 1.474(2) | 1.473(4), 1.478(4) | 1.482(3), 1.485(3) | 1.477(2), 1.478(3) |
| P-O | 1.5791(16), 1.5619(15) | 1.551(5), 1.552(5), 1.551(5), 1.563(5) | 1.515(4), 1.537(4), 1.553(3), 1.542(3) | 1.554(2), 1.560(3), 1.564(3), 1.561(3) |
| Ln-O(C) | ----- | 2.387(4), 2.417(4) | 2.387(3), 2.382(3) | 2.378(2), 2.400(2) |
| Ln-O(P) | ----- | 2.400(4), 2.434(4) | 2.344(3), 2.340(3) | 2.390(2), 2.424(2) |
| C(O)-C-P(O) | 110.9(2) | 110.6(4), 113.1(4) | 109.7(3), 110.1(3) | 110.4(2), 111.2(2) |
| O(C)-Ln-O(P) | ----- | 75.24(14), 73.53(15) | 74.17(10), 74.70(9) | 75.44(8), 74.06(8) |
| *inner sphere water* |  |  |  |  |
| Ln-O(water) | ----- | ----- | 2.413(3) | ----- |

The X-ray crystal structures of the Sm(NO_3_)_3_ and Tb(NO_3_)_3_ complexes with phenyl-substituted ligand **2** were also solved as 2:2 dimers (Figure 4). Here, two different solvates of the Tb(NO_3_)_3_ complex were isolated and analyzed (CH_3_OH and H_2_O). In all three structures the CMPO groups are bound in a bidentate manner to the Ln center. For the Sm(NO_3_)_3_ complex, the inner coordination sphere of the Ln ion is completed with three bidentate nitrate groups to give a 10-coordinate metal center. In the structure of the Tb(NO_3_)_3_(**2**)-methanol solvate, one inner sphere nitrate group was completely displaced by a CH_3_OH ligand to give a 9-coordinate metal. The coordination geometry of this metal center is a distorted monocapped square antiprism, where the oxygen atom O9 is considered to be the cap. A figure showing the inner coordination sphere of this metal is shown in the Supplementary Materials. For this structure, the electron density corresponding to phenyl ring C13-C28, along with the ipso P1 atom, was disordered. This disorder was modeled over two positions with an occupancy ratio of 0.773(17) to 0.267(17). In the structure of the Tb(NO_3_)_3_(**2**)-water solvate, two inner sphere nitrates were displaced by two aqua ligands to give an 8-coordinate metal with a distorted dodecahedral geometry (see Supplementary Materials for figure showing the atoms of the inner coordination shpere). All three Ln(NO_3_)_3_(**2**) complex structures show an extensive hydrogen bonding network between an amide hydrogen atom and a nearby nitrate group or solvent molecule. Interestingly, the only structure that contained intermolecular pi-pi interactions between aromatic rings of the ligands was the Tb(NO_3_)_3_(**2**)-methanol solvate.[27-31] Key bond lengths and angles for the phenyl-substituted ligand **2** and its Sm(NO_3_)_3_ and Tb(NO_3_)_3_ complexes are summarized in Table 4. For this set of complexes, a similar set of trends regarding bond lengths and angles are observed as described for the complexes of these metals with the ethoxy-substituted ligand **1**.


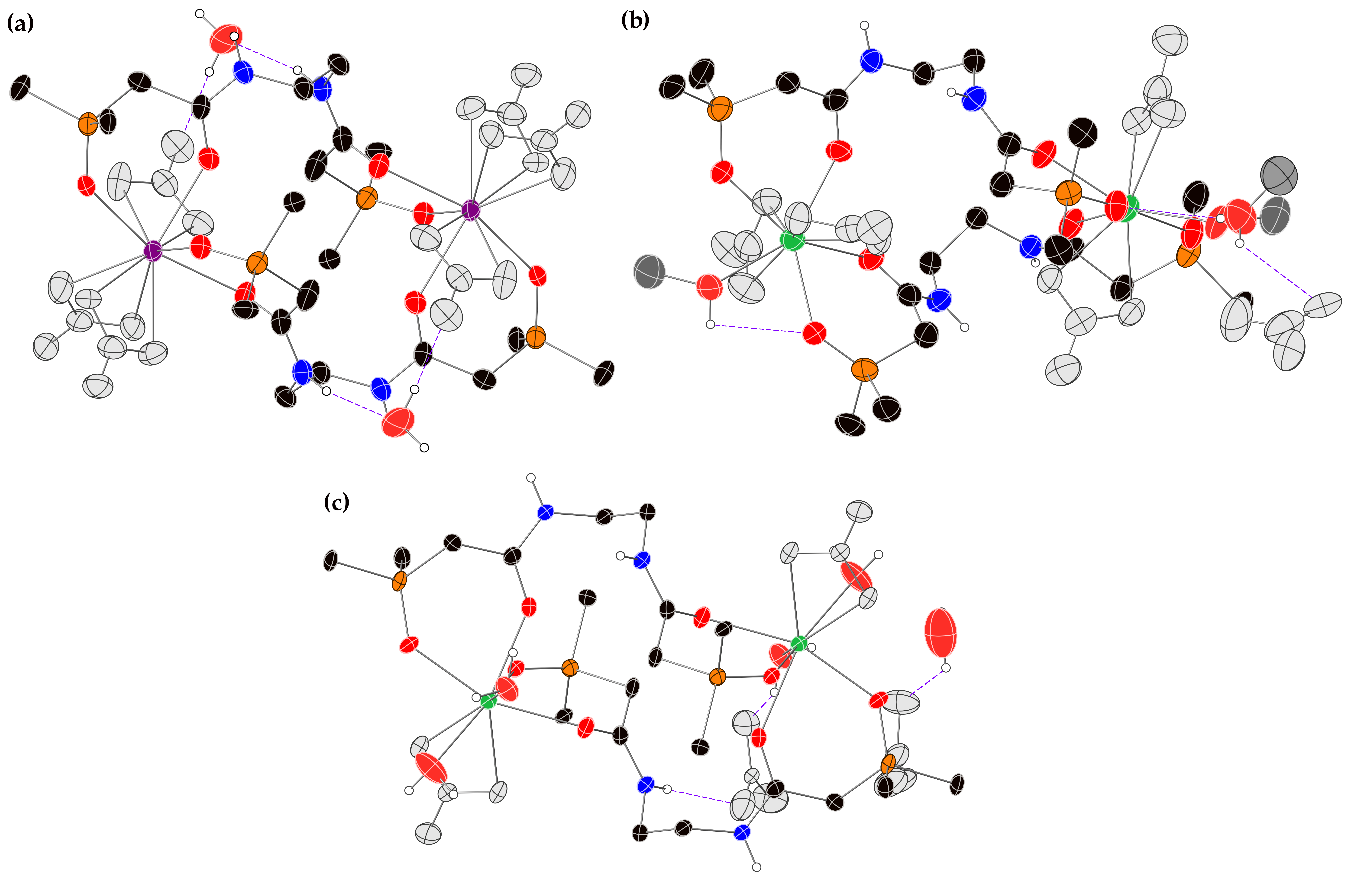


**Figure 4.** X-ray crystal structures of the 2:2 complexes between phenyl-substituted ligand **2** and Sm(NO_3_)_3_ and Tb(NO_3_)_3_ using standard CPK colors (Sm^3+^: purple, Tb^3+^: green). The atoms of the nitrate groups are colored grey in order to make it easier to visualize the ligand and metal coordination geometry. Thermal ellipsoids are drawn at the 40% probability level, and hydrogen bonding interactions are depicted with purple, dashed lines. (a) the [Sm(NO_3_)_3_(**2**)(H_2_O)_2_]_2_ complex; (b) the 2:2 [Tb(NO_3_)_3_(**2**)]_2_•H_2_O complex; (c) the major component of the 2:2 [Tb(NO_3_)_3_(**2**)(MeOH)]_2_ complex. For clarity, only the ipso carbon atom of each phenyl ring is shown, and only hydrogen atoms bonded to oxygen or nitrogen atoms are shown. Complete structures are shown in the Supplementary Materials.

**Table 4.** Selected bond distances (Å) and angles (°) for ligand **2** and its Sm(NO_3_)_3_ and Tb(NO_3_)_3_ complexes. For the Tb(NO_3_)_3_ complex, only the atoms that were not disordered are included here.

|  | **ligand 2** | [Sm(NO_3_)_3_(**2**)]_2_•H_2_O | [Tb(NO_3_)_3_(**2**)(MeOH)]_2_ | [Tb(NO_3_)_3_(**2**)(H_2_O)_2_]_2_ |
| --- | --- | --- | --- | --- |
| *ligand* |  |  |  |  |
| C=O | 1.224(3) | 1.251(11), 1.253(13) | 1.245(15), 1.255(14) | 1.262(8), 1.266(9) |
| P=O | 1.4912(18) | 1.511(7), 1.508(7) | 1.505(9) | 1.511(5), 1.509(5) |
| Ln-O(C) | ----- | 2.514(7), 2.396(7) | 2.365(8), 2.372(8) | 2.412(5), 2.352(5) |
| Ln-O(P) | ----- | 2.360(7), 2.387(7) | 2.332(8) | 2.277(5), 2.310(5) |
| C(O)-C-P(O) | 111.80(17) | 113.0(7), 115.0(8) | 111.7(9) | 112.8(5), 111.3(5) |
| O(C)-Ln-O(P) | ----- | 76.1(2), 74.5(2) | 75.3(3) | 79.05(17), 77.64(18) |
| *inner sphere water* |  |  |  |  |
| Ln-O(water) | ----- | ----- | ----- | 2.356(5), 2.414(6) |

*2.3* *Characterization of the Ln-ligand complexes in solution using ^1^H NMR*

Complexation of the Ln nitrates of Sm^3+^, Eu^3+^ and Tb^3+^ in solution by ligands **1** and **2** was studied by NMR spectroscopy*.* Initial work with DMSO-*d_6_* as a solvent for these experiments revealed that the Ln-ligand complexes were not formed in this highly competitive solvent. As such, we settled on acetonitrile-*d_3_* for NMR studies, which parallels the results of our luminescence experiments (*vide infra*). For the ^1^H NMR spectra of the Eu^3+^ and Tb^3+^ complexes, the resonances were severely broadened relative to the spectra of the ligands alone. However, the resonances in the ^1^H NMR spectra of the Sm^3+^ complexes were only slightly broadened, and were interpretable (Figure 5).

In the ^1^H NMR spectrum of the Sm^3+^-complex of the ethoxy-substituted ligand **1** (Figure 5, top), the resonance corresponding to the methylene group between the C=O and P=O bonds is shifted downfield relative to that of the free ligand **1** (Δδ = 0.68 ppm). The resonance corresponding to the methylene groups of the ethylenediamine cap shifts *upfield* 0.21 ppm for the Sm^3+^-complex relative to the free ligand. Although the magnitude of these shifts are likely influenced by the changing solvent system (CDCl_3_ for free ligand **1**, CD_3_CN for the Sm^3+^-complex), we are confident the magnitude of the shifts are indicative of the formation of a Sm^3+^-ligand **1** complex in solution. Furthermore, the direction of these shifts indicates that the Sm^3+^ ion is being bound in solution by either (or both) of the C=O and P=O groups of the ligand. We attribute the upfield shift of the ethylenediamine -C*H*_2_- resonance to the proximity of this group to a paramagnetic metal center, but that the amide -NH group is not directly involved in binding to this metal.

Information regarding the relative stability of the complexes in solution can also be gleaned by examining these spectra. The ^1^H NMR spectrum of the Sm^3+^-ligand **1** complex displays one set of broad signals, rather than separate signals for free and bound ligand. The broadness of the signals is likely from the presence of the paramagnetic Sm^3+^ ion. The presence of one set of signals indicates that the system experiences fast exchange on the ^1^H NMR time scale. As described above, our group isolated Sm^3+^-**1** dimeric complexes with a 2:2 metal-ligand stoichiometry in the solid state, but in previous work we reported the crystal structure of a metallopolymer chain having a 2:1 Sm(NO_3_)_3_-ligand **1** stoichiometry[26]. We’ve interpreted the combination of this solution ^1^H NMR and solid-state crystallographic data to mean that in solutions of acetonitrile there are varying geometries and stoichiometries of the Sm(NO_3_)_3_-ligand complexes, and they are all exchanging at a rate that is faster than the ^1^H NMR time scale. This dynamic solution is also likely present for the related Eu(NO_3_)_3_ and Tb(NO_3_)_3_ complexes.


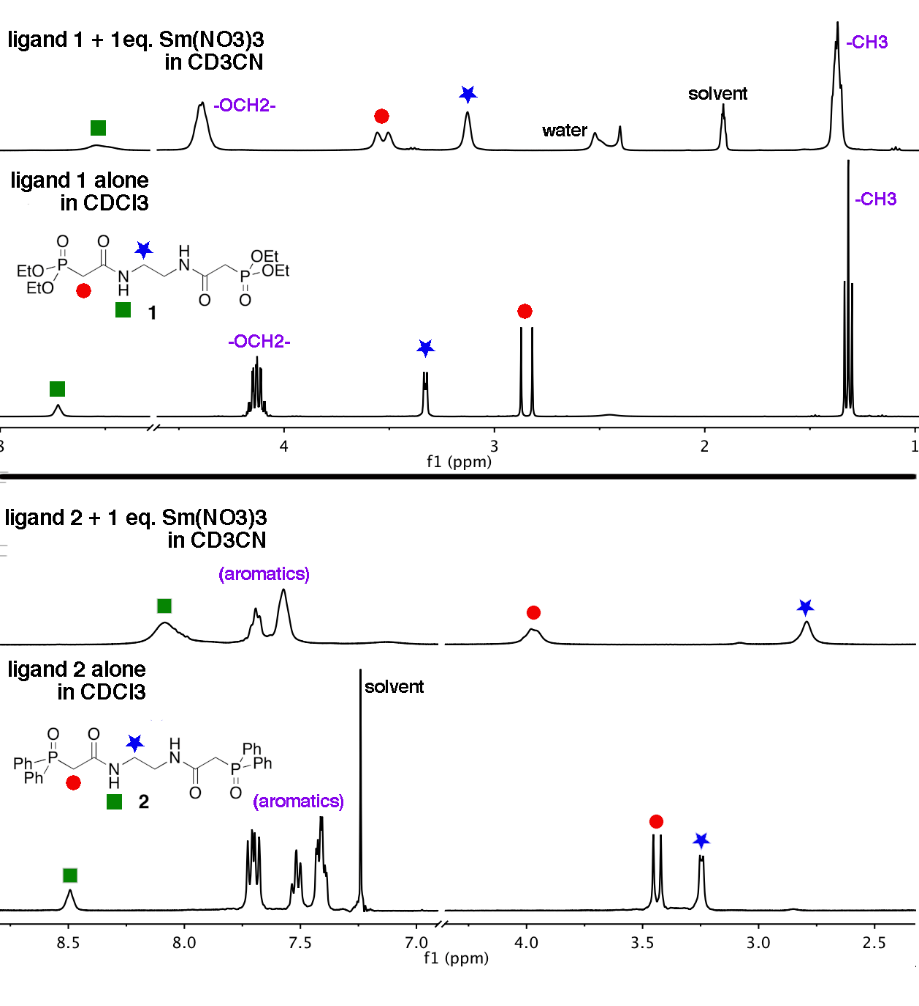


**Figure 5.** ^1^H NMR spectra of ligands 1 and 2, along with their Sm(NO_3_)_3_ complexes. (Top): ligand **1** in CDCl_3_, and with 1 eq. Sm(NO_3_)_3_ in CD_3_CN; (Bottom): ligand **2** in CDCl_3_, and with 1 eq. Sm(NO_3_)_3_ in CD_3_CN. For all spectra, key resonances have been labeled as: -N*H* (green square), -P-C*H*_2_-C- (red circle), -NH-C*H*_2_- (blue star).

For the ^1^H NMR spectrum of the Sm(NO_3_)_3_ complex of ligand **2** in CD_3_CN, we also see one set of relatively broad resonances, indicating that this system is experiencing metal-ligand exchange that is fast on the ^1^H NMR time scale. Similar shifts in the resonances corresponding to the C(O)-C*H*_2_-P(O) and ethylene diamine methylene groups are observed for this ligand system as described for ligand **1** (Δδ = +0.52 and -0.47 ppm, respectively). This indicates that, again, the Sm^3+^ ion is bound by the C=O and P=O groups, rather than the amide -NH group. In the solid state, only dimeric Ln-ligand **2** complexes were isolated by our group. However, the composition of the inner coordination sphere of the metal differed depending on the solvent used to grow the crystals (CH_3_CN vs. H_2_O vs. CH_3_OH). We propose that all Ln-ligand **2** complexes discussed in this paper are also present in a variety of geometries and stoichiometries in solutions of acetonitrile.

*2.4* *Characterization of the Ln-ligand complexes in solution using luminescence*

The final set of experiments our research group carried out to probe the solution behavior of these Ln-ligand complexes involved the characterization of their luminescence properties. We have previously studied a variety of multipodal ligands bearing this beta-phosphonate/phosphine oxide -amide chelating group, and we have shown that these types of compounds have the ability to sensitize the luminescence of Eu^3+^ and Tb^3+^[32-34]. Ligands **1** and **2** described in this paper are also able to sensitize this luminescence, albeit to a modest extent.

Most lanthanide metals (except for La^3+^ and Lu^3+^) luminesce at wavelengths in the near-IR, visible and UV regions of the electromagnetic spectrum. In the visible region, these emission bands are quite narrow (~10 nm), which renders these metals useful for inclusion in a variety of materials[5, 16], bioprobes[9, 13], sensors[11, 35], and metallopolymers[36]. Relatively low molar absorptivities of the Ln ions can make excitation a challenge for such applications; this can be overcome by instead exciting an appropriate bound organic ligand. To facilitate efficient sensitization of the Ln ion and enhance luminescence, the ligand must have an excited triplet state that is slightly higher in energy than the Ln ion emitting state (Δ*E* = 2000-4000 cm^-1^). This process was first discovered by Weissman in 1942[37], and a simplified Jablonski diagram describing the sensitization process (or, “antenna effect”) is shown in the Supplementary Materials.

We investigated the ability of ligands **1** and **2** to sensitize the luminescence of Eu^3+^ and Tb^3+^ in solutions of acetonitrile. A complex stoichiometry of 1:1 in solution was used, to mimic what was observed in our solid-state X-ray diffraction studies. The absorption and excitation spectra of the Eu(NO_3_)_3_-**1** and -**2** complexes are shown in Figure 6. The absorption spectrum of ethoxy-substituted ligand **1** is broad with a λ_max_ at 207 nm (shown in the Supplementary Materials). The λ_max_ of the UV-VIS absorption spectrum of the Eu(NO_3_)_3_(**1**)complex is red-shifted to 224 nm, and the excitation spectrum has a λ_mas_ at 240 nm. All three spectra are broad and have similar shapes. Similarly, the absorption and excitation spectra of the Eu(NO_3_)_3_(**2**) complex are similar with λ_max_ values of 266 and 277 nm, respectively (Figure 5). The absorption spectrum of the free phenyl-substituted ligand **2** is also broad with a λ_max_ of 224 nm (shown in Supplementary Materials). These features support the complexation of the Eu^3+^ ion by ligands **1** and **2** in solutions of acetonitrile, as well as the sensitization of metal centered emission. The spectra of the Tb(NO_3_)_3_ complexes of both ligands **1** and **2** are similar to that of the Eu^3+^ complexes, and are shown in the Supplementary Materials.

**
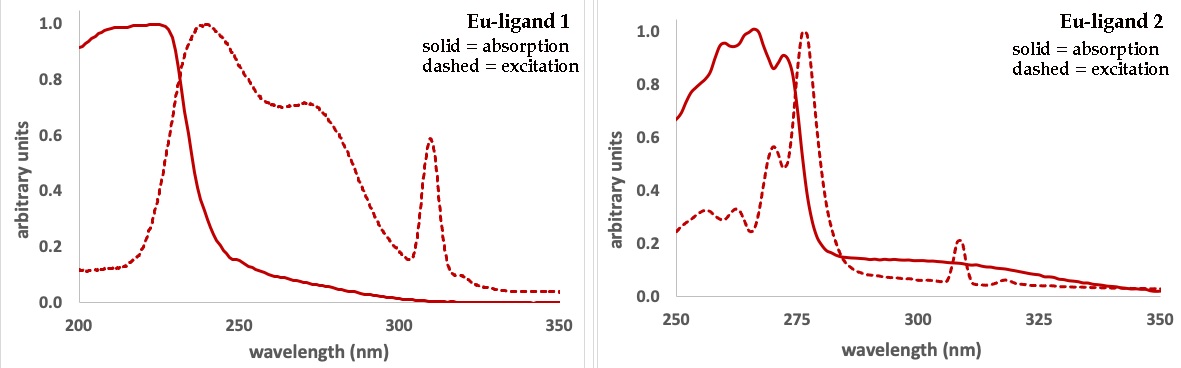
**

**Figure 6.** Absorption (solid line) and excitation (dashed line) spectra for the (left) Eu(NO_3_)_3_(**1**) and (right) Eu(NO_3_)_3_(**2**)**­** complexes in acetonitrile (1.0 mM complex concentration, emission wavelengths were monitored at 620 nm for the complex with ligand **1**, and 617 nm for the complex with ligand **2**, 2.0 nm excitation and emission slit widths). Note that the intensity values of all spectra shown here have been normalized to have their λ_max_ equal one (1.0) for ease of comparison.

The emission spectra for 1:1 solutions of ligands **1** and **2** with Eu(NO_3_)_3_ and Tb(NO_3_)_3_ in acetonitrile are shown in Figure 7. Solutions containing the Ln-complexes of ligand **1** were excited at 238 nm, while complexes of ligand **2** were excited at 260 nm. In both cases, characteristic emission bands are observed[16], with the Tb^3+^ complexes being brighter than the Eu^3+^ complexes. The spectra of the Tb^3+^ complexes are nearly identical, with the ^5^D_4_ → ^7^F*_J_* (*J* = 6, 5, 4, 3) transitions appearing at 489, 544, 585 and 622 nm, respectively. The spectra of the Eu^3+^ complexes are also quite similar, with peaks at 593 and 617 nm, corresponding to the ^5^D_0_ → ^7^F*_J_* transitions (*J* = 1, 2).


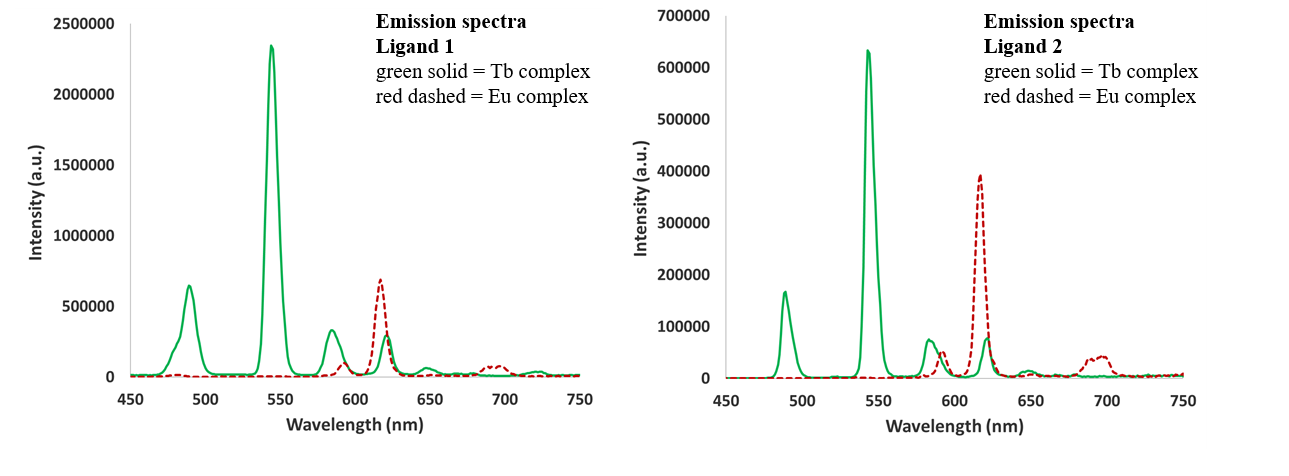


**Figure 7.** Emission spectra of solutions of ligands **1** (left) and **2** (right) with one equivalent of Eu(NO_3_)_3_ and Tb(NO_3_)_3_ in acetonitrile. For ligand **1**: excitation wavelength: 238 nm, excitation and emission slit widths = 5 nm, 2.2 mM ligand concentration; for ligand **2**: excitation wavelength: 260 nm, excitation and emission slit widths = 2 nm, 1.0 mM ligand concentration. Note: for the acquisition of the emission spectra of the ligand **1**-complexes the complex concentration is more than twice that of ligand **2**, and the excitation and emission slits were widened to accommodate the dimmer emission of these complexes.

Luminescence decay lifetimes for each of the 1:1 Ln-ligand complexes of the phenyl-substituted ligand **2** were acquired in acetonitrile. The lifetime values shown in Table 5 are from fits of the data to a single exponential decay. Decay lifetimes of 1.35 and 1.83 ms were obtained for the Eu^3+^ and the Tb^3+^ complex of ligand **2**, respectively. We did not measure luminescence decay lifetimes for complexes of the ethoxy-substituted ligand **1** because they were substantially dimmer than those with the phenyl-substituted ligand **2**.

**Table 5.** Emission lifetimes for 1:1 complexes of Eu(NO_3_)_3_ and Tb(NO_3_)_3_ with ligand **2** in acetonitrile. The error bars on these numbers represent the standard deviation from three trials.

| **ligand 2 complex** | **τ_ACN_/ms** |
| --- | --- |
| Eu | 1.35 ± 0.08 (at 616 nm) |
| Tb | 1.83 ± 0.05 (at 545 nm) |

3. Scope and Outlook

We have described here the characterization of two compounds bearing two-bidentate chelating groups as ligands for the Ln nitrate salts of Sm^3+^, Eu^3+^ and Tb^3+^. These organic compounds feature a relatively flexible ethylenediamine backbone, with hard (albeit neutral) oxygen donor atoms to facilitate Ln binding. As such, the Ln-ligand complexes are dynamic in solution as evidenced by the ^1^H NMR data and varied solid state X-ray structures presented here. These ligands are also able to sensitize the luminescence of Eu(NO_3_)_3_ and Tb(NO_3_)_3_, showing modest emission in solutions of acetonitrile. The impacts of straightforward derivations of the ligand backbone on these properties would be an interesting next set of experiments. For instance, would complex stability be increased with a more rigid backbone? Would the metal-centered luminescence be brighter with a conjugated backbone? These are questions we will continue to investigate to further expand the scope of such ligands for lanthanide coordination.

4. Materials and Methods

***4.1 General considerations***

All chemicals (including deuterated solvents) were used as purchased from Sigma-Aldrich, Strem Chemical or Acros Chemicals and used without further purification. ^1^H, ^13^C and ^31^P NMR spectral data were recorded on either a JEOL ECZS 400 or Varian Inova 400 FTNMR spectrophotometer. For ^1^H and ^13^C NMR spectra, chemical shifts are expressed as parts per million (δ) relative to SiMe_4_ (TMS, δ = 0), and referenced internally with respect to the protio solvent impurity. For ^31^P NMR spectra, chemical shifts are expressed as parts per million (δ) relative to H_3_PO_4_ (δ = 0). Both ^13^C and ^31^P NMR spectra were obtained as proton-decoupled data. IR spectra were acquired neat on a Jasco 4100 FTIR. Elemental (CHN) analyses were performed by Atlantic Microlab Inc., Norcross, GA; all CHN percentages calculated for lanthanide complexes assume one CMPO ligand + Ln(NO_3_)_3_ + residual water/solvents as indicated. Mass spectrometry data were acquired by the Lumigen Instrument Center at Wayne State University. Absorption spectra were acquired on a Shimadzu UV-2450 or Agilent 8453 UV-VIS spectrophotometer. Luminescence data were collected on either a Horiba Fluoromax 4 or a Hitachi F-7000 spectrophotometer. For determination of lifetime values, the decay curve was fit to a single exponential decay using the Horiba FluorEssence software package. These curves, along with the residuals, are shown in the Supplementary Materials.

***4.2 Single crystal X-Ray crystallography***

Crystals suitable for X-ray diffraction were mounted on a nylon loop using a small amount of paratone oil. Data were collected using a Bruker CCD (charge coupled device) based diffractometer equipped with an Oxford Cryostream low-temperature apparatus operating at 173(2) K. Data were measured using omega and phi scans of 0.5° per frame. The total number of images was based on results from the program COSMO[38] where redundancy was expected to be 4.0 and completeness of 100% out to 0.83 Å. Cell parameters were retrieved using APEX II software[39] and refined using SAINT on all observed reflections. Data reduction was performed using the SAINT software[40], which corrects for Lp. Scaling and absorption corrections were applied using SADABS[41] multi-scan technique, supplied by George Sheldrick. The structures were solved by the direct method using the SHELXS-97 program and refined by least squares method on F^2^, SHELXL-2014[42], which are incorporated in OLEX2[43, 44]. All non-hydrogen atoms were refined anisotropically. Unless otherwise noted, the locations of hydrogen atoms were calculated by geometrical methods and refined as a riding model. The crystals used for the diffraction studies showed no decomposition during data collection. Further crystallographic data and experimental details for structural analysis of all the complexes are summarized in Table 2, and selected bond lengths and angles with their estimated standard deviations are given in Tables 3 and 4. Complete tables for each structure reported here, along with diagrams depicting the thermal ellipsoids and descriptions of how any disordered electron density was treated, are provided in the Supplementary Information.

***4.3 Photophysical studies***

All luminescence studies were carried out with a 1:1 ratio of Ln(NO_3_)_3_•xH_2_O to ligands **1** and **2** in chromasolv grade CH_3_CN, or HPLC grade CH_3_OH. Solutions of complexes were prepared by either (a) dissolving solid ligand into an appropriate volume of solvent and adding solid metal or (b) combining appropriate volumes of ligand and metal stock solutions. Metal complex solutions were prepared immediately before analysis and were all at an overall 1.0 mM concentration of the 1:1 Ln-**1** and Ln-**2** complexes. Emission spectra were corrected for the varying sensitivity of the detector by applying the manufacturer’s protocols using the Horiba FluorEssence software package.

***4.4 Synthesis***

**Ligand 1.** This synthetic procedure represents an alternative to what was previously published by our group[25]. Ethylene diamine (1.0 mL, 0.015 mol) was dissolved in 8.3 mL methanol in a 25 mL round bottom flask. The mixture was cooled to -78 °C, and 8.9 mL (0.045 mol) triethyl phosphonoacetate **4** was added dropwise. The reaction mixture was allowed to warm to room temperature and stirred overnight. The solution was poured into methylene chloride (150 mL), and was extracted with 1M HCl (3 x 25 mL), saturated NaHCO_3_ (3 x 25 mL), and brine (1 x 25 mL). The aqueous layers were combined, and the product was back extracted with chloroform (3 x 25 mL). The volatiles were removed under reduced pressure to give 0.59 mg of pure ligand **1** (62%) as an off-white solid. IR: ν (cm^-1^): 3268 (N-H), 1666 (C=O), 1203 (P=O), 1016 (C-O), 963 (C-O); UV-VIS (1.0 mM, CH_3_CN): λ_max_ 207 nm; ^1^H NMR (300 MHz, CDCl_3_): δ 7.73 (broad, 2H, -N*H*), 4.14 (q, *J* = 7.0 Hz, 8H), 3.34 (d, *J* = 5.9 Hz, 4H), 2.85 (d, *J*_P-H_ = 15.8 Hz, 4H), 1.33 (t, *J* = 7.0 Hz, 12H); ^13^C NMR (75 MHz, CDCl_3_): δ 165.4, 62.9, 35.8 (d, *J*_P-C_ = 128 Hz), 16.5; ^31^P NMR (121 MHz, CDCl_3_): δ 24.5; HR-MS (ESI): expected for C_14_H_30_N_2_O_8_P_2_Na^+^: 439.1370; found: 439.1367. The X-ray crystal structure of this compound has been reported[25].

**Ligand 2.** Ethylenediamine (0.135 mL, 0.002 mol) and the *p*-nitrophenol ester **5**[24] (2.32 g, 0.006 mol) were dissolved in anhydrous, amylene-stabilized chloroform (42.5 mL) and stirred in a round bottom flask equipped with a reflux condenser under an atmosphere of nitrogen. The reaction mixture was heated to 45 °C and stirred for three days. After the solution was allowed to cool to room temperature, a few cm^3^ of 40% KOH was added and the reaction was stirred for four hours. The organic layer was separated and washed with 5% sodium carbonate (30 mL) and water (3 x 30 mL) and dried over anhydrous MgSO_4_. The organic volatiles were removed under reduced pressure to give a solid, which was triturated with ethyl acetate (3 x 5 mL). The off-white solid was placed under high vacuum overnight to give the pure product (0.38 g, 16% yield). IR: ν = 1661 cm^-1^ (C=O), 1173 cm^-1^ (P=O). UV-VIS (1.0 mM, CH_3_CN): λ_max_ 224 nm (note: there was undissolved ligand in the cuvette for this analysis; ligand **2** is poorly soluble in CH_3_CN); ^1^H NMR (400 MHz, CDCl_3_): δ = 8.49 (broad s, 2H, N*H*), 7.72 (m, 8H), 7.69 (m, 8H), 7.55 (m, 4H), 3.45 (d, *J*_P-H_ = 13.6 Hz, 4H), 3.26 (m, 4H); ^31^C NMR (100 MHz, CDCl_3_): δ = 165.4 (d, *J*_P-C_ = 5.9 Hz), 132.3 (d, *J*_P-C_ = 2.8 Hz), 131.8 (d, *J*_P-C_ = 103 Hz), 131.1 (d, *J*_P-C_ = 10.1 Hz), 128.8 (d, *J*_P-C_ = 12.2 Hz), 39.6 (d, *J*_P-C_ = 60.1 Hz), 38.4 (s); ^31^P NMR (161 MHz, CDCl_3_): δ = 32.1. HR-MS (ESI): expected for C_30_H_30_N_2_O_4_P_2_H^+^: 545.1754; found: 545.1755. Crystals suitable for X-ray diffraction were grown by vapor diffusion of diethyl ether into a solution of the Tb-complex in methanol.

**Ln-ligand complexes.** A general procedure for the synthesis of all Ln-ligand complexes is described below. Note that ligand **2** is poorly soluble in acetonitrile, but upon addition of the Ln(NO_3_)_3_ to the reaction mixture the ligand dissolved readily. Compound **1** or **2** (50 mg) was added to a round bottom flask and dissolved in 10-15 mL acetonitrile. One molar equivalent of the lanthanide nitrate hydrate was added as a solid, and the reaction mixture was stirred for thirty minutes at room temperature. The solvent was removed under reduced pressure to give a thin, clear film. Approximately 5 mL of diethyl ether was added to the flask and the film was scraped away from the glass using a metal spatula to give an off-white powder. The ether was removed with a Pasteur pipette, and the remaining solid was triturated one or two more times with fresh ether (~5 mL). The metal-ligand complexes were placed under high vacuum overnight to give the products in isolated yields that ranged from 70-80%. Characterization data for each Ln-ligand complex is given below; the ^1^H NMR spectra of the Eu^3+^ and Tb^3+^ complexes were quite broad and are not reported.

**Sm-1(NO_3_)_3_.** IR: ν (cm^-1^) = 3317 (N-H), 1626 (C=O), 1191 (P=O), 1014 (C-O); ^1^H NMR (CD_3_CN, 400 MHz, integration values are not reliable for this complex so they have not been reported): δ 7.54 (broad), 4.39 (broad s), 3.53 (d, *J*_P-H_ = 20.8 Hz), 3.13 (broad s), 1.37 (broad t, *J* = 3.7 Hz); ^13^C NMR (CD_3_CN, 100 MHz): δ 168.3 (d, *J*_P-c_ = 41 Hz), 65.3 (s), 39.6 (s), 32.9 (d, *J*_P-C_ = 139 Hz), 15.6 (s); ^31^P NMR (CD_3_CN, 161 MHz): δ 24.4; ESI-LRMS (M^+^, *m/z*): calcd for (C_14_H_30_N_2_O_8_P_2_)Sm(NO_3_)_2_: 692, found 692; calcd for (C_14_H_30_N_2_O_8_P_2_)_2_Sm(NO_3_)_2_: 1108, found 1108; Anal. calcd. for (C_14_H_30_N_2_O_8_P_2_)Sm(NO_3_)_3_(CH_3_CN)(C_4_H_10_O) (found): C, 24.44 (24.16); H, 4.41 (4.23); N, 8.55 (8.19). Crystals suitable for analysis by X-ray diffraction were grown by vapor diffusion of diethyl ether into a solution of the complex in acetonitrile. We note here that the crystal used to collect data was quite large and refracted exceptionally well. Because of this the absorption correction was not adequate, and there are large residual positive and negative electron density peaks in the model.

**Eu-1(NO_3_)_3_.** IR: ν (cm^-1^) = 3328 (N-H), 1627 (C=O), 1191 (P=O), 1013 (C-O); ESI-LRMS (M^+^, *m/z*): calcd for (C_14_H_30_N_2_O_8_P_2_)Eu(NO_3_)_2_: 693, found 693; calcd for (C_14_H_30_N_2_O_8_P_2_)_2_Eu(NO_3_)_2_: 1109, found 1109; Anal. calcd. for (C_14_H_30_N_2_O_8_P_2_)Eu(NO_3_)_3_ (found): C, 22.29 (22.69); H, 4.01 (4.23); N, 9.28 (8.75).

**Tb-1(NO_3_)_3_.** IR: ν (cm^-1^) = 3321 (N-H), 1627 (C=O), 1191 (P=O), 1014 (C-O); ESI-LRMS (M^+^, *m/z*): calcd for (C_14_H_30_N_2_O_8_P_2_)_2_Tb(NO_3_)_2_: 1115, found 1115; Anal. calcd. for (C_14_H_30_N_2_O_8_P_2_)Tb(NO_3_)_3_ (found): C, 22.09 (22.46); H, 3.97 (4.30); N, 9.20 (8.71). Crystals suitable for analysis by X-ray diffraction were grown by vapor diffusion of diethyl ether into a solution of the complex in acetonitrile.

**Sm-2(NO_3_)_3_.** IR: ν (cm^-1^) = 1623 (C=O), 1157 (P=O); ^1^H NMR (CD_3_CN, 400 MHz, all resonances are broad, and integration values are not reliable for this complex so they have not been reported): δ 8.08 (N*H*), 7.80-7.50, 3.97, 2.79; ^31^P NMR (161 MHz, CD_3_CN): δ 39.4 (broad); ^13^C NMR data was not obtained for this complex due to the relatively low solubility in CD_3_CN. ESI-LRMS (M^+^, *m/z*): calcd for (C_30_H_30_N_2_O_4_P_2_)_2_ Tb(NO_3_)_2_: 1364, found 1364; Anal. calcd. for (C_30_H_30_N_2_O_4_P_2_)Sm(NO_3_)_3_(CH_3_CN)(C_4_H_10_O) (found): C, 38.91 (39.20); H, 3.90 (3.61); N, 7.56 (7.38). Crystals suitable for analysis by X-ray diffraction were grown by slow evaporation of a CDCl_3_ solution in a NMR tube.

**Eu-2(NO_3_)_3_.** IR: ν (cm^-1^) = 1621 (C=O), 1156 (P=O); ESI-LRMS (M^+^, *m/z*): calcd for (C_30_H_30_N_2_O_4_P_2_)_2_Eu(NO_3_)_2_: 1365, found 1365; Anal. calcd. for (C_30_H_30_N_2_O_4_P_2_)Eu(NO_3_)_3_(H_2_O) (found): C, 37.75 (38.00); H, 3.59 (3.46); N, 7.34 (7.26).

**Tb-2(NO_3_)_3_.** IR: ν (cm^-1^) = 1621 (C=O), 1156 (P=O); ESI-LRMS (M^+^, *m/z*): calcd for (C_30_H_30_N_2_O_4_P_2_) Tb(NO_3_)_2_: 827, found 827; calcd for (C_30_H_30_N_2_O_4_P_2_)_2_Tb(NO_3_)_2_: 1371, found 1371; Anal. calcd. for (C_30_H_30_N_2_O_4_P_2_)Tb(NO_3_)_3_(H_2_O) (found): C, 37.49 (37.45); H, 3.57 (3.31); N, 7.29 (7.11). Crystals suitable for analysis by X-ray diffraction were grown by vapor diffusion of diethyl ether into a solution of the complex in acetonitrile.

**Supplementary Materials:** The following are available online at www.mdpi.com/xxx/s1, (1) X-Ray crystallography: full refinement details, tables of bond lengths and angles, figures of each structure depicting the thermal ellipsoids and atom numbering scheme, and descriptions of how any disordered electron density was treated; (2) Luminescence data: all UV-VIS absorption and excitation spectra, as well as all lifetime decay curves. All crystallographic data has been submitted to the Cambridge Structural Database with CCDC numbers 2003369-2003374. These files can be accessed free of charge at: www.ccdc.cam.ac.uk

**Funding:** We are grateful to the National Science Foundation, grant numbers MRI CHE-1725699, MRI CHE-1919817 and REU CHE-1559886 for instrument and student support. Additional financial support was provided by GVSU (OURS, CSCE, DSR, Chemistry Department Weldon Fund) and The University of Tampa. The instruments at Michigan State University were purchased or upgraded using departmental funds, and the Rigaku Synergy S Diffractometer was purchased with support from the MRI program by the National Science Foundation under grant No. 1919565.

**Acknowledgments:** We thank Pfizer, Inc. for the donation of a 400 MHz Varian INOVA NMR Spectrometer. We also thank Angelina Delabbio for acquiring the IR spectra of the Ln-**2** complexes.

**Author contributions:** Conceptualization, Eric J. Werner and Shannon M. Biros; Data curation, Eric J. Werner and Shannon M. Biros; Funding acquisition, Eric J. Werner and Shannon M. Biros; Investigation, Alan R. Lear, Jonah Lenters, Michael G. Patterson, Richard J. Staples, Eric J. Werner and Shannon M. Biros; Methodology, Eric J. Werner and Shannon M. Biros; Validation, Eric J. Werner and Shannon M. Biros; Writing – original draft, Shannon M. Biros; Writing – review & editing, Alan R. Lear, Jonah Lenters, Richard J. Staples, Eric J. Werner and Shannon M. Biros.

**Conflicts of Interest:** The authors declare no conflict of interest. The funders had no role in the design of the study; in the collection, analyses, or interpretation of data; in the writing of the manuscript, or in the decision to publish the results.

**Literature Cited.**

1. Cotton, S. *Lanthanide and Actinide Chemistry*. Wiley: 2006; p 280.

2. Bünzli, J.-C. G.; Piguet, C., "Taking Advantage of Luminescent Lanthanide Ions". *Chem. Soc. Rev.* **2005,** *34*, 1048-1077.

3. Bünzli, J.-C. G., "Benefiting from the Unique Properties of Lanthanide Ions". *Acc. Chem. Res.* **2006,** *39*, 53-61.

4. de Bettencourt-Dias, A., "Small Molecule Luminescent Lanthanide Ion Complexes - Photophysical Characterization and Recent Developments". *Curr. Org. Chem.* **2007,** *11*, 1460-1480.

5. de Bettencourt-Dias, A. *Luminescence of Lanthanide Ions in Coordination Compounds and Nanomaterials*. 1st ed.; Wiley: Chichester, 2014; p 384.

6. Bünzli, J.-C. G., "On the design of highly luminescent lanthanide complexes". *Coord. Chem. Rev.* **2015,** *293-294*, 19-47.

7. Hemmilä, I.; Laitala, V., "Progress in lanthanides as luminescent probes". *J. Fluoresc.* **2005,** *15*, 529-542.

8. Bünzli, J.-C. G., "Lanthanide Luminescent Bioprobes (LLBs)". *Chem. Lett.* **2009,** *38*, 104-109.

9. Thibon, A.; Pierre, V. C., "Principles of Responsive Lanthanide-Based Luminescent Probes". *Anal. Bioanal. Chem.* **2009,** *394*, 107-120.

10. Parker, D., "Luminescent Lanthanide Sensors for pH, *p*O_2_ and Selected Anions". *Coord. Chem. Rev.* **2000,** *205*, 109-130.

11. Spangler, C.; Schäferling, M., Luminescent Chemical and Physical Sensors Based on Lanthanide Complexes. In *Lanthanide Luminescence: Photophysical, Analytical and Biological Aspects*, Hänninen, P.; Härmä, H., Eds. Springer Heidelberg: Dordrecht, London, New York, 2011; p 400.

12. Faulkner, S.; Pope, S. J. A.; Burton-Pye, B. P., "Lanthanide complexes for luminescence imaging applications". *Appl. Spectrosc. Rev.* **2005,** *40*, 1-31.

13. Bünzli, J.-C. G., "Lanthanide Luminescence for Biomedical Analyses and Imaging". *Chem. Rev.* **2010,** *110*, 2729-2755.

14. Sprecher, B.; Xiao, Y.; Walton, A.; Speight, J.; Harris, R.; Kleijn, R.; Visser, G.; Kramer, G. J., "Life cycle inventory of the production of rare earths and the subsequent production of NdFeB rare earth permanent magnets". *Environ. Sci. Technol.* **2014,** *48*, 3951-3958.

15. Binnemans, K., "Lanthanide-Based Luminescent Hybrid Materials". *Chem. Rev.* **2009,** *109*, 4283-4374.

16. Eliseeva, S. V.; Bünzli, J.-C. G., "Lanthanide Luminescence for Functional Materials and Bio-sciences". *Chem. Soc. Rev.* **2010,** *39*, 189-227.

17. Jowitt, S. M.; Werner, T. T.; Weng, Z.; Mudd, G. M., "Recycling of the rare earth elements". *Curr. Opin. Green Sustain Chem.* **2018,** *13*, 1-7.

18. Binnemans, K.; Jones, P. T.; Blanpain, B.; Van Gerven, T.; Yang, Y.; Walton, A.; Buchert, M., "Recycling of rare earths: A critical review". *J. Clean. Prod.* **2013,** *51*, 1-22.

19. Haque, N.; Hughes, A.; Lim, S.; Vernon, C., "Rare Earth Elements: Overview of Mining, Mineralogy, Uses, Sustainability and Environmental Impact". *Resources* **2014,** *3*, 614-635.

20. Kumari, A.; Panda, R.; Jha, M. K.; Kumar, J. R.; Lee, J. Y., "Process development to recover rare earth metals from monazite mineral: A review". *Miner. Eng.* **2015,** *79*, 102-115.

21. Ansari, S. A.; Mohapatra, P. K., "A review on solid phase extraction of actinides and lanthanides with amide based extractants". *J. Chrom. A* **2017,** *1499*, 1-20.

22. Horwitz, E. P.; Kalina, D. C.; Diamond, H.; Vandegrift, G. F.; Schulz, W. W., "The TRUEX Process - A Process for the Extraction of the Transuranic Elements from Nitric Acid Wastes Utilizing Modified PUREX Solvent". *Solvent Extr. Ion Exch.* **1985,** *3*, 75-109.

23. Hamdouchi, C.; de Blas, J.; del Prado, M.; Gruber, J.; Heinz, B. A.; Vance, L., "2-Amino-3-substituted-6-[(E)-1-phenyl-2-(N-methylcarbamoyl)vinyl]imidazo-[1,2-a]pyridines as a Novel Class of Inhibitors of Human Rhinovirus: Stereospecific Synthesis and Antiviral Activity". *J. Med. Chem.* **1999,** *42*, 50-59.

24. Arnaud-Neu, F.; Böhmer, V.; Dozol, J.-F.; Grüttner, C.; Jakobi, R. A.; Kraft, D.; Mauprivez, O.; Rouquette, H.; Schwing-Weill, M.-J.; Simon, N.; Vogt, W., "Calixarenes with diphenylphosphoryl acetamide functions at the upper rim. A new class of highly efficient extractants for lanthanides and actinides". *J. Chem. Soc. Perkin Trans. 2* **1996**, 1175-1182.

25. VanderWeide, A. I.; Staples, R. J.; Biros, S. M., "Crystal structures of two bis-carbamoylmethylphosphine oxide (CMPO) compounds". *Acta Cryst.* **2019,** *E****75***, 991-996.

26. Stoscup, J. A.; Staples, R. J.; Biros, S. M., "Crystal structure of a samarium(III) nitrate chain cross-linked by a bis-carbamoylmethylphosphine oxide ligand". *Acta Cryst.* **2014,** *E****70***, 188-191.

27. Spek, A. L. *PLATON*, Utrecht University, The Netherlands, 1980.

28. Spek, A. L., "Single-crystal structure validation with the program PLATON". *J. Appl. Cryst.* **2002,** *36*, 7-13.

29. Spek, A. L., "Structure validation in chemical crystallography". *Acta Cryst.* **2009,** *D65*, 148-155.

30. Spek, A. L., "What makes a crystal structure report valid?". *Inorg. Chim. Acta.* **2018,** *470*, 232-237.

31. Spek, A. L., "*checkCIF* validation ALERTS: what they mean and how to respond". *Acta Cryst.* **2020,** *E76*, 1-11.

32. Sartain, H. T.; McGraw, S. N.; Lawrence, C. T.; Werner, E. J.; Biros, S. M., "A Novel Tripodal CMPO Ligand: Affinity for f-Elements, Computational Investigations and Luminescence Properties". *Inorg. Chim. Acta.* **2015,** *426*, 126-135.

33. Coburn, K. M.; Hardy, D. A.; Patterson, M. G.; McGraw, S. N.; Peruzzi, M. T.; Boucher, F.; Beelen, B.; Sartain, H. T.; Neils, T.; Lawrence, C. L.; Staples, R. J.; Werner, E. J.; Biros, S. M., "f-Element Coordination and Extraction Selectivity of a Carbamoylmethylphosphine Oxide Ligand Based on a Tripodal Phosphine Oxide Scaffold ". *Inorg. Chim. Acta.* **2016,** *449*, 96-106.

34. Patterson, M. G.; Mulville, A. K.; Connor, E. K.; Henry, A. T.; Hudson, M. J.; Tissue, K.; Biros, S. M.; Werner, E. J., "Lanthanide extraction selectivity of a tripodal carbamoylmethylphosphine oxide ligand system". *Dalton Trans.* **2018,** *47*, 14318-14326.

35. Cable, M. L.; Levine, D. J.; Kirby, J. P.; Gray, H. B.; Ponce, A., "Luminescent Lanthanide Sensors". *Adv. Inorg. Chem.* **2011,** *63*, 1-45.

36. de Bettencourt-Dias, A.; Rossini, J. S. K., "Ligand design for luminescent lanthanide-containing metallopolymers". *Inorg. Chem.* **2016,** *55*, 9954-9963.

37. Weissman, S. I., "Intramolecular Energy Transfer: The Fluorescence of Complexes of Europium". *J. Chem. Phys.* **1942,** *10*, 214-217.

38. 1.61, C. v. *Software for the CCD detector systems for determining data collection parameters*, Bruker Analytical X-ray Systems: Madison, WI, 2009.

39. v2010.11-3, A. *Software for the CCD detector system*, Bruker Analytical X-ray Systems: Madison, WI, 2010.

40. 7.68A, S. v. *Software for the Integration of CCD Detector System*, Bruker Analytical X-ray Systems: Madison, WI, 2010.

41. Blessing, R. H., "SADABS v2.008/2 Program for absorption correction using Bruker-AXS CCD based on the method of Robert Blessing". *Acta Cryst.* **1995,** *A51*, 33-38.

42. Sheldrick, G. M., "A short history of SHELX". *Acta Cryst.* **2008,** *A64*, 112-122.

43. Dolomanov, O. V.; Bourhis, L. J.; Gildea, R. J.; Howard, J. A. K.; Puschmann, H., "OLEX2: a complete structure solution, refinement and analysis program". *J. Appl. Cryst.* **2009,** *42*, 339-341.

44. Bourhis, L. J.; Dolomanov, O. V.; Gildea, R. J.; Howard, J. A. K.; Puschmann, H., "The anatomy of a comprehensive constrained, restrained, refinement program for the modern computing environment - *Olex2* dissected". *Acta Cryst.* **2015,** ***A71***, 59-75.

| 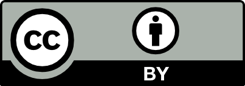 | © 2019 by the authors. Submitted for possible open access publication under the terms and conditions of the Creative Commons Attribution (CC BY) license (http://creativecommons.org/licenses/by/4.0/). |
| --- | --- |
