# Supplementary material for: Two Beta-Phosphorylamide Compounds as Ligands for Sm3+, Eu3+, and Tb3+: X-ray Crystallography and Luminescence Properties
Source: Molecules. 2020 Jun 28;25(13):2971. doi: 10.3390/molecules25132971 (PMC7411983; doi:10.3390/molecules25132971)

checkCIF/PLATON report

Structure factors have been supplied for datablock(s) gvsu819f

THIS REPORT IS FOR GUIDANCE ONLY. IF USED AS PART OF A REVIEW PROCEDURE FOR PUBLICATION, IT SHOULD NOT REPLACE THE EXPERTISE OF AN EXPERIENCED CRYSTALLOGRAPHIC REFEREE.

No syntax errors found. CIF dictionary Interpreting this report

Datablock: gvsu819f

```
Bond precision:  C-C = 0.0113 A          Wavelength=0.71073

Cell:            a=32.318(11)     b=12.538(4)         c=16.447(6)
                 alpha=90         beta=116.217(4)     gamma=90
Temperature:     173 K
```

	Calculated	Reported
Volume	5979(4)	5979(4)
Space group	C 2/c	C 1 2/c 1
Hall group	-C 2yc	-C 2yc
Moiety formula	C28 H60 N10 O34 P4 Sm2, 2(C2 H3 N)	C28 H60 N10 O34 P4 Sm2, 2(C2 H3 N)
Sum formula	C32 H66 N12 O34 P4 Sm2	C32 H66 N12 O34 P4 Sm2
Mr	1587.57	1587.54
Dx,g cm-3	1.764	1.764
Z	4	4
Mu (mm-1)	2.153	2.154
F000	3192.0	3192.0
F000’	3193.96	
h,k,lmax	40,15,20	40,15,20
Nref	6132	6120
Tmin,Tmax	0.575,0.838	0.657,0.745
Tmin’	0.543	

```
Correction method= # Reported T Limits: Tmin=0.657 Tmax=0.745
AbsCorr = MULTI-SCAN

Data completeness= 0.998          Theta(max)= 26.401

R(reflections)= 0.0532( 4315)     wR2(reflections)= 0.1487( 6120)

S = 1.058                    Npar= 402
```

The following ALERTS were generated. Each ALERT has the format
test-name_ALERT_alert-type_alert-level.
Click on the hyperlinks for more details of the test.

Alert level A

PLAT973_ALERT_2_A Check Calcd Positive Resid. Density on Sm1 3.88 eA-3

Author Response: This crystal was quite large, and diffracted very well Our hypotheses are that either (a) the absorption correction will not work well due to the high level of diffaction, or (b) that the crystal is twinned and high angle data intensity is too far off. The large residual positive density peak is near the metal, and the other large residual negative density peaks are compensating for this. We tried a number of different absorption corrections, but none improved the residual peaks.

Alert level B

PLAT094_ALERT_2_B Ratio of Maximum / Minimum Residual Density .... 4.45 Report

Author Response: This crystal was quite large, and diffracted very well Our hypotheses are that either (a) the absorption correction will not work well due to the high level of diffaction, or (b) that the crystal is twinned and high angle data intensity is too far off. The large residual positive density peak is near the metal, and the other large residual negative density peaks are compensating for this. We tried a number of different absorption corrections, but none improved the residual peaks.

PLAT971_ALERT_2_B Check Calcd Resid. Dens. 1.73A From C14 2.69 eA-3

Author Response: This crystal was quite large, and diffracted very well Our hypotheses are that either (a) the absorption correction will not work well due to the high level of diffaction, or (b) that the crystal is twinned and high angle data intensity is too far off. The large residual positive density peak is near the metal, and the other large residual negative density peaks are compensating for this. We tried a number of different absorption corrections, but none improved the residual peaks.

PLAT971_ALERT_2_B Check Calcd Resid. Dens. 1.35A From O14 2.55 eA-3

Author Response: This crystal was quite large, and diffracted very well Our hypotheses are that either (a) the absorption correction will not work well due to the high level of diffaction, or (b) that the crystal is twinned and high angle data intensity is too far off. The large residual positive density peak is near the metal, and the other large residual negative density peaks are compensating for this. We tried a number of different absorption corrections, but none improved the residual peaks.

Alert level C

```
PLAT220_ALERT_2_C NonSolvent Resd 1  C   Ueq(max) / Ueq(min) Range        3.3 Ratio
PLAT222_ALERT_3_C NonSolvent Resd 1  H   Uiso(max)/Uiso(min) Range        4.6 Ratio
PLAT241_ALERT_2_C High   MainMol Ueq as Compared to Neighbors of         O9 Check
PLAT241_ALERT_2_C High   MainMol Ueq as Compared to Neighbors of        C10 Check
PLAT244_ALERT_4_C Low    Solvent Ueq as Compared to Neighbors of        C1S Check
PLAT342_ALERT_3_C Low Bond Precision on  C-C Bonds ...............    0.01129 Ang.
PLAT911_ALERT_3_C Missing FCF Refl Between Thmin & STh/L=    0.600          2 Report
PLAT976_ALERT_2_C Check Calcd Resid. Dens.  0.89A   From O14          -0.61 eA-3
PLAT978_ALERT_2_C Number C-C Bonds with Positive Residual Density.          0 Info
```

Alert level G

```
PLAT002_ALERT_2_G Number of Distance or Angle Restraints on AtSite          2 Note
PLAT128_ALERT_4_G Alternate Setting for Input Space Group     C2/c       I2/a Note
PLAT172_ALERT_4_G The CIF-Embedded .res File Contains DFIX Records          1 Report
PLAT232_ALERT_2_G Hirshfeld Test Diff (M-X)  Sm1      --O10      .        5.7 s.u.
PLAT232_ALERT_2_G Hirshfeld Test Diff (M-X)  Sm1      --O12      .        6.0 s.u.
PLAT300_ALERT_4_G Atom Site Occupancy of H10A       Constrained at        0.5 Check
PLAT300_ALERT_4_G Atom Site Occupancy of H10B       Constrained at        0.5 Check
PLAT300_ALERT_4_G Atom Site Occupancy of H10C       Constrained at        0.5 Check
PLAT300_ALERT_4_G Atom Site Occupancy of H10D       Constrained at        0.5 Check
PLAT300_ALERT_4_G Atom Site Occupancy of H10E       Constrained at        0.5 Check
PLAT300_ALERT_4_G Atom Site Occupancy of H10F       Constrained at        0.5 Check
PLAT301_ALERT_3_G Main Residue  Disorder ..............(Resd  1  )          3% Note
PLAT720_ALERT_4_G Number of Unusual/Non-Standard Labels ..........          5 Note
PLAT793_ALERT_4_G Model has Chirality at P1          (Centro SPGR)          R Verify
PLAT794_ALERT_5_G Tentative Bond Valency for Sm1       (III)     .       3.52 Info
PLAT860_ALERT_3_G Number of Least-Squares Restraints .............          1 Note
PLAT910_ALERT_3_G Missing # of FCF Reflection(s) Below Theta(Min).          1 Note
PLAT912_ALERT_4_G Missing # of FCF Reflections Above STh/L=  0.600          9 Note
```

```
   1 ALERT level A = Most likely a serious problem - resolve or explain
   3 ALERT level B = A potentially serious problem, consider carefully
   9 ALERT level C = Check. Ensure it is not caused by an omission or oversight
  18 ALERT level G = General information/check it is not something unexpected

   0 ALERT type 1 CIF construction/syntax error, inconsistent or missing data
  12 ALERT type 2 Indicator that the structure model may be wrong or deficient
   6 ALERT type 3 Indicator that the structure quality may be low
  12 ALERT type 4 Improvement, methodology, query or suggestion
   1 ALERT type 5 Informative message, check
```

It is advisable to attempt to resolve as many as possible of the alerts in all categories. Often the minor alerts point to easily fixed oversights, errors and omissions in your CIF or refinement strategy, so attention to these fine details can be worthwhile. In order to resolve some of the more serious problems it may be necessary to carry out additional measurements or structure refinements. However, the purpose of your study may justify the reported deviations and the more serious of these should normally be commented upon in the discussion or experimental section of a paper or in the "special_details" fields of the CIF. checkCIF was carefully designed to identify outliers and unusual parameters, but every test has its limitations and alerts that are not important in a particular case may appear. Conversely, the absence of alerts does not guarantee there are no aspects of the results needing attention. It is up to the individual to critically assess their own results and, if necessary, seek expert advice.

Publication of your CIF in IUCr journals

A basic structural check has been run on your CIF. These basic checks will be run on all CIFs submitted for publication in IUCr journals (*Acta Crystallographica*, *Journal of Applied Crystallography*, *Journal of Synchrotron Radiation*); however, if you intend to submit to *Acta Crystallographica Section C* or *E* or *IUCrData*, you should make sure that full publication checks are run on the final version of your CIF prior to submission.

Publication of your CIF in other journals

Please refer to the *Notes for Authors* of the relevant journal for any special instructions relating to CIF submission.

PLATON version of 22/12/2019; check.def file version of 13/12/2019

Datablock gvsu819f - ellipsoid plot

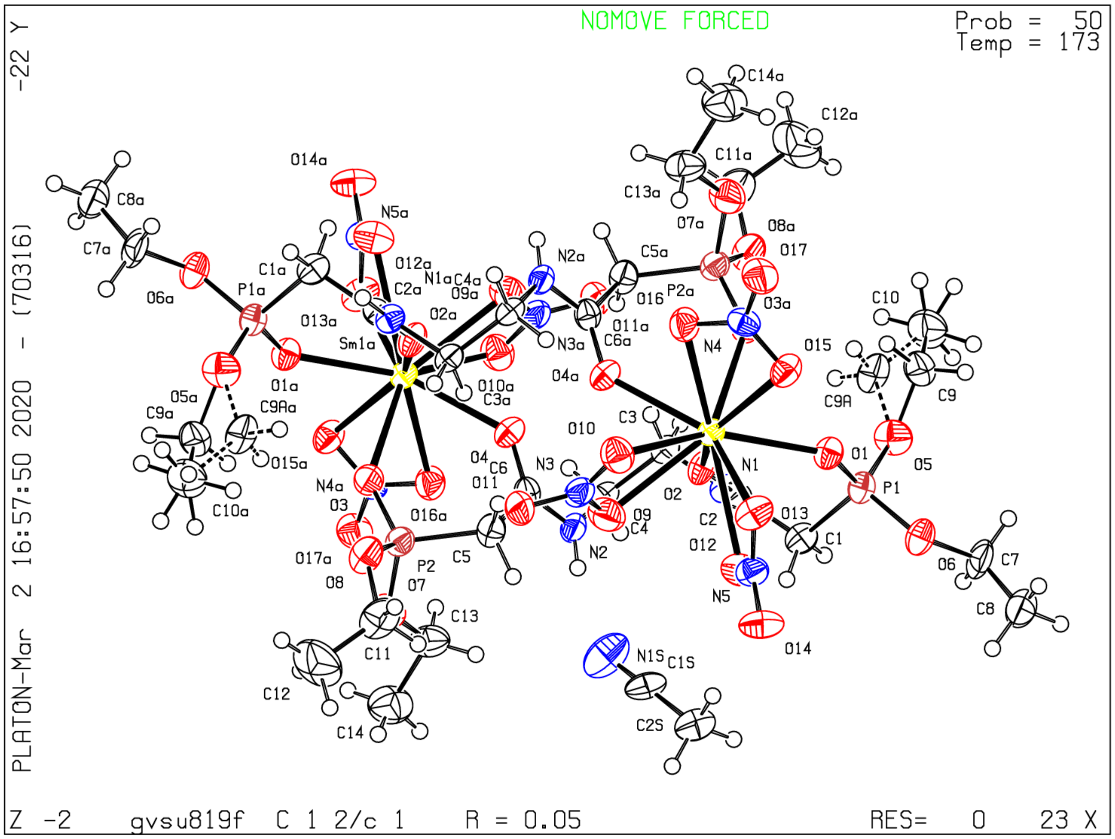

Supplement: Supplementary file 1 [file GVSU819F_checkcif.pdf › supp_files_rev2/check_cif_reports/GVSU819F_checkcif.pdf]
